# DNA moiré superlattices

**DOI:** 10.1038/s41565-025-01976-3

**Published:** 2025-07-17

**Authors:** Xinxin Jing, Nicolas Kroneberg, Andreas Peil, Benjamin Renz, Longjiang Ding, Tobias Heil, Katharina Hipp, Peter A. van Aken, Hao Yan, Na Liu

**Affiliations:** 1 2nd Physics Institute, University of Stuttgart, Stuttgart, Germany; 2 Max Planck Institute for Solid State Research, Stuttgart, Germany; 3 Electron Microscopy, Max Planck Institute for Biology Tübingen, Tübingen, Germany; 4 Biodesign Center for Molecular Design and Biomimetics, Arizona State University, Tempe, AZ USA

**Keywords:** DNA nanotechnology, Structural materials

## Abstract

Moiré superlattices have been extensively designed and implemented in atomic-scale van der Waals systems and submicrometre-scale photonic systems. However, bridging the structural gap between these scales has remained a substantial challenge. Here we demonstrate engineered DNA moiré superlattices with sublattice constants as small as ~2 nm and moiré periodicities spanning tens of nanometres. Using twisted DNA origami nanoseeds, we precisely control the layered registry of 2D microscale single-stranded tile DNA sublattices, achieving seed-defined twist angles with deviations below 2°, along with customizable interlayer spacing, stacking sequences and sublattice symmetries. The modularity of nucleation sites on the seeds enables synthetic control over the nucleation and growth pathways, resulting in a high bilayer fraction of 90%. Notably, we demonstrate a gradient moiré superlattice with a gradual variation in moiré periodicity, highlighting the potential of DNA nanotechnology to construct entirely new artificial structures and materials from the bottom up.

## Abstract (web summary)

Bottom-up, seeded epitaxial assembly using twisted DNA origami seeds directs the formation of 2D DNA lattices to create moiré superlattices, enabling a design space for programmable materials that combine molecular precision with mesoscale complexity.

## Main

**Fig. 1:**
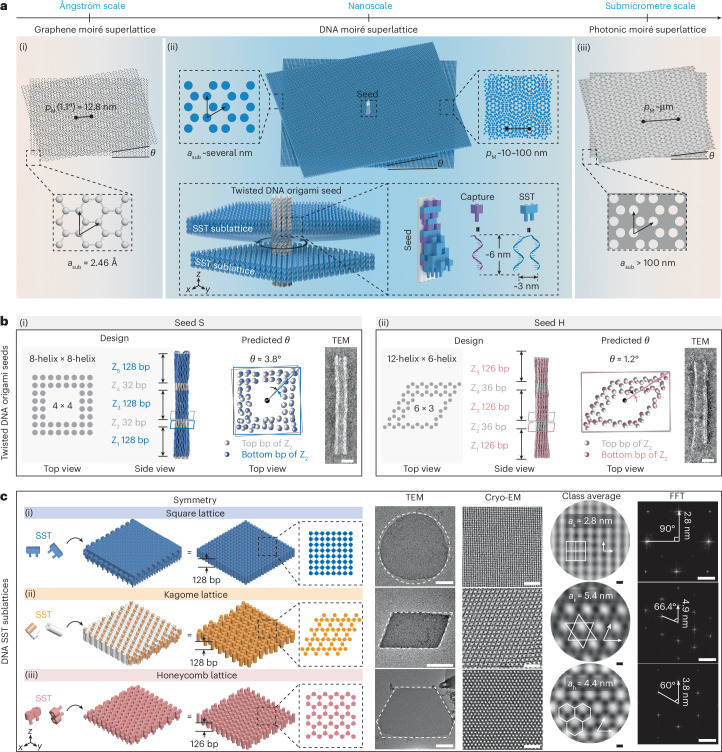
Building blocks of DNA moiré superlattices. **a**, Moiré superlattices across different length scales. (i) Graphene moiré superlattice with *a*_sub_ at the ångström scale. *a*_sub_ = 2.46 Å, *p*_M_ = 12.8 nm at *θ* = 1.1°. (ii) DNA moiré superlattice with *a*_sub_ at the nanoscale. *a*_sub_, ~several nanometres; *p*_M_, ~10–100 nm. Twisted DNA origami seed templates the growth of stacked DNA SST sublattices with a prescribed twist angle *θ*. Captures (purple) are extended from the origami seed. Each SST (blue) measures ~6 nm × 3 nm. (iii) Photonic moiré superlattice with *a*_sub_ at the submicrometre scale. *a*_sub_ > 100 nm, *p*_M_ ~μm. **b**, Twisted DNA origami seeds. Design schematic, *θ* prediction based on coarse-grained simulation, and TEM image of seed S (i) and seed H (ii). Scale bars, 20 nm. **c**, DNA SST sublattices with different lattice symmetries. Design schematics, TEM images (scale bars, 1 μm), cryo-EM images (scale bars, 20 nm), class averages (scale bars, 2 nm) and FFT patterns (scale bars, 0.2 nm^−1^) for square (i), kagome (ii) and honeycomb (iii) sublattices. Lattice constants, interplane spacings and interplane angles are marked in the corresponding class-average images and FFT patterns. Source data

Moiré patterns are commonly observed phenomena across all length scales. A moiré pattern arises from the spatial modulation of stacked sublattices, which have slightly different lattice constants (*a*_sub_) and/or orientations (*θ*)^1^. This pattern forms a superlattice with a moiré periodicity (*p*_M_), spanning multiple unit cells of the sublattices. For moiré superlattices composed of sublattices with lattice constants *a*_sub_ at the ångström scale, a prominent example is twisted bilayer graphene^2–6^ (Fig. 1a(i)). Specifically, when two graphene sheets (*a*_sub_ = 2.46 Å) are overlaid at a magic twist angle of 1.1°, the resulting moiré superlattice exhibits a much larger periodicity *p*_M_ = 12.8 nm, accompanied by substantial modifications to the electronic band structure. Remarkably, the emergence of flat electronic bands and correlated electronic states has enabled many intriguing quantum phenomena, laying the foundation for the burgeoning field of twistronics^7^. Meanwhile, the advent of twistronics has spurred the creation of its photonic analogues. In twisted nanophotonic systems, *a*_sub_ of the sublattices is typically at the submicrometre scale, ranging from tens to hundreds of nanometres^8,9^ (Fig. 1a(iii)). However, for both twistronics and twisted nanophotonic systems, meticulous fabrication steps, such as the transfer, stacking, twisting and alignment of sublattices, are often required to achieve target moiré superlattices^10,11^.

So far, the realization of moiré superlattices that comprise sublattices with *a*_sub_ of several nanometres remains highly challenging. At this intermediate length scale, the hurdles stem not only from the aforementioned technological complexities but also from the lack of accessible materials. For *a*_sub_ at the ångström scale, a variety of atomic lattices, such as van der Waals materials, are readily available^12^, while for *a*_sub_ at the submicrometre scale, a plethora of artificial lattices, such as metasurfaces, can be created using state-of-the-art fabrication techniques.

Here, we demonstrate a new class of moiré superlattices, which bridge the structural gap between the ångström and submicrometre length scales using bottom-up DNA nanotechnology^13–19^ (Fig. 1a(ii)). Featuring engineered symmetries, twist angles, moiré periodicities and stacking sequences, these DNA moiré superlattices have no natural counterparts, serving as programmable analogues to twisted van der Waals and photonic systems. Creating DNA moiré superlattices in twisted bilayers or multilayers requires the precise alignment of two-dimensional (2D) DNA sublattices (*a*_sub_ is approximately several nanometres) with small twist angles and nanoscale interlayer spacings. We achieve this spatial registry of the 2D sublattices using a common DNA nanoseed with an intrinsic twist. Two DNA-assembly strategies, DNA origami^16^ and DNA brick^19^ techniques, are used to construct the two key building blocks of DNA moiré superlattices: twisted DNA origami seeds and 2D DNA tile sublattices composed of single-stranded tiles (SSTs), respectively (Fig. 1a(ii)). The SSTs nucleate at prescribed binding segments along the twisted origami seed. The subsequent growth of the 2D SST sublattices occurs in the *x*–*y* plane, with their self-registry along the origami seed at designated *z* positions. The stacking of the SST sublattices thus follows the twist theme of the central origami seed, resulting in a moiré superlattice with a periodicity *p*_M_ of approximately tens of nanometres. Asymmetric seeds further enable gradient moiré periodicities, an architectural motif previously unattainable in DNA structures^20–22^.

## Twisted DNA origami seed and DNA SST sublattices

The first building block of a DNA moiré superlattice, the nanometre-sized seed, is a custom-shaped DNA origami bundle, composed of interconnected DNA helices. Two types of hollow seeds (seed S and seed H) are used as shown in Fig. 1b (see Supplementary Figs. 1–7 for the detailed design and calculation). Seed S incorporates different functional segments. Z_1_, Z_3_ and Z_5_, each 128 base pairs (bp; 43.5 nm) in height, serve as nucleation and growth regions for three SST sublattices. Z_2_ and Z_4_, each 32 bp (10.9 nm) in height, play a dual role. They define accurate interlayer spacings and introduce a right-handed twist of *θ* ≈ 3.8° between the adjacent SST sublattices. It is noteworthy that the twist angle *θ* of the DNA moiré superlattice is determined solely by the twisting segments Z_2_ and/or Z_4_, rather than reflecting the global twist of the seed. For seed H, the spacing and twisting segment (Z_2_ or Z_4_) is 36 bp (12.2 nm) in height, generating a right-handed twist of *θ* ≈ 1.2° along the *z* axis. The nucleation and growth segments (Z_1_, Z_3_ and Z_5_) are each 126 bp (42.8 nm) in height. Transmission electron microscopy (TEM) images of representative seed S and seed H shown in Fig. 1b align with our design specifications.

The second building block is the 2D, micrometre-sized SST sublattices with defined lattice symmetries and heights (Fig. 1c). Three representative lattice symmetries, square^19^ (128 bp), kagome (128 bp) and honeycomb (126 bp) monolayer lattices, matching the heights of their respective nucleation and growth segments, are assembled from sequence-specific SSTs mediated by local binding interactions. The kagome lattice can be viewed as a loosely packed square lattice with voids. The relief of the under-winding strain within the lattice results in a symmetry transformation from square to kagome. Details of the design and assembly protocols can be found in Supplementary Figs. 8–23.

TEM and cryogenic electron microscopy (cryo-EM) images in Fig. 1c confirm the lattice constants and symmetries of the assembled SST sublattices. The square lattice appears round rather than square, probably due to high under-winding strain resulting from the dense packing of SSTs. The kagome and honeycomb lattices exhibit rhomboid and asymmetric hexagonal shapes, creating well-defined edges that conform to their lattice symmetries, respectively (Supplementary Figs. 17 and 22). Class-average cryo-EM images reveal the lattice constants of these monolayer lattices: square *a*_s_ = 2.8 nm, kagome *a*_k_ = 5.4 nm and honeycomb *a*_h_ = 4.4 nm. The fast Fourier transform (FFT) patterns provide the corresponding interplanar distances and angles. When measured on dried TEM grids, the lattice constants are slightly smaller, with values of 2.2 nm for the square, 4.4 nm for the kagome and 3.5 nm for the honeycomb, probably due to structural shrinkage upon drying (Supplementary Figs. 11, 16 and 21). Similarly, the height of these monolayer lattices, ~40 nm as measured by atomic force microscopy (AFM) in air, is also slightly shorter than the designed values (Supplementary Figs. 12, 18 and 23).

## Synthetic regulation of nucleation pathways

**Fig. 2:**
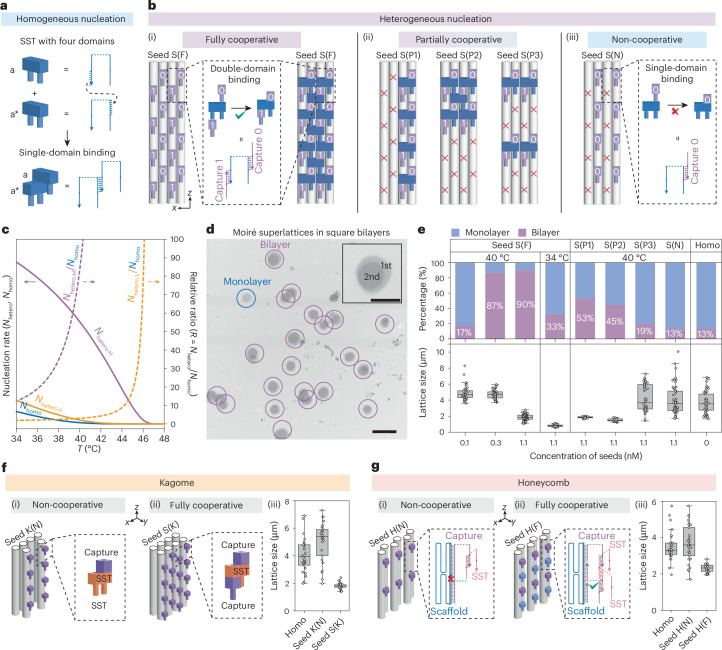
Synthetic regulation of nucleation pathways. **a**, Homogeneous (unseeded) nucleation of SSTs via single-domain binding. **b**, Heterogenous (seeded) nucleation of SSTs in different nucleation modes, enabled by variations in modular capture numbers and patterns on the origami seed. Half of one lateral surface (Z_1_, Z_3_ or Z_5_) of seed S is shown. (i) Fully cooperative mode via double-domain binding (seed S(F)). (ii) Partially cooperative mode: seed S(P1), seed S(P2) and seed S(P3) with discontinuous distributions of capture pairs. The red cross indicates lack of capture. (iii) Non-cooperative mode: seed S(N) with half of the captures (0 or 1). **c**, Nucleation rate (*N*_hetero_, *N*_homo_; solid lines) and relative ratio (*R* = *N*_hetero_/*N*_homo_; dashed lines) as a function of temperature under low- and high-wetting conditions. **d**, Bright-field optical microscopy image of DNA moiré superlattices in square lattice bilayers at 0.3 nM seed S(F). Scale bar, 10 µm. Inset: enlarged view. Scale bar, 5 µm. **e**, Statistical analysis of the seeded bilayer yields and lattice sizes with different seeds at varying seed concentrations and incubation temperatures. Homogeneous nucleation serves as the reference. From left to right, *N* (bilayer fraction) = 70, 126, 113, 193, 256, 252, 118, 46 and 84, respectively; *N* (lattice size) = 42, 46, 73, 30, 20, 20, 44, 53 and 45, respectively. **f**, (i) Schematic of seed K(N). (ii) Schematic of seed S(K) for the kagome DNA moiré superlattice. (iii) Statistical analysis of the lattice size. *N* = 30. **g**, (i) Schematic of seed H(N). (ii) Schematic of seed H(F) for the honeycomb DNA moiré superlattice. (iii) Statistical analysis of the lattice size. *N* = 30. For the box plots, the central line is the median, the minima and maxima of the box extend to the 25th and 75th percentiles, and whiskers extend to data points within 1.5 × interquartile range of Q1 and Q3. Source data

The seeded growth of DNA moiré superlattices in twisted square bilayers will serve as a model system to investigate the key factors regulating the nucleation pathways. Homogeneous (unseeded) nucleation of 32-nt SSTs, each containing four 8-nt domains, occurs slowly via single-domain binding, followed by rapid growth to form monolayer lattices^19,23^ (Fig. 2a). By contrast, heterogeneous (seeded) nucleation is guided by capture strands on the origami seed (seed S), which define modular SST nucleation sites. The number and patterns of these sites influence surface wetting behaviour, consistent with classical nucleation theory^24^. To probe different nucleation modes, we design three distinct capture patterns: fully cooperative (seed S(F)), partially cooperative (seed S(P1–P3)) and non-cooperative (seed S(N)) (Fig. 2b; see Supplementary Fig. 24 for detailed design). Seed S(F) features full capture pairs, each consisting of capture 0 and capture 1 that enable double-domain SST binding^25^ (Fig. 2b(i)). All-atom molecular dynamics simulations show that this double-domain binding stabilizes SST positioning and conformation at the seeded interface by mitigating electrostatic repulsion, thereby promoting subsequent SST binding (Supplementary Fig. 25). In seed S(P1–P3), partial removal of capture pairs disrupts their continuity along the *x* and/or *z* axes, allowing double-domain binding only at specific sites (Fig. 2b(ii)). In seed S(N), the omission of either all capture 1s or capture 0s abolishes the formation of capture pairs, eliminating double-domain binding and cooperativity (Fig. 2b(iii)). Using classical nucleation theory^26^, we qualitatively correlate these three nucleation modes with wetting phenomena (Fig. 2c; see Supplementary Figs. 26 and 27 for detailed calculations). Overall, a positive correlation between *N*_hetero_/*N*_homo_ and *T* suggests that elevated temperatures accentuate differences between heterogeneous and homogeneous nucleation pathways. In low-wetting (that is, partially or non-cooperative) mode, *N*_hetero_/*N*_homo_ peaks near the melting temperature, where absolute nucleation rates are low. By contrast, in high-wetting (that is, fully cooperative) mode, *N*_hetero_/*N*_homo_ remains elevated across a broad temperature range, supporting highly selective heterogeneous nucleation even at lower temperatures without compromising the absolute nucleation rate.

Optimal assembly conditions are first determined by experimentally investigating the seeded growth of DNA moiré superlattices in twisted square bilayers under the fully cooperative mode. Purified seed S(F) constructs with captures on Z_1_ and Z_3_ are tested at concentrations ranging from 0.1 to 1.1 nM. They are mixed with 1.2 µM SSTs and 40 mM Mg^2+^, preheated at 45 °C for 10 min and incubated at 40 °C, a temperature at which homogeneous nucleation and growth are effectively suppressed for at least 12 h (Supplementary Fig. 28). Figure 2d shows a representative optical microscopy image of DNA moiré superlattices formed at a seed concentration of 0.3 nM, highlighting bilayers (purple) and monolayers (blue). The predominance of bilayers confirms efficient seeded growth (Supplementary Figs. 29 and 30). Statistical analysis reveals that the bilayer fraction increases with seed concentration, reaching up to 90% at 1.1 nM (Fig. 2e and Supplementary Figs. 31–33). Meanwhile, higher seed concentrations result in more nuclei, yielding smaller lattices: 4.9 ± 0.9 µm at 0.1 nM, 4.7 ± 0.6 µm at 0.3 nM and 1.9 ± 0.4 µm at 1.1 nM. Notably, lattice size variability also decreases from ±1.4 µm (unseeded) to ±0.4 µm at 1.1 nM, consistent with previous observations in one-dimensional seeded growth of DNA nanotubes^27^. However, when incubated at 34 °C, a temperature previously optimal for square sublattices, the bilayer fraction at 1.1 nM drops to 33% and the lattice size shrinks to 0.8 ± 0.1 µm (Fig. 2e and Supplementary Fig. 34). This trend aligns with Fig. 2c, where lower *R* at reduced temperatures leads to increased homogeneous nucleation, resulting in a decreased bilayer fraction and smaller lattices.

Using the optimized conditions (1.1 nM seed, 40 °C incubation), we investigate how engineered capture patterns regulate nucleation pathways (Fig. 2e and Supplementary Figs. 35–38). In the partially cooperative mode, seeds S(P1), S(P2) and S(P3) contain identical numbers of capture pairs but differ in spatial arrangement, resulting in distinct bilayer fractions and lattice size distributions. Specifically, seed S(P1) and seed S(P2) each yield ~50% bilayers with narrow size distributions, indicating a partial loss of the seeding effect compared with seed S(F). By contrast, seed S(P3) shows a reduced bilayer fraction (19%) and a broader size distribution (4.2 ± 1.6 µm), closely resembling homogeneous nucleation (13% bilayers, 3.8 ± 1.4 µm lattice size). These results highlight the critical importance of *z*-axis continuity of capture pairs for efficient seeded nucleation. In the non-cooperative mode, seed S(N), which retains the same number of captures as those three seeds in the partially cooperative mode but lacks any capture pairs, further reduces the bilayer fraction to 13% and yields an even broader size distribution (4.0 ± 1.8 µm). TEM imaging confirms no SST attachment on seed S(P3) or seed S(N) during early incubation. By contrast, seed S(F) exhibits clear SST binding, characterized by a ‘liquid spreading’ phenotype at 5 min, followed by layer-by-layer growth at 1 h and 2.5 h, and perfect sublattice alignment with the seed by 24 h (Supplementary Figs. 39 and 40). Taken together, these findings demonstrate that effective nucleation and growth of SSTs on the lateral surface of the origami seed require double-domain binding enabled by capture pairs^25^. Beyond this, both a critical number and a specific arrangement of capture pairs are essential for mitigating electrostatic repulsion at component interfaces and ensuring successful seeded nucleation.

Our design principle for seeded nucleation is generalizable to DNA moiré superlattices with diverse lattice symmetries. For kagome symmetry, an intuitive design using a kagome-array DNA origami seed (seed K(N); Fig. 2f(i) and Supplementary Fig. 41) lacks capture pairs, resulting in the non-cooperative mode and, thus, no seeding effect. This is indicated by the resulting lattice size (5.1 ± 1.4 µm), which is comparable to that from homogeneous nucleation (4.0 ± 1.1 µm) (Fig. 2f(iii) and Supplementary Fig. 42). To enable cooperative seeding, we instead use a square-array seed (seed S(K)), leveraging the geometric compatibility between square and kagome symmetries (Fig. 2f(ii) and Supplementary Fig. 41). This design supports the fully cooperative mode and yields a much narrower lattice size distribution (1.8 ± 0.2 µm), confirming effective seeded growth (Fig. 2f(iii) and Supplementary Fig. 42). Interestingly, seed S(K) undergoes a square-to-rhombohedral deformation that conforms to the kagome sublattice symmetry (Supplementary Fig. 43). We further extend our design principle to honeycomb symmetry. A honeycomb array seed (seed H(N)) produces discontinuous capture patterns along the *z* axis, resulting in non-cooperative nucleation (Fig. 2g(i) and Supplementary Fig. 44). To address this, we introduce staple captures with parallel crossovers that function analogously to the non-extendable scaffold captures, enabling the fully cooperative mode (seed H(F)) (Fig. 2g(ii) and Supplementary Fig. 44). This design yields the narrowest lattice size distribution (1.7 ± 0.3 µm), outperforming both non-cooperative seeding (3.3 ± 1.2 µm) and homogeneous nucleation (3.1 ± 0.9 µm) (Fig. 2g(iii) and Supplementary Figs. 45 and 46). These results validate the generality of our seeded nucleation framework across different lattice symmetries.

## DNA moiré superlattices

**Fig. 3:**
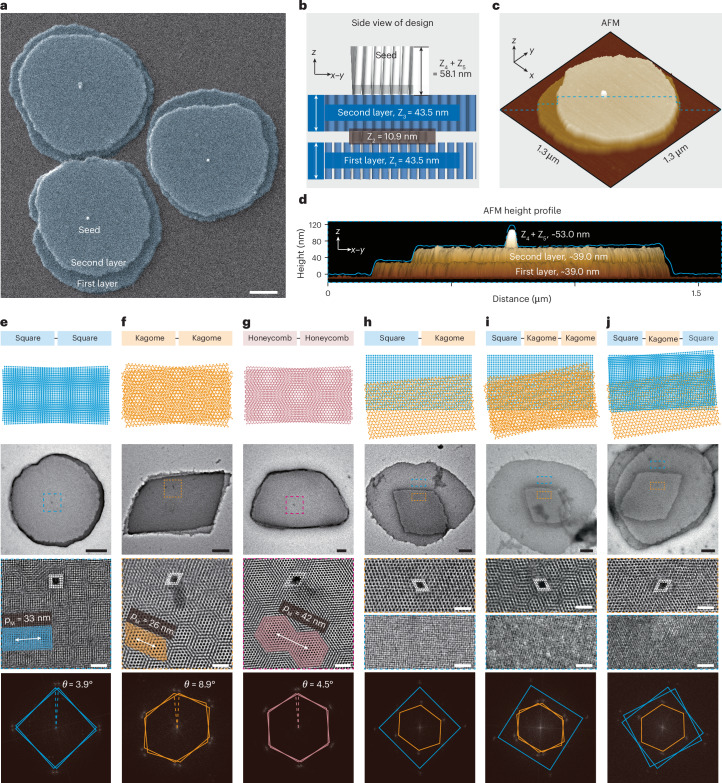
Engineered DNA moiré superlattices. **a**, An SEM image of the DNA moiré superlattices in square lattice bilayers. Scale bar, 200 nm. **b**, A side-view schematic of the square lattice bilayer. **c**, An AFM image of a representative square lattice bilayer. **d**, The AFM height profile of the square lattice bilayer. **e**–**g**, TEM images, FFT patterns, simulated seeds and superlattice patterns of square (**e**), kagome (**f**) and honeycomb (**g**) lattice bilayers. In each case, the experimental TEM image is overlaid with a schematic of the origami seed and superlattice pattern. Scale bars, 200 nm (low magnification) and 20 nm (high magnification). **h**–**j**, TEM images, FFT patterns and simulated seeds of hybrid bilayer (**h**) and trilayer (**i**,**j**) systems with various lattice symmetries. Scale bars, 200 nm (low magnification) and 20 nm (high magnification). Lattice symmetries of the constituent layers are depicted in the corresponding schematics for each superlattice in **e**–**j**. Source data

Figure 3a shows a representative scanning electron microscopy (SEM) image of DNA moiré superlattices, featuring two square sublattices grown from the Z_1_ and Z_3_ segments of seed S(F) (Supplementary Fig. 47). Two stacked, micrometre-sized SST monolayers are threaded together by a central nanoseed, visible as a white bulge, demonstrating successful seeded growth of the bilayer (see Supplementary Fig. 48 for more SEM images). The height profile of the DNA moiré superlattices is characterized using AFM (Fig. 3b–d and Supplementary Fig. 49). Both monolayers measure ~39.0 nm in height, slightly lower than the designed value of 43.5 nm, probably due to the drying process required for AFM imaging. The interlayer Z_2_ segment (nominally 10.9 nm) is undetectable. This suggests that the 2D micrometre-sized sublattices come into contact, forming a gapless bilayer upon drying. The capture-free segments Z_4_ + Z_5_ (Fig. 3a, white bulge) have a measured total height of ~53.0 nm, closely matching the designed height of 58.1 nm.

We further characterize the moiré pattern, twist angle *θ* and superlattice periodicity *p*_M_ in twisted square bilayers using TEM (Fig. 3e). The overview TEM image clearly displays the origami seed and two constituent sublattices, while a high-magnification view highlights the hollow seed seamlessly integrated into the superlattice. A prominent moiré pattern is observed, with *p*_M_ ≈ 33 nm, much larger than the sublattice spacing (*a*_sub_ ≈ 2.2 nm), corresponding to a twist angle of *θ* ≈ 3.9° based on the relation *p*_M_ =*a*_sub_/2sin(*θ*/2). This agrees well with the FFT analysis and the simulated *θ* ≈ 3.8°. Statistical analysis (*N* = 20) yields an average *θ* = 5.0° ± 1.8° (Supplementary Figs. 50–55), with deviations probably due to heterogeneity in seed twist and drying-induced structural deformation. Meanwhile, the close agreement supports that *θ* is primarily determined by the twisting segment Z_2_. The 2D micrometre-sized DNA sublattices possess a high torsional persistence length exceeding 10^7^ nm (ref. ^28^) (Supplementary Fig. 13), allowing the initially twisted Z_1_ and Z_3_ segments to relax and conform to the untwisted characteristics of the sublattices.

We also showcase a library of twisted bilayer and trilayer structures with various symmetries. Figure 3f and Fig. 3g show the TEM images of twisted kagome and honeycomb bilayers seeded with seed S(K) and seed H(F), respectively. High-magnification TEM images reveal moiré patterns with *p*_M_ ≈ 26 nm (corresponding to *θ* = 8.9° with *a*_sub_ = 4.4 nm) for the kagome bilayer and *p*_M_ ≈ 42 nm (corresponding to *θ* = 4.5° with *a*_sub_ = 3.5 nm) for the honeycomb bilayer. The statistical twist angles are 5.2° ± 2.0° for the kagome bilayer and 2.6° ± 1.2° for the honeycomb bilayer, which agree well with the predictions (Supplementary Figs. 56–60). A notable feature of the twisted bilayers with identical sublattices (square–square, kagome–kagome and honeycomb–honeycomb) is the nearly complete overlap of the two monolayers, highlighting the synergy achieved through seeded growth in a one-step assembly process. Furthermore, we also construct twisted trilayers in all three symmetries using seeds with extended captures from Z_1_, Z_3_ and Z_5_ segments (Supplementary Figs. 61 and 62). The twist angles between neighbouring stacked monolayers are almost identical, as they are defined by the Z_2_ and Z_4_ segments of the respective seeds.

Figure 3h–j shows the TEM images of twisted hybrid bilayers and trilayers comprising different sublattices. As seed S can template both square and kagome sublattices, a variety of bilayer and trilayer configurations can be achieved by altering the stacking sequences of the monolayers (Supplementary Fig. 63). Representative configurations, such as square–kagome, square–kagome–kagome and square–kagome–square are constructed. The kagome sublattices are readily distinguishable by their distinct shapes and smaller sizes compared with the square sublattices. The high-magnification TEM images and corresponding FFT patterns further confirm the successful stacking of square and kagome sublattices in the designated sequences (see Supplementary Figs. 64–66 for more TEM images).

## DNA gradient moiré superlattices

**Fig. 4:**
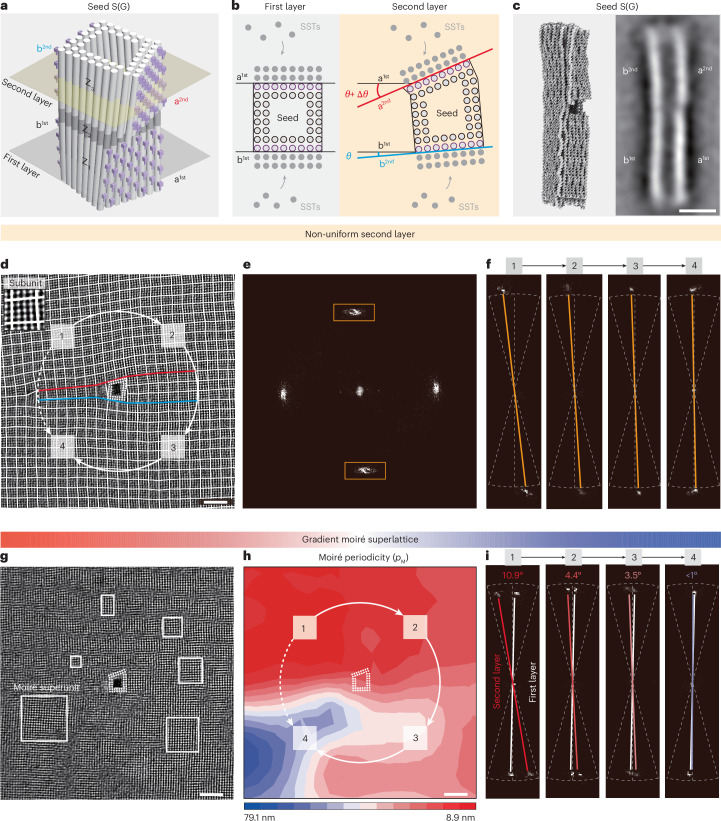
Gradient DNA moiré superlattice. **a**,**b**, A schematic of seed S(G) for gradient DNA moiré superlattices in square lattice bilayers. Z_3_ has a trapezoid-shaped cross-section with two non-parallel growth surfaces, which induce a gradual change in lattice orientation and, thus, in moiré periodicity *p*_M_. **c**, Simulation and class-average TEM image of seed S(G). The growth surfaces are marked in the TEM image. Scale bar, 20 nm. **d**–**f**, Structural characterizations of the seeded second layer on Z_3_ of seed S(G). **d**, TEM image analysed by marking the SST units, each comprising 4-helix × 4-helix (white boxes). The arrows highlight the change in lattice orientation. The solid arrows indicate a gradual clockwise change, while the dashed arrow indicates a sharp counterclockwise change. Red and blue lines indicate surface a^2nd^ and surface b^2nd^, respectively. Scale bar, 20 nm. **e**,**f**, FFT patterns for the overall (**e**) and selected (**f**) areas. **g**–**i**, Structural characterizations of the gradient DNA moiré superlattice. **g**, A TEM image with boxes highlighting the gradual changing moiré superunits. Scale bar, 20 nm. **h**, A heat map of the moiré superunits with arrows indicating the direction from smaller to larger *p*_M_. The solid arrows indicate a gradual clockwise change, while the dashed arrow indicates a sharp counterclockwise change. Scale bar, 20 nm. **i**, FFT patterns for the selected areas with white lines corresponding to the first layer and coloured lines corresponding to the second layer. The respective twist angles are shown on top. Source data

Next, we demonstrate the construction of gradient moiré superlattices, a system previously unexplored at this length scale^29^. These moiré superlattices feature gradual *p*_M_ variations in the 2D plane, enabling spatial modulation of moiré patterns. This is achieved using an asymmetric origami seed, termed seed S(G), that templates two square SST sublattices, while gradually altering their relative lattice orientation. Seed S(G) contains three functional regions (Fig. 4a,b and Supplementary Fig. 67). Z_1_, which has a square-shaped cross-section with two parallel growth surfaces (a^1st^ and b^1st^), serves as the growth segment for a uniform square sublattice (first layer). Z_3_, which has a trapezoid-shaped cross-section with two non-parallel growth surfaces (tilted a^2nd^ and non-tilted b^2nd^), acts as the growth segment for a non-uniform square sublattice (second layer). Z_2_ functions as the spacing and twist segment, introducing a twist angle of *θ* ≈ 2.8° between surface b^1st^ and surface b^2nd^ with a predicted *p*_M_ ≈ 45.0 nm. The tilted surface a^2nd^ contributes an additional Δ*θ* ≈ 10.9°, giving rise to a total twist of *θ* + Δ*θ* ≈ 13.7° relative to surface a^1st^ (Supplementary Fig. 68) with a predicted *p*_M_ ≈ 9.2 nm.

Figure 4c shows both the simulated result and the TEM image of the origami seed S(G), featuring its asymmetric geometry (Supplementary Fig. 69). Figure 4d–f displays an enlarged TEM image of a representative seeded second layer along with its corresponding FFT patterns (see overview TEM image in Supplementary Fig. 70). The non-parallel growth surfaces, a^2nd^ and b^2nd^, induce a gradual change in subunit orientation in the clockwise direction (solid arrows) and a sharp change in the counterclockwise direction (dashed arrow). This evolution in orientation is more evident in the FFT patterns, where intensity maxima appear smeared (highlighted by solid orange boxes) rather than as discrete high-intensity points, reflecting the mixing of different lattice orientations on the second layer (Fig. 4e). The twisting of the subunit orientation decreases progressively from area 1 to area 4, as shown by the shifts in the discrete intensity peaks (Fig. 4f). Importantly, at the interface between the red and blue lines, the SST subunits adjust their orientations to enable seamless intersubunit binding, avoiding the formation of twin boundaries and incomplete subunits (Supplementary Fig. 70). This minimizes domain mismatches among SSTs and reduces the system’s free energy.

Figure 4g shows the TEM image of the seeded gradient moiré superlattice, with representative moiré superunits outlined in white boxes (see overview TEM image in Supplementary Fig. 71). The gradient in *p*_M_ is more clearly visualized in the heat map (Fig. 4h) derived from the experimental data in Fig. 4g. As the subunit orientation twist decreases clockwise within the second layer, *p*_M_ increases clockwise around the seed accordingly, ranging from 8.9 nm to 79.1 nm. This gradient increase in *p*_M_ arises from a corresponding decrease in twist angles, as confirmed by the FFT patterns in Fig. 4i. Additional gradient moiré superlattices are shown in Supplementary Fig. 72, demonstrating the robustness of our approach.

## Conclusions

In this Article, we have introduced seeded epitaxial assembly in DNA nanotechnology, fundamentally diverging from established epitaxial paradigms. Conventional epitaxy^30^, hypotaxy^31^ and graphoepitaxy^32^ typically rely on lattice-matched crystalline templates or predefined surface patterns to direct structural growth. However, moiré-patterned templates with *a*_sub_ at the nanoscale are non-existent, necessitating a new assembly scheme. Central to our approach are modular capture strands positioned along the lateral surface of the twisted DNA origami seed. These captures guide the nucleation of SSTs, which then assemble layer by layer into individual SST sublattices threaded by the common seed. Crucially, the resulting moiré superlattice is oriented orthogonally to the seed’s lateral surface, different from conventional epitaxy, where growth aligns with the underlying lattice or pattern.

Beyond its methodological impact, our approach unlocks the mesoscopic regime of moiré superlattices (*a*_sub_ of approximately several nanometres), where phononic crystals^33,34^ with tailored dispersion and topological properties become feasible. While DNA itself is suboptimal for direct phonon control, silicification^35,36^ and other material conversion strategies^37,38^ could transform DNA moiré superlattices into rigid architectures suitable for phonon engineering. Also, the superlattices can serve as addressable molecular templates for positioning inorganic materials, such as metallic nanoparticles, fluorophores and quantum dots, among others, creating photonic devices with tailored optical properties^39^. In addition, DNA origami seeds can be deterministically positioned on substrates via DNA origami placement^40^. Integrating the seeded epitaxial growth with top-down nanofabrication could enable site-specific assembly of moiré superlattices at predefined locations on chip, a level of spatial control that remains challenging to achieve with transition-metal dichalcogenides or semiconductors.

## Methods

### Materials

Staple strands for the DNA origami seeds were purchased from Sigma-Aldrich (desalt purification). SST strands for the SST sublattices were purchased from Sangon Biotech (PAGE purification). The sequences of all strands can be found in Supplementary Tables 1–6. Scaffold DNA strands (p7560, CS3-L and CS4) were purchased from Tilibit Nanosystems. All DNA strands were stored at −20 °C after being dissolved in ultrapure water. Other chemicals were purchased from Sigma-Aldrich. Carbon-coated TEM grids were purchased from Ted Pella. Quantifoil grids were purchased from Quantifoil Micro Tools GmbH. AFM tips were purchased from Bruker.

### Preparation of DNA origami seeds

DNA origami seeds were designed using caDNAno software. Seed S is a DNA origami bundle consisting of 48 helices (8-helix × 8-helix rim, 4-helix × 4-helix pore) arranged in a square array (Fig. 1b(i)). Seed H is a DNA origami bundle composed of 54 helices (12-helix × 6-helix rim, 6-helix × 3-helix pore) arranged in a honeycomb array (Fig. 1b(ii)). The hollow seed design facilitates the accurate identification of the seed within a DNA moiré superlattice for microscopy characterizations. The twisted geometries of the DNA origami seeds and the twist angles of the Z_2_ (or Z_4_) segments were simulated using the SNUPI program^28^. The simulation parameters are provided in Supplementary Fig. 3. Position vectors on the seeds were calculated on the basis of coordinates extracted from the SNUPI simulation output files. To assemble the DNA origami seeds, staple strands were mixed with scaffold strands in a molar ratio of 10:1 in TE-MgCl_2_ buffer (10 mM Tris, 1 mM EDTA and 20 mM MgCl_2_, pH 7.8). The mixture was then annealed in a PCR thermocycler using the following protocol: 65 °C for 20 min; from 60 °C to 40 °C at 40 min per degree Celsius; and from 40 °C to 25 °C at 15 min per degree Celsius.

### Agarose gel analysis and sample purification

The DNA origami seeds were electrophoresed on a 1.0 % agarose gel containing 1× GelRed in 0.5× TBE buffer with 11 mM Mg^2+^ for 3 h at 80 V with ice cooling. The target band was excised from the gel and squeezed between two glass slides. The concentration of the purified DNA origami seeds was determined using ultraviolet–visible absorption spectroscopy. All purified DNA origami seeds were stored at 4 °C before further use.

### Growth of unseeded DNA sublattice monolayers

The square and honeycomb lattices are created using 32-nt SSTs with four 8-nt domains and 36-nt SSTs with four 9-nt domains, respectively. The kagome lattice is formed using two 32-nt SST variants: one adopts a U-shape, as in the square lattice, while the other is linear, comprising three domains (8 nt, 16 nt and 8 nt). To form the lattice monolayers, the SST strands were mixed in an equimolar stoichiometric ratio from a 200 µM stock in TE buffer (10 mM Tris and 1 mM EDTA, pH 7.8) supplemented with 40 mM MgCl_2_. The mixture was then annealed in a PCR thermocycler using the following protocol: 65 °C for 20 min; 48 °C to 47 °C at 12 h per degree Celsius for the honeycomb lattice; 44 °C to 43 °C at 12 h per degree Celsius for the kagome lattice; and 39 °C to 38 °C at 12 h per degree Celsius for the square lattice.

### Growth of seeded monolayers, bilayers and trilayers

The SST strands (1.2 µM), purified DNA origami seeds (0.1–1.1 nM) and MgCl_2_ (final concentration of 40 mM) were separately preheated for 5 min at proper temperatures (honeycomb, 50 °C; kagome, 48 °C; square, 45 °C). They were then mixed quickly to avoid temperature fluctuations and incubated for 5 min. Finally, seeded growth proceeded at the designated nucleation and growth temperatures.

### Dynamic light scattering measurements of homogeneous nucleation kinetics

Dynamic light scattering experiments were conducted using Zetasizer Pro (Malvern Instruments). The SST strands were pipetted into a cuvette, and the size of individual SST strands was measured. To obtain their nucleation kinetics, the SST strands and 40 mM MgCl_2_ were preheated for 5 min and then mixed. Measurements were taken immediately on the mixture at 15-s intervals.

### Optical microscopy imaging

A 1 cm × 1 cm silicon wafer was cleaned sequentially with Milli-Q water, acetone and ethanol. It was then dried using nitrogen gas. Afterwards, the wafer was treated with plasma cleaning (30 mA, 5 min) to ensure a hydrophilic surface. Tenfold diluted DNA sublattices or superlattices were absorbed for 1 h onto the freshly prepared silicon wafer. Then,75% (vol/vol), 90% (vol/vol) and 100% (vol/vol) ethanol was sequentially used to wash the surface after absorption. Finally, the wafer was dried using nitrogen gas.

### SEM imaging

Tenfold diluted DNA sublattices or superlattices were absorbed for 1 h onto the freshly prepared 1 cm × 1 cm silicon wafer. The wafer was then stained for 30 s using a 2% aqueous uranyl formate solution containing 25 mM NaOH. Then, 75% (vol/vol), 90% (vol/vol) and 100% (vol/vol) ethanol was sequentially used to wash the surface after absorption. Finally, the wafer was dried using nitrogen gas. SEM imaging was performed using Raith eLine Plus.

### AFM imaging

The silicon wafers with stained DNA samples were directly used for AFM imaging with a ScanAsyst-air tip in air mode.

### TEM imaging

Five microlitres of purified DNA origami seeds (1 nM) or tenfold diluted DNA sublattices or superlattices were absorbed for 10 min onto a glow discharged, carbon-coated TEM grid. The grids were then stained for 10 s using a 2% aqueous uranyl formate solution containing 25 mM NaOH. TEM imaging was performed using Phillip CM 200 TEM operated at 200 kV. STEM images were performed using JEOL JEM-ARM200F operated at 60 kV, equipped with a cold field-emission gun and a probe Cs corrector (DCOR, CEOS GmbH).

### Cryo-EM characterization

Three microlitres of DNA lattices were pipetted onto glow-discharged Quantifoil grids. The sample was plunge-frozen using a Vitrobot Mark IV at 20 °C and humidity of 90%, with a wait time of 0 s, blot time of 6 s, blot force of −1 and drain time of 0 s. Imaging was performed using Talos Arctica (Thermo Fisher Scientific) operated at 200 kV, equipped with a Falcon III detector. Class-average cryo-EM images were obtained using the EMAN2 software package, version 2.99.47.

### All-atom molecular dynamics simulations

Molecular dynamics simulations were performed using the Gromacs2021 software package. The AMBER99sb force field was used to describe the interactions within the system. The simulation box was constructed using Gromacs, with all components of the system being properly positioned according to the requirements. The box was then filled with water, utilizing the TIP3P water model. Mg^2+^ and Cl^−^ were added to neutralize the system, ensuring that it was overall charge neutral. Before the production run, the system underwent energy minimization followed by a 100-ps equilibration simulation to allow proper relaxation. The production molecular dynamics simulation used the leapfrog algorithm to integrate Newton’s equations of motion, with an integration time step of 0.002 ps and a total of 50,000,000 steps, resulting in a total simulation time of 100 ns. The V-rescale thermostat was used to maintain the simulation temperature at 318.15 K, and the Parrinello–Rahman barostat was applied to keep the pressure constant at 1.0 bar. The Verlet scheme was used for neighbour searching. The cut-off radius for Coulombic interactions was set to 1.2 nm, with the long-range electrostatic interactions being corrected using the particle mesh Ewald method.

### Statistics and reproducibility

No statistical method was used to predetermine sample size. No data were excluded from the analyses. The experiments were not randomized. The investigators were not blinded to allocation during experiments and outcome assessment.

## Online content

Any methods, additional references, Nature Portfolio reporting summaries, source data, extended data, supplementary information, acknowledgements, peer review information; details of author contributions and competing interests; and statements of data and code availability are available at 10.1038/s41565-025-01976-3.

## Supplementary information

Supplementary Information Supplementary Figs. 1–72 and Tables 1–6.

Supplementary Data 1–15 Supplementary Data 1. Lattice size of the square SST sublattices within different temperature ranges. Supplementary Data 2. Height profiles of SST sublattices with different symmetries. Supplementary Data 3. Trajectory PDB files of molecular simulations. Supplementary Data 4. Electrostatic interaction energies between SST and seed with different capture designs. Supplementary Data 5. Dynamic light scattering (DLS) size of square SST sublattices at initial nucleation. Supplementary Data 6. Twisted angle statistics of DNA moiré superlattices. Supplementary Data 7. Seed position statistics of DNA moiré superlattices. Supplementary Data 8. Height profile of square bilayer DNA moiré superlattices with Z_2_ = 0 bp. Supplementary Data 9. FFT patterns of square bilayer DNA moiré superlattices. Supplementary Data 10. FFT patterns of square bilayer DNA moiré superlattices with Z_2_ = 0 bp. Supplementary Data 11. FFT patterns of kagome bilayer DNA moiré superlattices. Supplementary Data 12. FFT patterns of honeycomb bilayer DNA moiré superlattices. Supplementary Data 13. FFT patterns of square bilayers formed via random monolayer overlap in unseeded growth. Supplementary Data 14. FFT patterns of kagome bilayers formed via random monolayer overlap in unseeded growth. Supplementary Data 15. FFT patterns of honeycomb bilayers formed via random monolayer overlap in unseeded growth.

## Source data

Source Data Figs. 1–4 Source Data Fig. 1. Statistical source data. Source Data Fig. 2. Statistical source data. Source Data Fig. 3. Statistical source data. Source Data Fig. 4. Statistical source data.

## Data Availability

All the data that support the findings of this study are available within the article and its Supplementary Information, and from the corresponding author upon request. Source data are available via Zenodo at 10.5281/zenodo.15632199 (ref. ^41^). Source data are provided with this paper.
