# Supplementary material for: DNA moiré superlattices
Source: Nat Nanotechnol. 2025 Jul 17;20(10):1464–72. doi: 10.1038/s41565-025-01976-3 (PMC12534185; doi:10.1038/s41565-025-01976-3)
Supplement: Supplementary file 1 — Supplementary Figs. 1–72 and Tables 1–6. [file 41565_2025_1976_MOESM1_ESM.pdf]

nature nanotechnology

Supplementary information https://doi.org/10.1038/s41565-025-01976-3

DNA moiré superlattices

In the format provided by the authors and unedited

Contents

Supplementary Figures 1-72

Supplementary Tables 1-6

Additional References

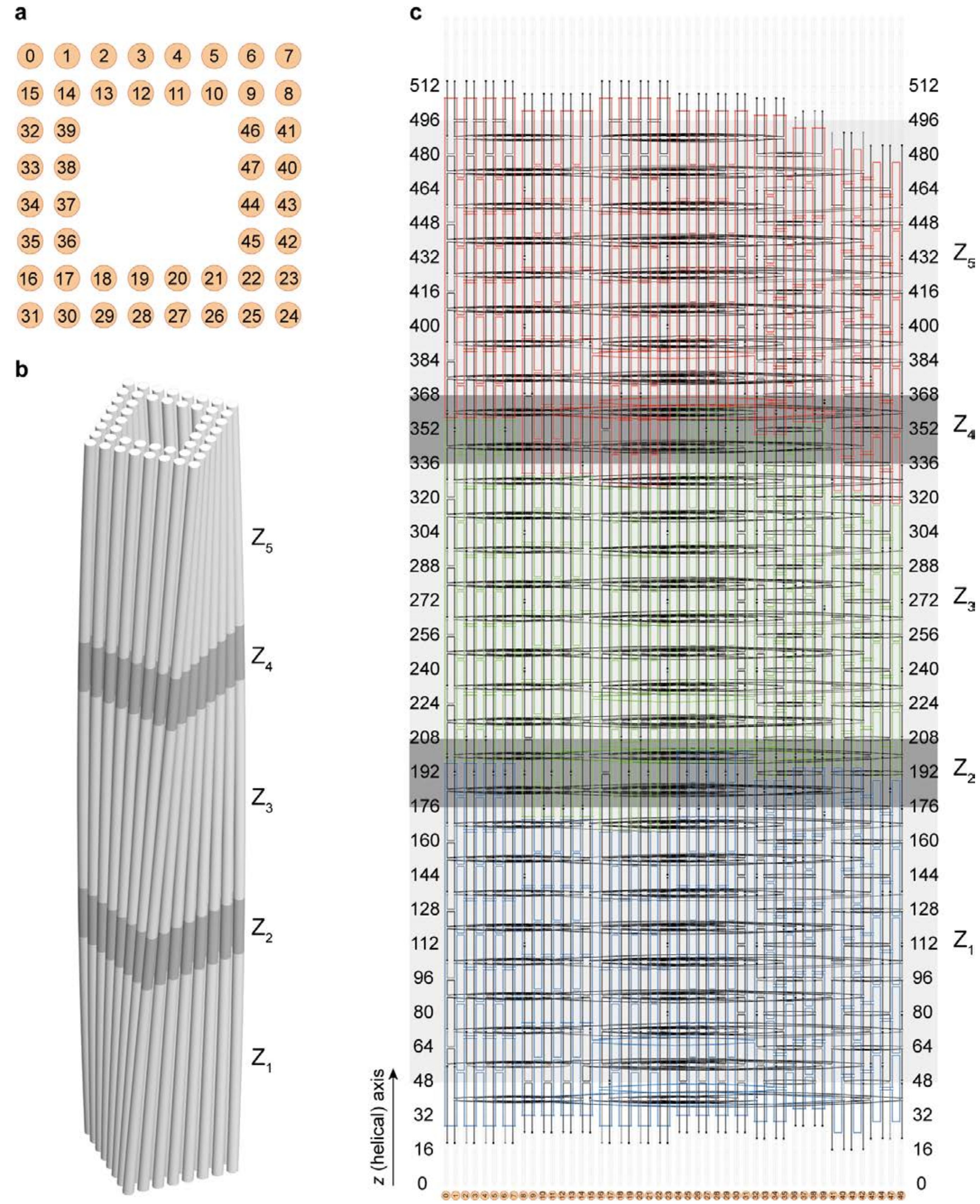

Supplementary Fig. 1 | Strand diagrams of seed-S. a, Cross-section view in caDNAno format. **b,** 3D view of the model. **c,** Detailed diagram of all strands in caDNAno format. The numbers at the bottom indicate the helices, while the numbers on the left and right indicate the positions of the bases along the *z* (helical) axis. Blue, green and red strands represent three sequence-orthogonal scaffolds: p7560, CS3-L and CS4, respectively. To aid visualization, the twist of the seed is intentionally amplified in the schematic.

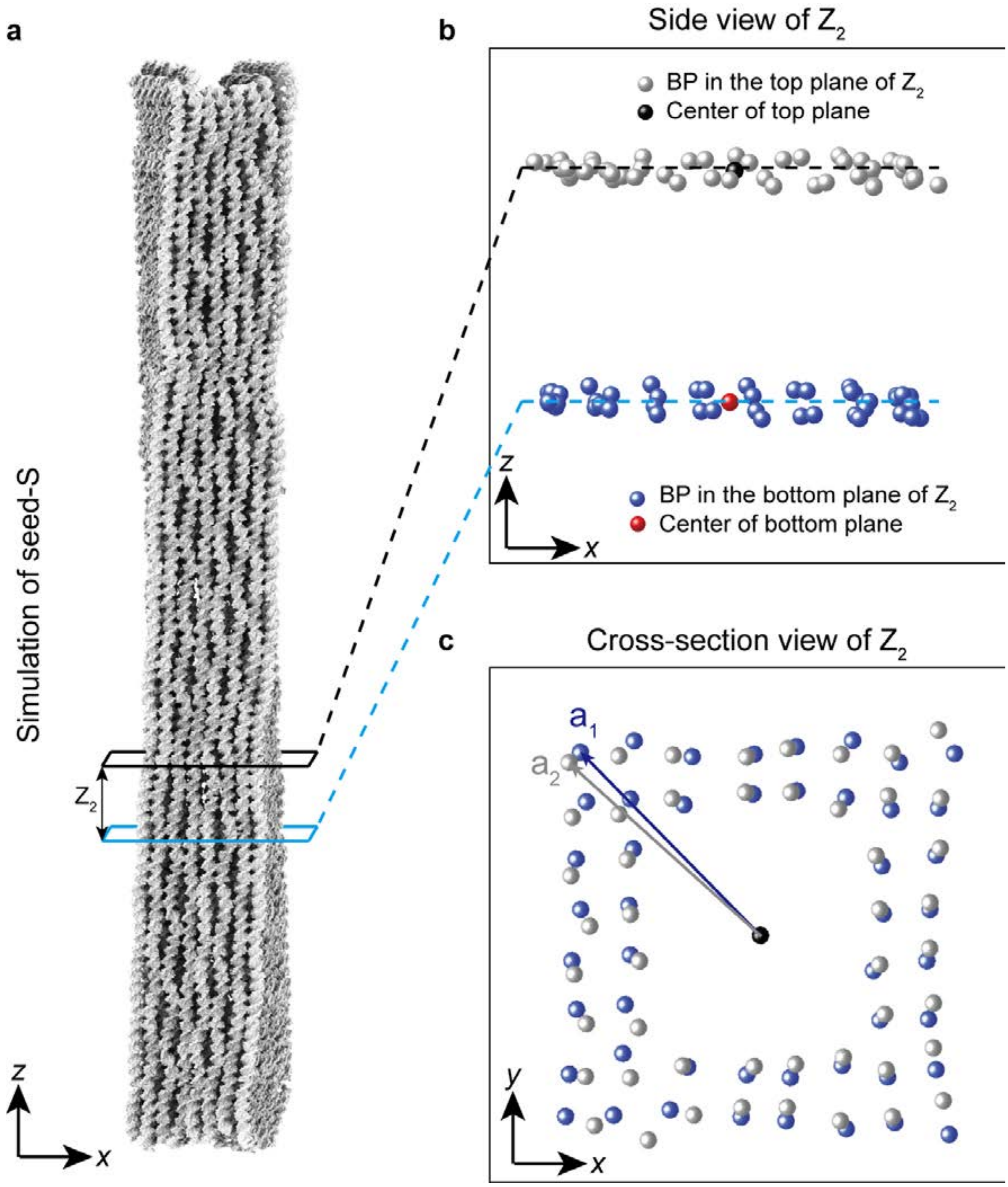

Supplementary Fig. 2 | Calculation of the twist angle of seed-S. a, Simulated 3D view of seed-S using the SNUPI program. The bottom and top planes of the Z_2 segment are marked. **b,** Bottom and top planes of the Z_2 segment. Each sphere (blue or grey) represents a base pair (BP). The center point (red or black sphere) was obtained by averaging the coordinates of all BPs in each plane. **c,** Cross-section view of the bottom (blue) and top (grey) planes of Z_2. For each helix, a BP in the bottom plane has a position vector, a_1 (x_1, y_1, z_1), while its corresponding BP in the top plane has a position vector a_2 (x_2, y_2, z_2). The twist angle for each helix was calculated based on the equation,

$$\cos\theta = \frac{a_1 \cdot a_2}{|a_1||a_2|} = \frac{x_1x_2 + y_1y_2 + z_1z_2}{\sqrt{x_1^2 + y_1^2 + z_1^2}\sqrt{x_2^2 + y_2^2 + z_2^2}}$$

The overall twist angle of Z_2 was obtained by averaging θ for all 48 helices of seed-S [1]. For seed-S without any insertion or deletion of bases, the average twist angle for Z_2 (32 bp) is θ ~3.8°.

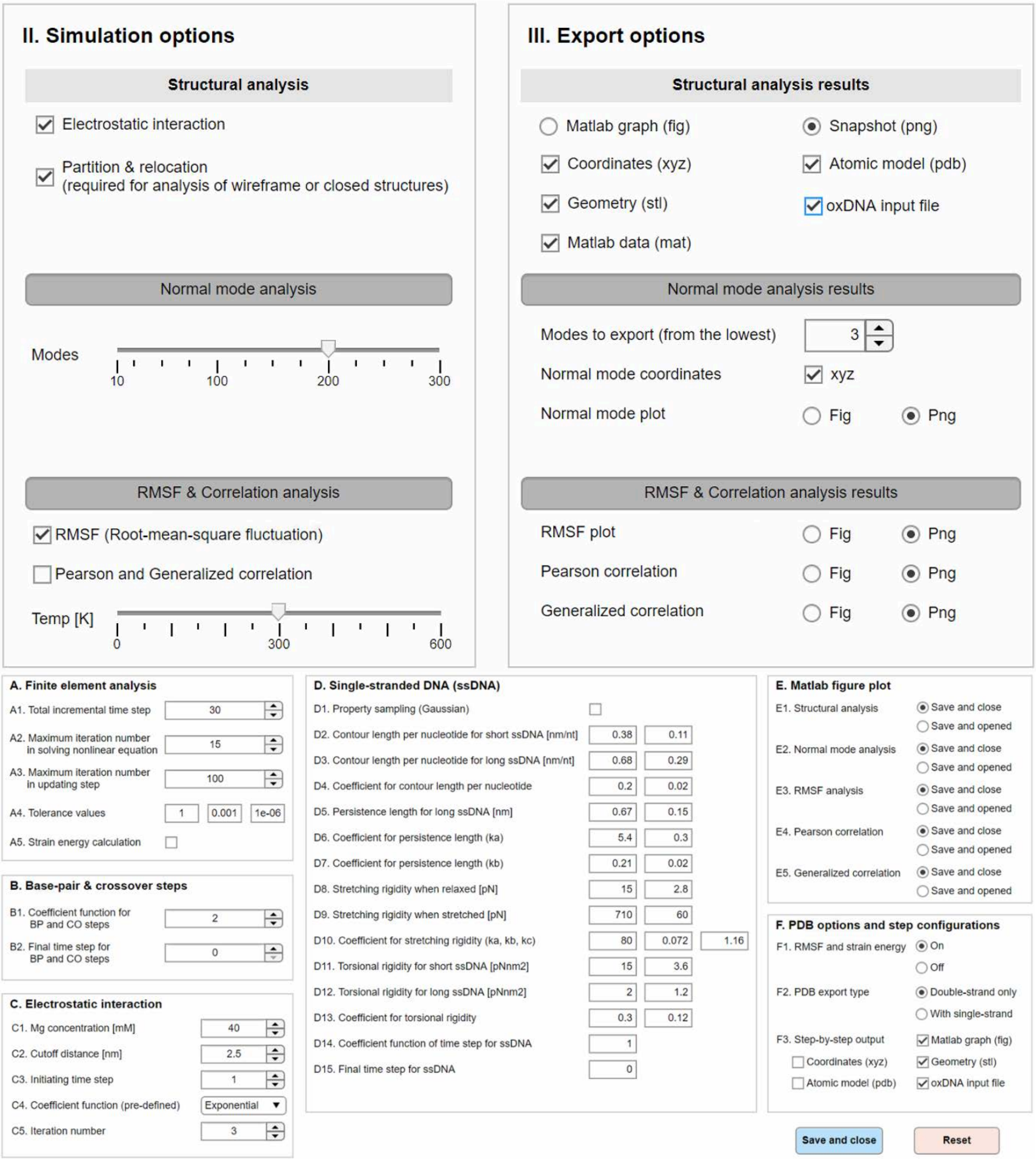

Supplementary Fig. 3 | Parameters used in coarse-grained simulations (SNUPI).

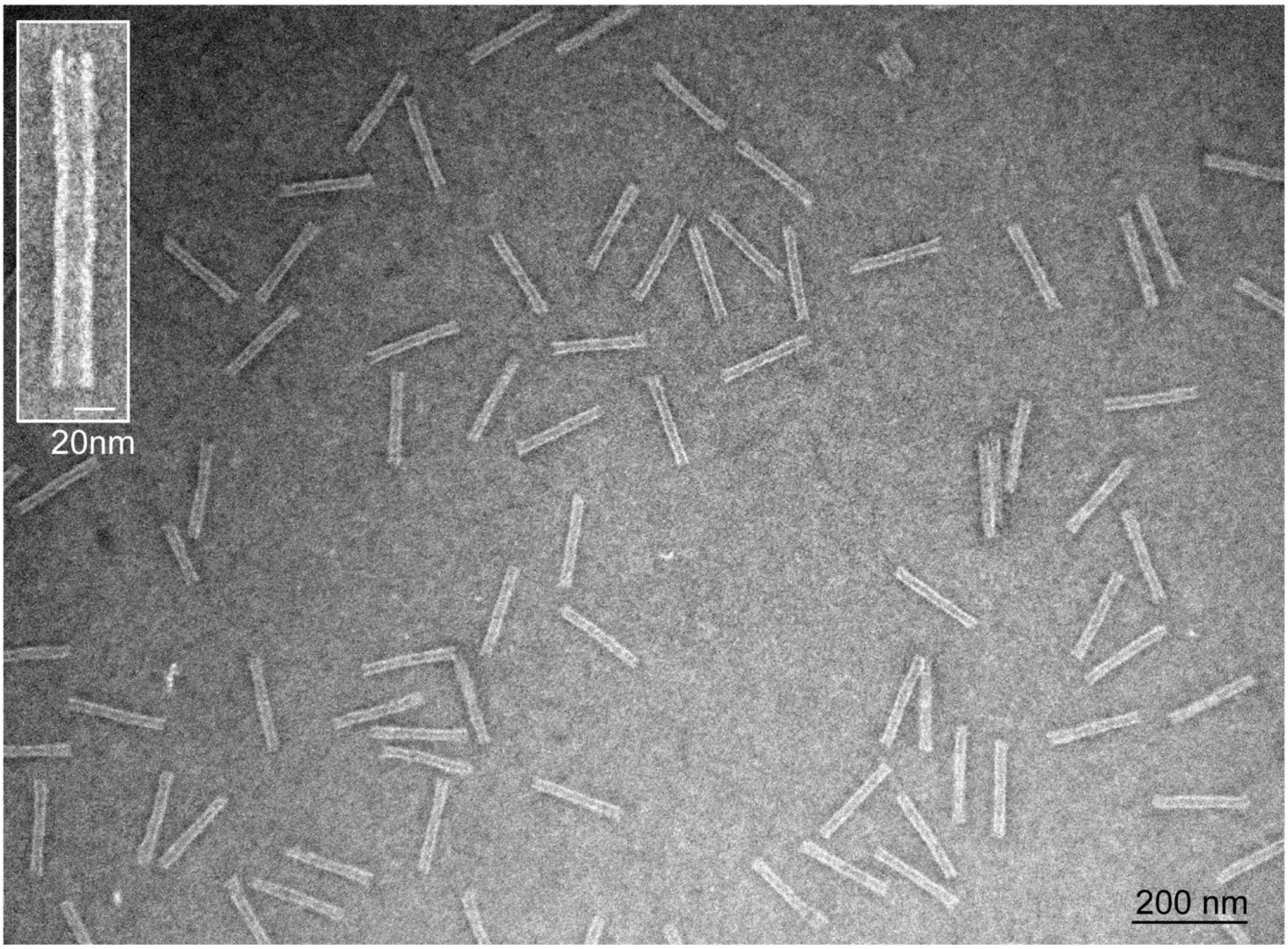

Supplementary Fig. 4 | TEM image of seed-S. Inset: high-magnification image.

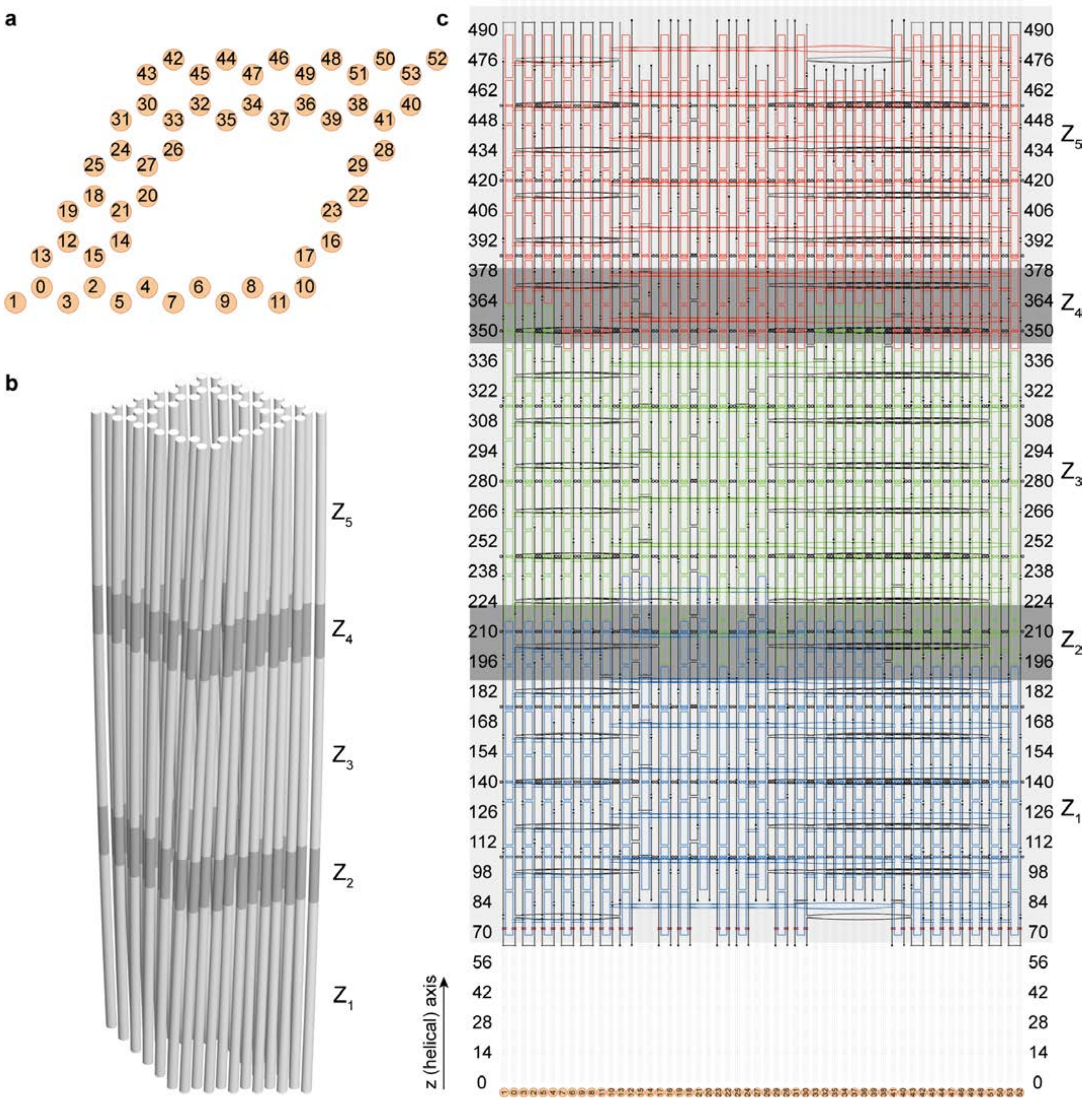

Supplementary Fig. 5 | Strand diagrams of seed-H. a, Cross-section view in caDNAno format. **b,** 3D view of the model. **c,** Detailed diagram of all strands in caDNAno format. The numbers at the bottom indicate the helices, while the numbers on the left and right indicate the positions of the bases along the *z* (helical) axis. Blue, green and red strands represent three orthogonal scaffolds: p7560, CS3-L and CS4, respectively. The Z_2 and Z_4 segments have one base insertion for each helix to generate a RH twist. Meanwhile, the Z_1, Z_3 and Z_5 segments have three base insertions for each helix to match the domain design of the honeycomb SST sublattice. To aid visualization, the twist of the seed is intentionally amplified in the schematic.

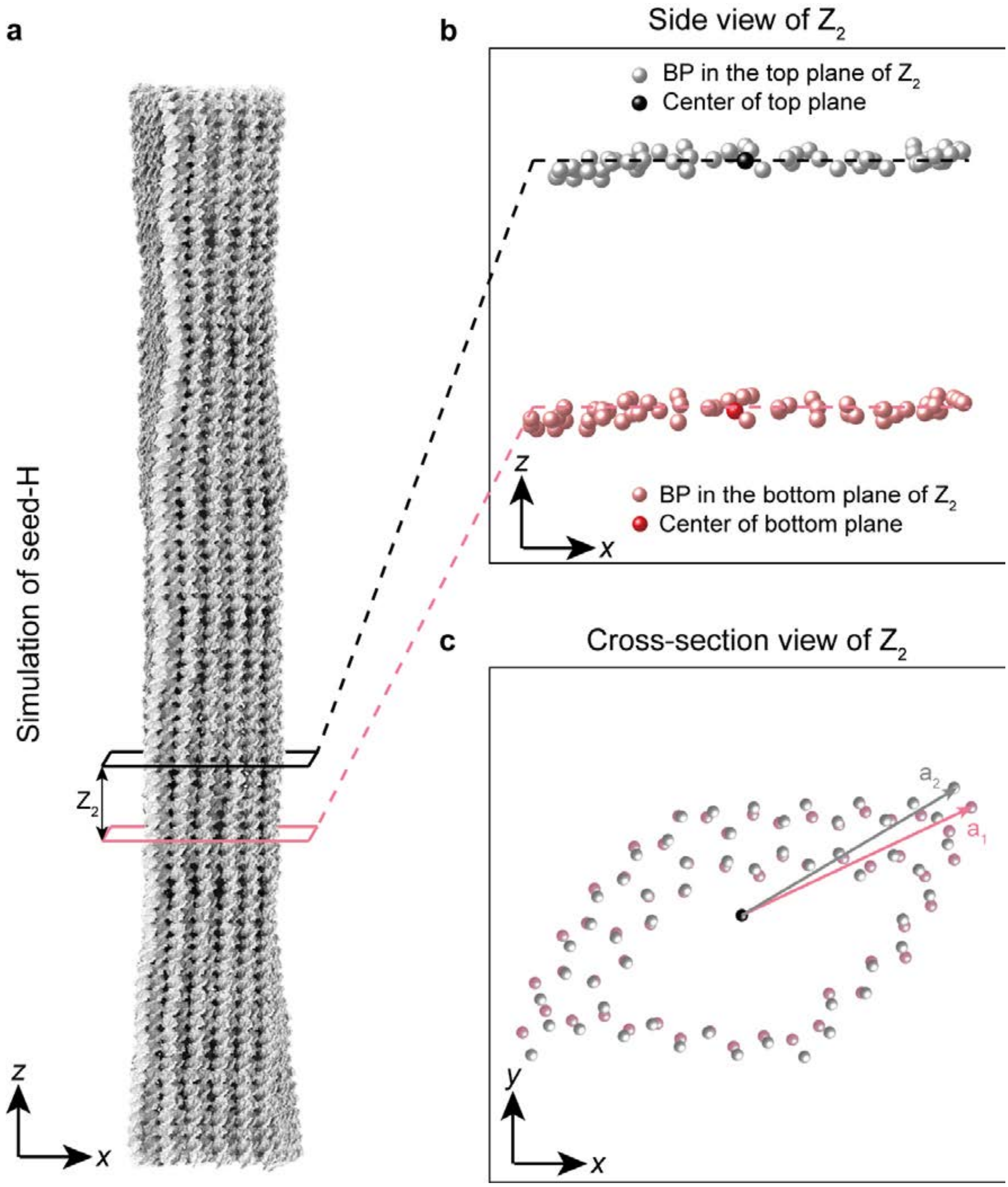

Supplementary Fig. 6 | Calculation of the twist angle of seed-H. a, Simulated 3D view of seed-H using the SNUPI program. The bottom and top planes of the Z_2 segment are marked. **b,** Bottom and top planes of the Z_2 segment. Each sphere (pink or grey) represents a BP. The center point (red or black sphere) was obtained by averaging the coordinates of all BPs in each plane. **c,** Cross-section view of the bottom (pink) and top (grey) planes of Z_2. The overall twist angle of Z_2 was obtained by averaging θ for all 54 helices of seed-H. For Z_2 (36 bp) with one base insertion for each helix, the average twist angle is $\theta \sim 1.2°$.

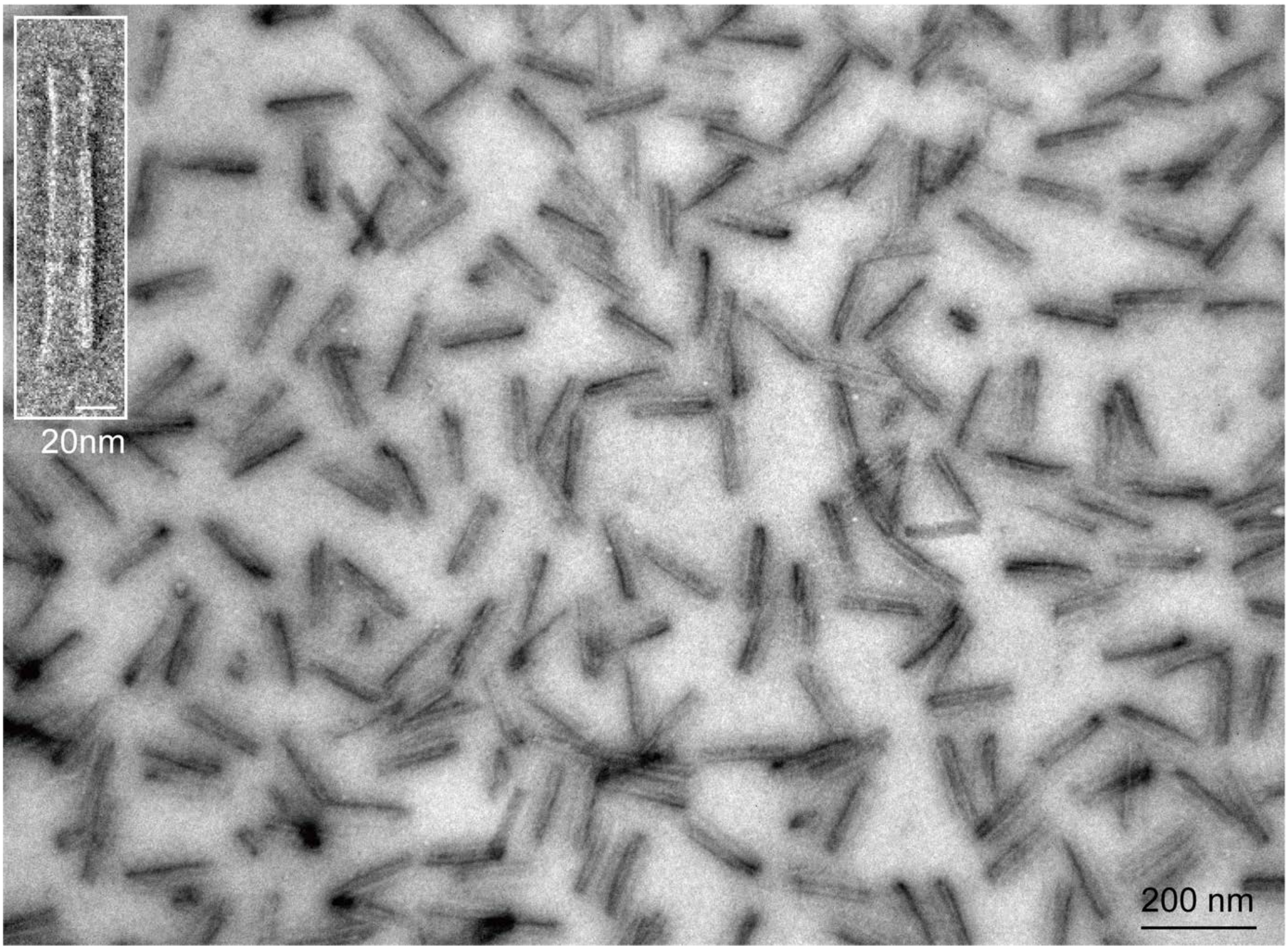

Supplementary Fig. 7 | TEM image of seed-H. Inset: high-magnification image.

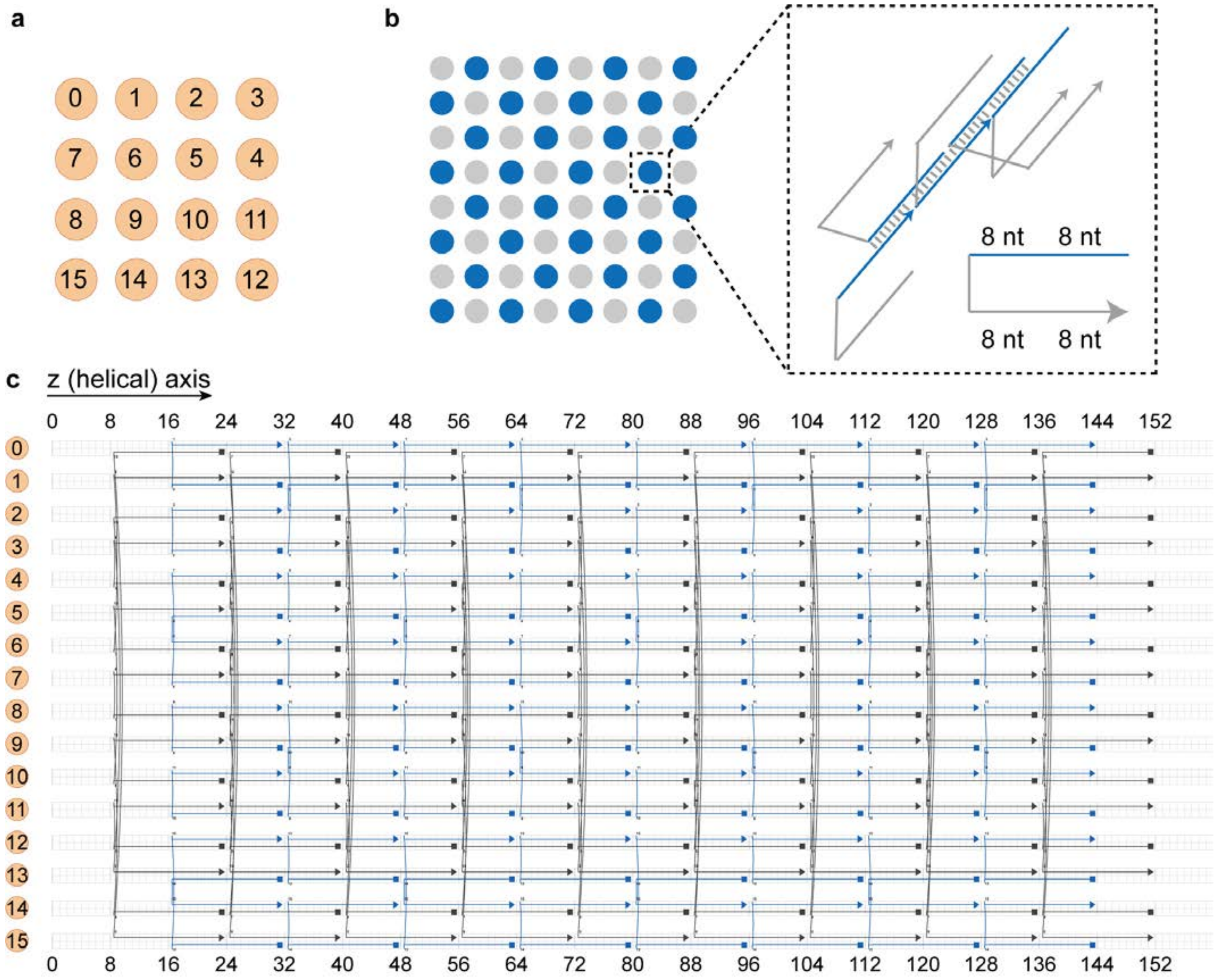

Supplementary Fig. 8 | Strand diagrams of the square SST sublattice. **a,** Cross-section view in caDNAno format. **b,** Domain design for each SST strand. **c,** Detailed diagram of all strands in caDNAno format. The numbers on the left indicate the helices, while the numbers on the top and bottom indicate the positions of the bases along the *z* (helical) axis.

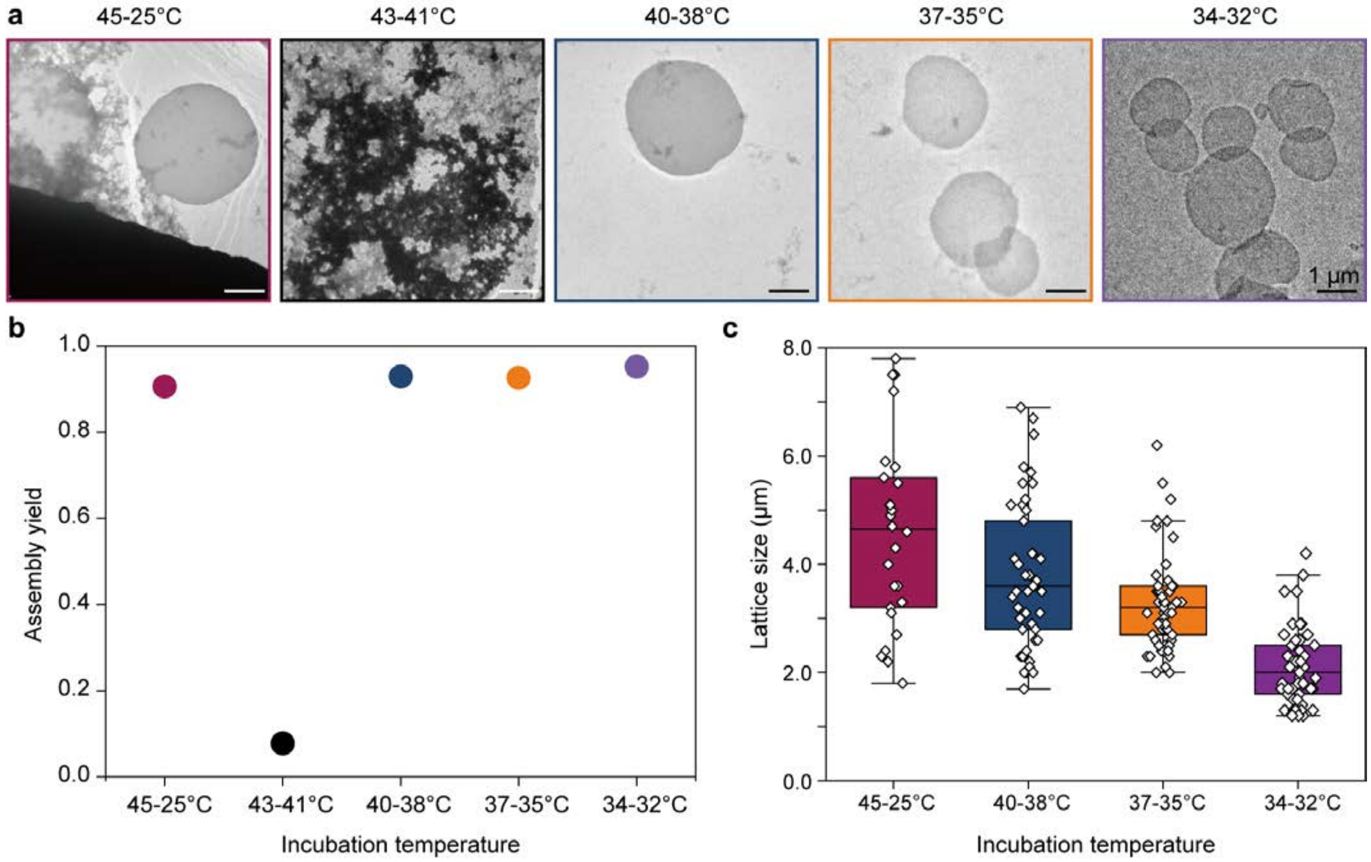

Supplementary Fig. 9 | Temperature influence on the square SST sublattice assembly. a, TEM images, **b,** assembly yield, and **c,** lattice size of the square SST sublattices assembled over 7 days within different temperature ranges. The SST assembly yield was estimated by subtracting the proportion of unassembled SSTs in the supernatant reaction buffer, because the assembled sublattices precipitated from the reaction buffer [2]. 40 °C-38 °C was approximately the starting assembly temperature for the square SST sublattices with an assembly yield > 90%. Lower temperature ranges (*e.g.*, 34 °C-32 °C) did not significantly improve the yield, but reduced the average lattice size. In **c**, from left to right, $N = 26, 45, 56, 51$, respectively. For the boxplots, the central line is the median, the minima and maxima of the box extends to the 25th and 75th percentiles, whiskers extend to data points within 1.5 × IQR of Q1 and Q3.

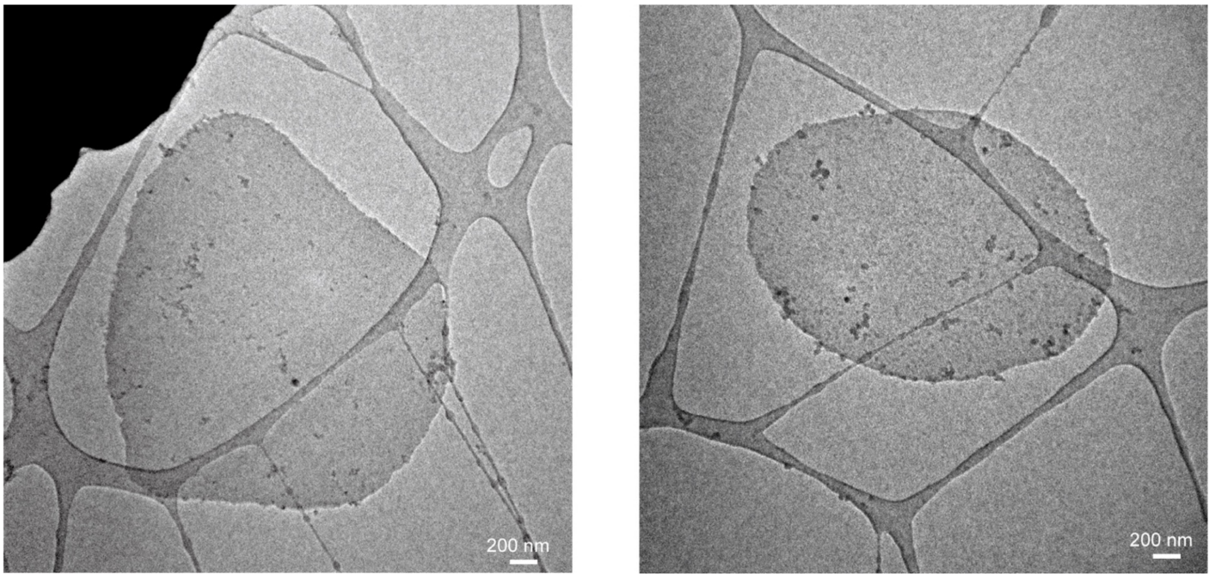

Supplementary Fig. 10 | Self-supporting SST sublattices on lacey carbon grids, demonstrating high structural rigidity.

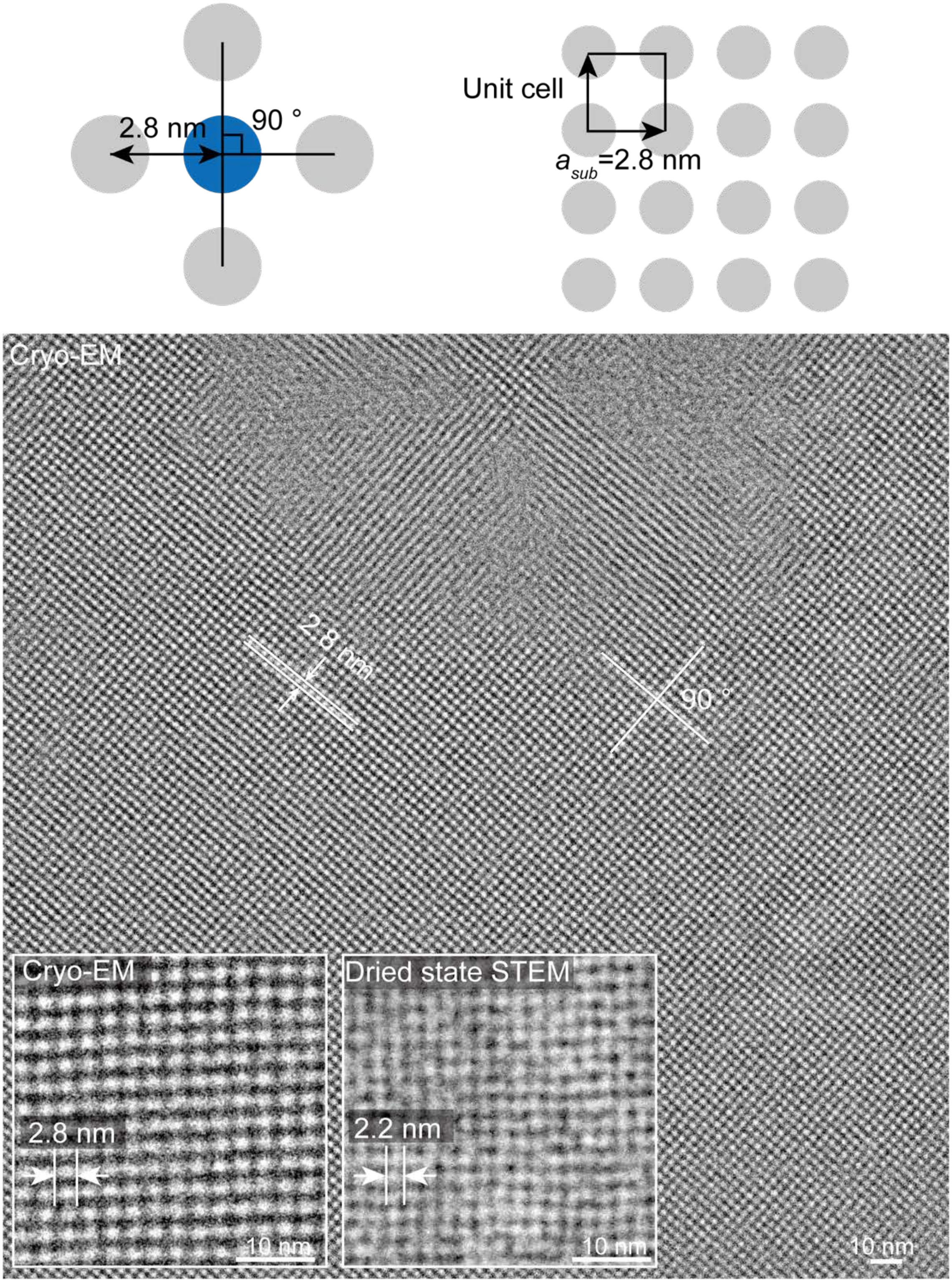

Supplementary Fig. 11 | High-magnification images of the square SST sublattice. The cryo-EM image reveals a C_4 symmetry with a_{sub} = 2.8 nm, corresponding to both the interplane and interhelix distances for square symmetry, and an interplanar angle of 90°. Inset left: high-resolution cryo-EM image, showing a lattice constant of 2.8 nm. Inset right: high-resolution STEM image of the dried state, showing a lattice constant reduction to 2.2 nm due to structural shrinkage.

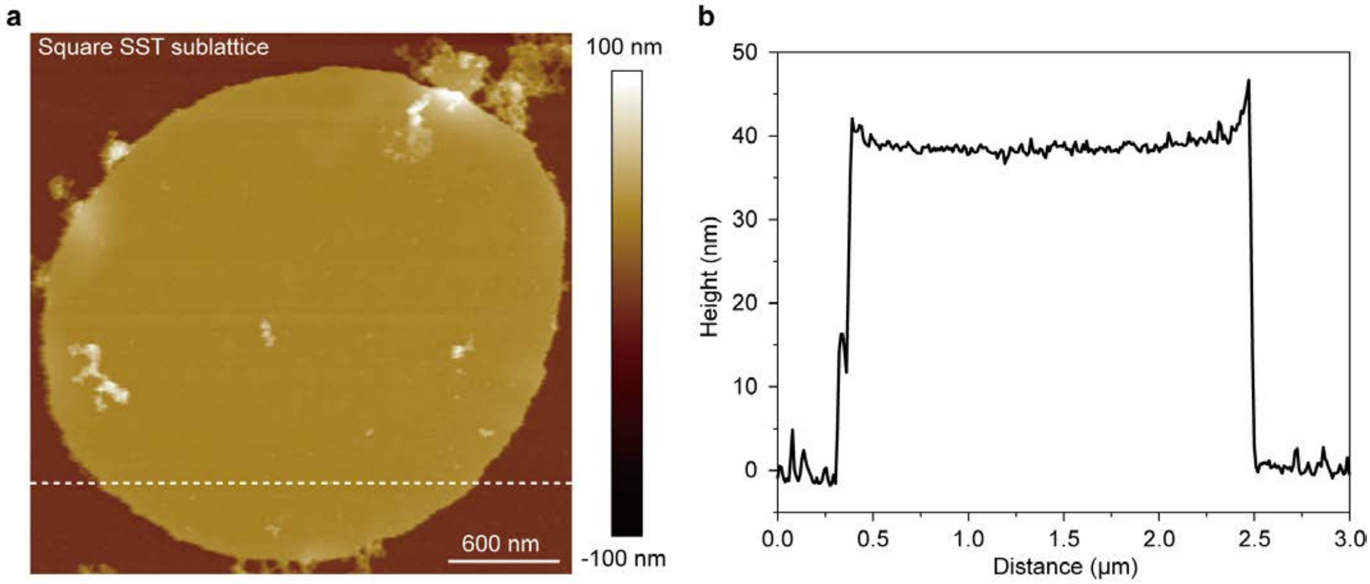

Supplementary Fig. 12 | a, AFM image, and b, height profile of the square SST sublattice.

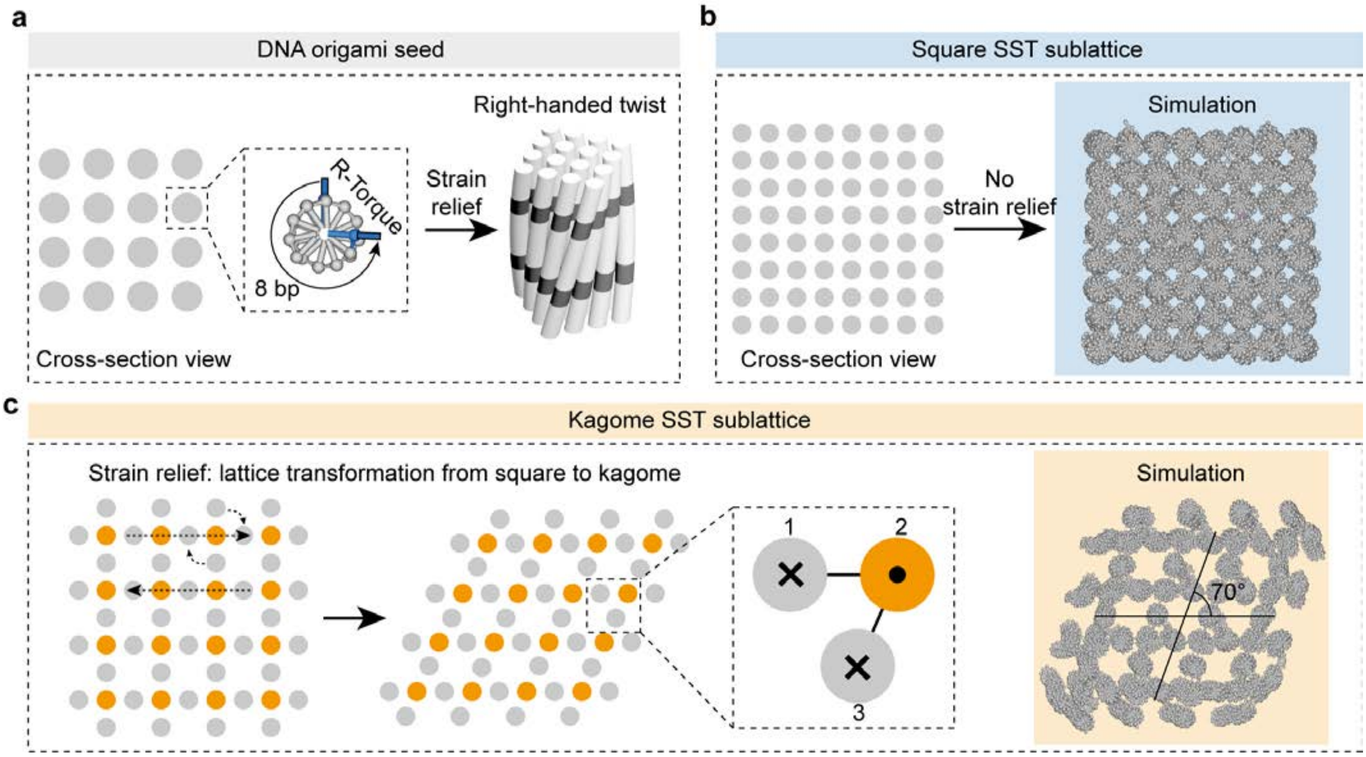

Supplementary Fig. 13 | Design of the kagome SST sublattice. a, In a DNA origami bundle with helices arranged in a square array and 8 bp domains, under-winding strain that exerts a right-handed torque can be alleviated by a compensatory global right-handed twist [3]. **b,** The microscale square SST sublattice does not relieve strain via a global twist due to its dense packing (exceeding 10^5 helices) in the *x*-*y* plane and a high torsional persistent length >10^7 nm [4]. Coarse-grained simulations of an 8-helix × 8-helix × 128 bp DNA structure show no twist. The high torsional rigidity of the SST sublattice also suggests that the nucleation and growth segments (Z_1, Z_3, or Z_5) of the twisted origami seed, with just 128 bp (43.5 nm in length) do not retain their twist after being incorporated into the SST sublattice. Only the Z_2 and Z_4 segments contribute to the interlayer twist. **c,** A loosely packed square SST sublattice relieves strain through a lattice transformation from square to kagome symmetry. In simulation, our design shows an interplanar angle of approximately 70°, differing from 60° of conventional kagome symmetry. Electrostatic repulsion between helix-1 and helix-3, which lack direct connectivity, contributes to this angle difference.

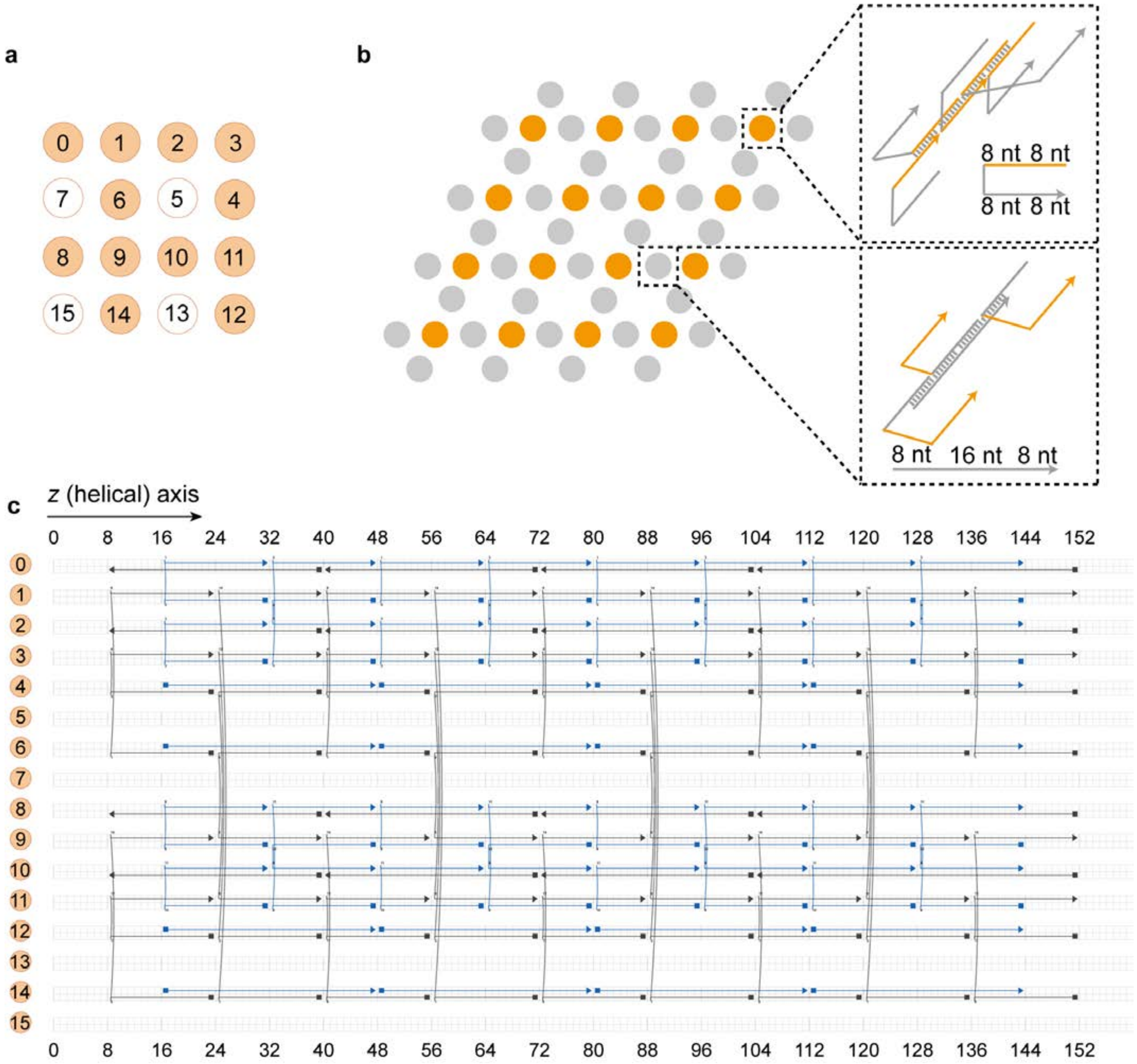

Supplementary Fig. 14 | Strand diagrams of the kagome SST sublattice. a, Cross-section view in caDNAno format. **b,** Domain design for each SST strand. In addition to the 32-nt SST used in the square sublattice, a linear-shaped SST with three domains (8 nt, 16 nt, 8 nt) is also used here in the kagome sublattice. **c,** Detailed diagram of all strands in caDNAno format. The numbers on the left indicate the helices, while the numbers on the top and bottom indicate the positions of the bases along the *z* (helical) axis.

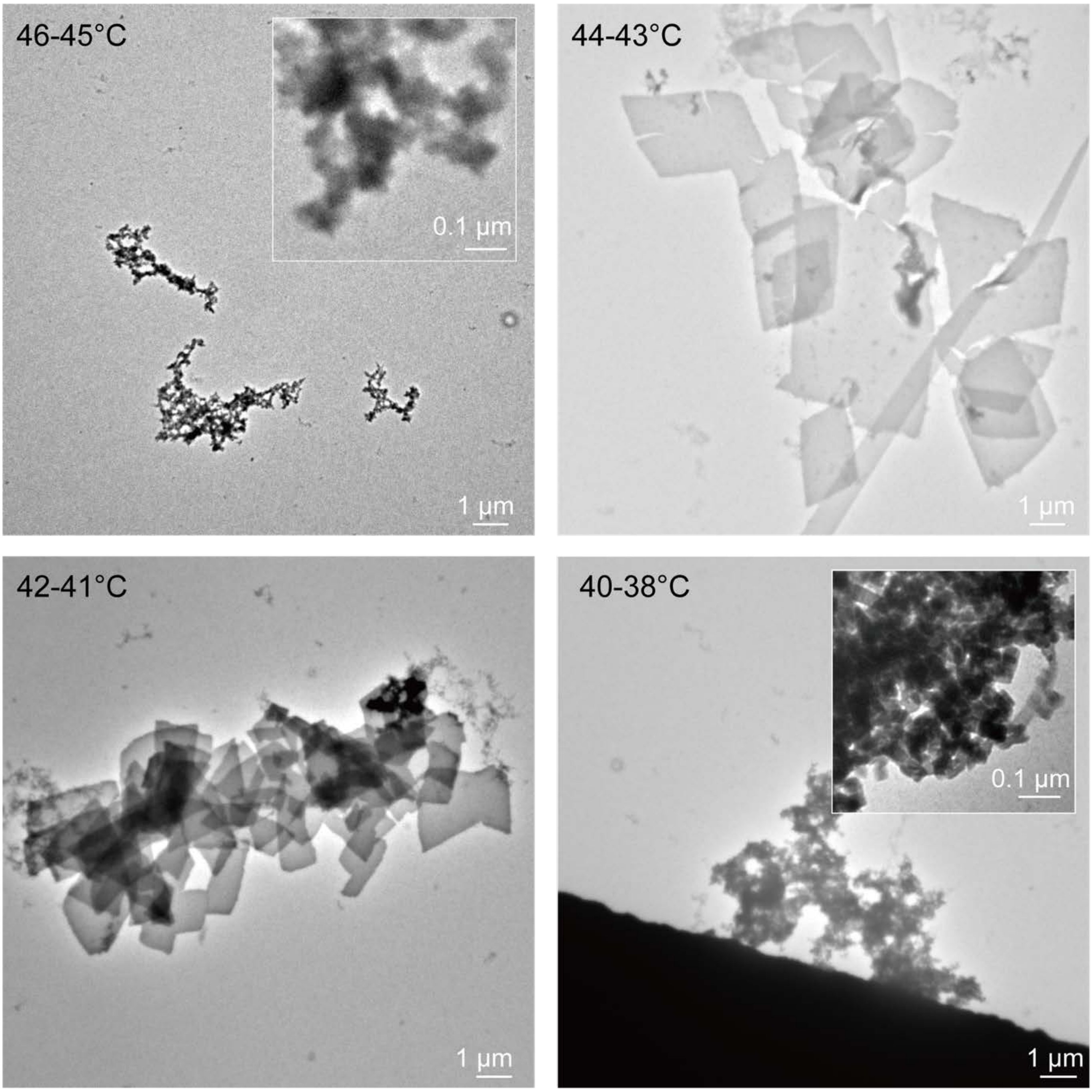

Supplementary Fig. 15 | Temperature influence on the kagome SST sublattice assembly. TEM images of the kagome SST sublattices assembled over 24 hours within different temperature ranges. The results suggest an optimal assembly temperature range of 44°C-43°C.

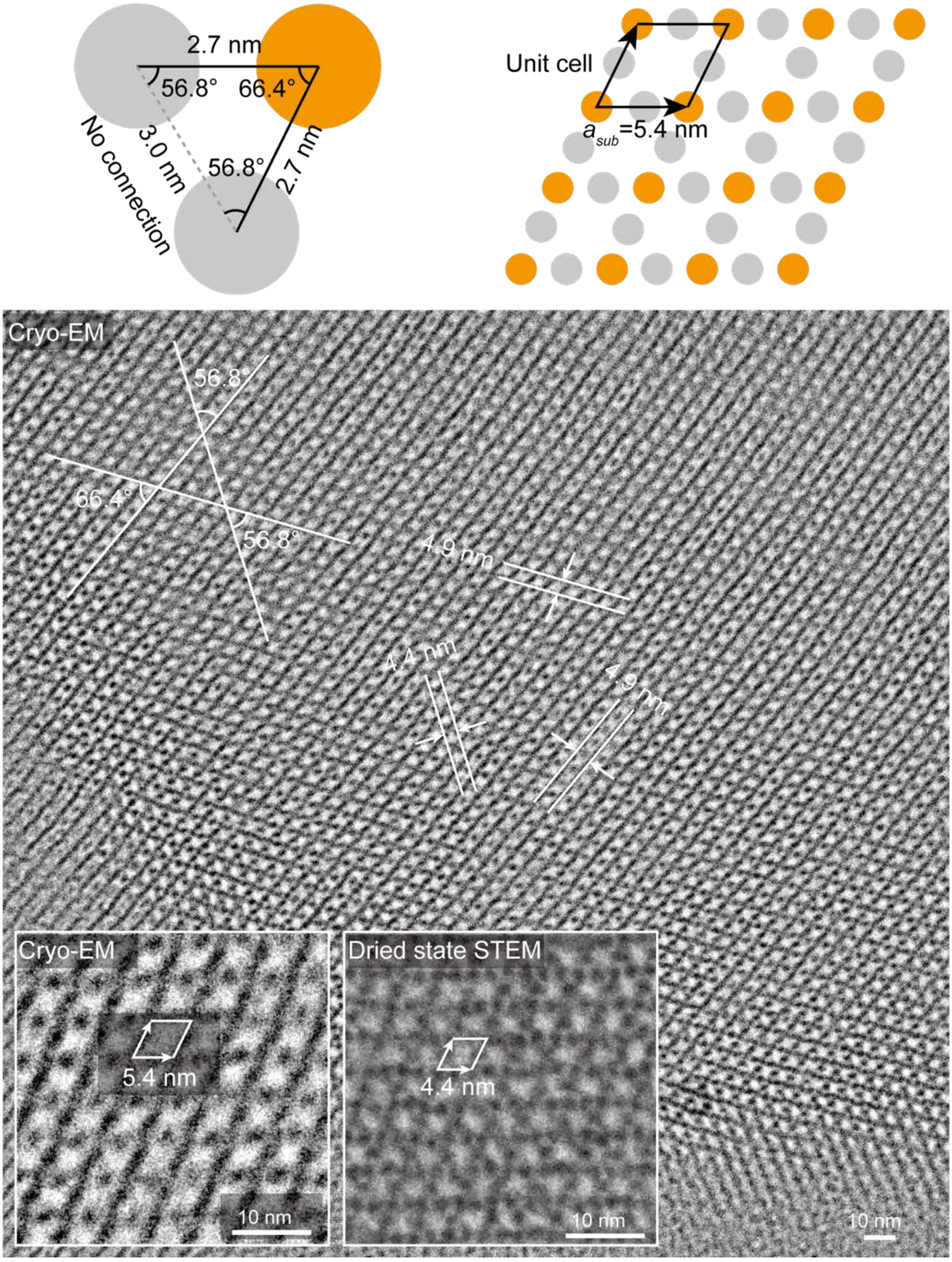

Supplementary Fig. 16 | High-magnification image of the kagome SST sublattice. The cryo-EM image reveals a near-C_6 symmetry with distinct interhelix distances and angles. The interhelix distances are 2.7 nm for connected helices and 3.0 nm for non-connected helices. The interplane distances are 4.9 nm, 4.9 nm, and 4.4 nm. a_{sub} is 5.4 nm in the cryo-state and 4.4 nm in the dried state, as shown in the insets.

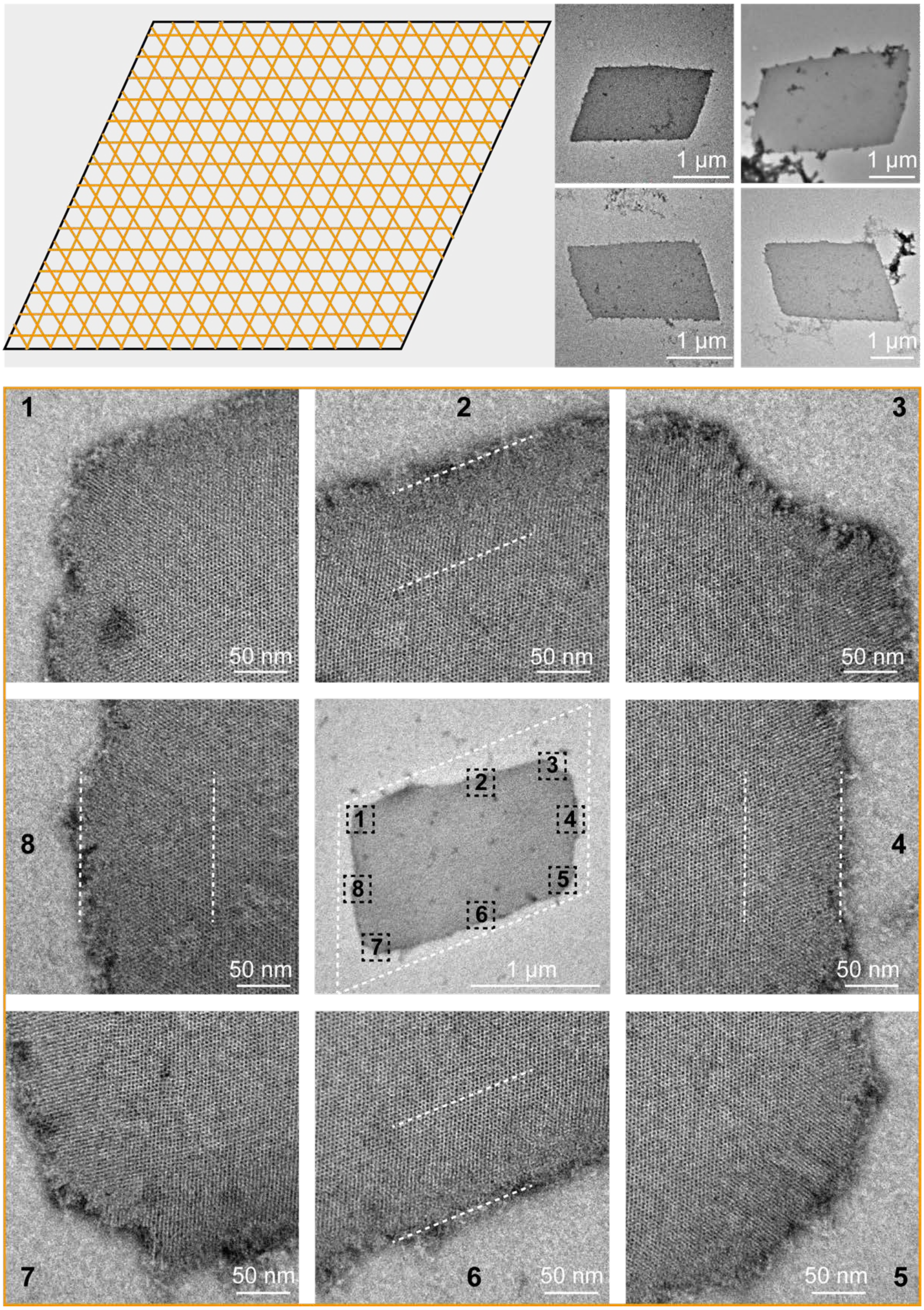

Supplementary Fig. 17 | Symmetry-conformed lattice shape for the kagome SST sublattice. Low- and high-magnification TEM images show that the four edges align well with the orientations of the corresponding lattice planes.

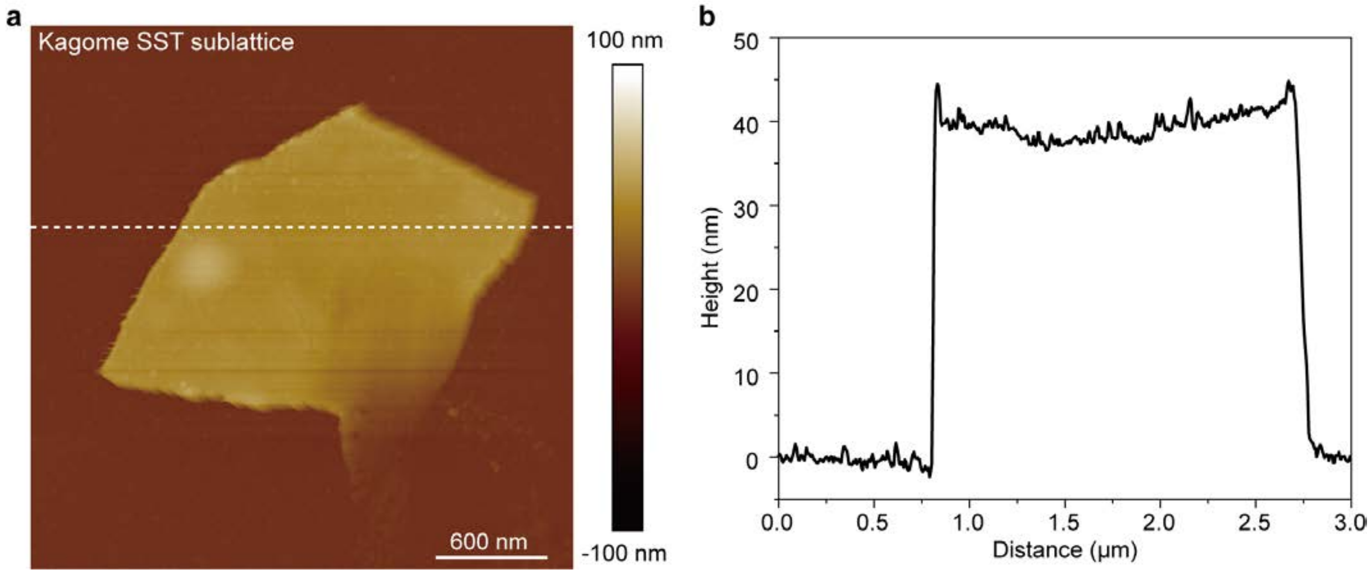

Supplementary Fig. 18 | a, AFM image and b, height profile of the kagome SST sublattice.

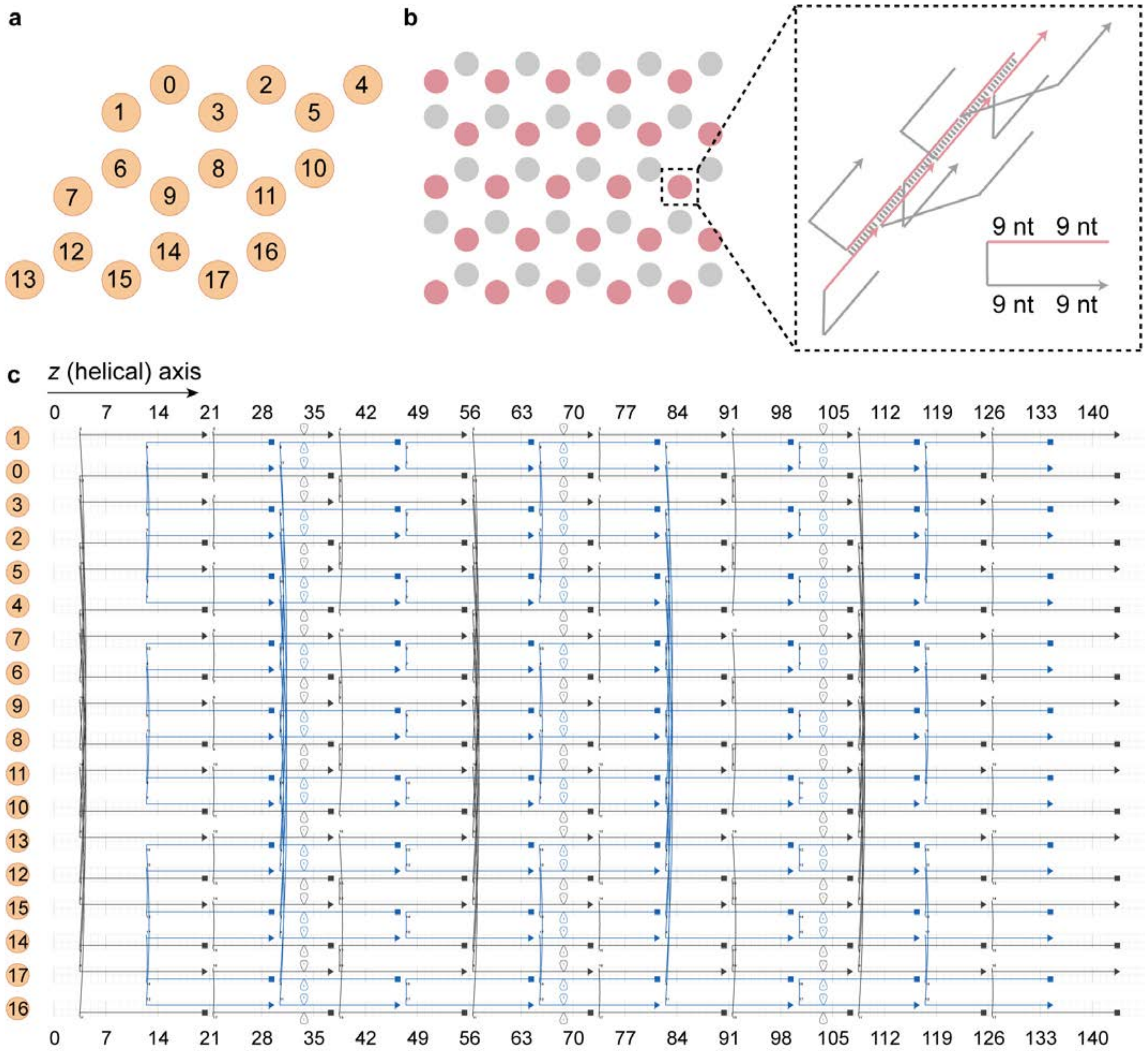

Supplementary Fig. 19 | Strand diagrams of the honeycomb SST sublattice. a, Cross-section view in caDNAno format. **b,** Domain design for each SST strand. **c,** Detailed diagram of all strands in caDNAno format. The numbers on the left indicate the helices, while the numbers on the top and bottom indicate the positions of the bases along the *z* (helical) axis.

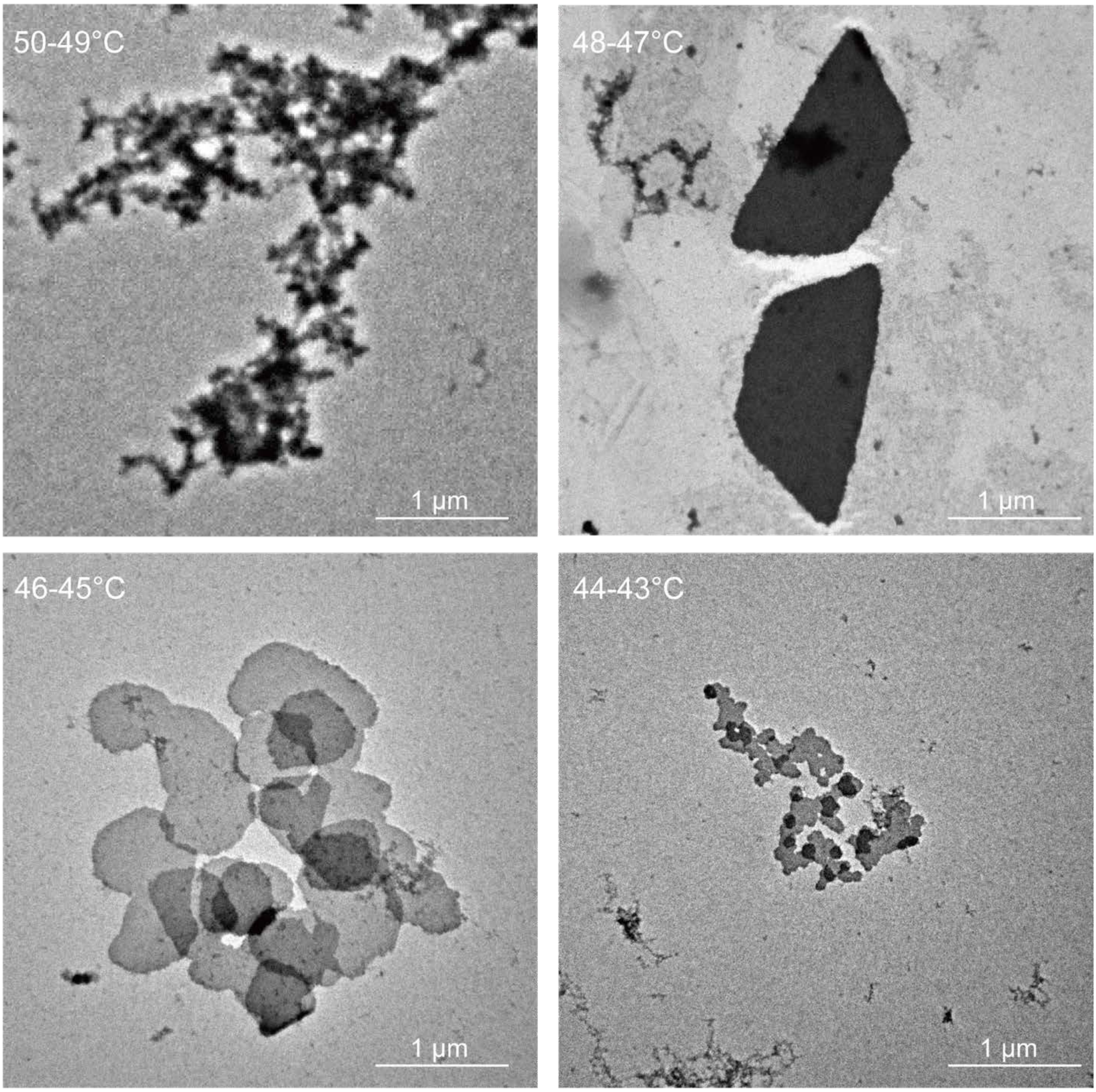

Supplementary Fig. 20 | Temperature influence on the honeycomb SST sublattice assembly. TEM images of the honeycomb SST sublattices assembled over 24 hours within different temperature ranges. The results suggest an optimal assembly temperature range of 48°C-47°C.

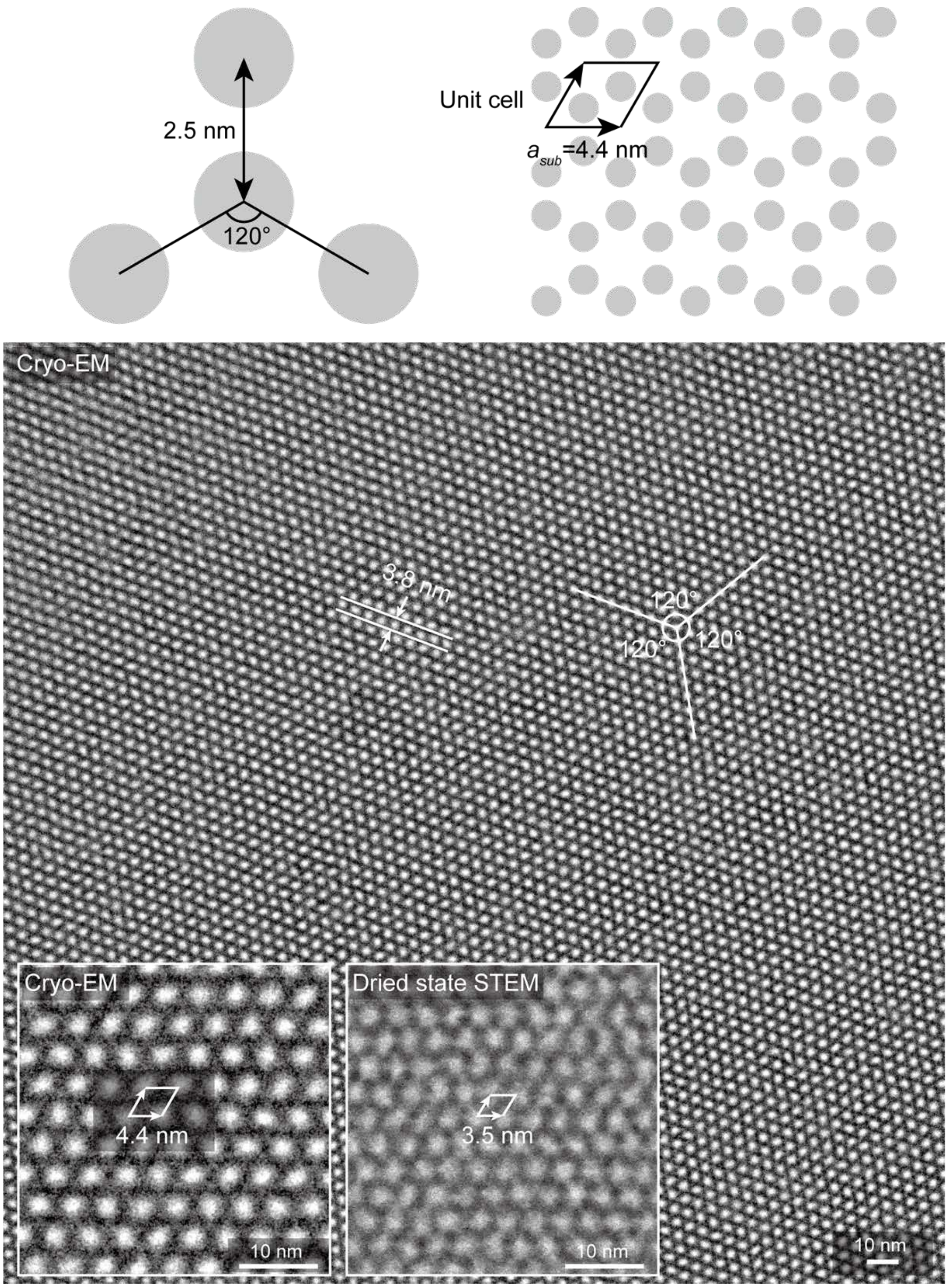

Supplementary Fig. 21 | High-magnification image of the honeycomb SST sublattice. The cryo-EM image reveals a C_6 symmetry with a_{sub}= 4.4 nm, an interplanar distance of 3.8 nm, and an interplanar angle of 120°. The interhelix distance is 2.5 nm, indicating denser packing than in the square and kagome SST sublattices. In the dried state, a_{sub} is reduced to 3.5 nm due to structural shrinkage.

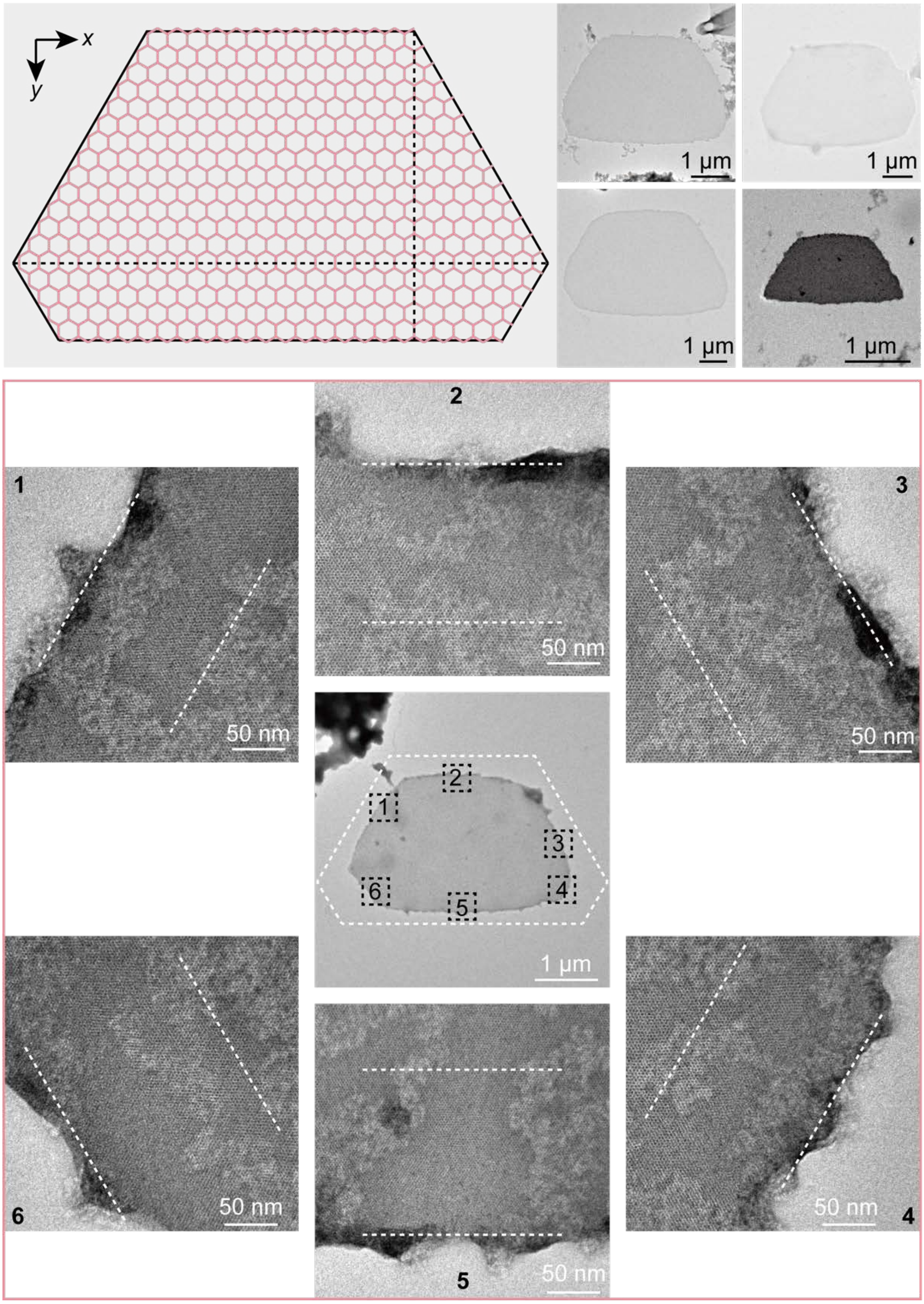

Supplementary Fig. 22 | Symmetry-conformed lattice shape for the honeycomb SST sublattice. Low-magnification TEM images reveal an asymmetric hexagonal shape. High-magnification TEM images show that the six edges align with the orientations of the corresponding lattice planes.

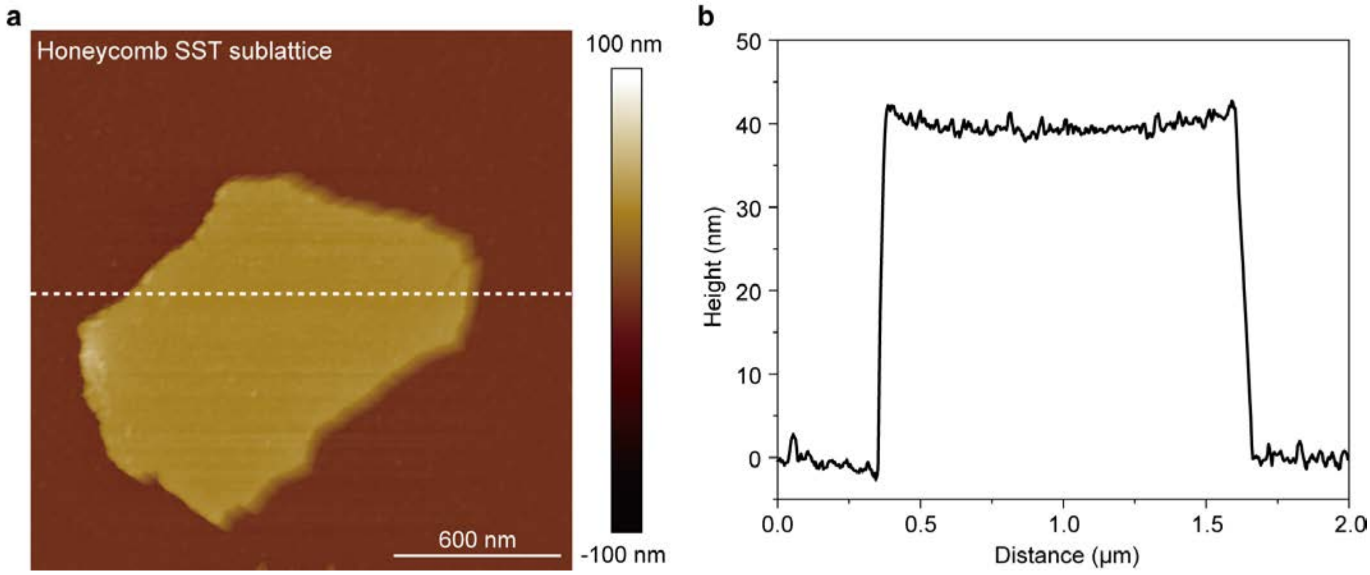

Supplementary Fig. 23 | a, AFM image and b, height profile of the honeycomb SST sublattice.

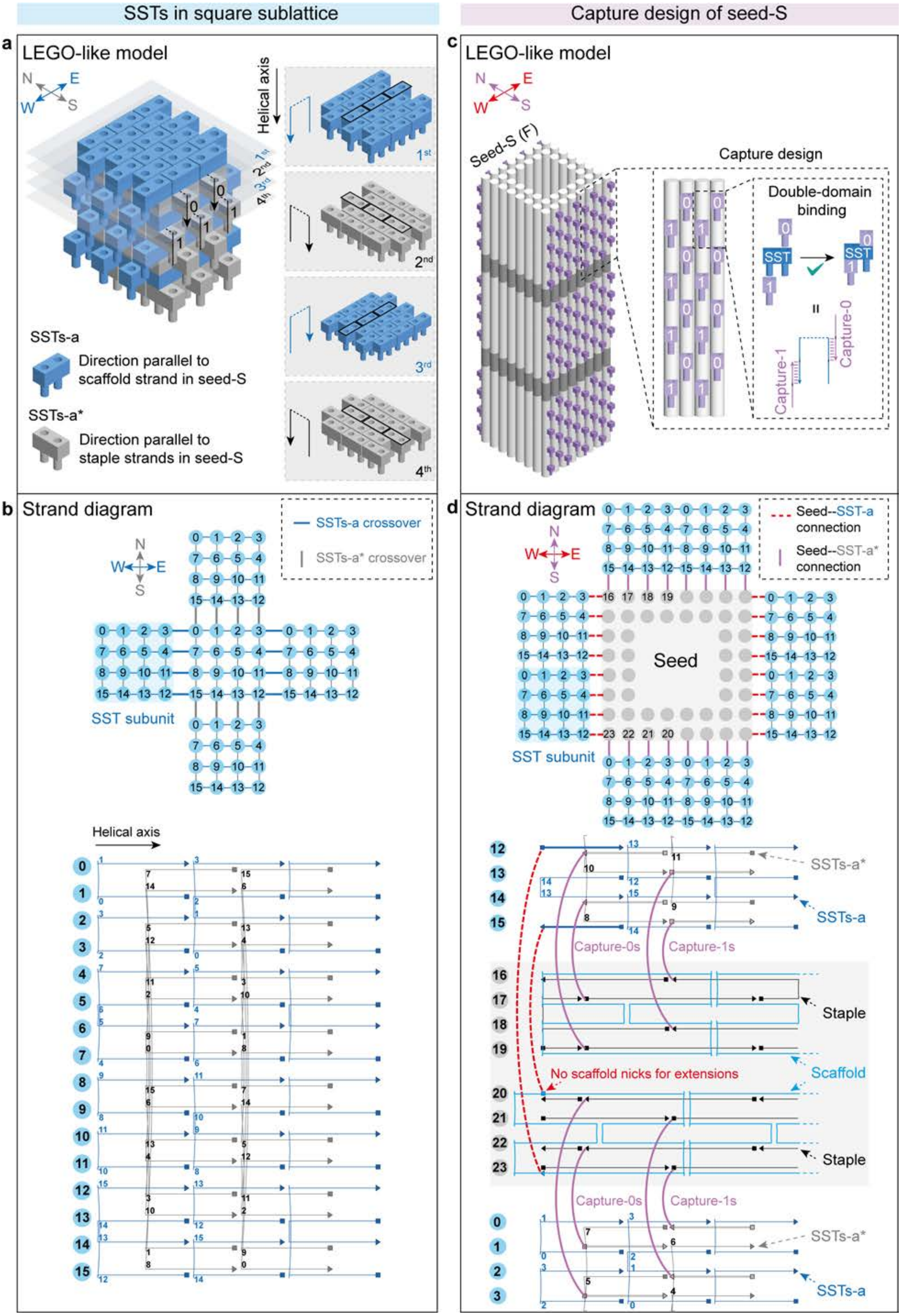

Supplementary Fig. 24 | Capture design of seed-S for seeded growth of square SST superlattices. a, LEGO-like model, and **b,** strand diagram of the square SST sublattice. Two complementary sets, SSTs-a (blue) and SSTs-a* (grey), interact via single-domain binding [5] (also refer to **Fig. 2A**). They are parallel to scaffold and staple strands in seed-S, respectively. In a subunit of the square SST sublattice, SSTs-a and SSTs-a* rotate along the helical axis in C_4 symmetry, alternating layer by layer, and exposing unbound domains of SSTs-a

and SSTs-a* on orthogonal surfaces (a-domains: west/east; a*-domains: north/south). Meanwhile, the unbound domains on each surface are reversed in direction (*e.g.*, a*-0 in the 2nd layer and a*-1 in the 4th layer on surface-south), forming a double-domain binding pair to capture neighboring SSTs. SSTs-a on surfaces-west/east follow the same growth scheme. **c,** LEGO-like model, and **d,** strand diagram illustrate the capture design of seed-S. Scaffold routing restricts capture extensions from the scaffold positions without nicks. Thus, only surfaces-north/south can extend captures, equivalent to SSTs-a*, which help capture SSTs-a onto the origami seed. Helices 16-19 and 20-23 serve as representative growth surfaces of seed-S, illustrating capture extension. Captures from helices 16-19 of the seed bind SSTs-a from helices 12-15, while captures from helices 20-23 of the seed bind helices 0-3. To enable this, SSTs-a* crossovers between surface-north and surface-south of the SST subunit are severed, with resulting half SSTs-a* connecting to corresponding positions on the seed. Accordingly, the captures on the seed retain the same crossovers, domains and sequences as the SST sublattice. Nucleation and growth occur only on surface-north and surface-south of the seed. Over time, SSTs grow and subsequently fuse into a 2D SST sublattice.

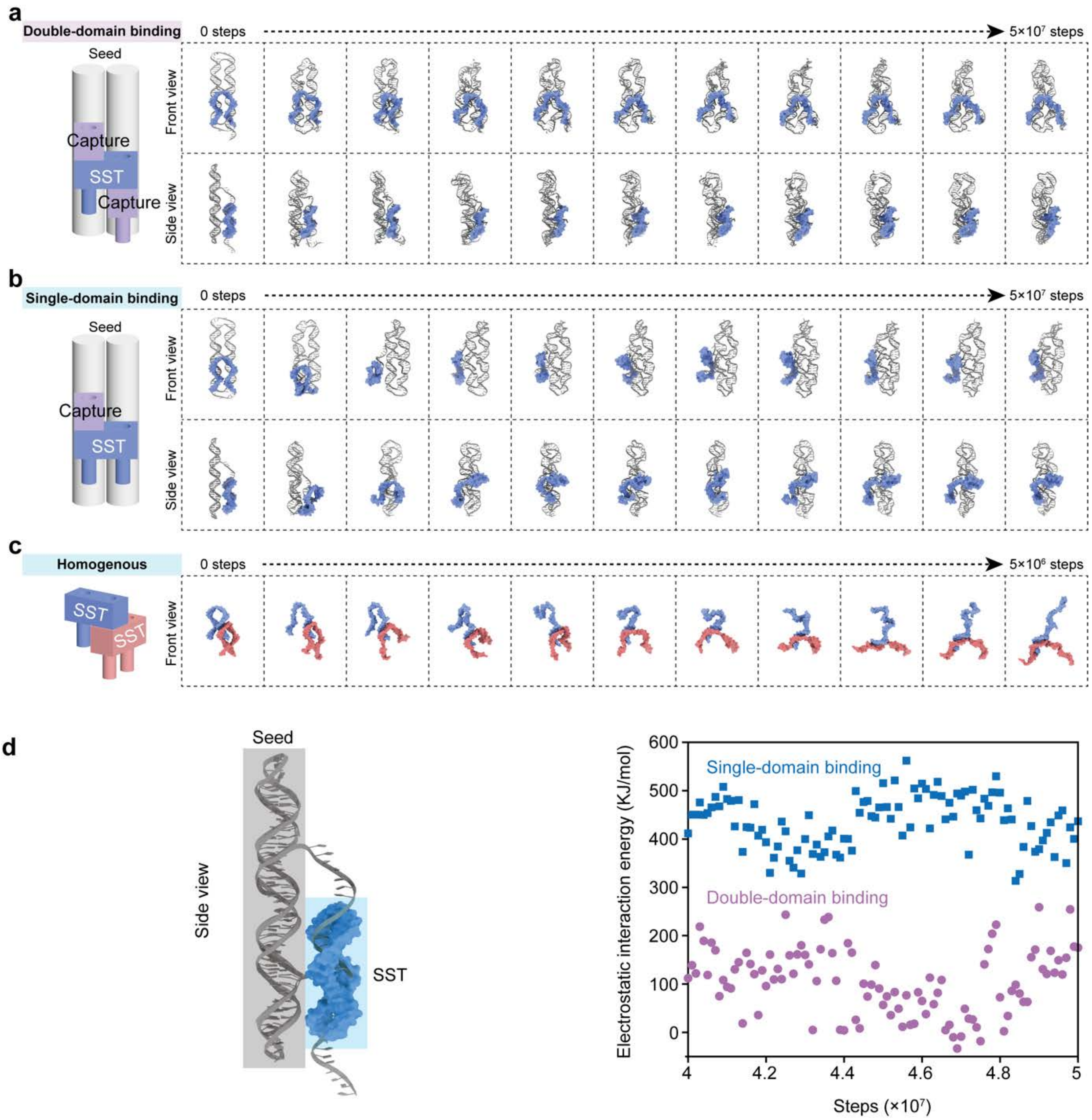

Supplementary Fig. 25 | All-atom molecular dynamics simulations elucidating the influence of electrostatic repulsion on seed design across different nucleation modes. Snapshots were taken at intervals of 5×10^6 steps for double-domain binding (**a**) and single-domain binding (**b**) in heterogeneous nucleation, and at 5×10^5 steps for homogeneous nucleation (**c**). **d**, Electrostatic interaction energies between the seed and SST for double-domain binding (purple) and single-domain binding (blue) in heterogeneous nucleation.

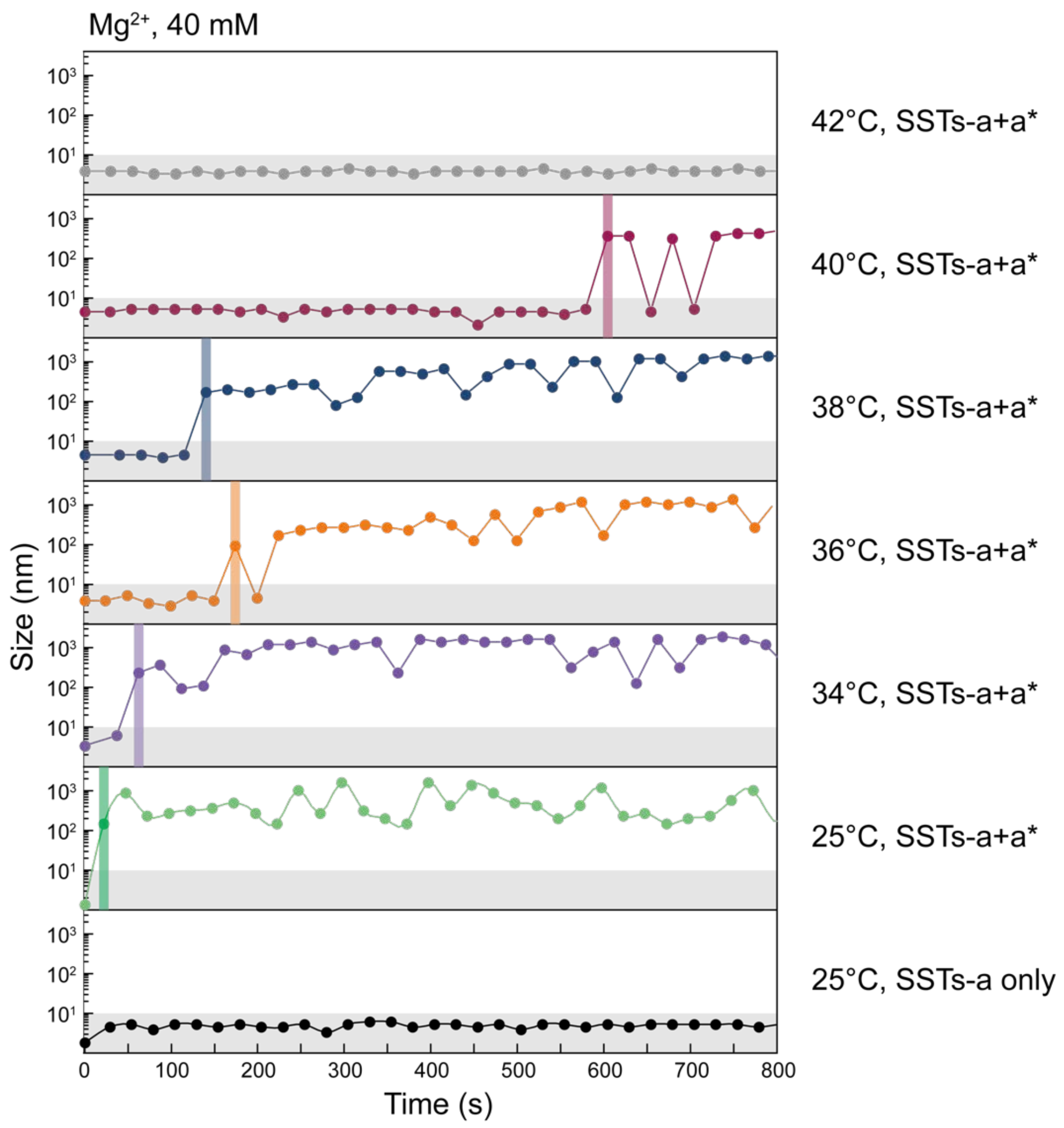

Supplementary Fig. 26 | Size characterization of homogenous (unseeded) nucleation for square SST sublattices. DLS was used to measure the sizes of square SST sublattices at different temperatures during incubation with 40 mM Mg^{2+}. The results suggest temperature-dependent nucleation kinetics. Time points for the first size burst are marked by vertical-colored lines in the figure, representing nucleation initiation in different cases. At 25 °C, SSTs-a only exhibit stable sizes below 10 nm (highlighted in grey), indicating no nucleation due to the absence of complementary SSTs-a*. Upon addition of SSTs-a*, nucleation proceeds gradually at 25 °C, with size reaching close to 1000 nm. As temperature increases, nucleation decelerates. The nucleation rate at 40°C is roughly 10 times slower than that at 34°C. At 42°C, nucleation is undetectable within the measured time frame (800 s), consistent with our observation that no sublattices form above 40°C (**Supplementary Fig. 9**).

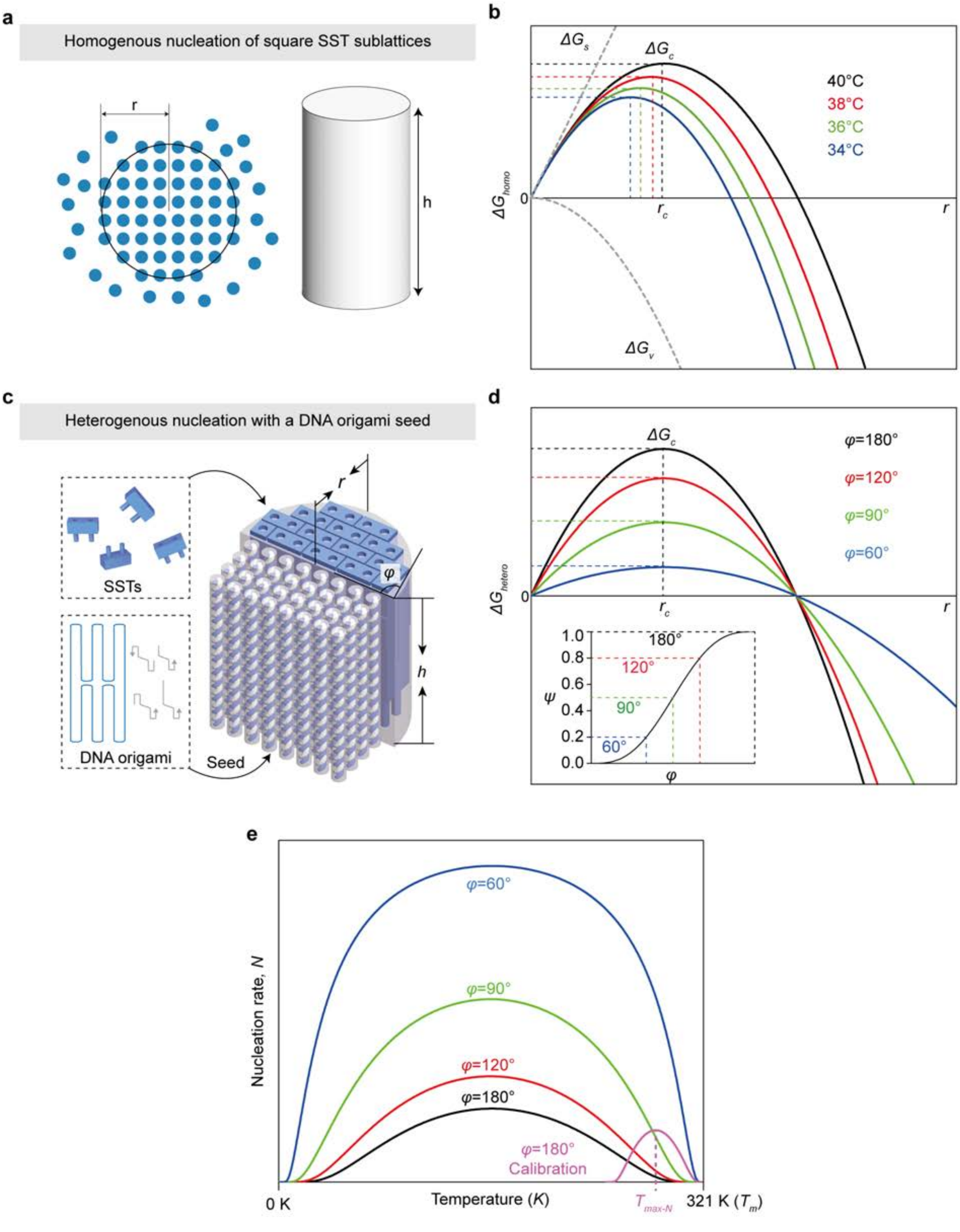

Supplementary Fig. 27 | Thermodynamic analysis of homogenous (unseeded) and heterogenous (seeded) nucleation based on classical nucleation theory (CNT) [6]. a, 2D cylinder model, and **b,** free energy (ΔG_{homo}) as a function of nucleus radius (r) for homogenous nucleation of square SST sublattices. In the 3D sphere model of CNT, the system's free energy is the sum of volume energy ΔG_v and surface energy ΔG_s, expressed as follows:

$$\Delta G_{homo}^{3D} = \Delta G_v + \Delta G_s = \Delta g_v \cdot \rho \cdot \frac{4}{3}\pi \cdot r^3 + \gamma \cdot 4\pi r^2 = \frac{q_m}{T_m}\Delta T \cdot \rho \cdot \frac{4}{3}\pi \cdot r^3 + 4\pi\gamma \cdot r^2 = -\frac{4\pi\rho q_m}{3T_m} \cdot |\Delta T| \cdot r^3 + 4\pi\gamma \cdot r^2 \quad (1)$$

where g_v is the volume Gibbs energy per unit mass, ρ is the nucleus density, γ is surface tension, q_m is the specific heat of solidification, T_m is the melting temperature of 32-nt SSTs (~ 321 K) [7], and ΔT is the supercooling temperature (ΔT=T-T_m). r and ΔT serve as two variables. For a 2D cylinder with constant height h, the free energy can be written as:

$$\Delta G_{homo}^{2D} = \Delta G_v + \Delta G_s = \Delta g_v \cdot \rho \cdot h \cdot \pi r^2 + \gamma \cdot h \cdot 2\pi r = -\frac{\pi h \rho q_m}{T_m} \cdot |\Delta T| \cdot r^2 + 2\pi h \gamma \cdot r \quad (2)$$

$$\frac{d(\Delta G)}{dr} = 0 \quad (3)$$

$$r_{c,homo} = \frac{\gamma T_m}{\rho q_m |\Delta T|} \quad (4)$$

$$\Delta G_{c,homo} = \frac{\pi h \gamma^2 T_m}{\rho q_m |\Delta T|} \quad (5)$$

The critical nucleation radius ($r_{c,homo}$) and critical free energy ($\Delta G_{c,homo}$, kinetic barrier) are calculated and marked in **b**. It reveals that lower temperatures yield smaller r_c and lower ΔG_c, facilitating faster nucleation, but also generating more nuclei and thus decreased lattice size (see **Supplementary Fig. 9**). **c,** Schematic and **d,** free energy (ΔG_{hetero}) as a function of r for heterogenous nucleation. Here, SSTs bind onto the origami seed's lateral surface of height h with contact angle φ as described by surface wetting in CNT. Including seed surface energy yields a modified equation:

$$\Delta G_{hetero} = \psi \cdot \Delta G_{homo},\ \psi = \frac{\varphi}{180^\circ} - \frac{\cos\varphi \sin\varphi}{\pi} \quad (6)$$

$$r_{c,hetero} = r_{c,homo} = \frac{\gamma T_m}{\rho q_m |\Delta T|} \quad (7)$$

$$\Delta G_{c,hetero} = \psi \cdot \Delta G_{c,homo} = \psi \cdot \frac{\pi h \gamma^2 T_m}{\rho q_m |\Delta T|} \quad (8)$$

ΔG_{hetero} as a function of r with different φ at a fixed temperature are plotted in **d**. Calculated $r_{c,hetero}$ and $\Delta G_{c,hetero}$ are highlighted on the curves, while the relation between ψ and φ is shown in the inset. Notably, $r_{c,hetero} = r_{c,homo}$, which means that designing larger seeds (> r_c) can more readily bypass the kinetic barrier ΔG_c, favouring seeded nucleation. Our experiments use an 8-helix × 8-helix DNA origami seed with a cross-section four times larger than the SST subunit. Additionally, $\Delta G_{c,hetero} = \Delta G_{c,homo}$ for $\varphi = 180°$, while decreasing φ (higher wetting) reduces $\Delta G_{c,hetero}$ as revealed by ψ in the inset, highlighting the importance of the capture design in determining seed-SST surface affinity. **e,** Nucleation rate (N) as a function of temperature for heterogeneous nucleation, calculated using the following equation [6]:

$$N \sim \exp\left(\frac{-\Delta G_c}{k_B \cdot T}\right) = a \cdot \exp\left(\frac{-\pi h \gamma^2 T_m \cdot \psi}{k_B \rho q_m \cdot T \cdot |T - T_m|}\right) = a \cdot \exp\left(b \cdot \frac{T_m \cdot \psi}{T \cdot |T - T_m|}\right) \quad (9)$$

where constants a and b simplify the variables to contact angle φ and temperature T. Smaller φ (higher wetting) enhances seeded nucleation, evidenced by increased nucleation rates. The nucleation rate also depends on the diffusion coefficient of SSTs (<1, not shown in equation (9)), shifting N curves to higher temperatures, *e.g.*, the purple curve. After this calibration, the resulting $T_{\max\text{-}N}$ becomes more realistic than the non-calibrated value (around 160.5 K). Based on our TEM and DLS results (**Supplementary Fig. 9 and 26**), $T_{\max\text{-}N}$ = 34°C (307 K). The nucleation rate ratio between two temperatures, T_1 and T_2, can be determined by

$$\frac{N_{T1}}{N_{T2}} = \exp\left(b \cdot T_m \cdot \psi \cdot \left(\frac{1}{T_1 \cdot |T_1 - T_m|} - \frac{1}{T_2 \cdot |T_2 - T_m|}\right)\right) \quad (10)$$

As shown in **Supplementary Fig. 26**, the homogenous nucleation rate (ψ = 1) at 40°C (313 K) is approximately 10 times lower than that at 34°C (307 K) with T_m = 48°C (321 K), allowing calculation of b. For these calculations, temperature-dependence of the diffusion coefficients is not considered, because for the temperatures of interest, for instance, T_1 = 313 K and T_2 = 307 K, the diffusion coefficient difference is negligible (< 2%) [6]. The nucleation rate ratio R (=N_{hetero}/N_{homo}) between heterogenous and homogenous nucleation can be calculated by

$$R = \frac{N_{hetero}}{N_{homo}} = \exp\left(b \cdot T_m \cdot \frac{\psi - 1}{T \cdot |T - T_m|}\right) \quad (11)$$

φ=120°and φ=60° are chosen to qualitatively represent low- and high-wetting, respectively (**Fig. 2c**).

The analogy between our system and classical nucleation theory arises from the fact that SST binding on the lateral surface of the origami seed mirrors liquid-surface wetting behavior. Specifically, the fully-cooperative

mode represents high wetting, where capture pairs facilitate layer-by-layer SST binding on the seed surface. The partially-cooperative mode corresponds to low wetting, with sparse capture pairs leading to initial patchy SST attachment. The non-cooperative mode and homogeneous nucleation resemble dewetting, where SST binding on the seed is ineffective or SSTs interact without surface guidance.

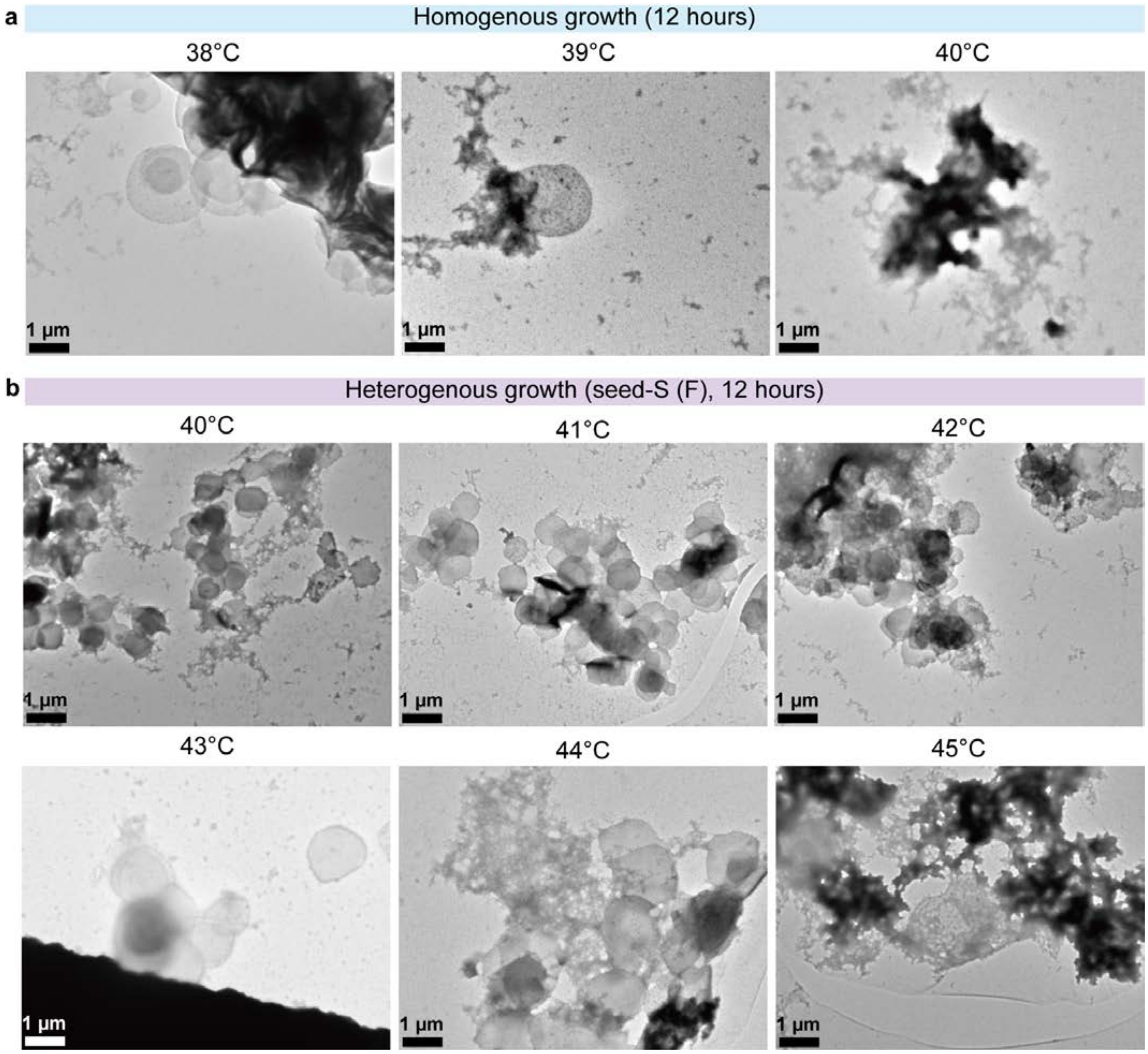

Supplementary Fig. 28 | Temperature optimization for seeded growth of square SST superlattices. a, For (unseeded) homogeneous growth, sublattices form only at 38°C or 39°C over 12 hours. At 40°C, no lattice growth occurs within 12 hours. **b,** For heterogeneous growth (seed-S (F)), sublattices develop even at 45°C, close to the melting temperature of 48°C. 40°C is identified as the optimal incubation temperature, at which homogeneous growth does not occur within 12 hours, whereas heterogeneous growth demonstrates high yield.

Homogenous growth (unseeded)

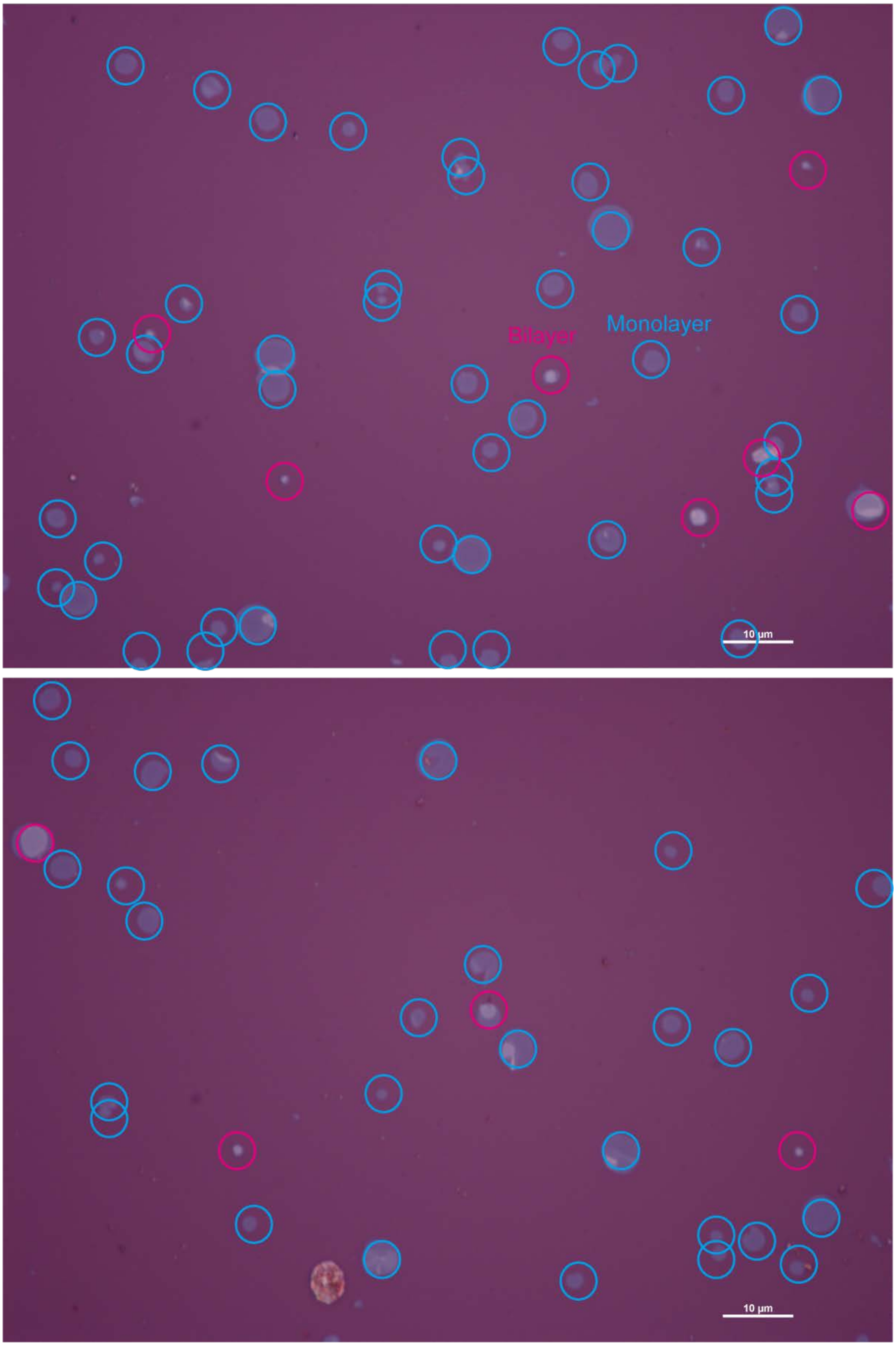

Supplementary Fig. 29 | Bright-field optical microscopy images of square SST sublattices without seeds. The monolayers and bilayers are highlighted in blue and pink, respectively. $N_{monolayer}$ = 73; $N_{bilayer}$ = 11.

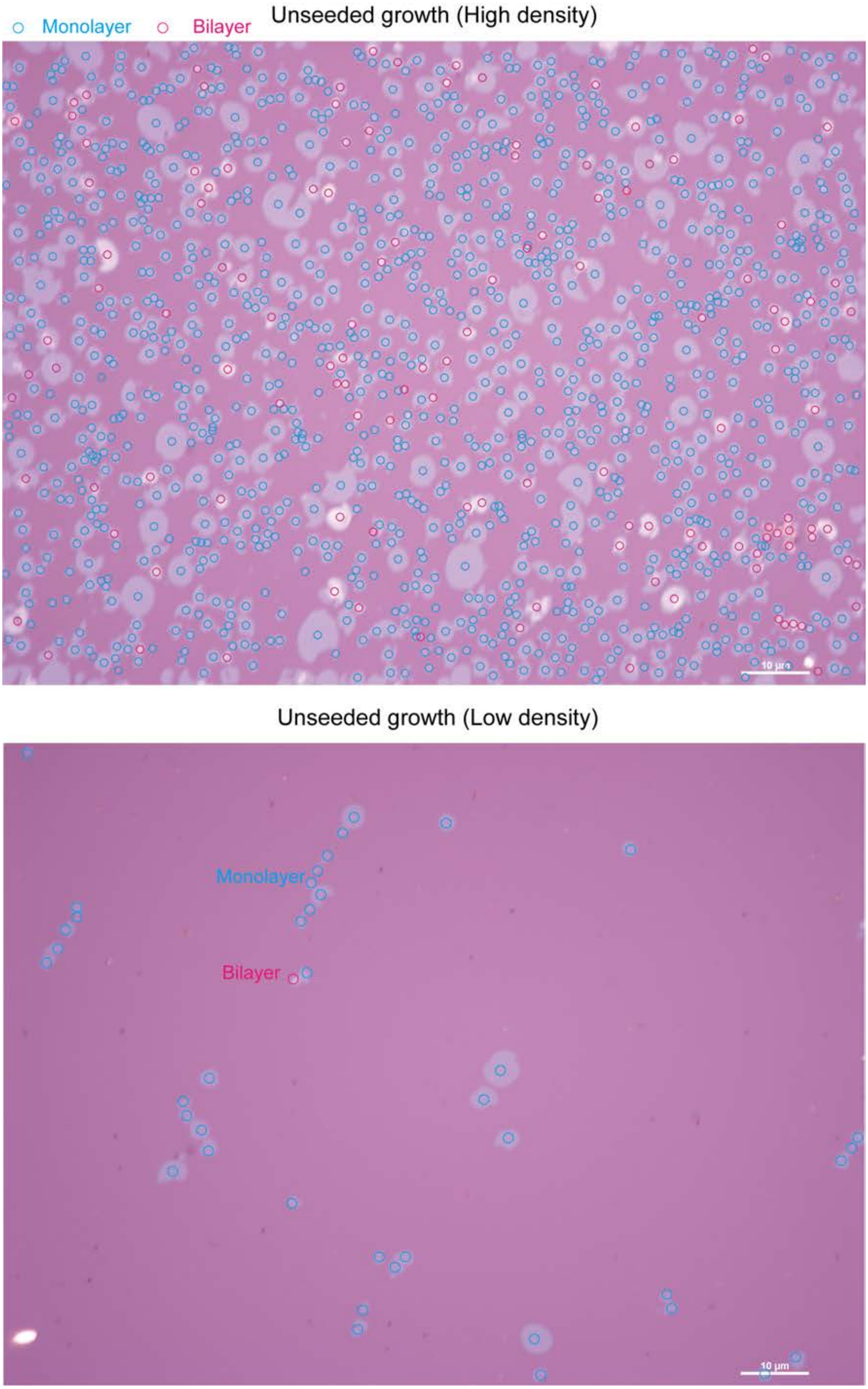

Supplementary Fig. 30 | Control experiments. The results show that the frequency of bilayer formation due to random monolayer overlap decreases from ~ 10 % (N = 1314) to ~2 % *(N = 42)*, as the structural density is reduced.

Heterogenous growth on Z_1 and Z_3 segments of seed-S(F), 0.1 nM, 40°C, 24 hours

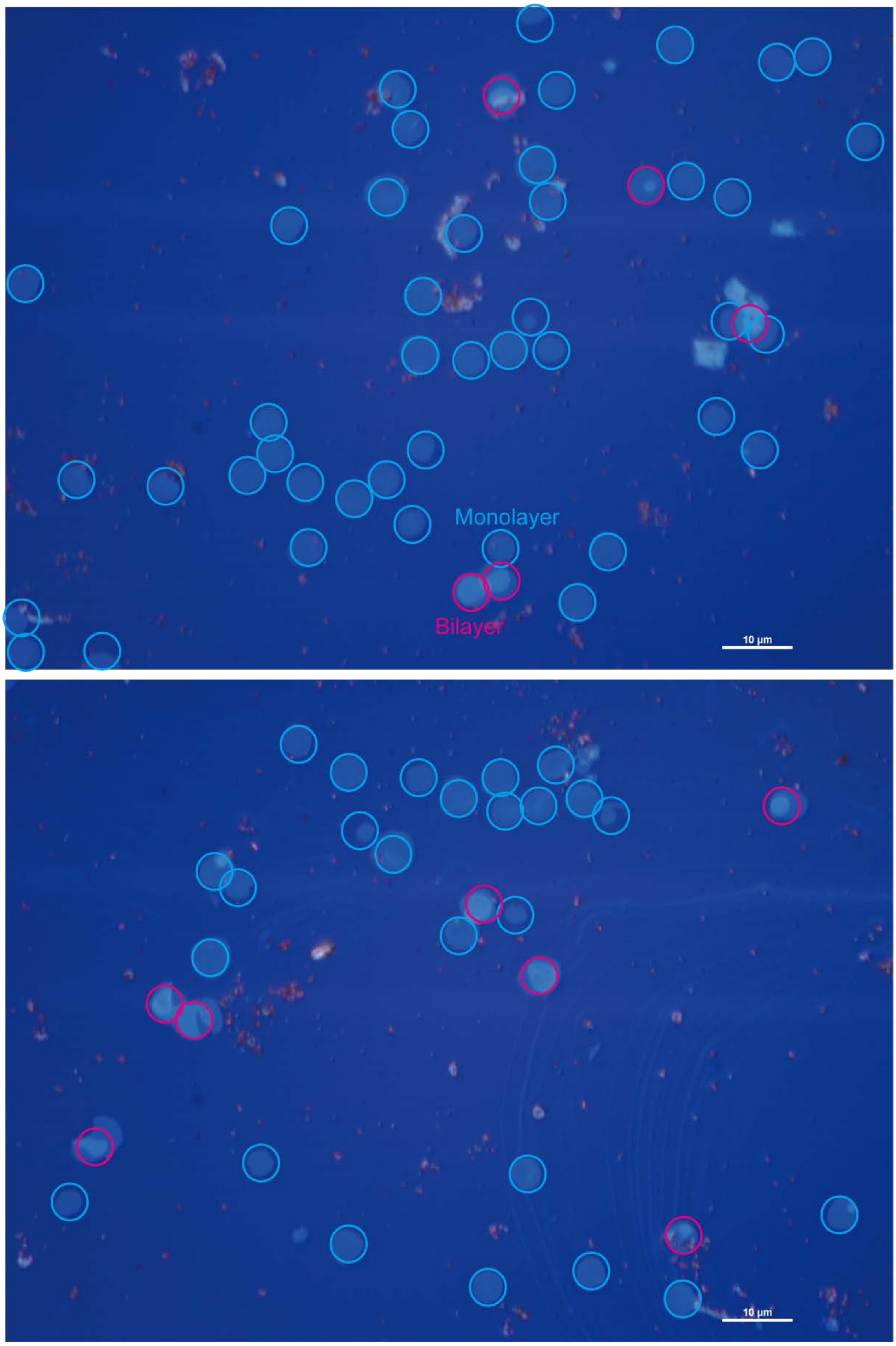

Supplementary Fig. 31 | Bright-field optical microscopy images of square bilayers formed with seed-S (F), 0.1 nM at 40 °C. $N_{\text{monolayer}}$ = 58 and N_{bilayer} = 12.

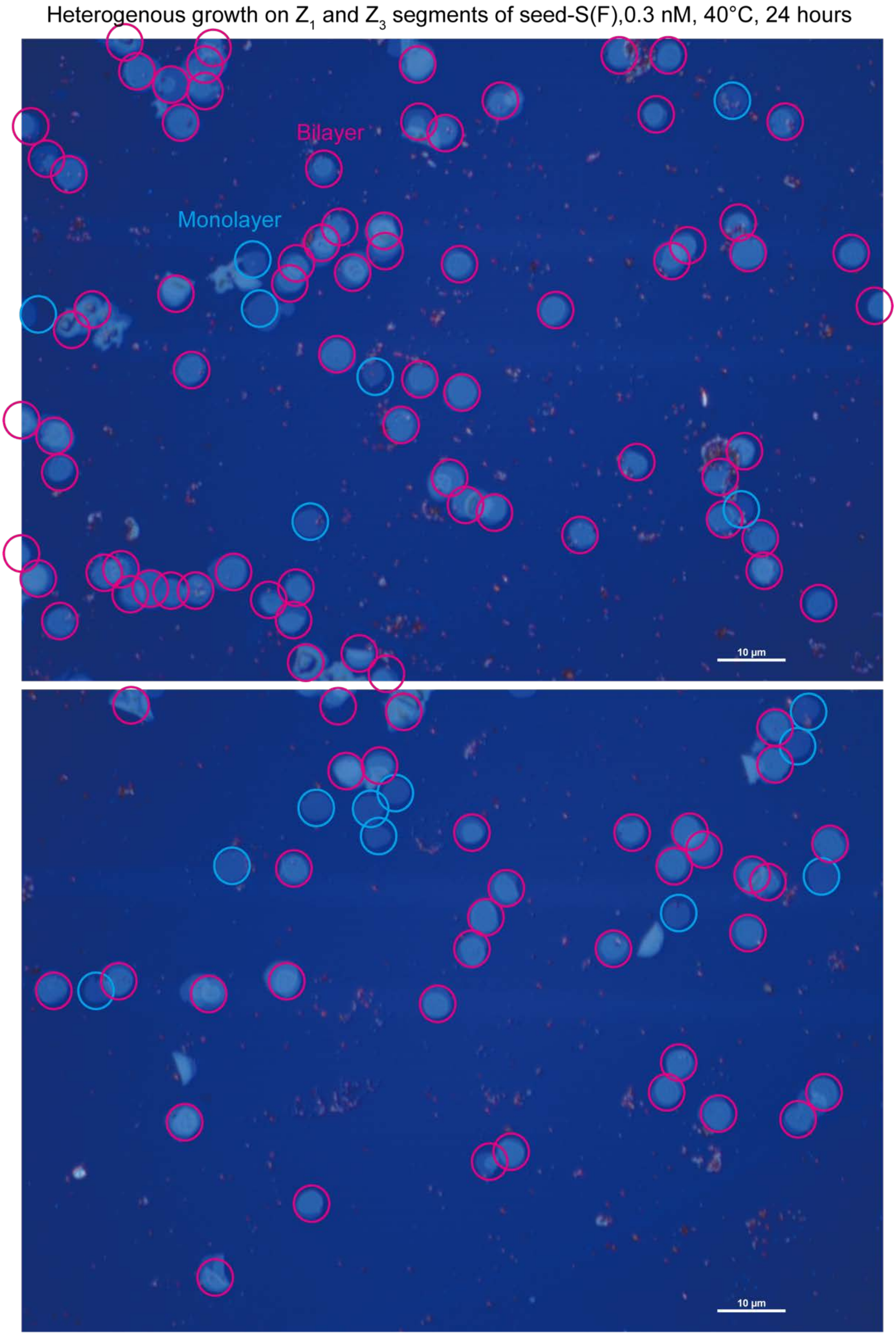

Supplementary Fig. 32 | Bright-field optical microscopy images of square bilayers formed with seed-S (F), 0.3 nM at 40 °C. $N_{\text{monolayer}}$ = 17 and N_{bilayer} = 109.

Heterogenous growth on Z_1 and Z_3 segments of seed-S(F),1.1 nM, 40°C, 24 hours

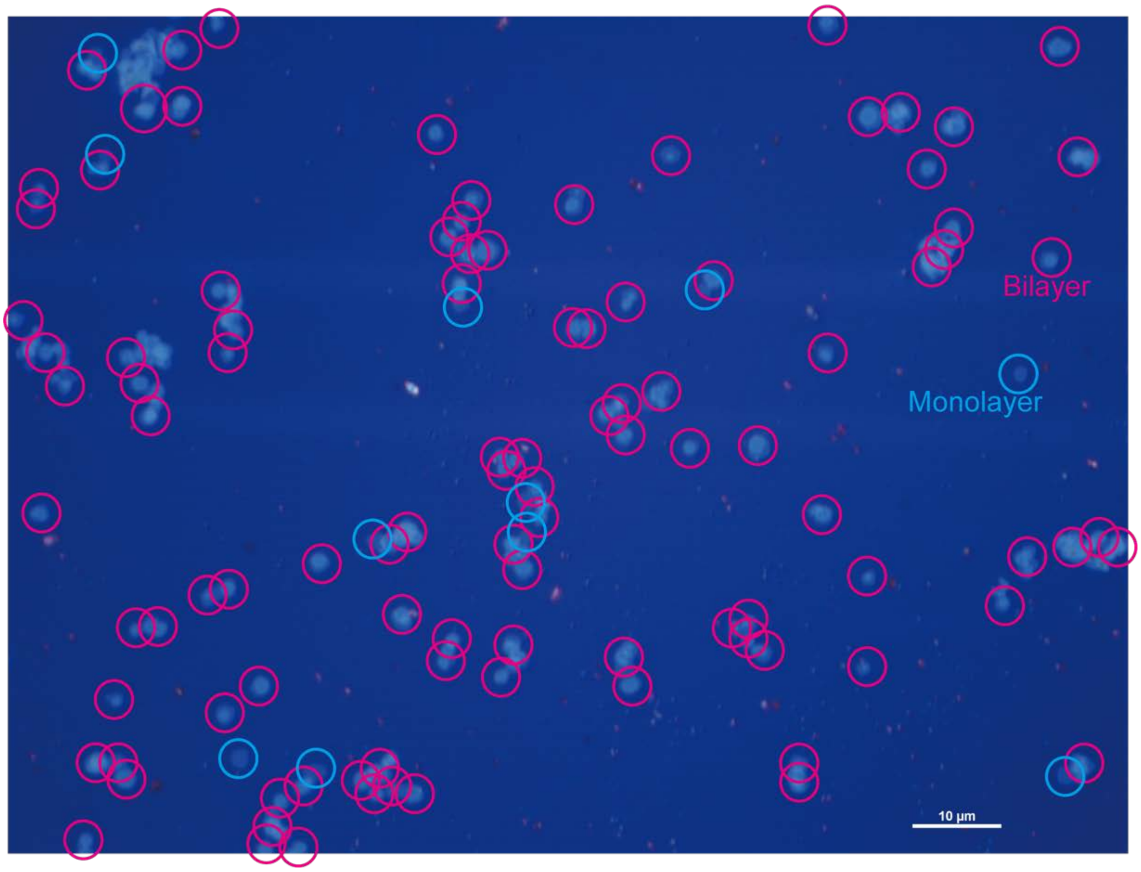

Supplementary Fig. 33 | Bright-field optical microscopy images of square bilayers formed with seed-S (F), 1.1 nM at 40 °C. $N_{\mathrm{monolayer}}$ = 11 and N_{bilayer} = 102.

Heterogenous growth on Z_1 and Z_3 segments of seed-S(F),1.1 nM, 34°C, 24 hours

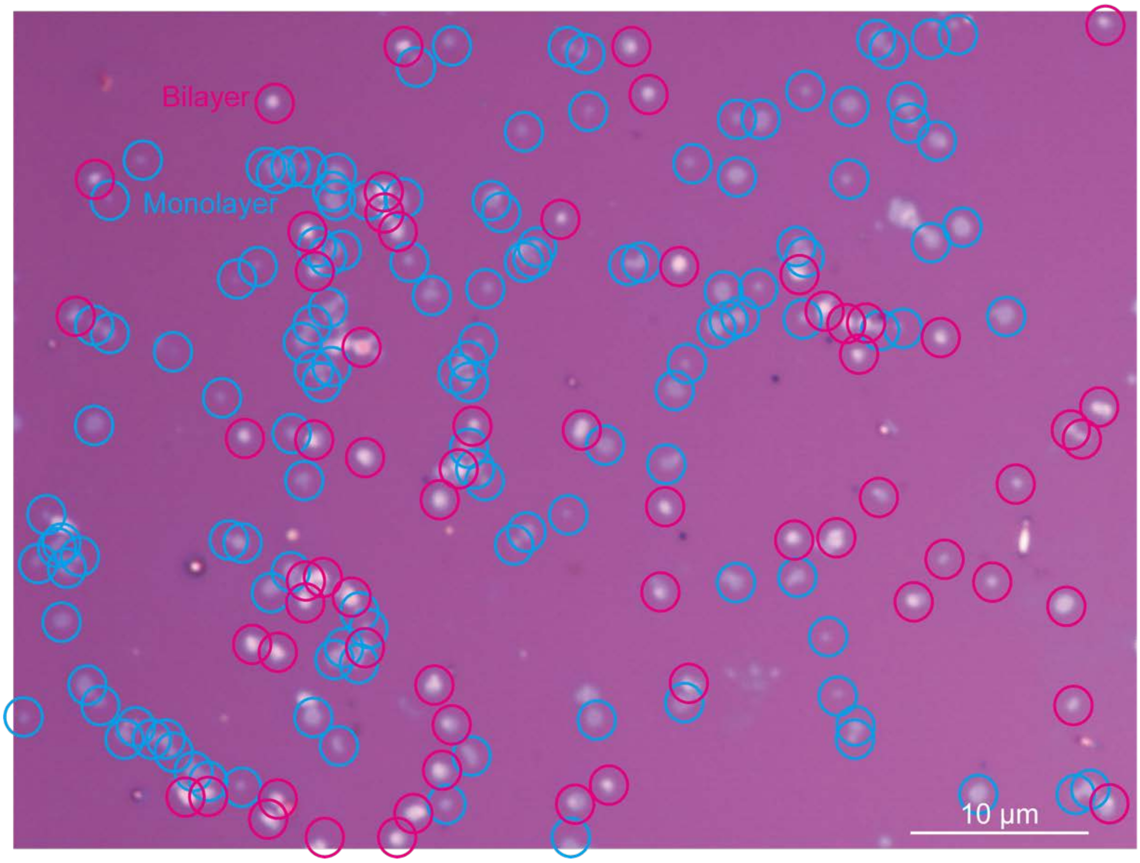

Supplementary Fig. 34 | Bright-field optical microscopy images of square bilayers formed with seed-S (F), 1.1 nM at 34 °C. $N_{monolayer}$ = 130 and $N_{bilayer}$ = 63.

Heterogenous growth on Z_1 and Z_3 segments of seed-S(P1), 1.1 nM, 40 °C, 24 hours

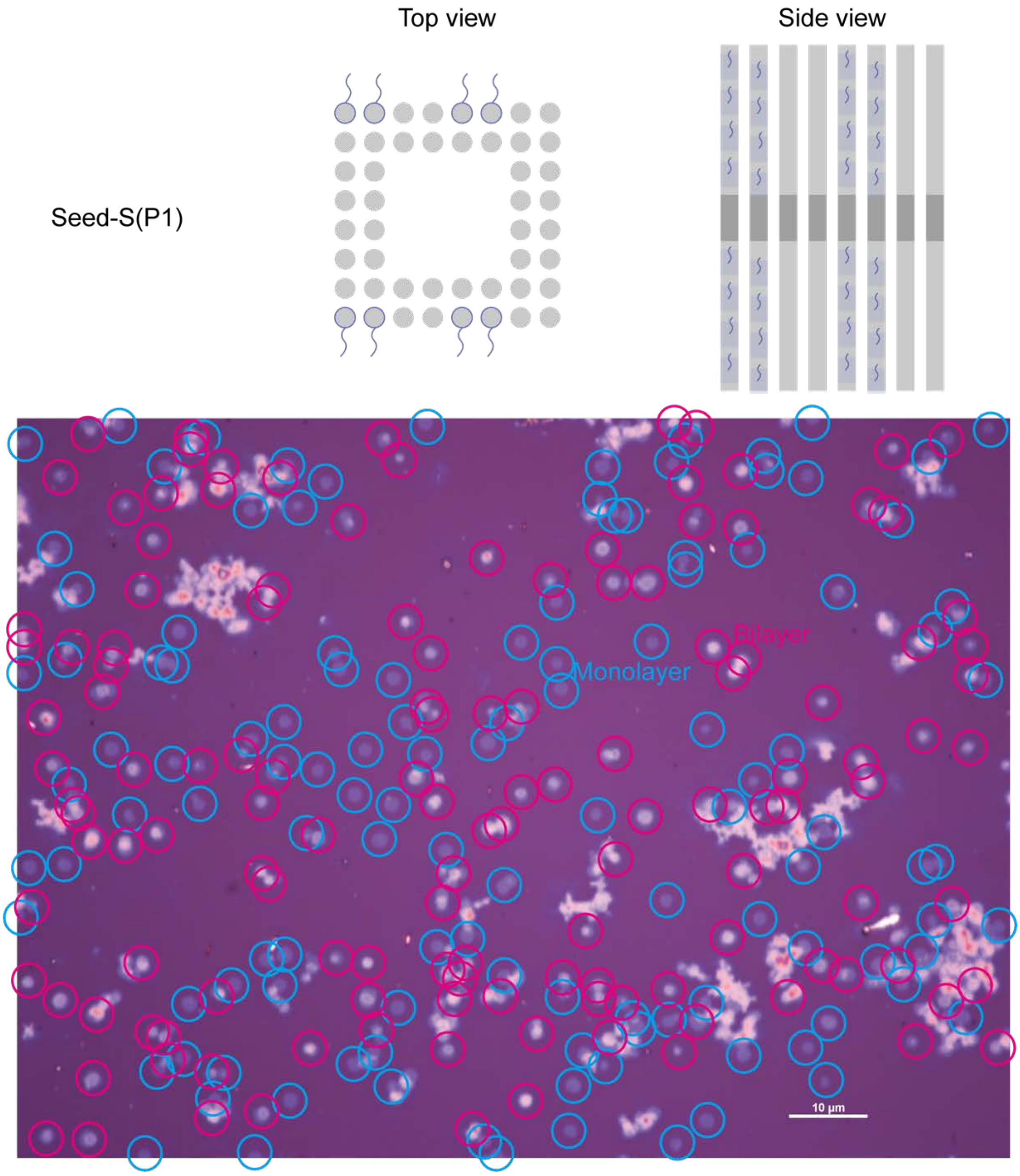

Supplementary Fig. 35 | Bright-field optical microscopy images of square bilayers formed with seed-S (P1), 1.1 nM at 40 °C. $N_{\text{monolayer}} = 121$ and $N_{\text{bilayer}} = 135$.

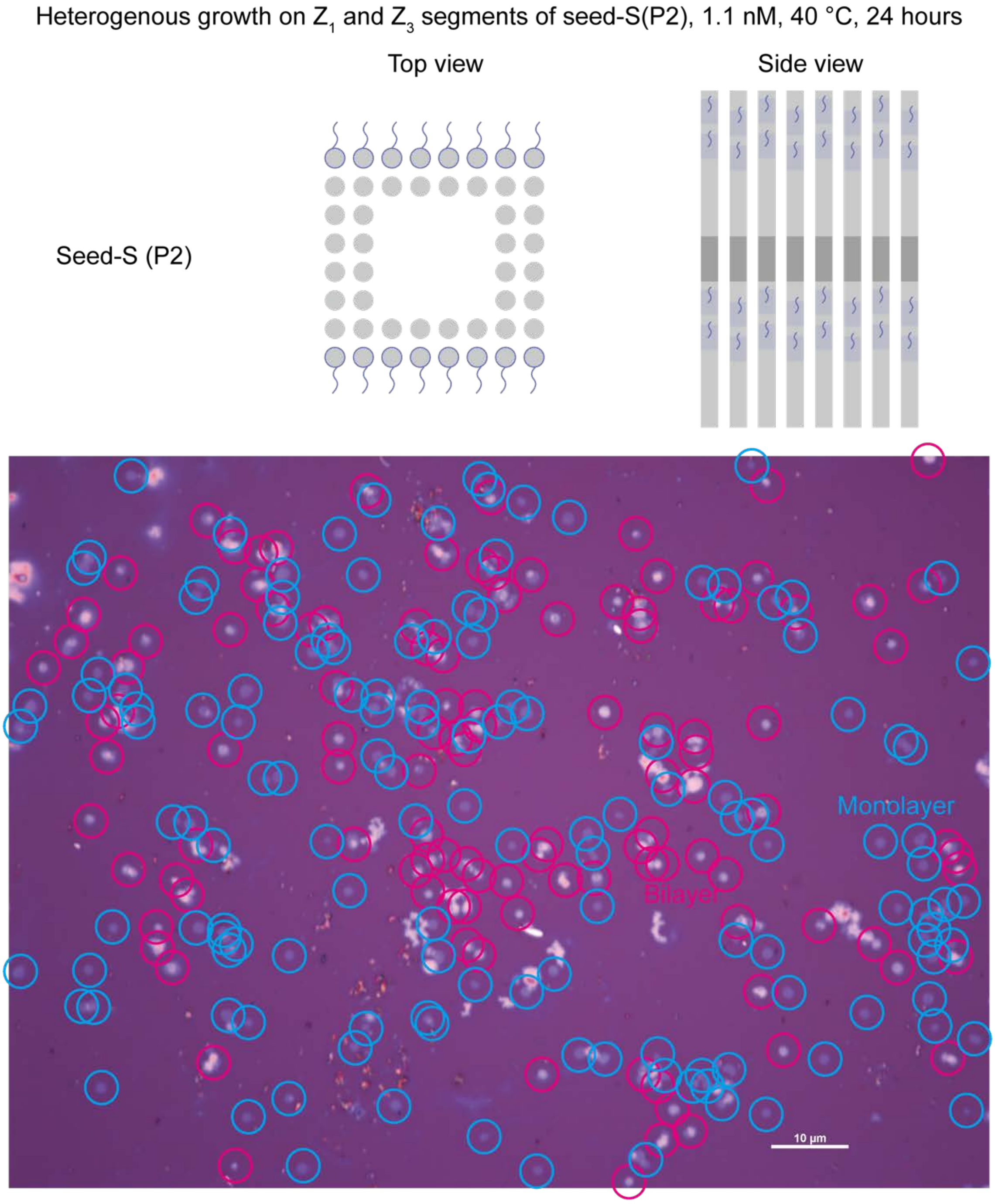

Supplementary Fig. 36 | Bright-field optical microscopy images of square bilayers formed with seed-S (P2), 1.1 nM at 40 °C. $N_{\text{monolayer}}$ = 138 and N_{bilayer} = 114.

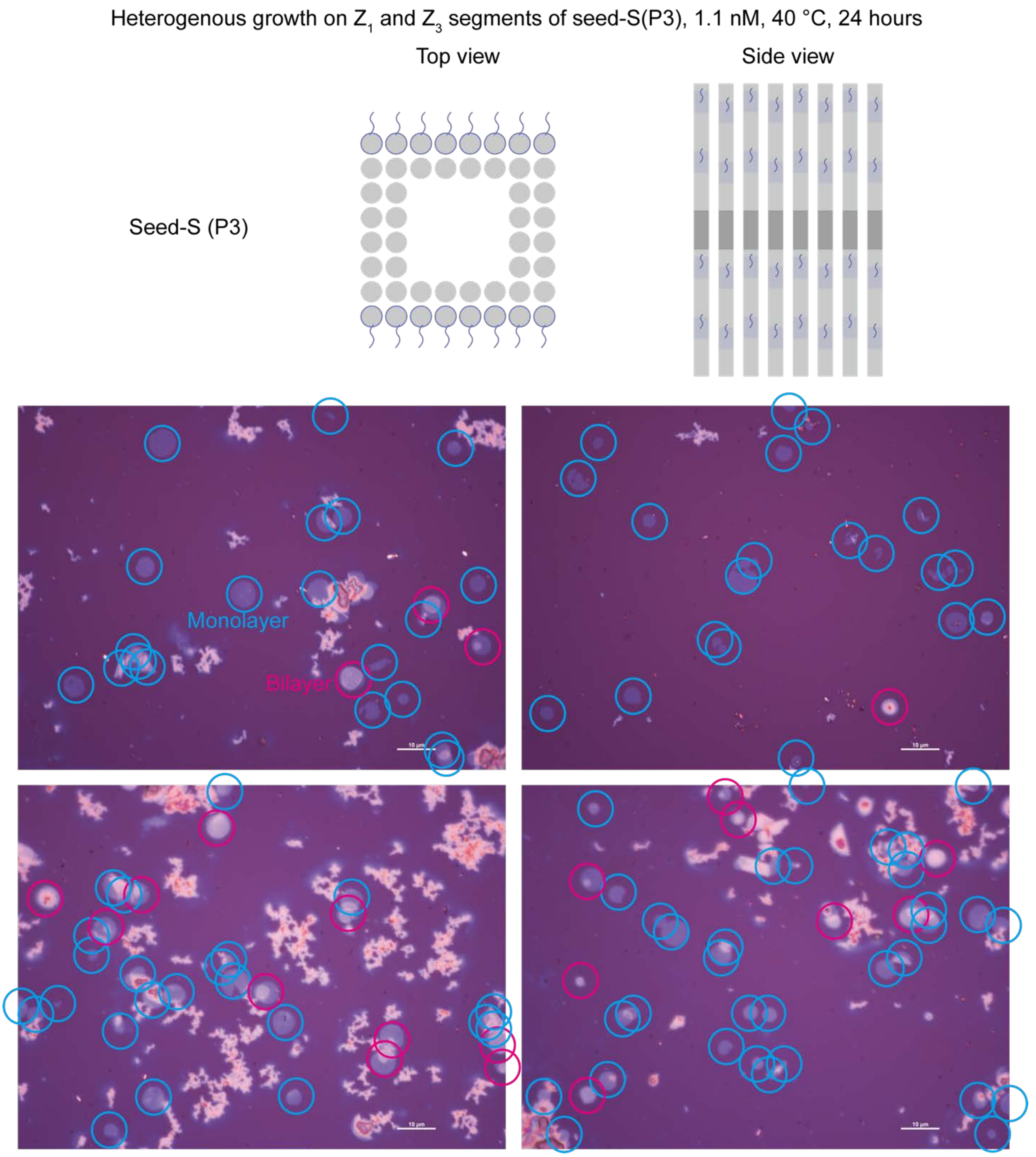

Supplementary Fig. 37 | Bright-field optical microscopy images of square bilayers formed with seed-S (P3), 1.1 nM at 40 °C. $N_{\text{monolayer}}$ = 96 and N_{bilayer} = 22.

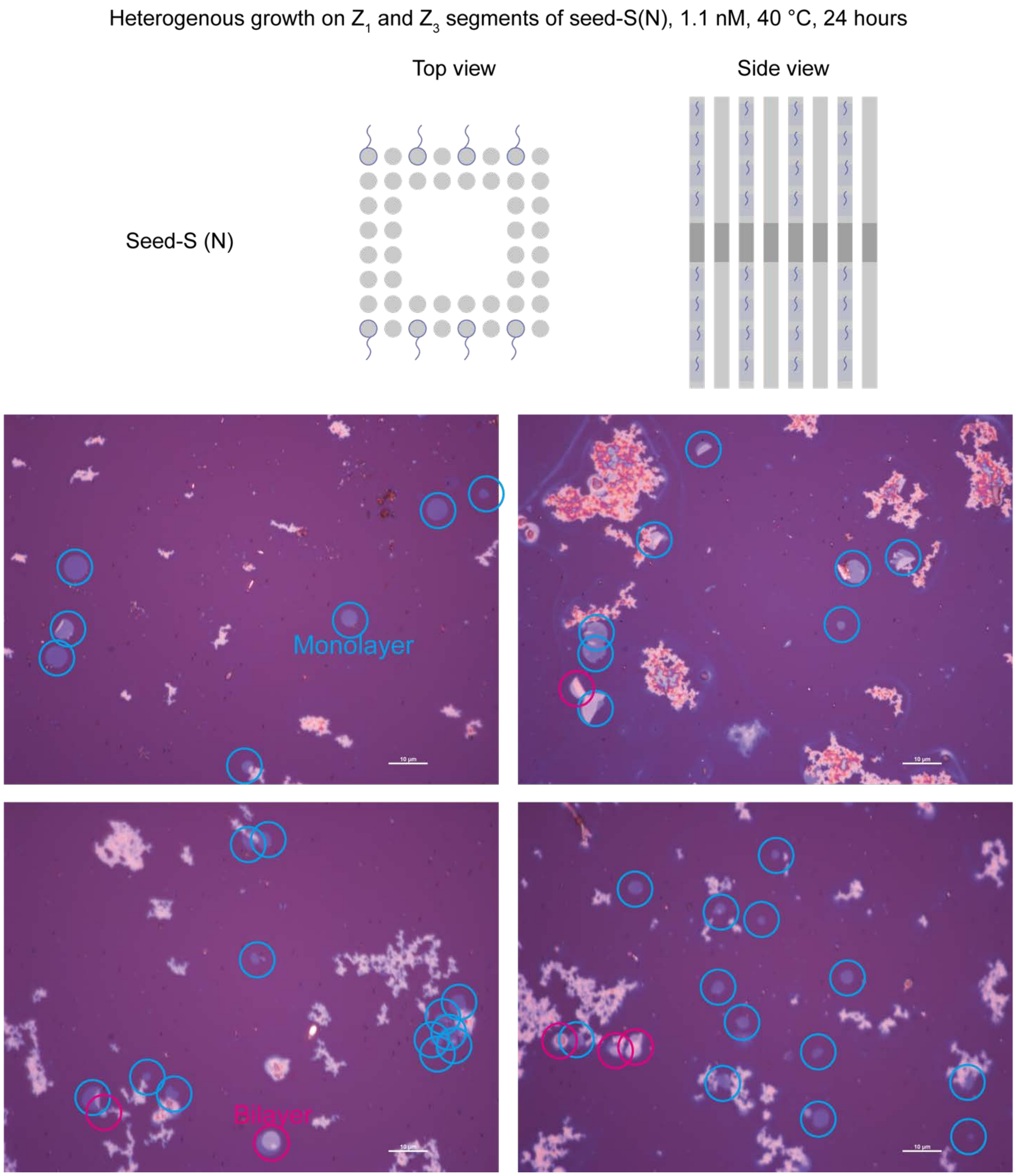

Supplementary Fig. 38 | Bright-field optical microscopy images of square bilayers formed with seed-S (N), 1.1 nM at 40 °C. $N_{\mathrm{monolayer}} = 40$ and $N_{\mathrm{bilayer}} = 6$.

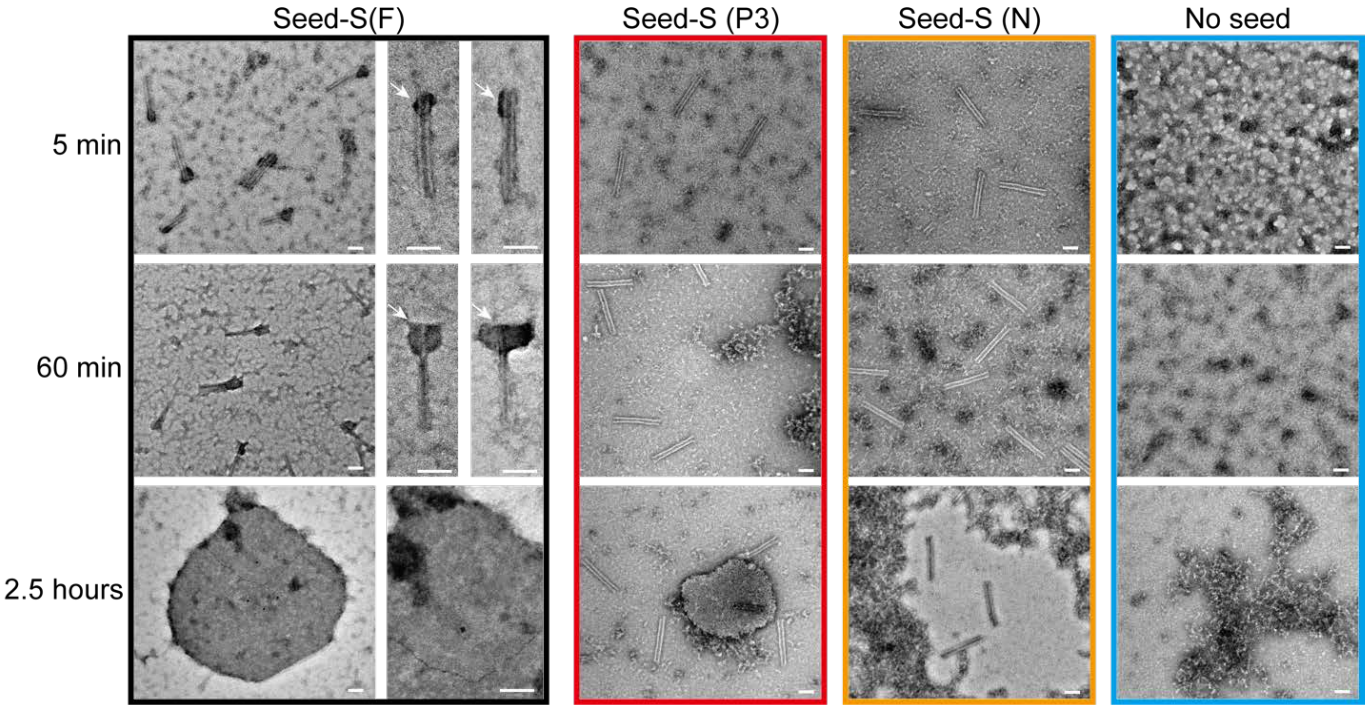

Supplementary Fig. 39 | Initial nucleation and growth of SSTs on seed-S (F), seed-S (P3), seed-S (N), and (unseeded) homogenous growth. Captures were only extended from the Z_1 segment. TEM images were taken at 5 min, 60 min, and 2.5 hours. At 5 min, only seed-S (F) shows SST attachment on the Z_1 segment, while no SST attachment is visible on seed-S (P3) and seed-S (N). After 60 min, SSTs adhere to the two facing surfaces (north and south surfaces in **Supplementary Fig. 24**) of seed-S(F), thus lying flat on the TEM grid. No SST attachment is observed on seed-S (P3) and seed-S (N). After 2.5 hours, lattices (~ hundreds of nm) form for seed-S (F), with hollow seeds visible. Free seeds are absent. For seed-S(P3), free seeds without SST attachment are observed. Very few small lattices are visible. Seed-S (N) shows many aggregates and free seeds. For (unseeded) homogenous growth, only aggregates are visible, increasing in size over time. Seed concentration = 1.1 nM. Scale bars, 50 nm.

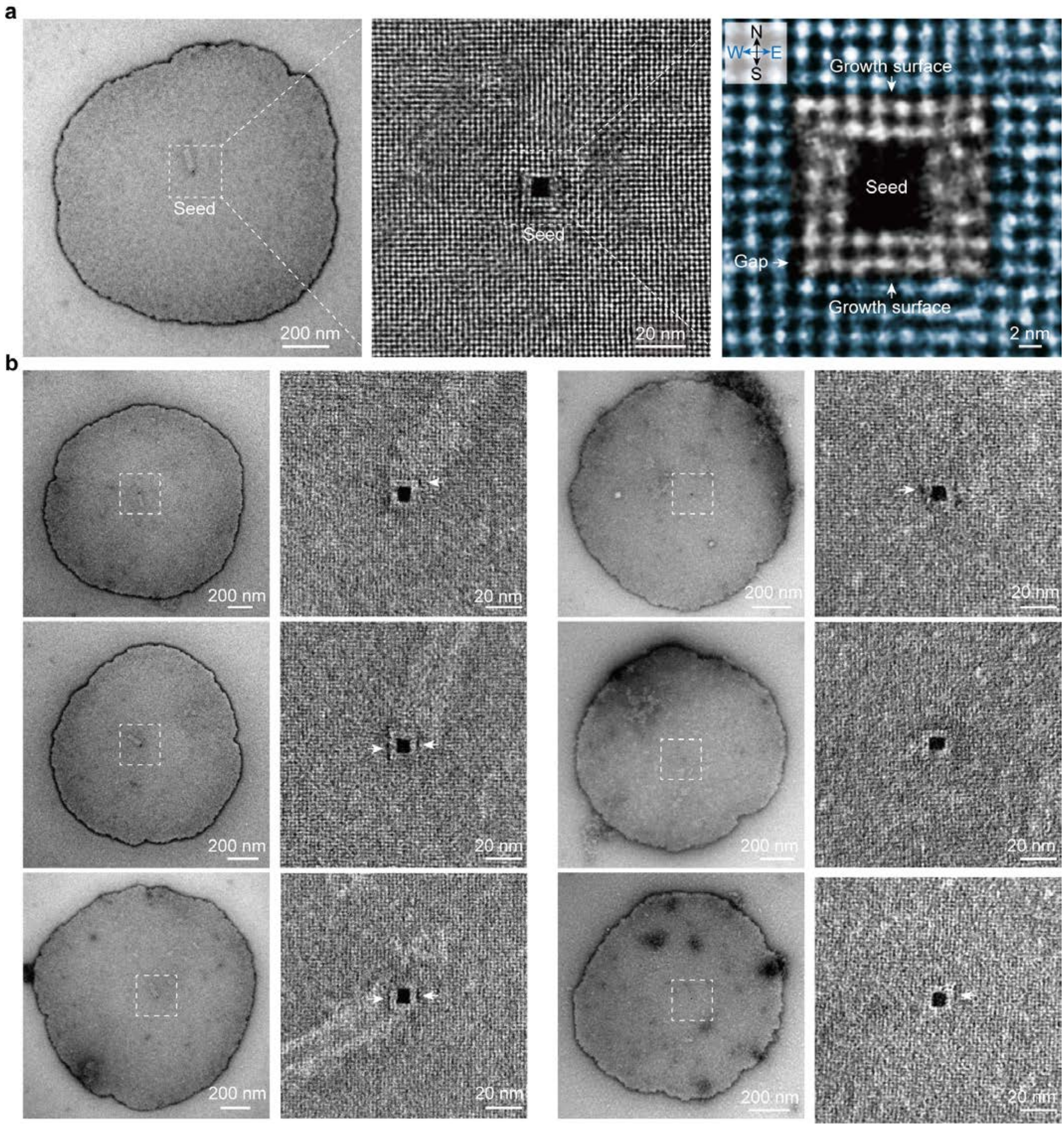

Supplementary Fig. 40 | Seeded growth of square lattice monolayers at 1.1 nM seed-S (F), 40°C, for 24 hours. Captures were only extended from the Z_1 segment. **a,** Hollow seed (8-helix × 8-helix rim with 4-helix × 4-helix pore) is clearly visible. The SST lattice aligns with the seed lattice orientation, indicating that the twist of the seed in Z_1 is alleviated by the SST lattice. Gaps between the seed and SST lattice on the capture-free seed surfaces are also identified. **b,** Additional TEM images.

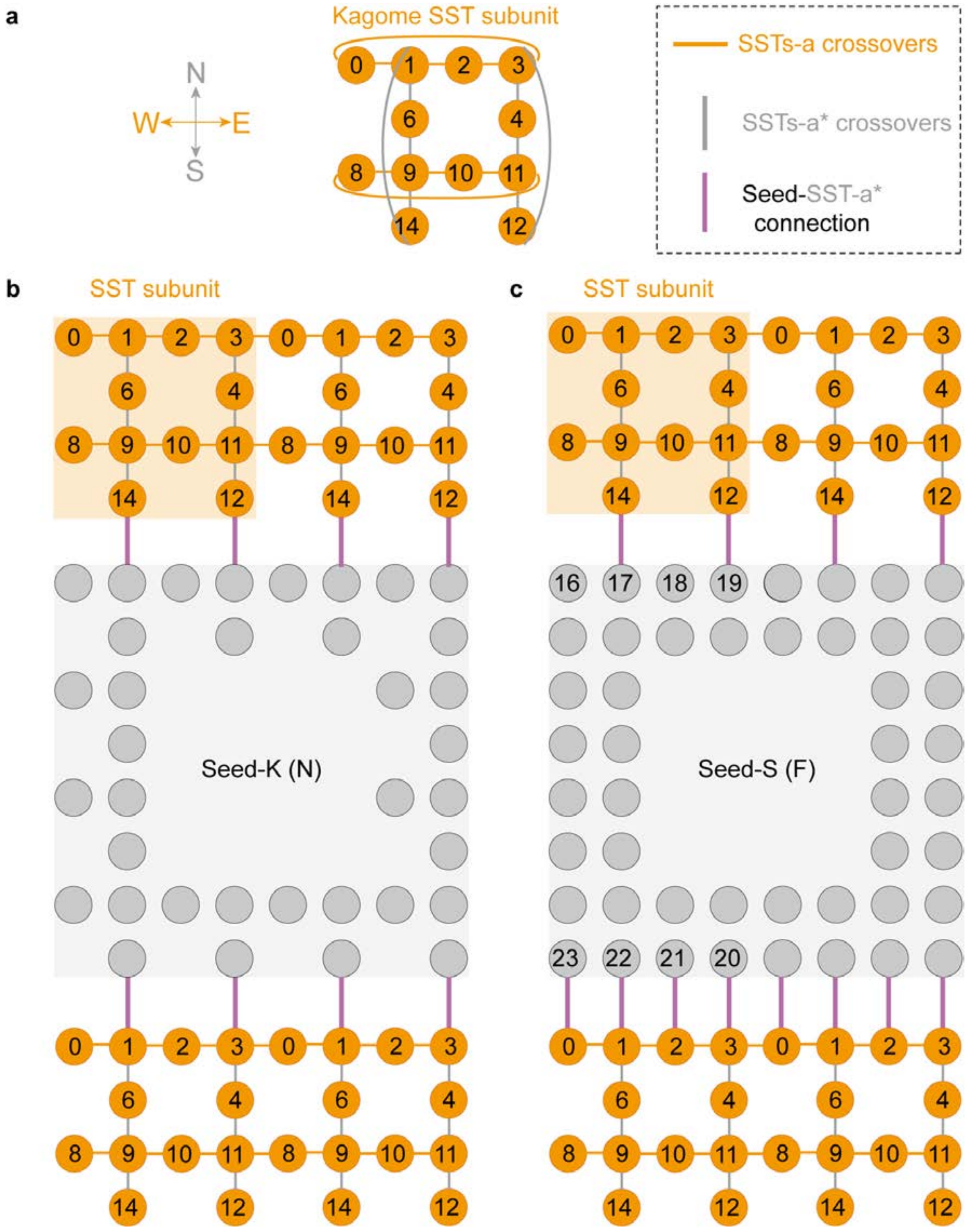

Supplementary Fig. 41 | Seed design for the kagome superlattice. a, SST subunit of the kagome sublattice. The kagome sublattice is transformed from a loosely packed square sublattice. Its subunit thus retains crossover and domain designs similar to the square subunit. Half crossovers of both SSTs-a* and SSTs-a are removed relative to the square subunit. **b,** Capture design for the kagome-lattice seed-K (N) adopts the same helix arrangement as the kagome SST subunit, yielding the same capture pattern as seen in seed-S (N). **c,** A square-lattice seed, termed seed-S (K), is employed, so that helices 28-31 of the seed bind helices 0-3 of the SST subunit in a fully-cooperative mode.

a

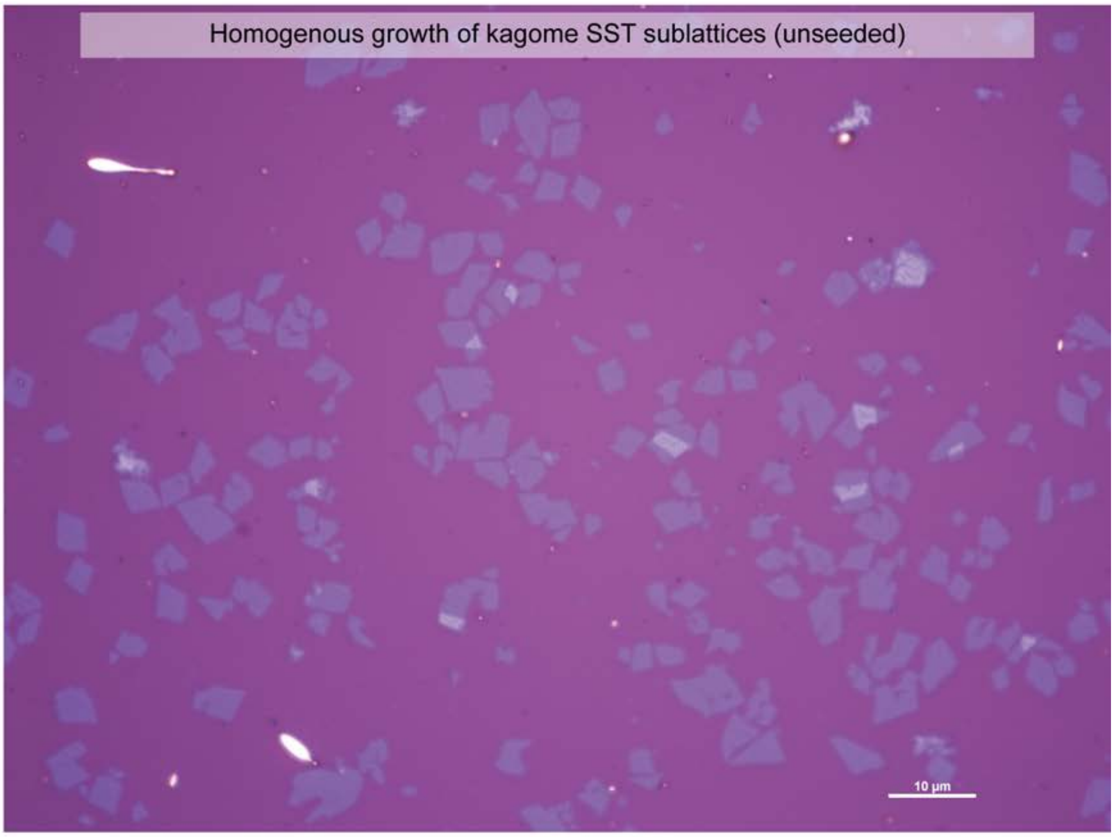
Homogenous growth of kagome SST sublattices (unseeded)
10 μm

b

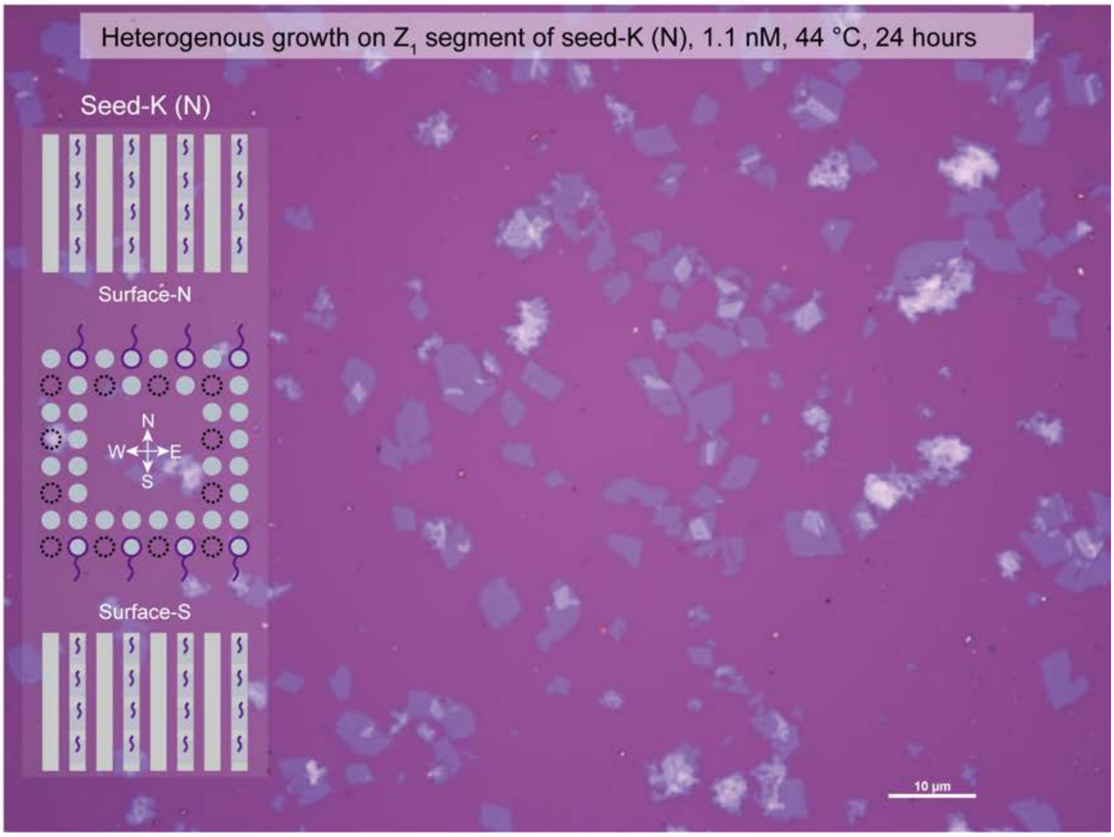
Heterogenous growth on Z1 segment of seed-K (N), 1.1 nM, 44 °C, 24 hours
Seed-K (N)
Surface-N
N
W
E
S
Surface-S
10 μm

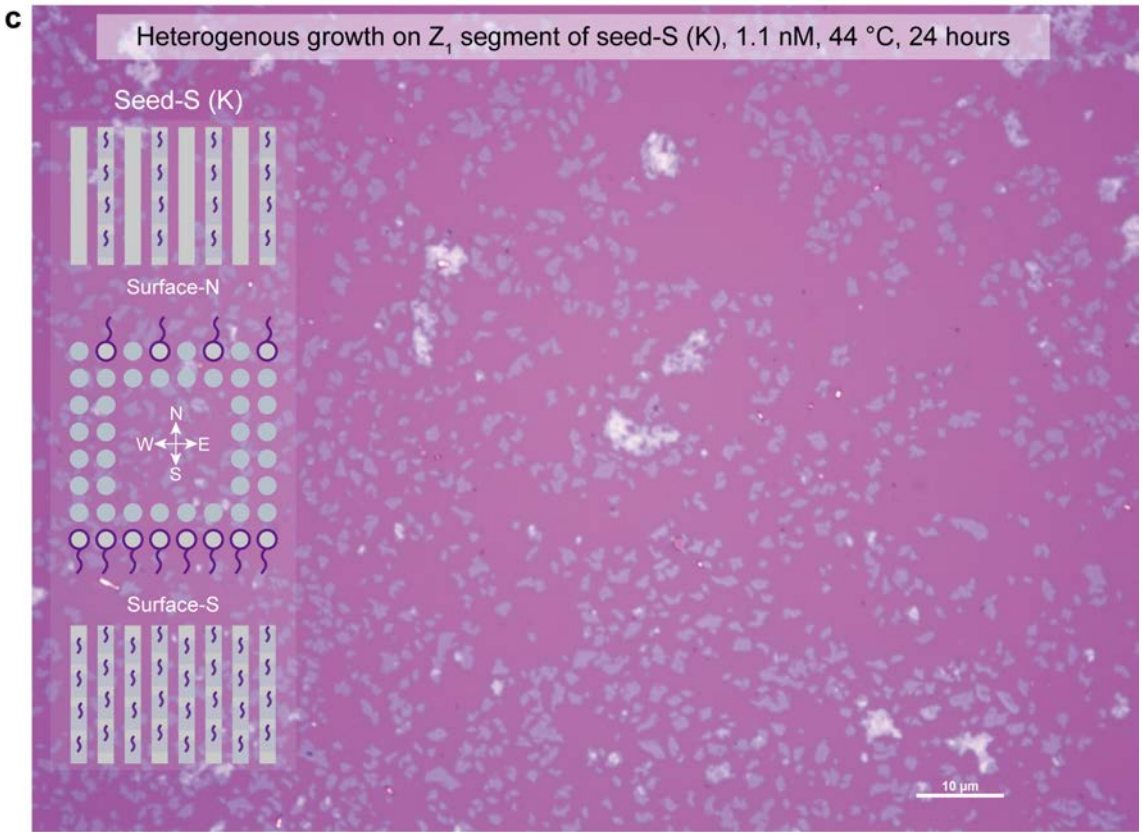

Supplementary Fig. 42 | Seeded growth of kagome monolayers with seed-K (N) and seed-S (K). a, Homogenous growth that forms monolayers is used as a control. **b,** With seed-K (N). **c,** With seed-S (K). Captures are extended only from the Z_1 segment of the seed. Seed concentration was 1.1 nM with an incubation temperature of 44°C for 24 hours.

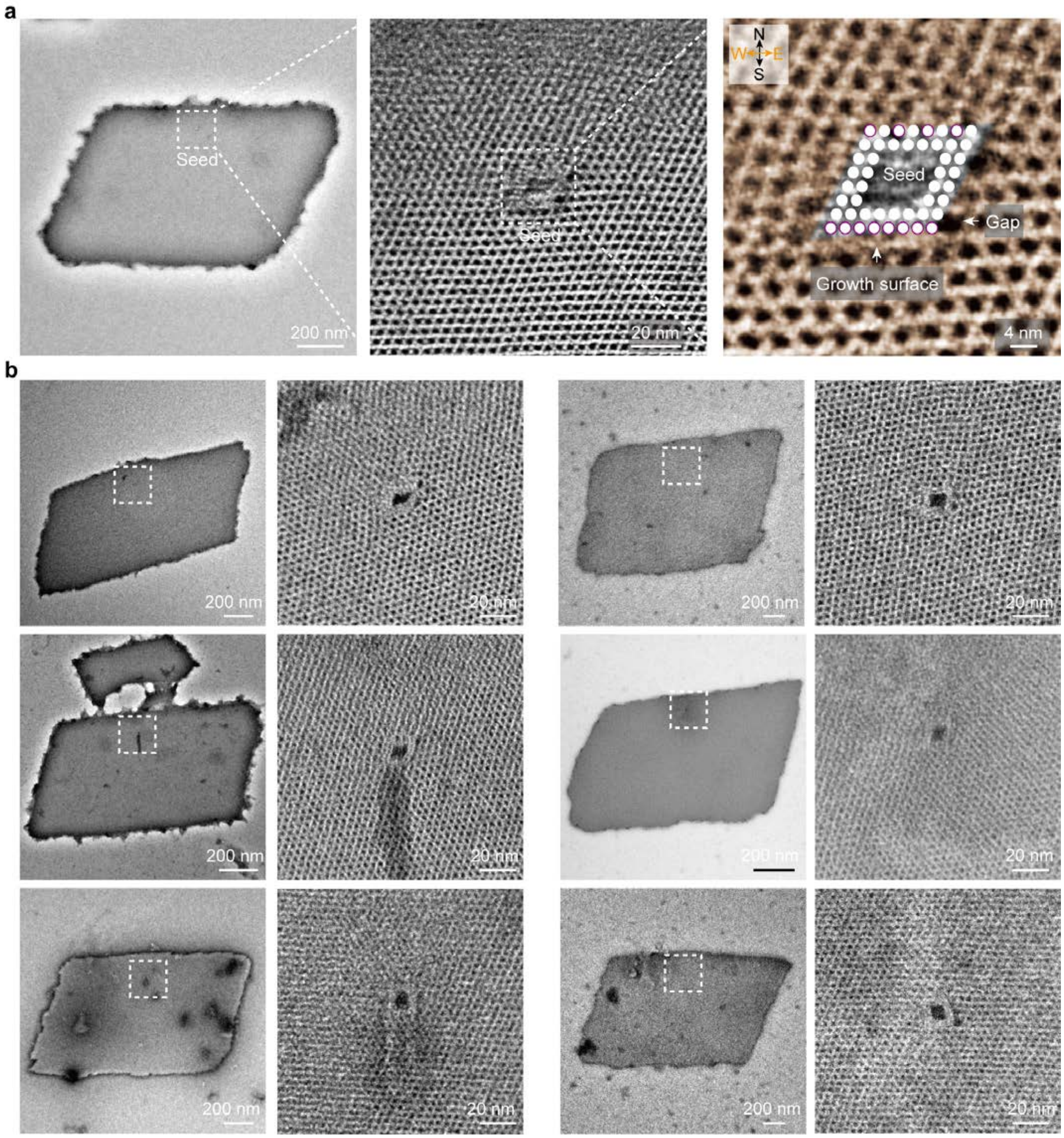

Supplementary Fig. 43 | Seeded growth of kagome monolayers with seed-S (K) at a seed concentration of 1.1 nM with incubation at 44°C for 24 hours. **a,** Transformation in symmetry from square to rhomboid within the seed is clearly identified, conforming with the kagome sublattice. Growth surfaces of the seed and the gaps between the seed and sublattice on capture-free surfaces are marked by white arrows. **b,** Additional TEM images.

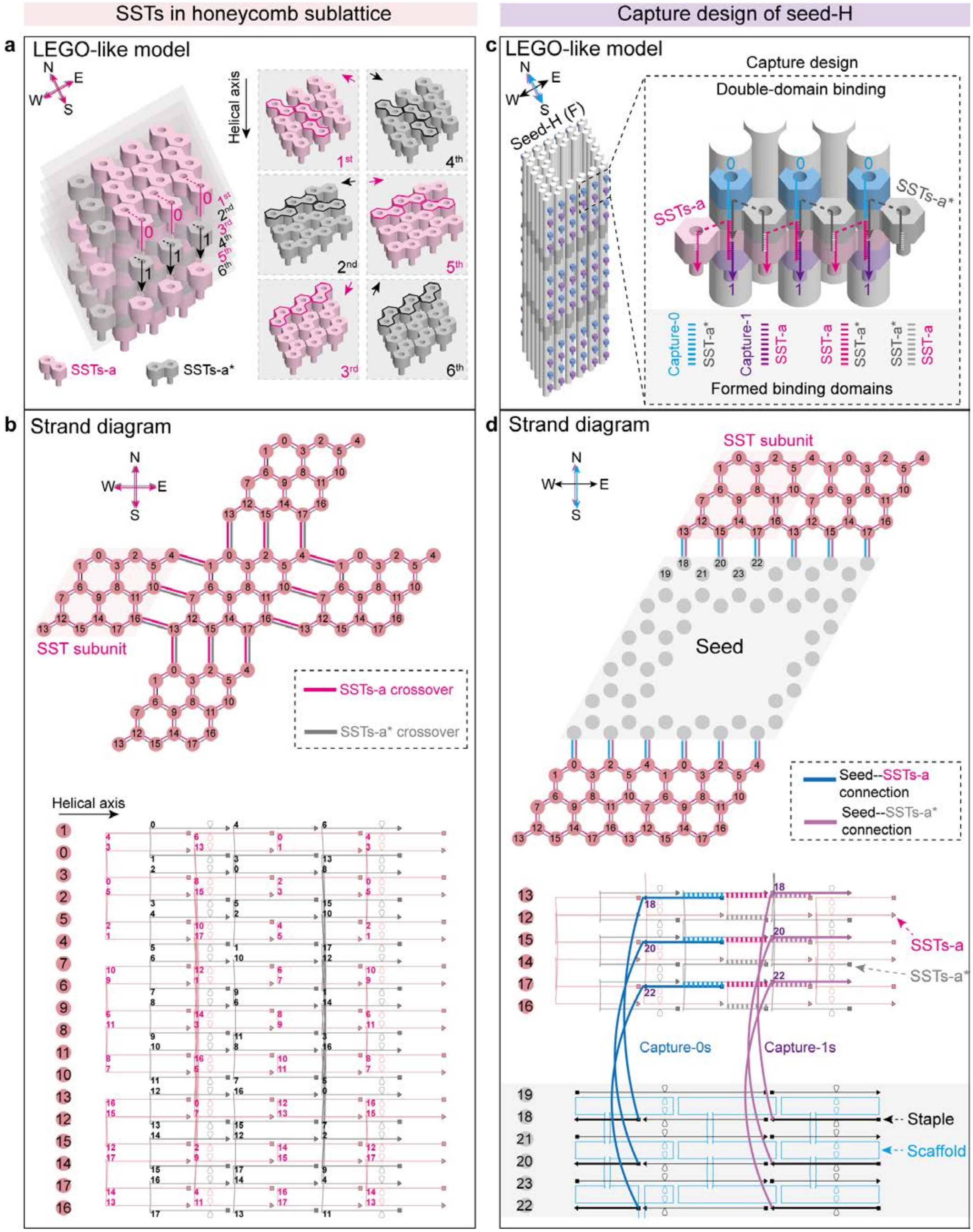

Supplementary Fig. 44 | Capture design of seed-H for seeded growth of honecomb SST superlattices. a, Lego-like model of SSTs in the honeycomb sublattice, which adopt a different design compared to square and kagome SST sublattices. Since the 9-nt domain defines a rotation angle of 300°, 6 layers of SSTs make a full rotation of 360°, 60° per layer along the helical axis. SSTs-a and SSTs-a* alternate layer by layer, with a-layers and a*-layers appearing in the opposite directions every three layers (*e.g.*, 1st layer and 4th layer). **b,** Strand diagram of SSTs in the honeycomb sublattice. Each helix has unbinding domains from both SSTs-a and SSTs-a* in all three directions to bind neighboring SSTs. This poses a challenge for designing the captures

on the seed, as both ‘scaffold’ captures and staple captures are required. **c**, **d,** To address this, we extend equivalent ‘scaffold’ captures using staples (capture-0s), though producing parallel staple-capture crossovers, as shown in the strand diagram **d**. With the help of capture-0s, double-domain binding can be realized, thus satisfying the fully-cooperative growth mode (seed-H (F)). LEGO-like model **c** shows the capture number and pattern, in which surfaces-north/south have full extended captures including capture-0s and capture-1s, while surfaces-east/west have no captures. Inset illustrates initial capturing of SSTs. For each SST captured, 2× effective binding domains are formed, one from the capture-SST binding and the other from the SST-SST binding. Thus, an indirect double-domain binding is established for seed-H (F). Without capture-0s, double-domain binding cannot be realized (seed-H (N)). The captures, captured SSTs and corresponding binding domains are also shown in the strand diagram. Notably, each capture-0 cooperates with two capture-1s both in the same helix and in the neighboring helix, resulting in a three-capture group rather than the two-capture group on seed-S (F).

a

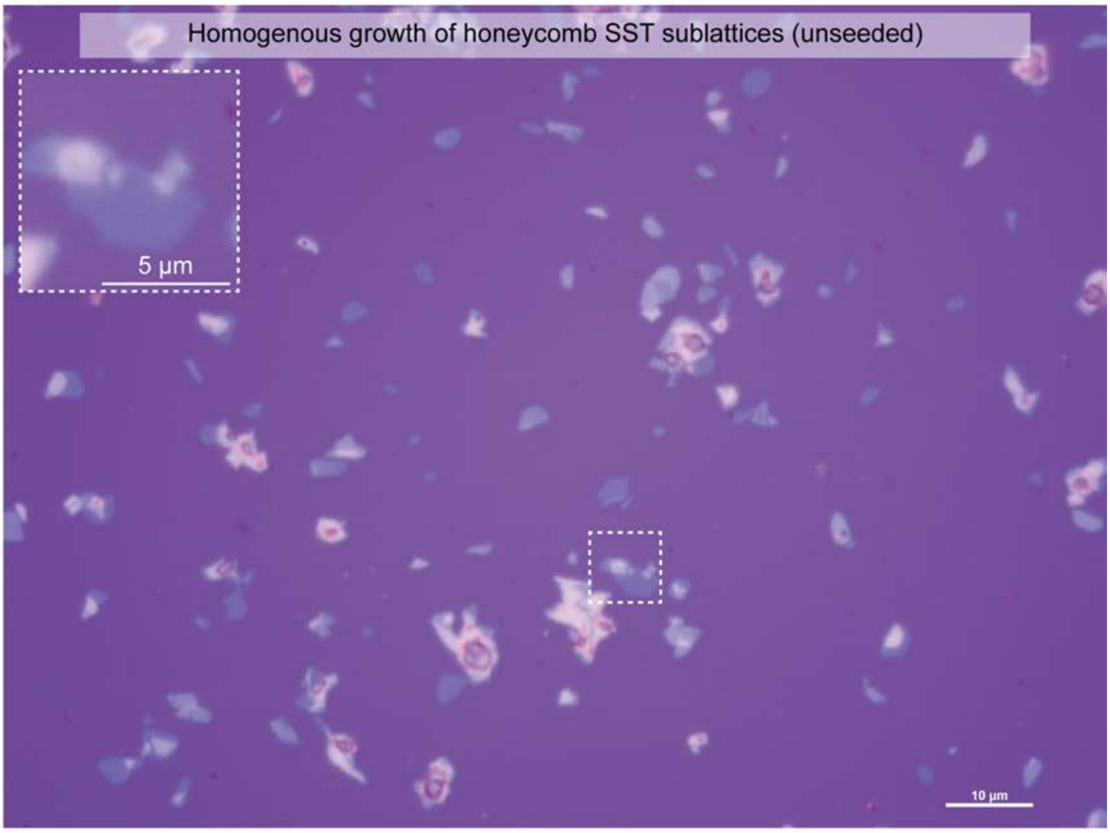

Homogenous growth of honeycomb SST sublattices (unseeded)
5 μm
10 μm

b

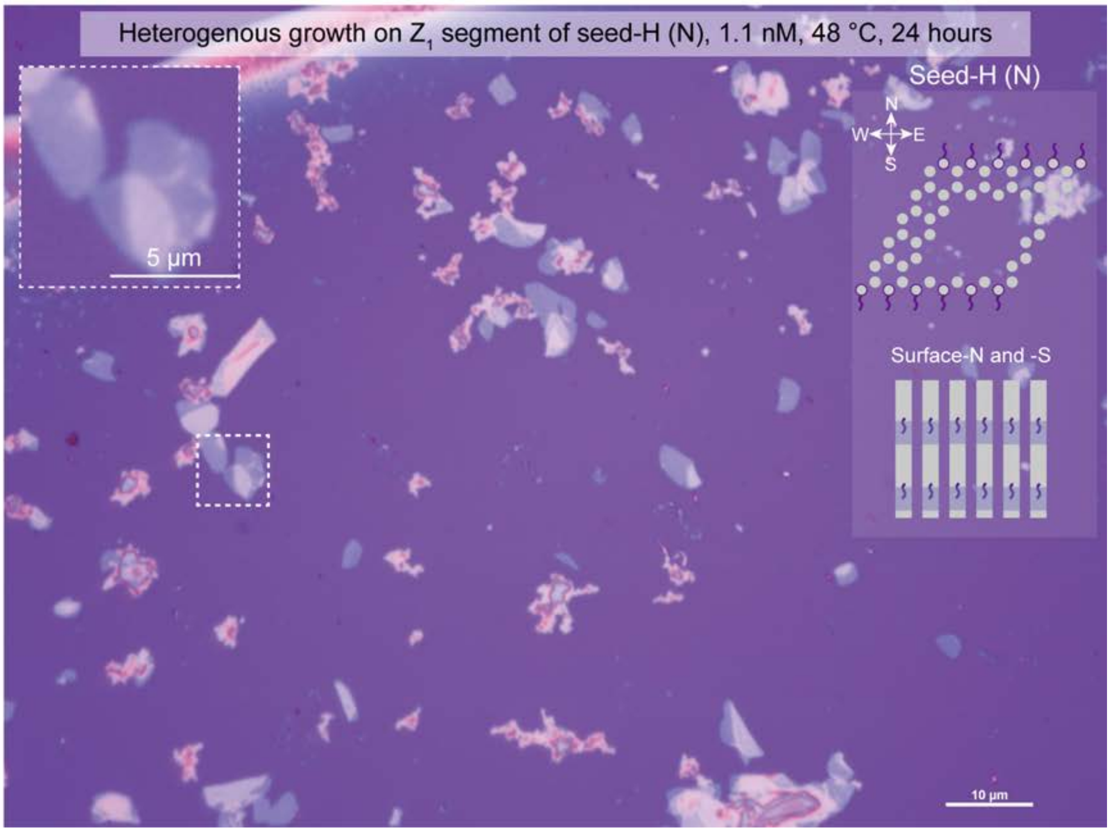

Heterogenous growth on Z1 segment of seed-H (N), 1.1 nM, 48 °C, 24 hours
5 μm
Seed-H (N)
N
W
E
S
Surface-N and -S
10 μm

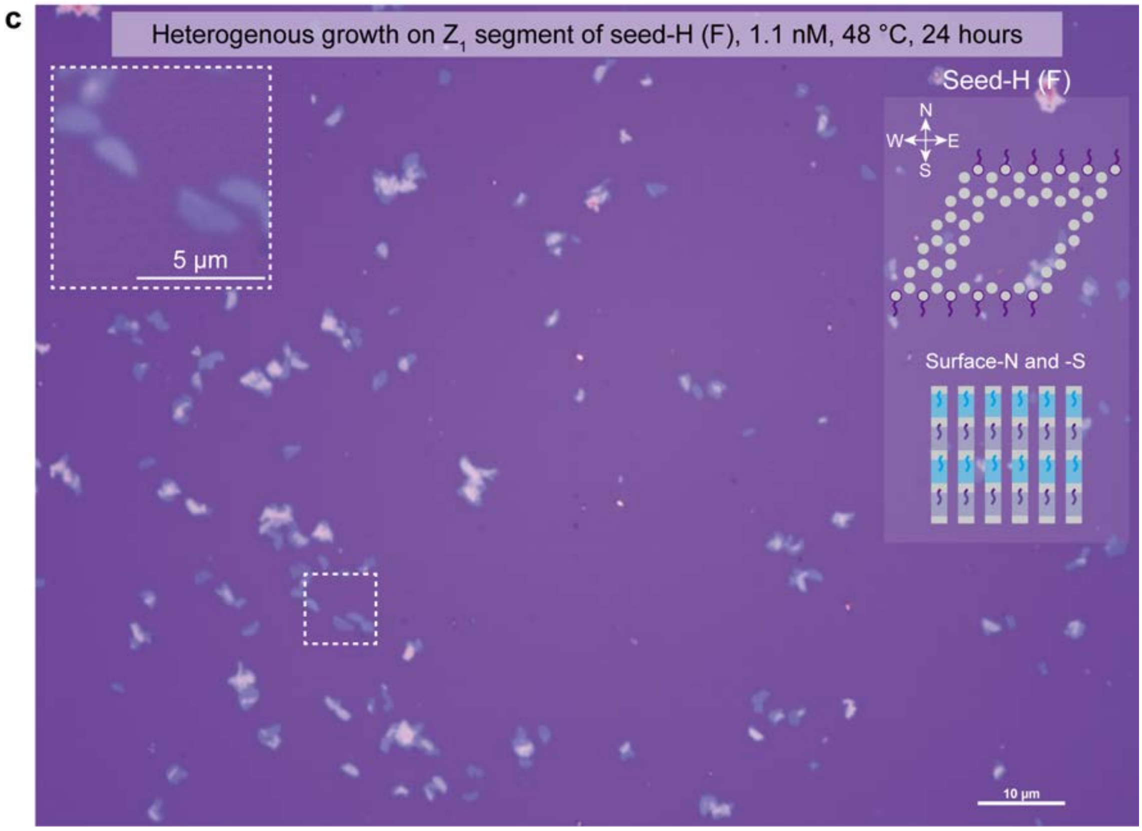

Supplementary Fig. 45 | Seeded growth of honeycomb monolayers with seed-H (N) and seed-H (F). a, Homogenous growth that forms monolayers is used as a control. **b,** With seed-H (N). **c,** With seed-H (F). Captures are extended only from the Z_1 segment of the seed. Seed concentration was 1.1 nM with an incubation temperature of 48°C for 24 hours.

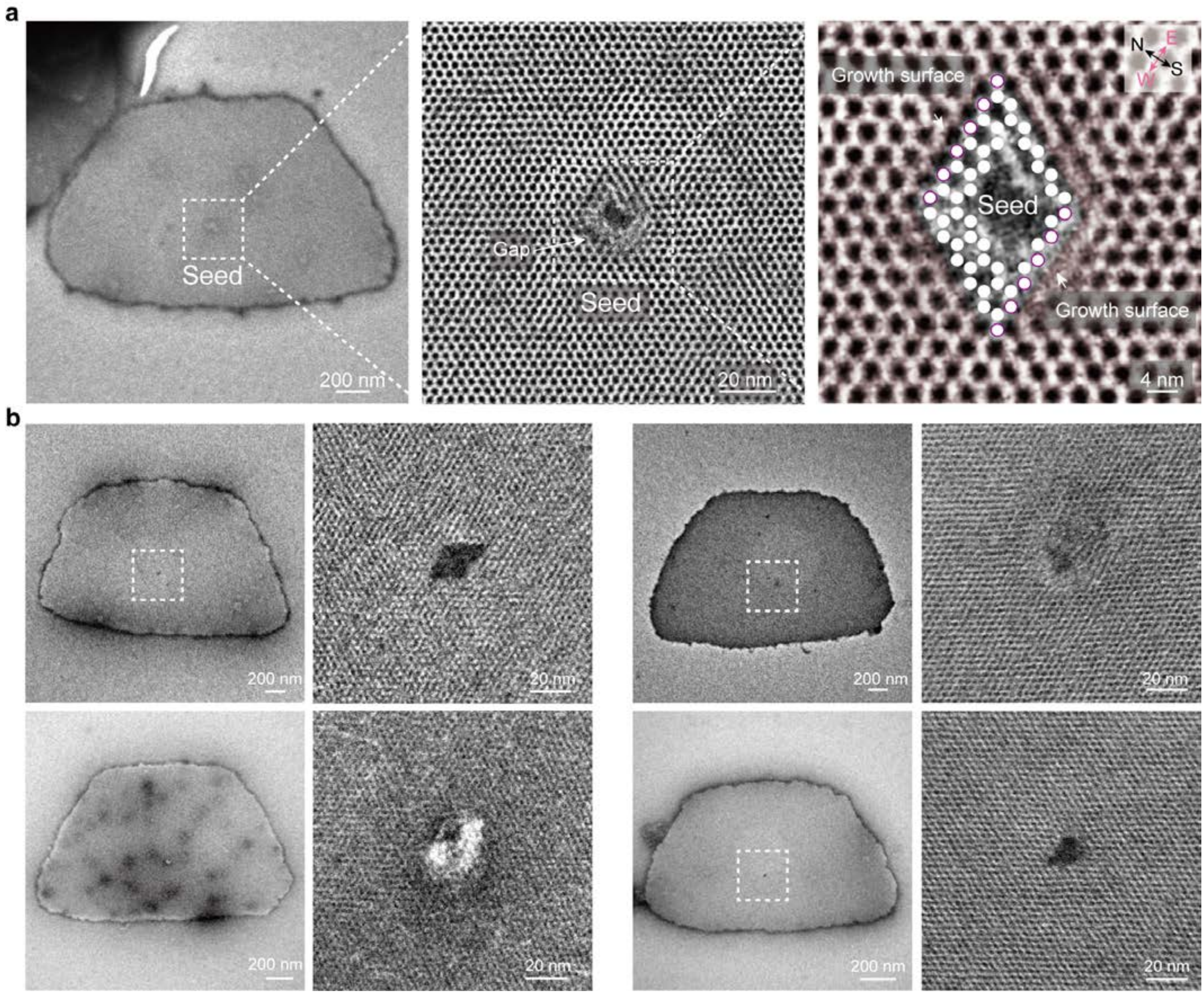

Supplementary Fig. 46 | Seeded growth of honeycomb monolayers with seed-H (F) at a seed concentration of 1.1 nM with incubation at 48°C for 24 hours. a, Hollow seed (12-helix×6-helix rim with 6-helix × 3-helix pore) is clearly visible. Growth surfaces of the seed and the gaps between the seed and sublattice on capture-free surfaces are marked by white arrows. **b,** Additional TEM images.

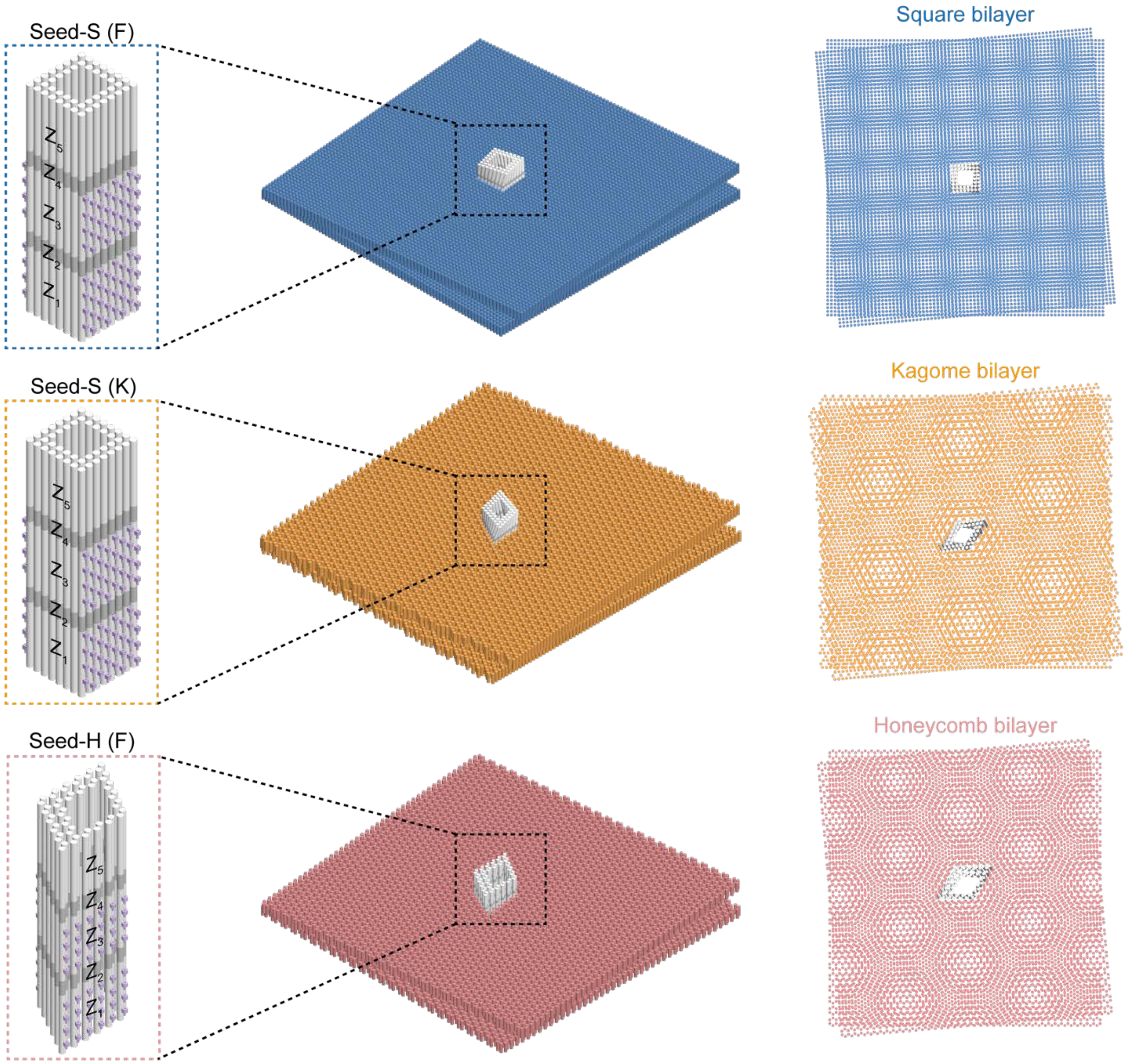

Supplementary Fig. 47 | Schematics of seeded square, kagome and honeycomb bilayers.

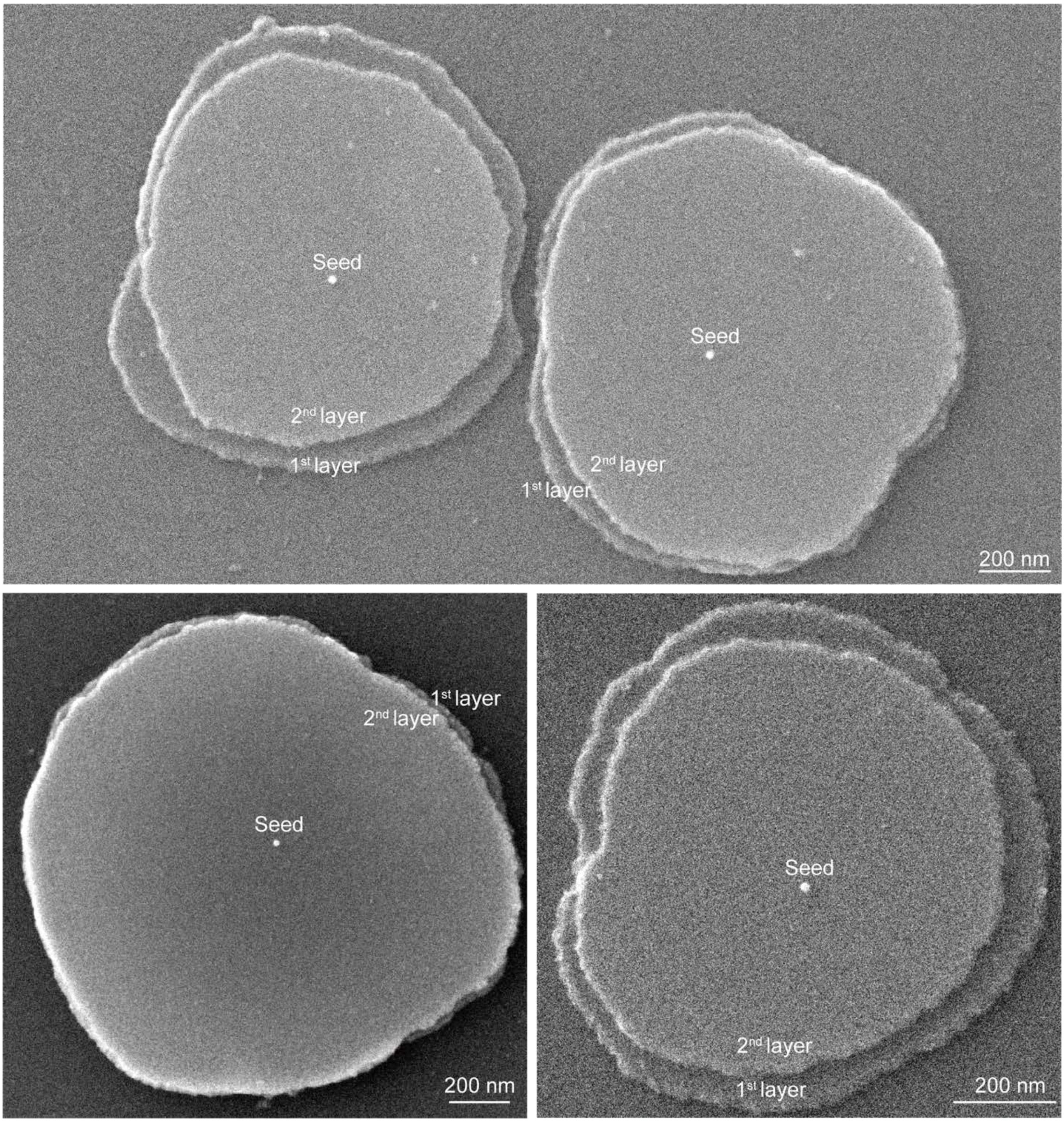

Supplementary Fig. 48 | SEM images of seeded square bilayers with seed-S (F). Captures are extended from the Z_1 and Z_3 segments.

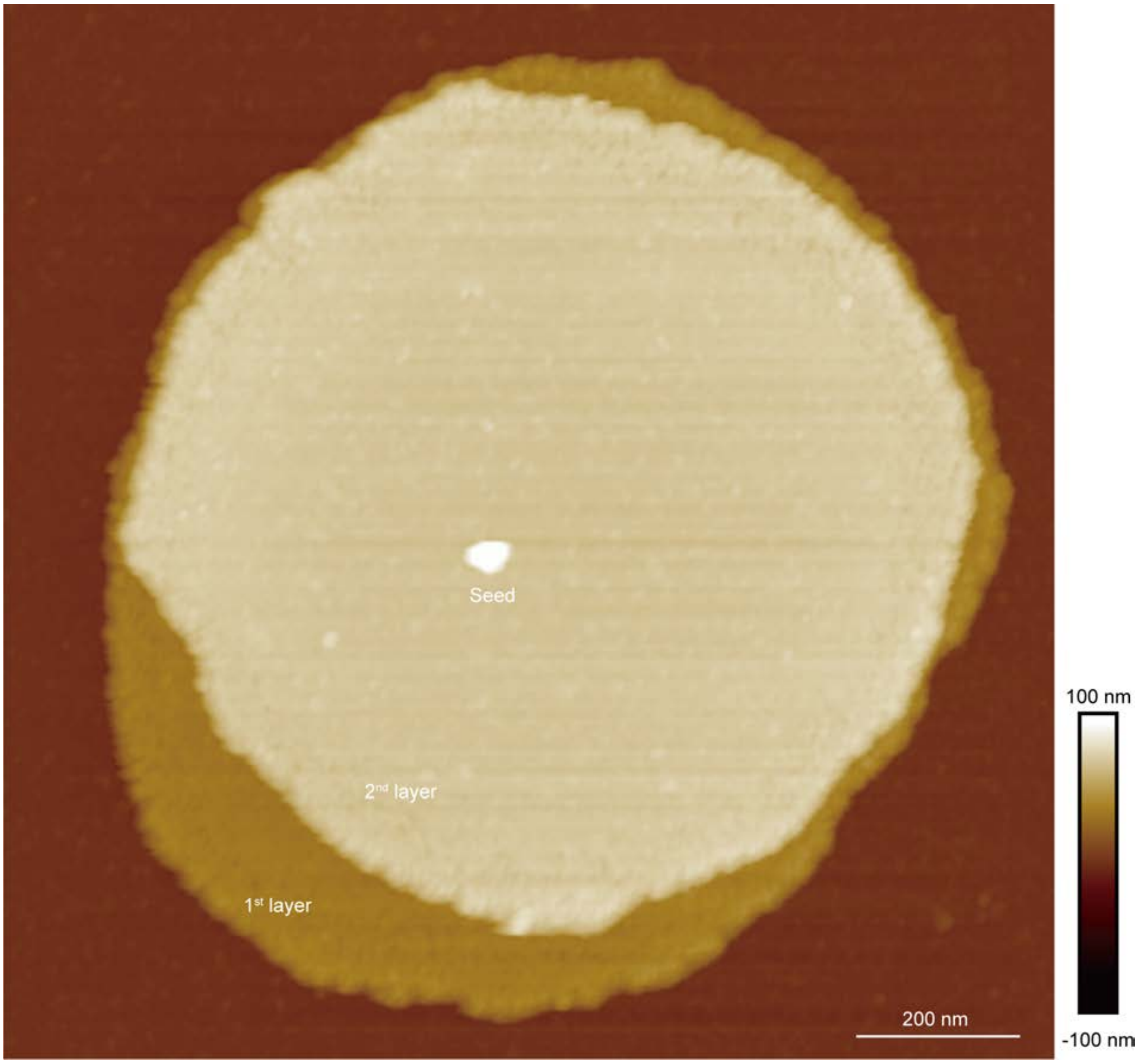

Supplementary Fig. 49 | AFM images of seeded square bilayers with seed-S (F). Captures are extended from the Z_1 and Z_3 segments. The height profile is shown in **Fig. 3d**.

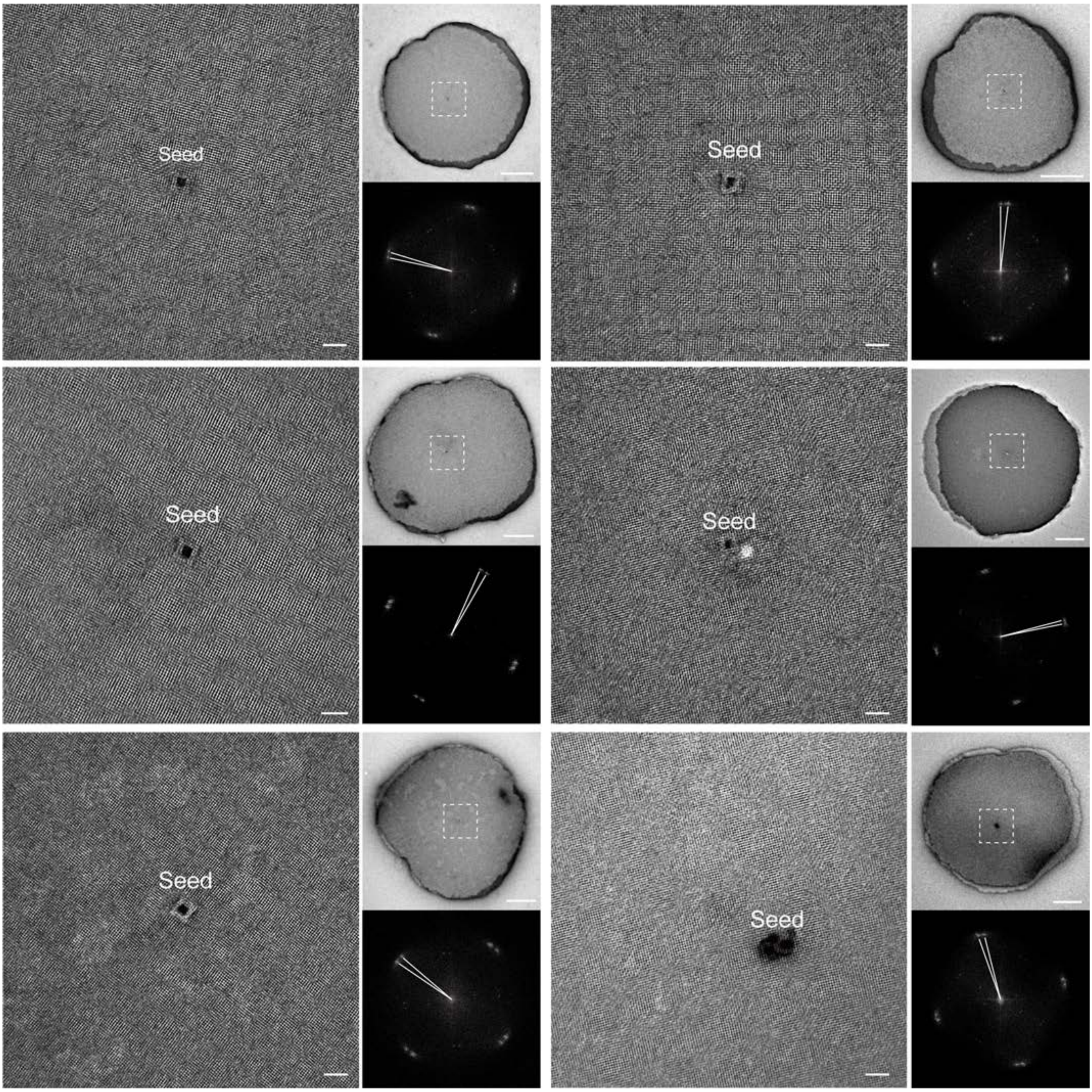

Supplementary Fig. 50 | TEM images of seeded square bilayers with seed-S (F). Captures are extended from the Z_1 and Z_3 segments. Scale bars: high-magnification images, 20 nm; low-magnification images, 200 nm.

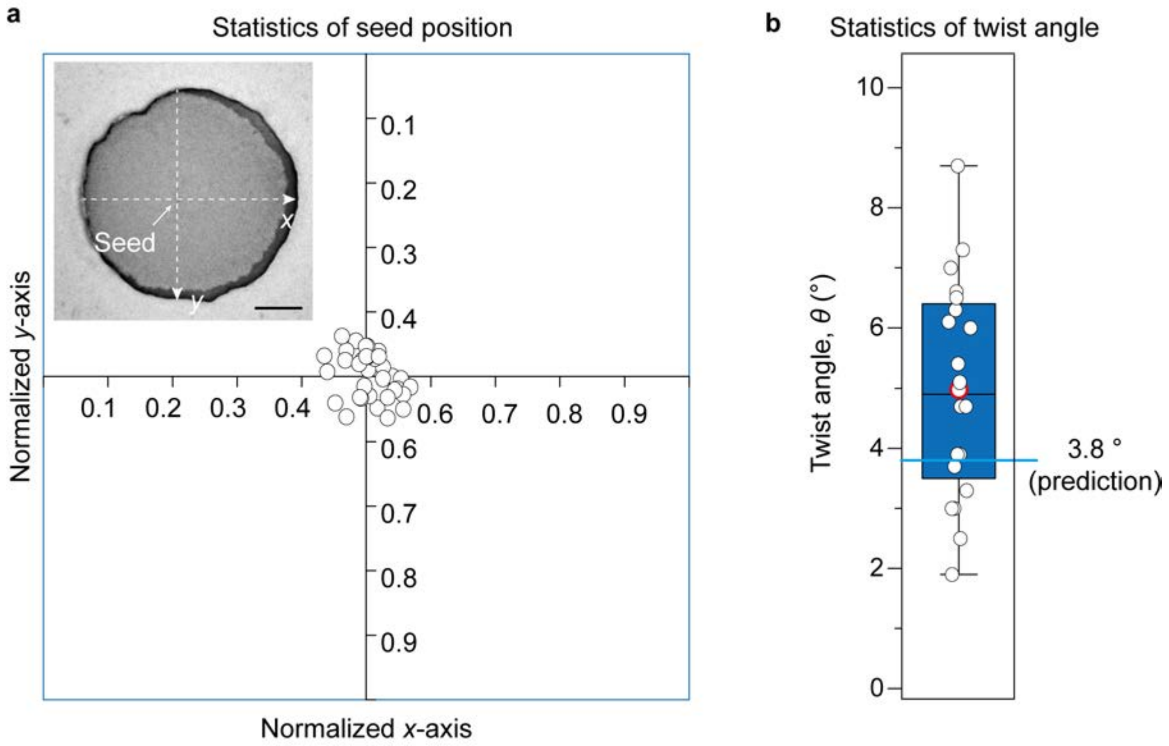

Supplementary Fig. 51 | Seed position and twist angle statistics for seeded square bilayers. a, Seed position statistics yield an average position at (0.5, 0.5), indicating a central seed location. $N = 36$. Scale bar, 200 nm. **b,** Twist angle (θ) statistics yield an average value of 5.0°, with the predicted angle (3.8°) highlighted. $N = 20$. For the boxplot, the central line is the median, the minima and maxima of the box extends to the 25th and 75th percentiles, whiskers extend to data points within 1.5 × IQR of Q1 and Q3.

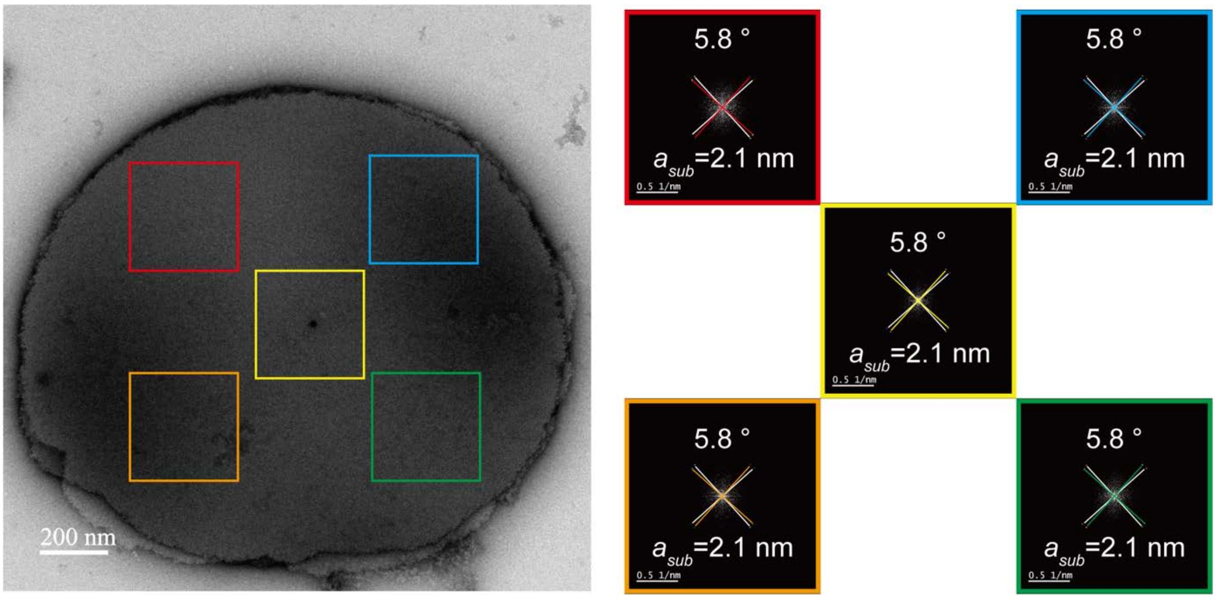

Supplementary Fig. 52 | FFT analyses of multiple regions (300 nm² each) within a seeded square bilayer. The results reveal a high degree of uniformity in both a_{sub} and θ across the sample.

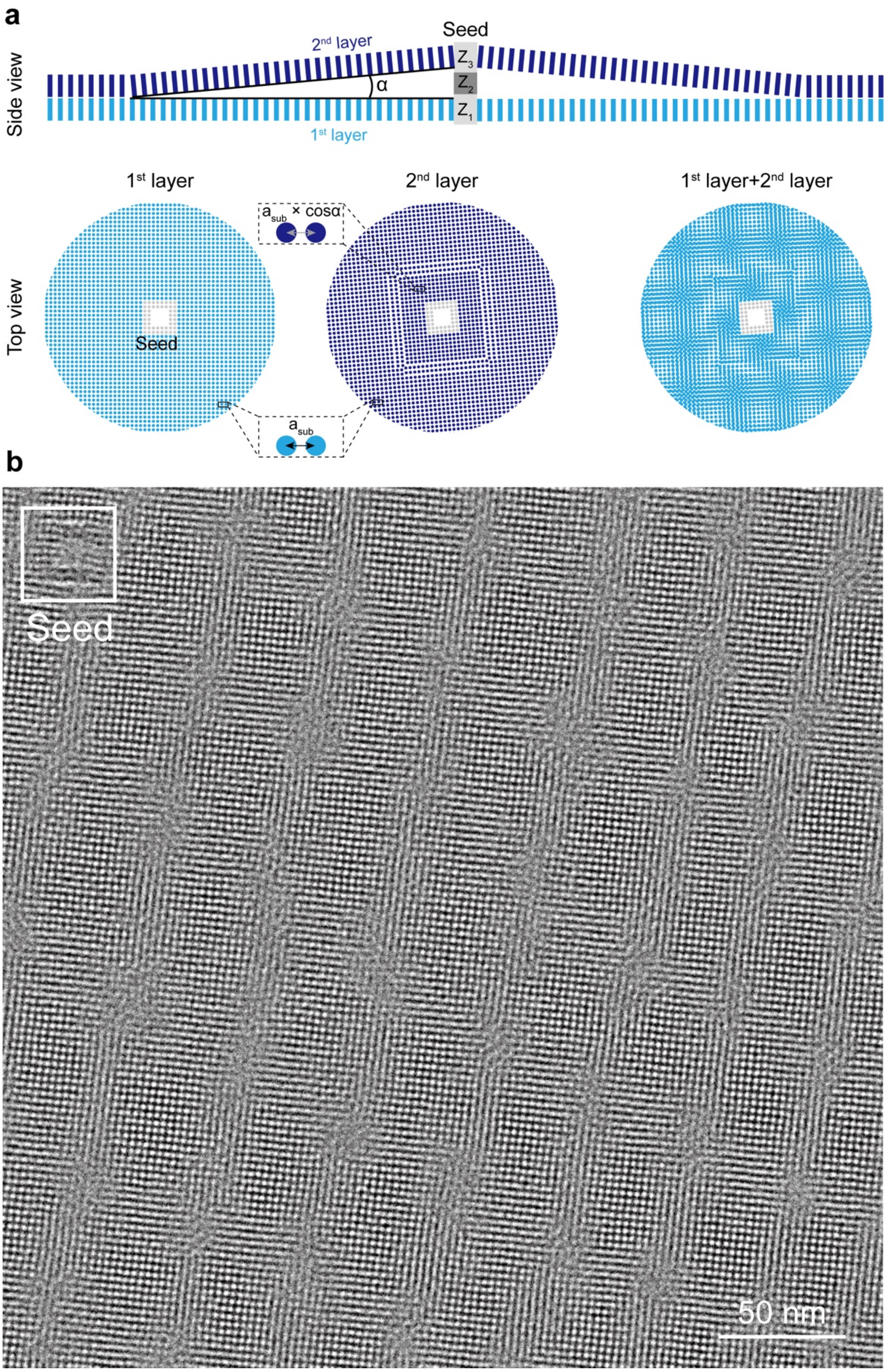

Supplementary Fig. 53 | a, Schematic illustration showing how subtle vertical distortions can lead to observable variations in the resulting moiré pattern. **b,** Cryo-EM image of a seeded square bilayer, demonstrating that the moiré pattern extends uniformly from the seed over several hundred nanometers without noticeable distortion.

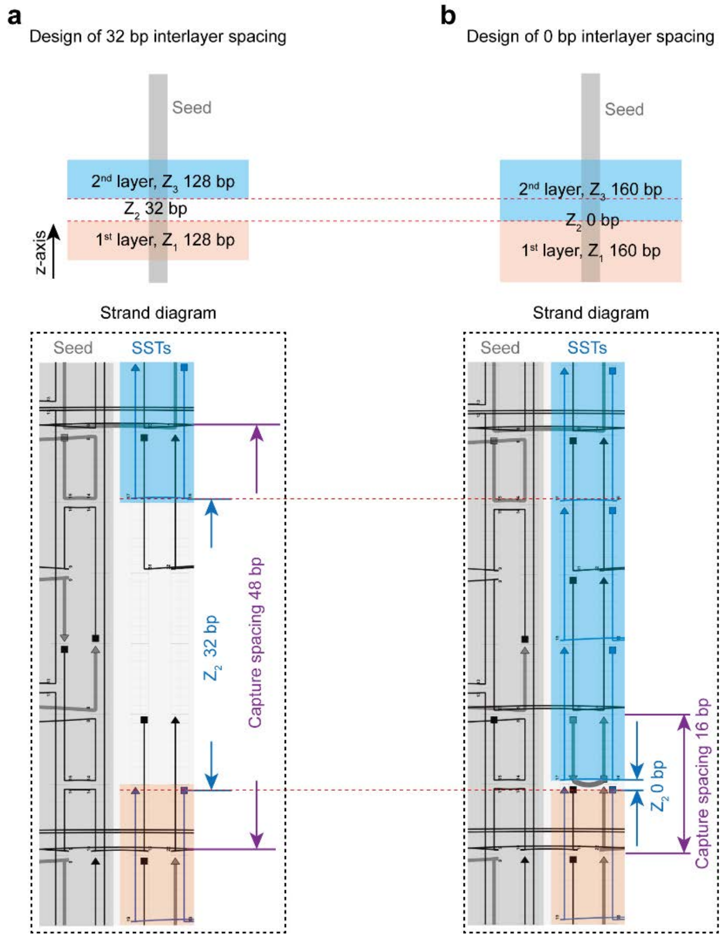

Supplementary Fig. 54 | Tunable interlayer spacing. a, Design of 32 bp interlayer spacing. **b,** Design of 0 bp interlayer spacing.

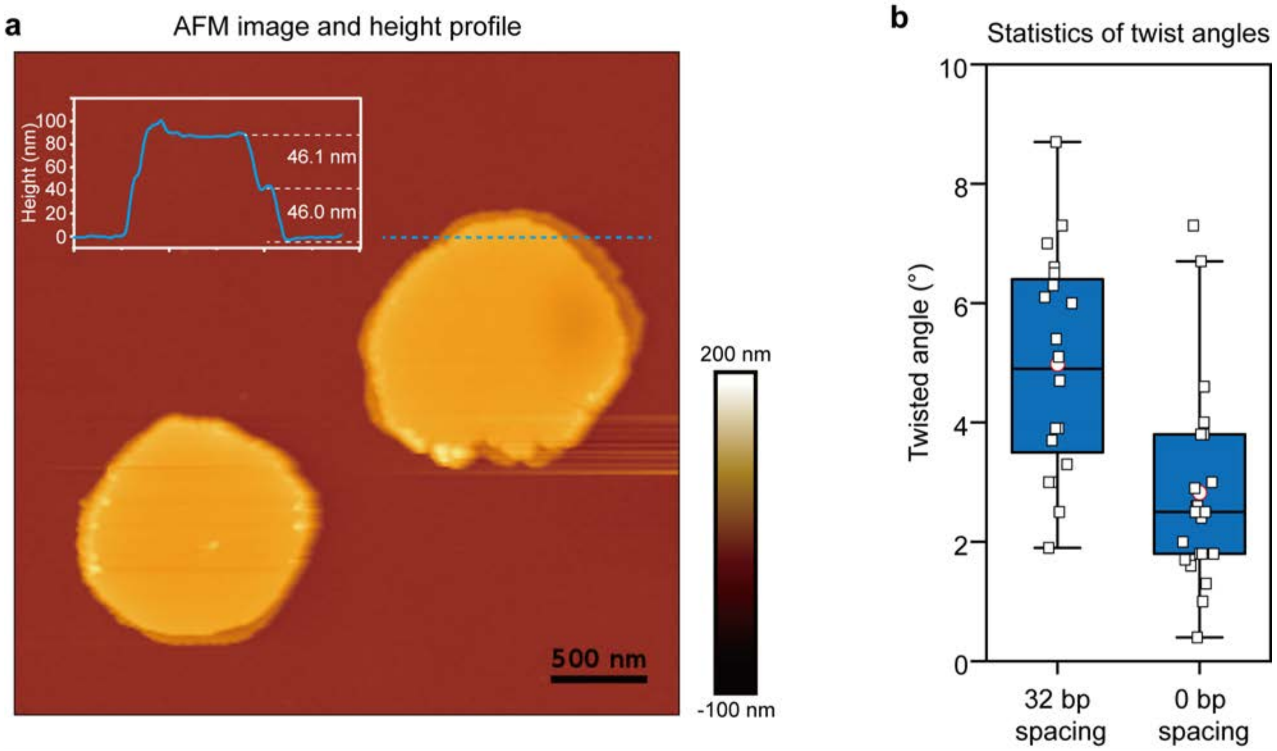

Supplementary Fig. 55 | a, AFM image and height profile of seeded square bilayers with $Z_2 = 0$ bp. **b,** twist angle distributions for $Z_2 = 32$ bp and $Z_2 = 0$ bp. $N = 20$ (32 bp) and 22 (0 bp). For $Z_2 = 0$ bp, although the sublattices are in contact, the 16 bp capture spacing on the origami seed, unanchored by SSTs, introduces a residual twist between the layers. The theoretical twist introduced by this 16 bp segment is approximately 1.9°, which accounts for the nonzero twist for $Z_2 = 0$ bp. This reasoning also explains why the experimentally observed average twist angle for $Z_2 = 32$ bp ($\theta = 5.0° \pm 1.8°$) is larger than the predicted value (3.8°). In this context, the 16 bp capture spacing can be regarded as a baseline offset of 1.9°. Subtracting this offset results in a further improved agreement between the experimental data and theoretical predictions. For the boxplots, the central line is the median, the minima and maxima of the box extends to the 25th and 75th percentiles, whiskers extend to data points within 1.5 × IQR of Q1 and Q3.

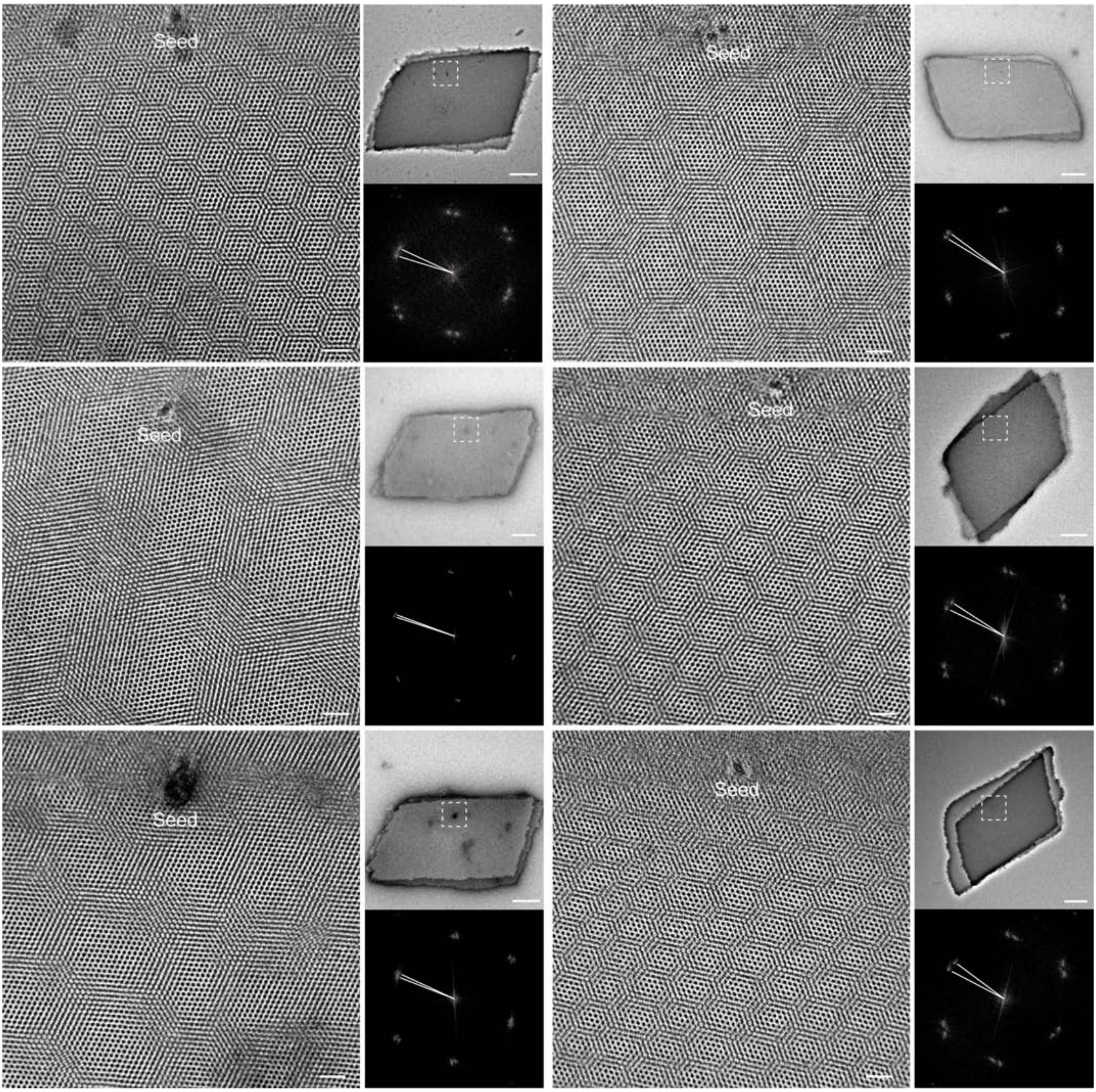

Supplementary Fig. 56 | TEM images of seeded kagome bilayers with seed-S (K). Captures are extended from the Z_1 and Z_3 segments. Scale bars: high-magnification images, 20 nm; low-magnification images, 200 nm.

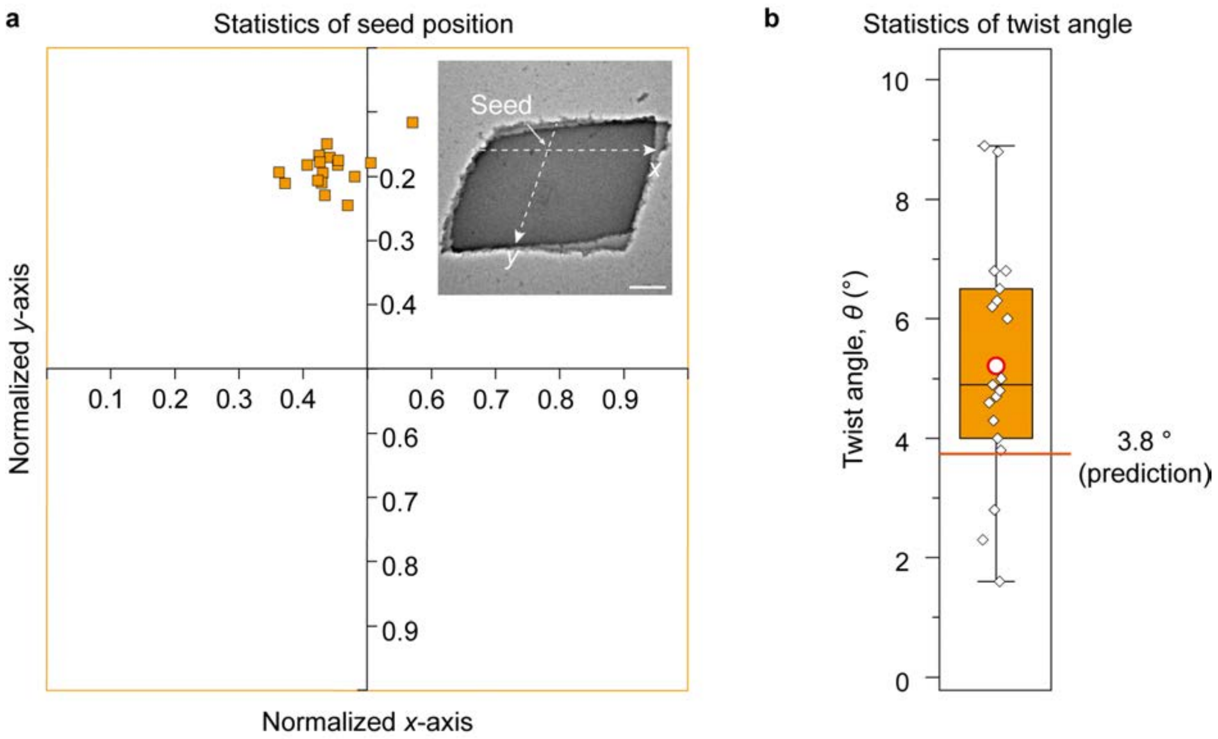

Supplementary Fig. 57 | Seed position and twist angle statistics for seeded kagome bilayers. a, Seed position statistics yield an average position at (0.44, 0.19). $N = 17$. Scale bar, 200 nm. The off-center feature of seed-S (K) likely arises from different capture designs on surface-north (non-cooperative) and surface-south (fully-cooperative) as shown in **Supplementary Fig. 41**. Nucleation and growth of SSTs favor surface-south, causing asymmetry. **b,** Twist angle (θ) statistics yield an average value of 5.2°, with the predicted angle (3.8°) highlighted. $N = 19$. For the boxplot, the central line is the median, the minima and maxima of the box extends to the 25th and 75th percentiles, whiskers extend to data points within 1.5 × IQR of Q1 and Q3.

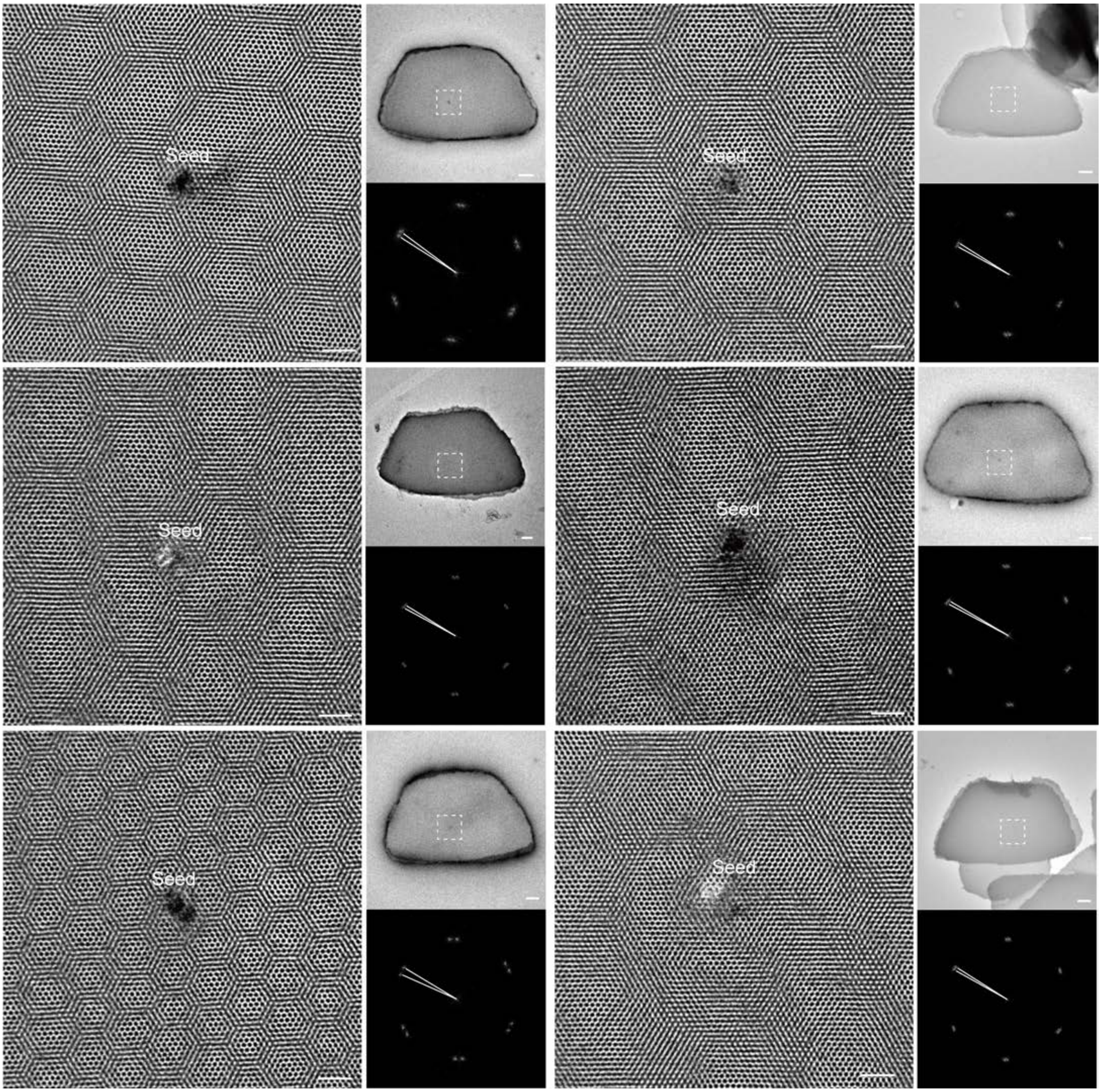

Supplementary Fig. 58 | TEM images of seeded honeycomb bilayers with seed-H (F). Captures are extended from the Z_1 and Z_3 segments. Scale bars: high-magnification images, 20 nm; low-magnification images, 200 nm.

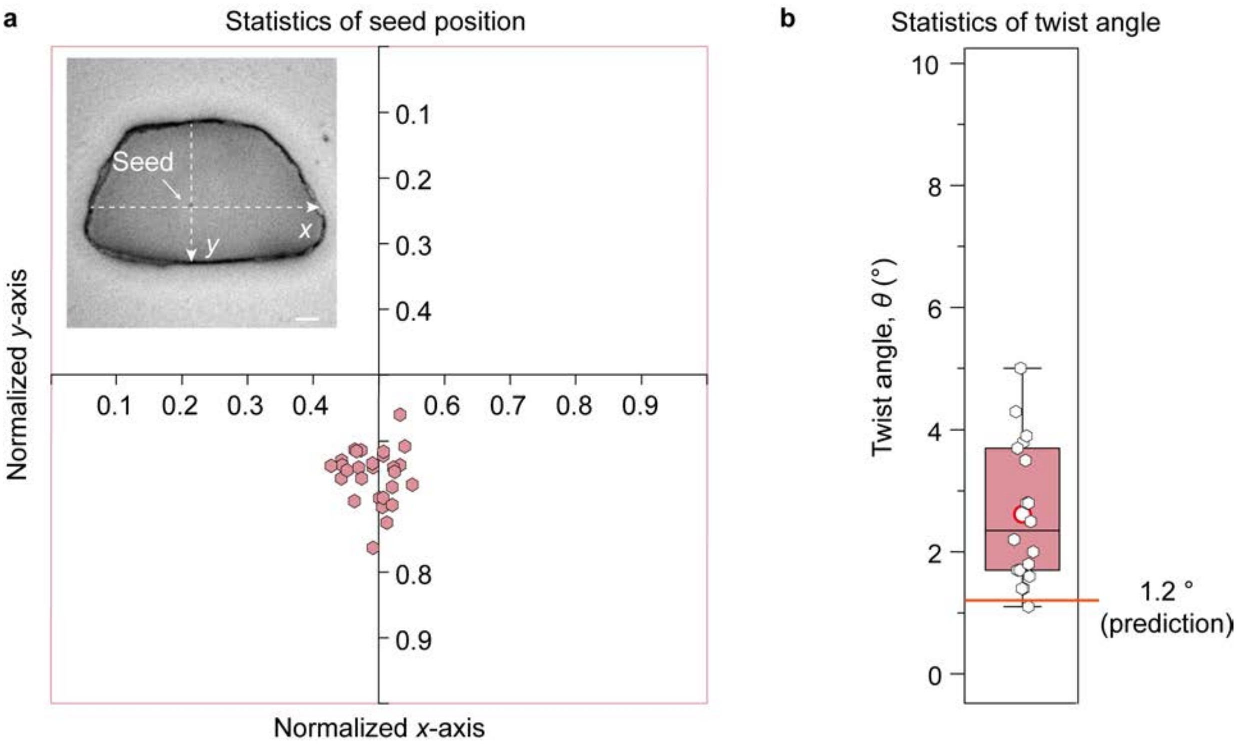

Supplementary Fig. 59 | Seed position and twist angle statistics for seeded honeycomb bilayers. a, Seed position statistics yield an average position at (0.49, 0.65), with a shift in the + *y* direction due to the inherent asymmetry of the sublattice, not the seed design. *N* = 28. Scale bar, 200 nm. The same asymmetric characteristics appear in the unseeded honeycomb sublattice, as shown in **Supplementary Fig. 22. b,** Twist angle (θ) statistics yield an average value of 2.6°, with the predicted angle (1.2°) highlighted. *N* = 18. For the boxplot, the central line is the median, the minima and maxima of the box extends to the 25th and 75th percentiles, whiskers extend to data points within 1.5 × IQR of Q1 and Q3.

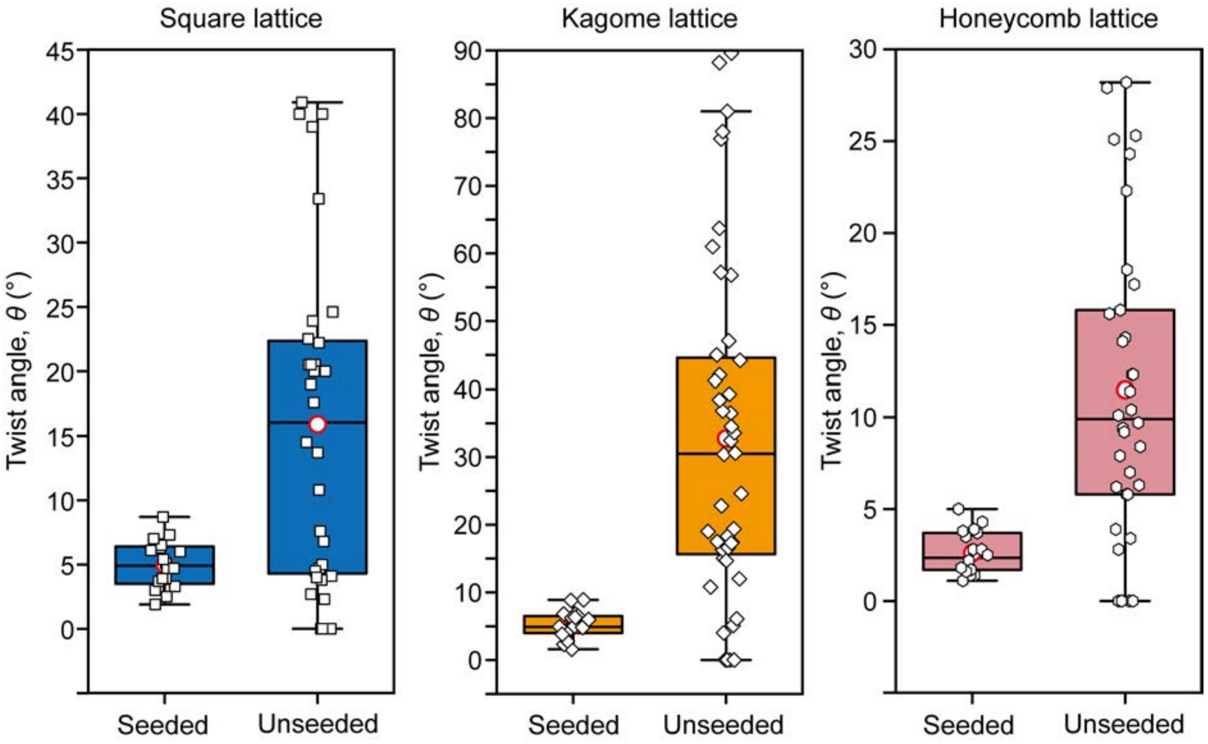

Supplementary Fig. 60 | Twist angle distributions under heterogeneous (seeded) and homogeneous (unseeded) growth. Seeded bilayer growth results in narrow, well-defined twist angle distributions across different lattice symmetries, whereas bilayers formed via random monolayer overlap in unseeded growth exhibit broad, uncontrollable angle distributions. Note that due to varying rotational symmetries, twist angles are analyzed within the following ranges: 0°-45° for square, 0°-90° for slanted kagome, and 0°-30° for honeycomb lattices in homogeneous growth. From left to right, N = 20, 32, 19, 44, 18, 34, respectively. For the boxplots, the central line is the median, the minima and maxima of the box extends to the 25th and 75th percentiles, whiskers extend to data points within 1.5 × IQR of Q1 and Q3.

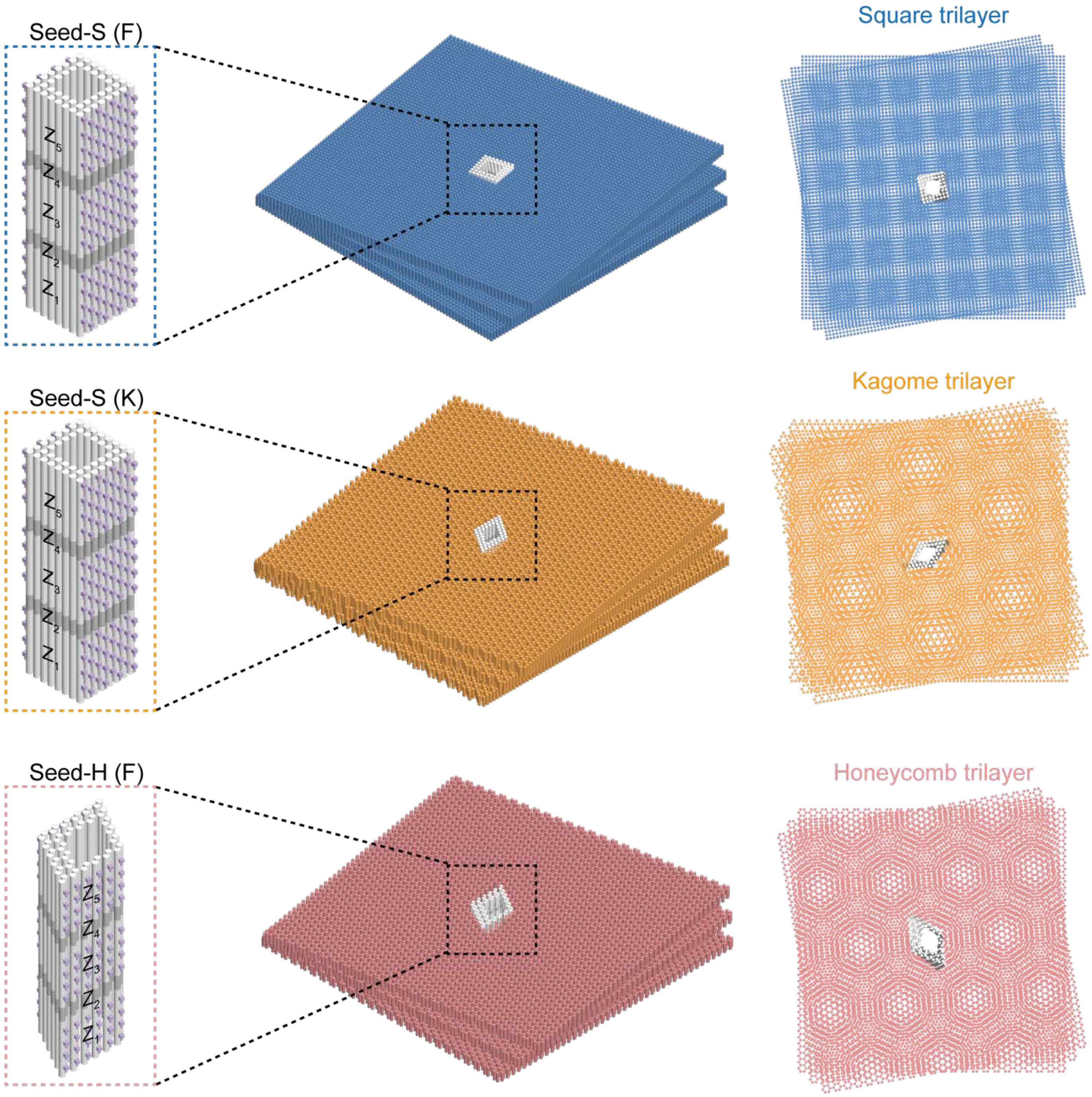

Supplementary Fig. 61 | Schematics of seeded square, kagome and honeycomb trilayers with seed-S (F), seed-S (K), seed-H (F), respectively. Captures are extended from the Z_1, Z_3 and Z_5 segments.

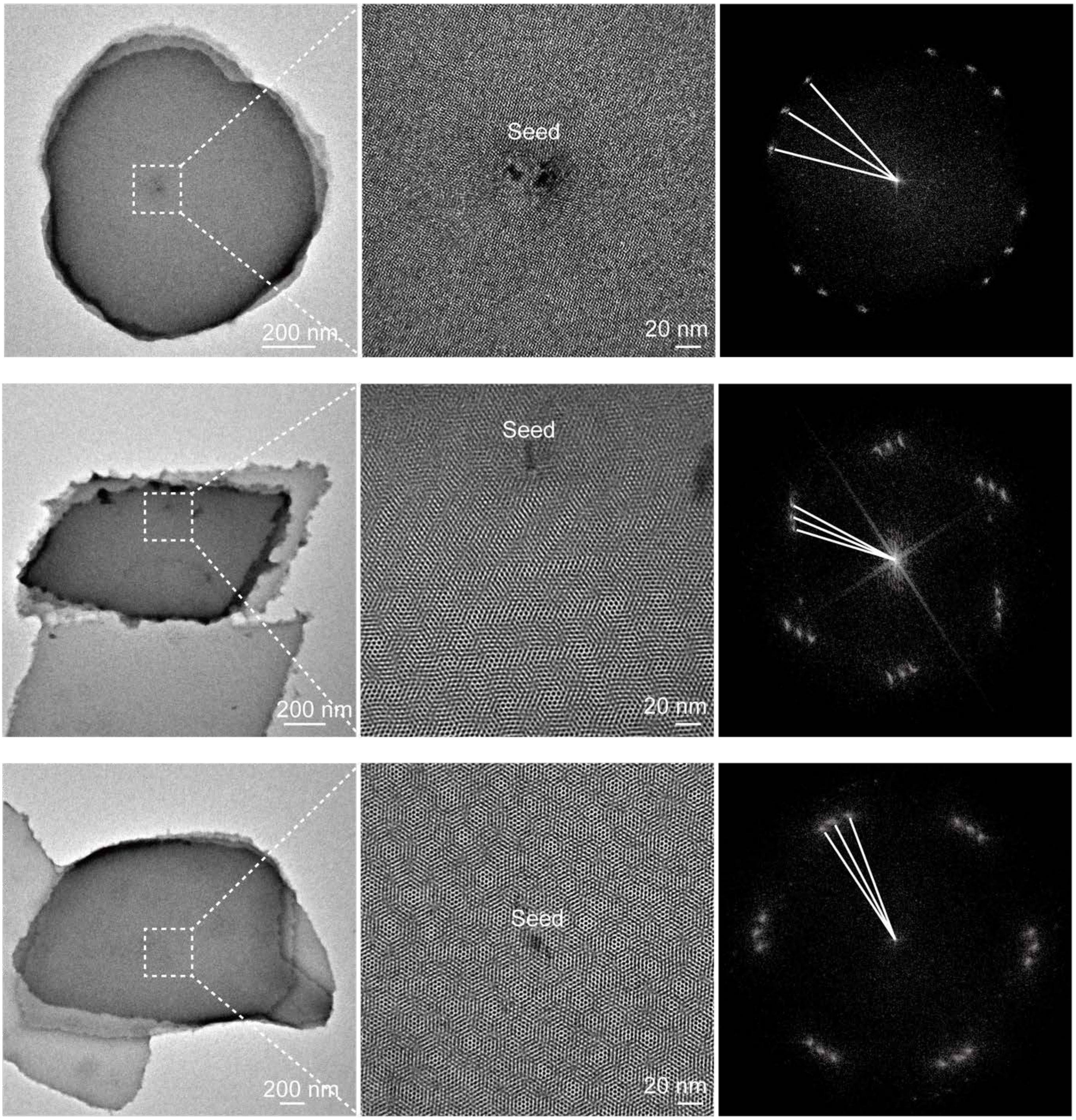

Supplementary Fig. 62 | TEM images of seeded square, kagome and honeycomb trilayers.

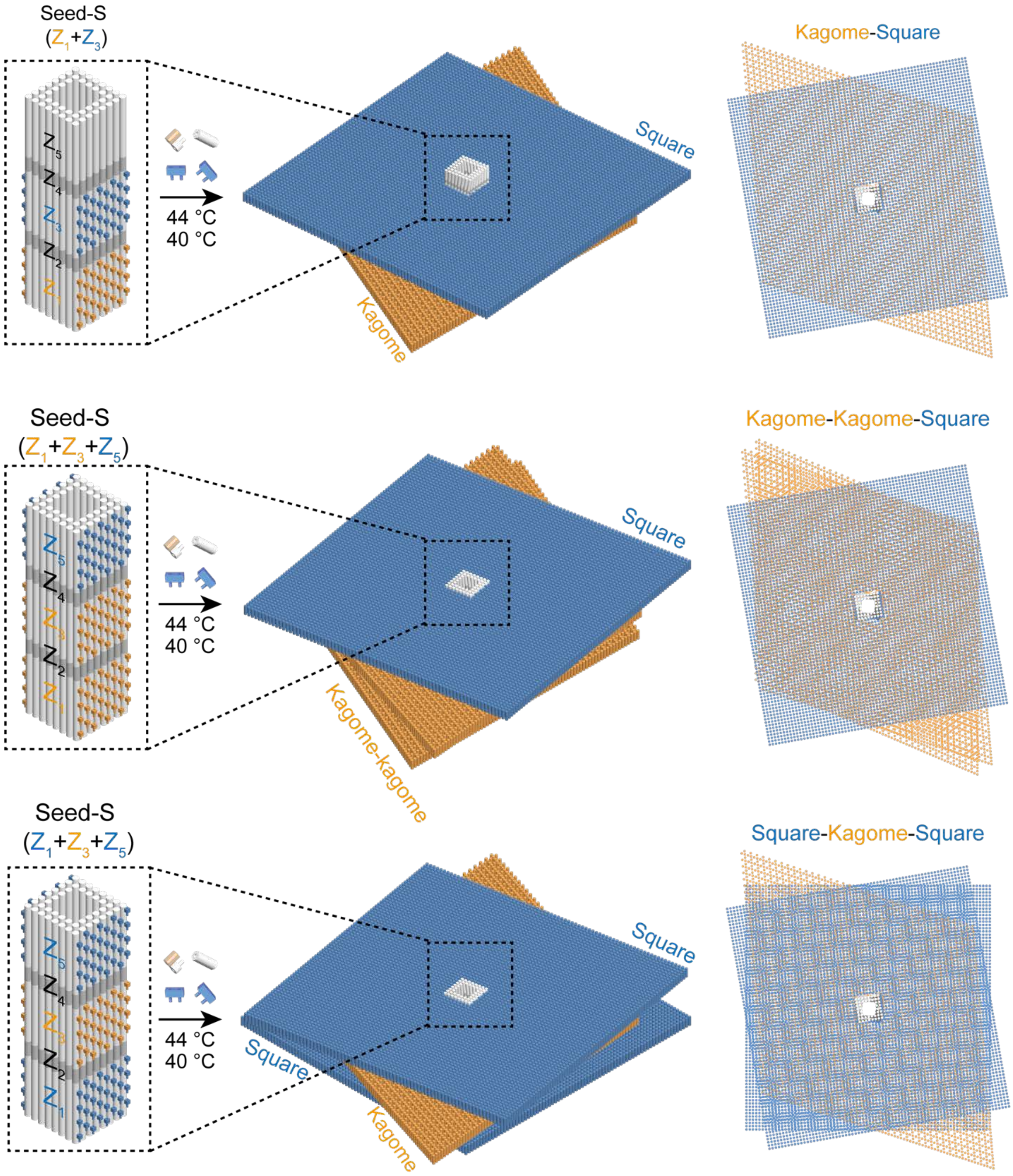

Supplementary Fig. 63 | Schematics of seeded bilayers and trilayers with seed-S, consisting of different sublattices. Captures are extended from the Z_1, Z_3 and Z_5 segments. Kagome and square SSTs were mixed with seed-S at 46°C, followed by incubation at 44 °C for kagome sublattice growth and 40 °C for square sublattice growth, respectively.

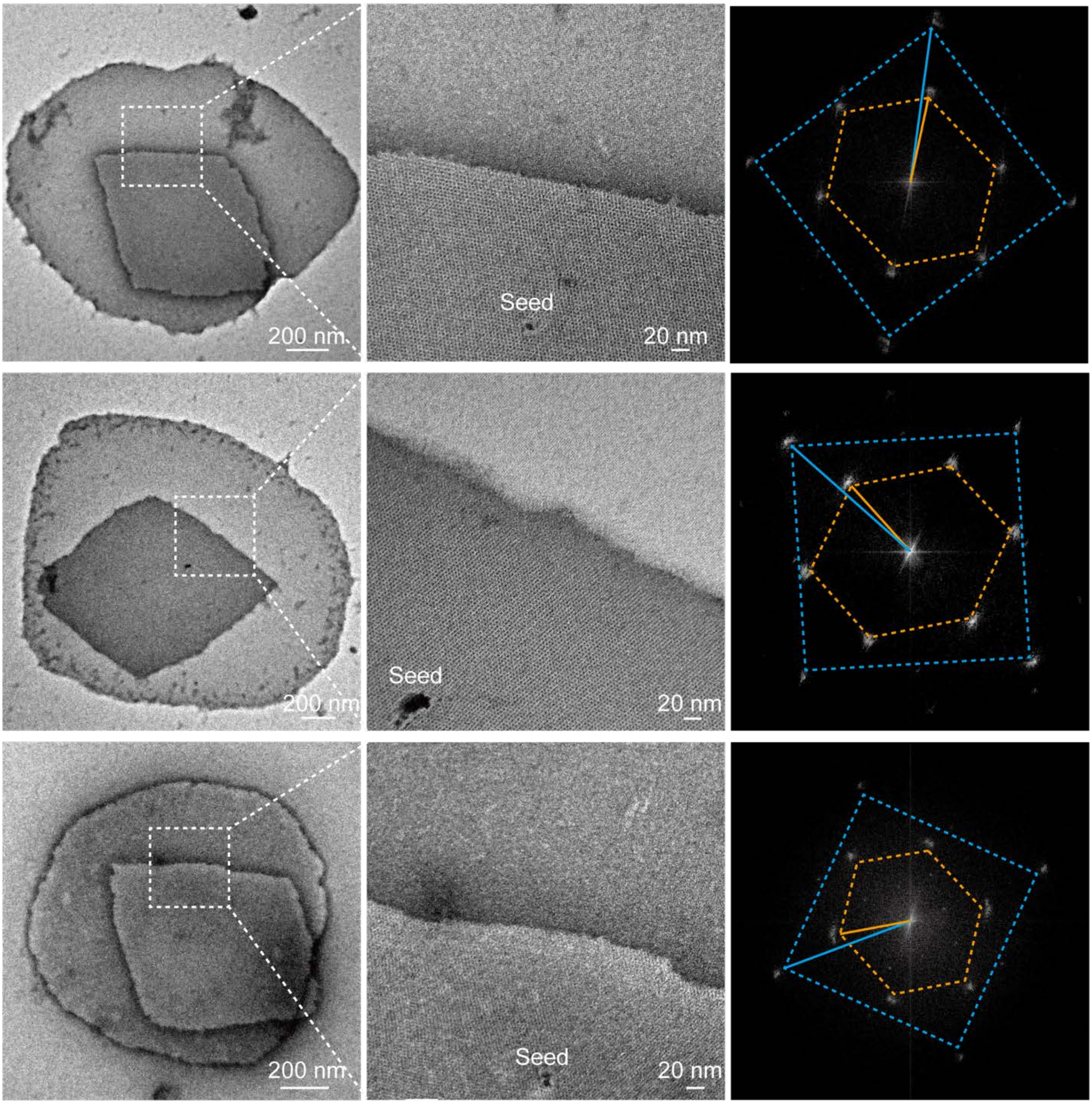

Supplementary Fig. 64 | TEM images of seeded kagome-square bilayers with seed-S. Z_1 templates the growth of the kagome sublattice, while Z_3 directs the growth of the square sublattice. FFT patterns demonstrate the twist feature between the stacked sublattices.

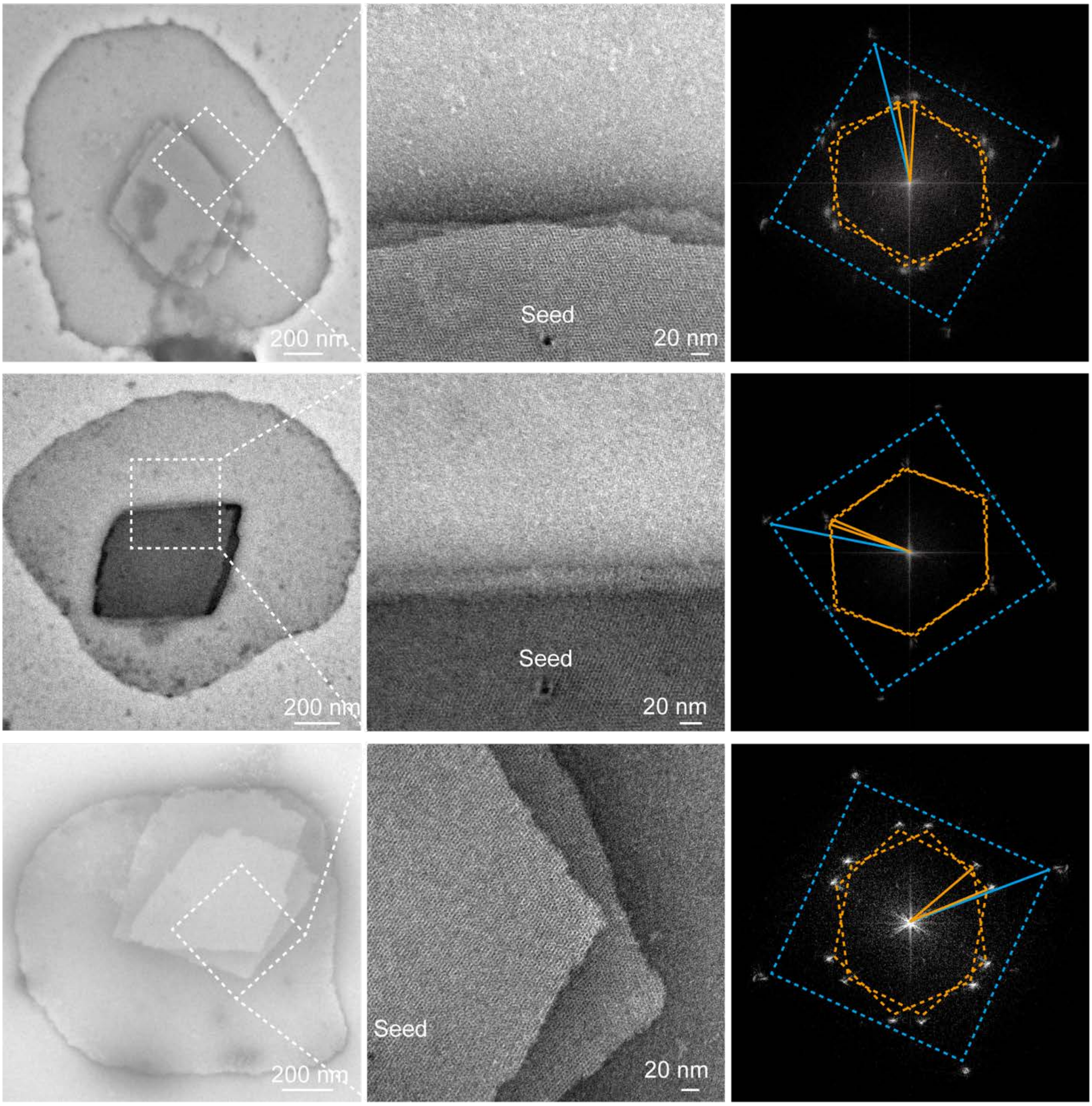

Supplementary Fig. 65 | TEM images of seeded kagome-kagome-square trilayers with seed-S. Z_1 and Z_3 template the kagome sublattices, while Z_5 directs the square sublattice. FFT patterns demonstrate the twist feature between the stacked sublattices.

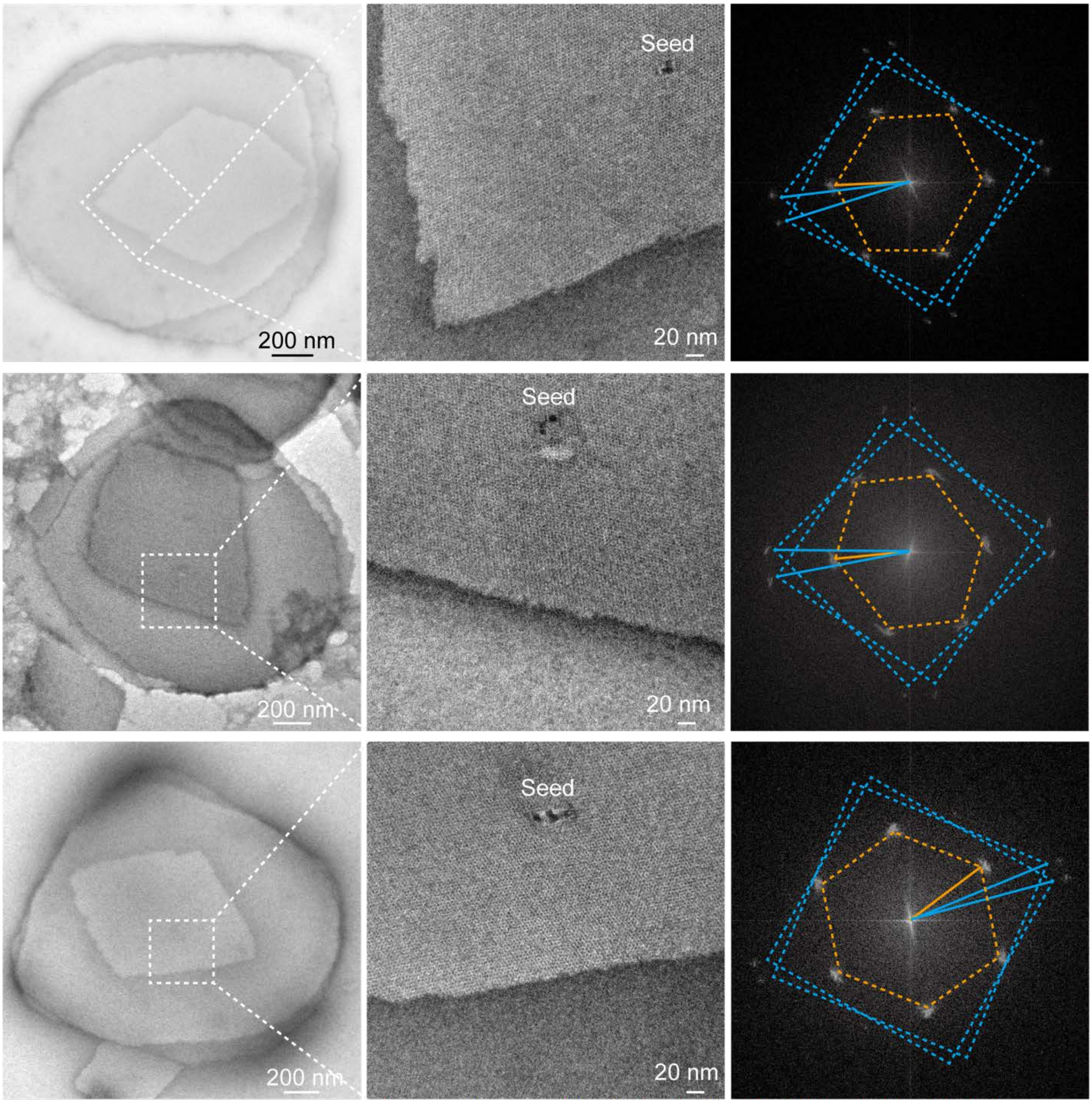

Supplementary Fig. 66 | TEM images of seeded square-kagome-square trilayers with seed-S. Z_1 and Z_5 template the square sublattices, while Z_3 directs the kagome sublattice. FFT patterns demonstrate the twist feature between the stacked sublattices.

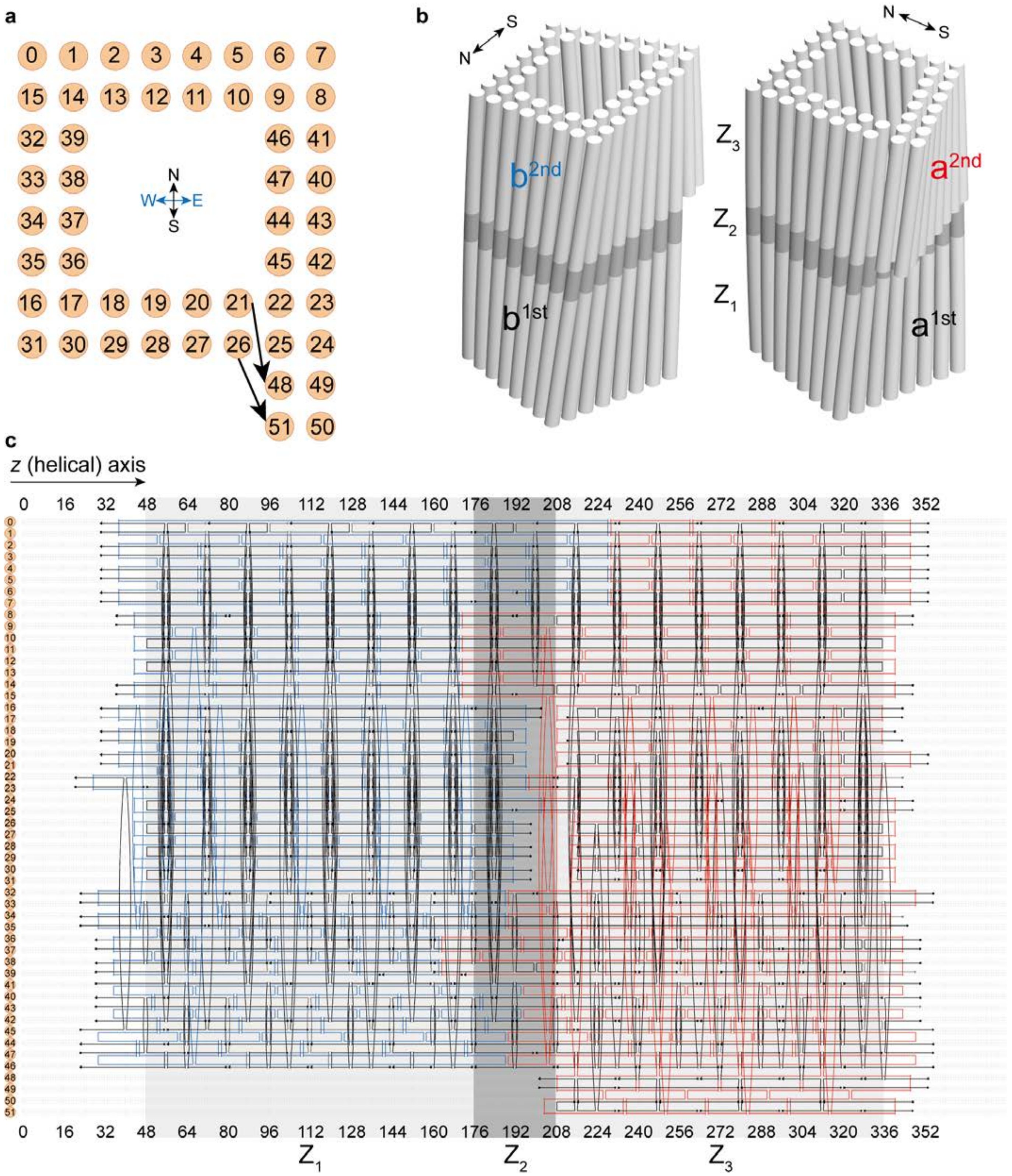

Supplementary Fig. 67 | Seed-S (G) for gradient DNA moiré superlattices. a, Cross-section view of seed-S (G) in caDNAno format. In the Z_3 segment, helix 21 is connected to helix 48 instead of helix 22, and helix 26 is connected to helix 51 instead of helix 25. **b,** Schematic of seed-S (G) (not to scale). **c,** Strand diagram in caDNAno format. The numbers on the left indicate the helices, while the numbers on the top and bottom indicate the positions of the bases along the *z*-axis. Blue and red strands represent two orthogonal scaffolds p7560 and CS3-L, respectively.

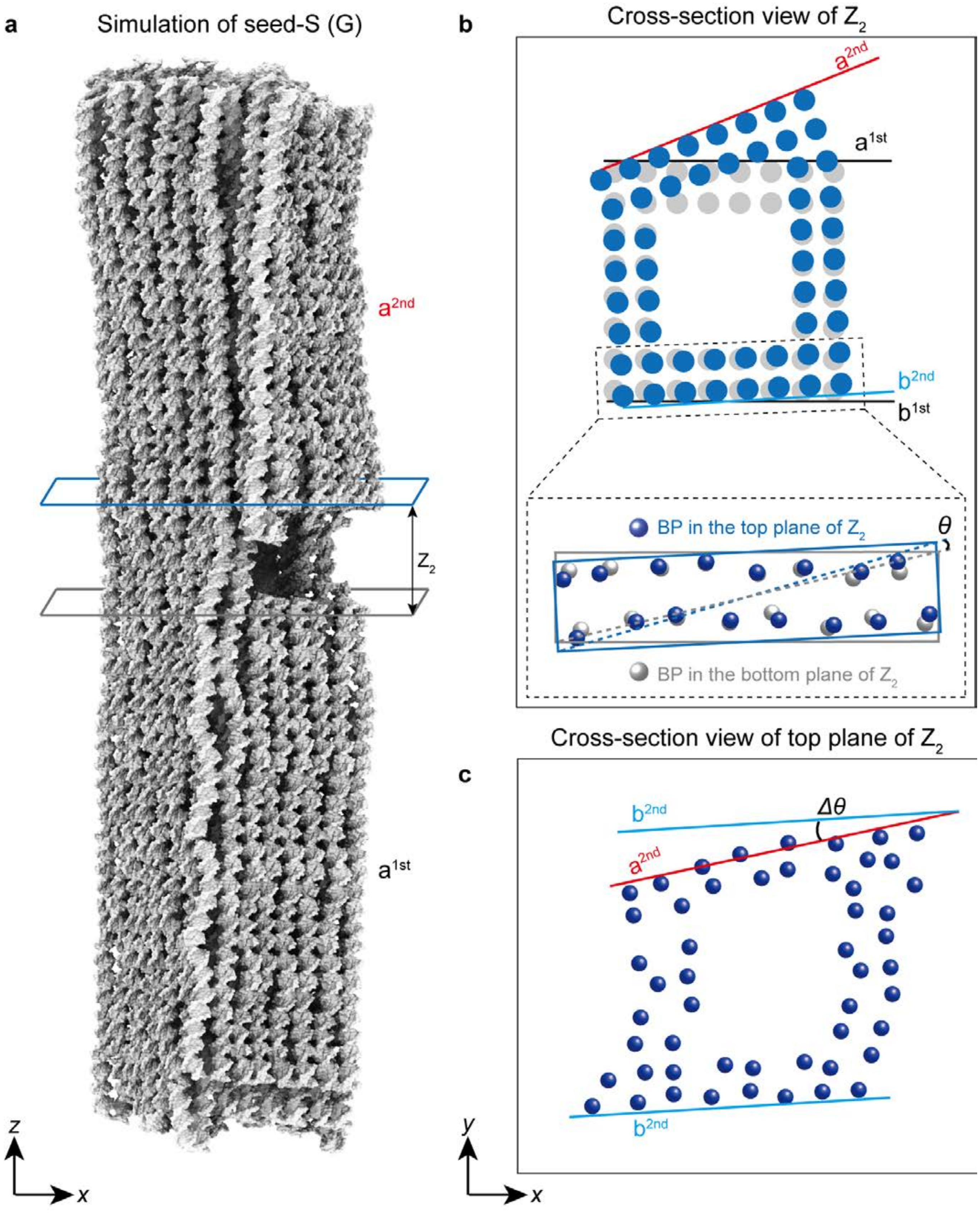

Supplementary Fig. 68 |Calculation of the twist angle of seed-S (G). a, Simulated 3D view of seed-S (G) using the SNUPI program. **b,** Cross-section view of the bottom and top planes of the Z_2 segment, also showing four SST growth surfaces (a^{1st} and b^{1st} of Z_1, a^{2nd} and b^{2nd} of Z_3). The zoom-in view shows surface-b^{1st} and surface-b^{2nd} of Z_2, extracted from the simulation. Each sphere (blue or grey) represents a BP. The twist angle (θ) was obtained by calculating the relative angle between surface-b^{1st} and surface-b^{2nd}. **c,** Cross-section view of the top plane of Z_2, extracted from the simulation. The tilting angle ($\Delta\theta$) between surface-a^{2nd} and surface-b^{2nd} was then obtained.

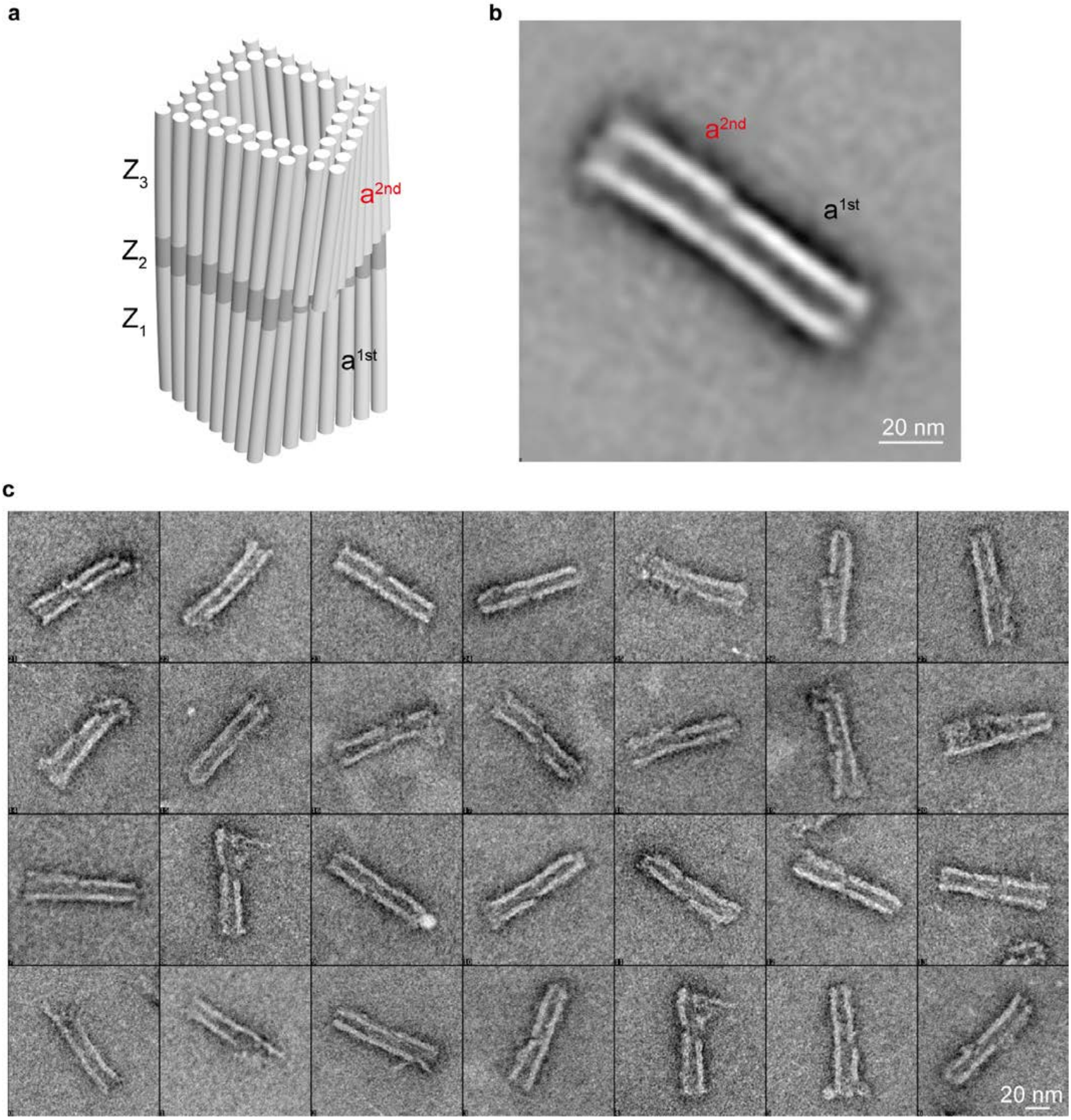

Supplementary Fig. 69 | TEM images of seed-S (G). a, Schematic and **b,** class-average TEM image of seed-S (G). **c,** Exemplary TEM images used for the class-average processing.

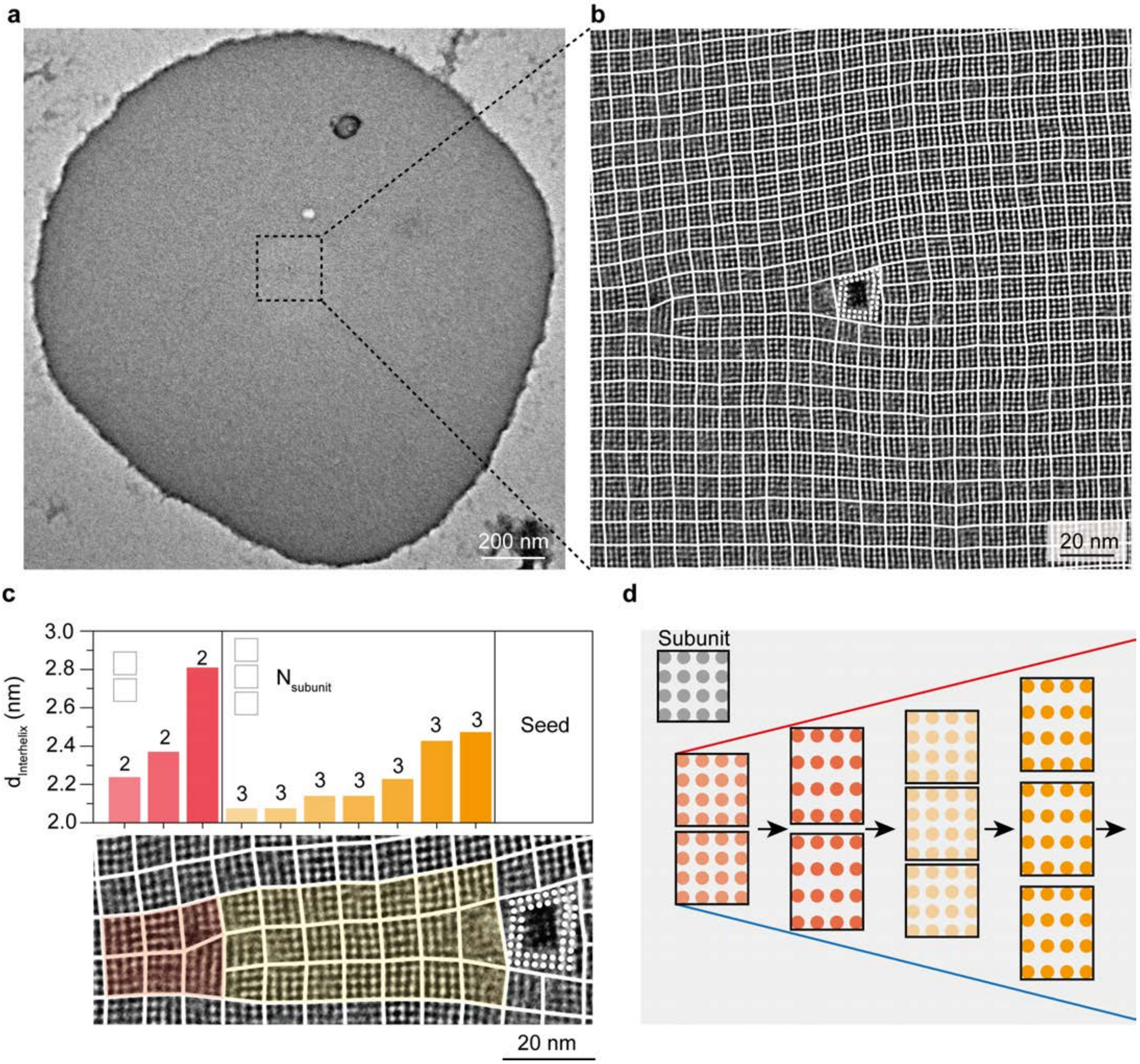

Supplementary Fig. 70 | TEM images of the non-uniform 2nd layer of seed-S (G). a, Low- and **b,** high-magnification TEM images of the 2nd layer. Each box represents an SST subunit, 4-helix × 4-helix. Seed-S (G) is marked in the image. **c,** Analysis of the interhelix distance ($d_{interhelix}$) in the left section of seed-S (G). SSTs adjust $d_{interhelix}$ to maintain an integer number of subunits and avoid structural domain mismatches, as shown in **d**.

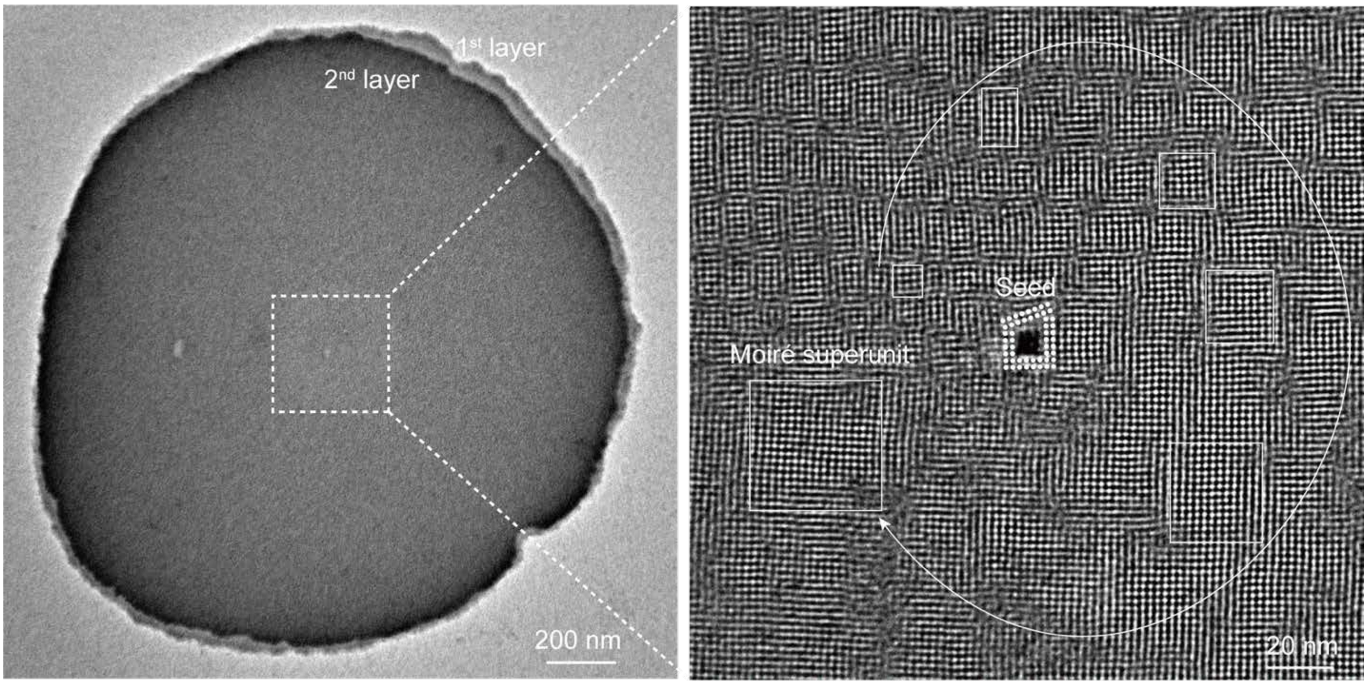

Supplementary Fig. 71 | Overview and enlarged TEM images of gradient DNA moiré superlattices in bilayers. The 1st layer, 2nd layer, seed, moiré superunit, and gradient p_M are marked in the images.

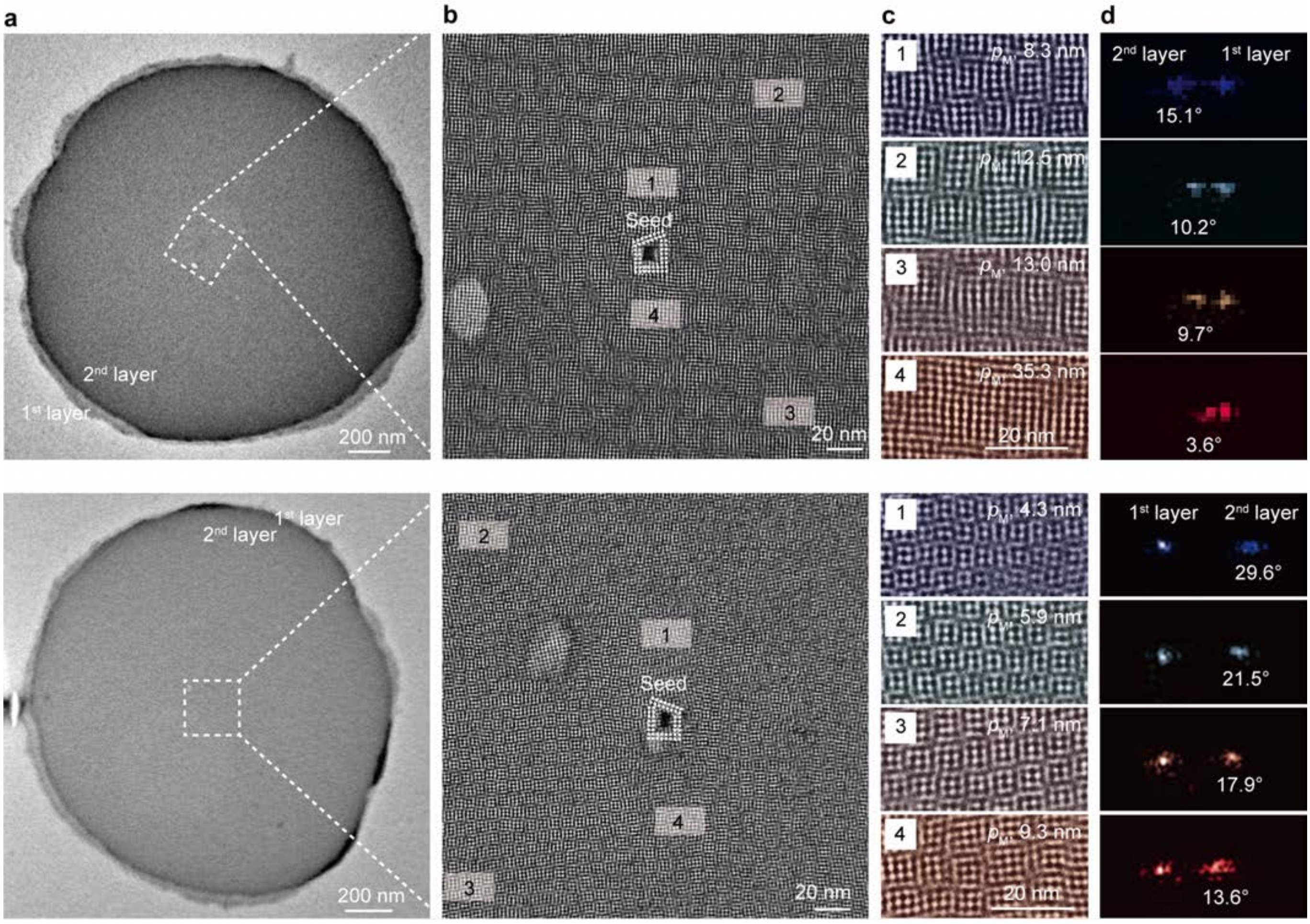

Supplementary Fig. 72 | Additional TEM images of gradient DNA moiré superlattices in bilayers. a, Overview and **b,** enlarged TEM images, with the 1st layer, 2nd layer, and seed marked. **c,** High-magnification views and **d,** FFT patterns of selected areas in **b**, displaying p_M and twist angles. Note that the bottom sample is flipped 180° relative to the top sample, causing the gradient direction to rotate counterclockwise.

Supplementary Table 1 | DNA sequences of seed-S (see Supplementary Fig. 1 for the strand diagram)

Colored sequences represent captures, 16 nt

Start	Sequences
	**Core Strands**
8[508]	TTTTTTTTTCACCCGATATAGACAGTGACATTTAATTATATTTTTTTT
34[423]	TGCCAAGGTATGTCCACAAGGAATTAAGCTAG
6[391]	GATTGCATGCACTCTTTGTCTAGCCTTGCTAGGAAGAGTGTAACCAAGAGGCCGGC
45[400]	GGTTAGTGCCATGGTTAGTCCAGTTGGCGCTCCAACAATGTGCTCTTGAATAGACA
29[192]	CGGTGTACAGAAAGTGGGTTCATTTTTTCATC
30[191]	GACAGTGTGAATTACCCAATCATAACCTATATAACACTTCTTCCAGTT
35[152]	AATCCGTTGAGTGTTGTCACCAAAATAGCGAGTAGCCGGAACGAGGCG
10[119]	TATTAATTTTAAAGAAAGATAATACATTTACC
10[439]	CCCTTTGTATTAGCCGAAAACCACATTACGAC
13[24]	TTTTTTTTGAGTTTCGTCACCAGTCATGTACCAGAACGCGAGGCGCCG
37[120]	CTGGGGTGTGAAATTGCGTTGCGCTCAGTGCT
4[167]	TAGCCGACCGTGTGATTCCTATATGTAAATGC
0[487]	AGCTGGATTGCATCCTAGGTCAACCACGTTAAGCGGGCAAGACCATAGATACTTAG
28[471]	ATTAACACTGTTTGTACTTTTATTCGGCCTTA
10[471]	GCAGACAGAGTCGTACCATGTCCTTCGTAATT
42[255]	AACTGCGACGTTGTTGCCATTCATAGTTGCCT
47[21]	TTTTTTTTCGGAACCTATTATTCTCCTTGATATAACTAATTTGCCAGTTAC
37[344]	TACAGGTCACAAGTGTGCGCAAGTAAGAGTGA
39[27]	TTTTTTTTAGGGTTTTCCCAGTCACGACGCTGTAGCAATCTAAAG
45[176]	AAACCTAACCCACGTCAATGAAGTACAAGAGGTGTAAGGTCCTACCGG
28[151]	CATCAAAAAGATTGAAGGATAGCGTCCAAGTA
28[175]	TTTAAGGATTTGAAGGAAAACGAGACAGTGTA
30[311]	GGTATCTGGTAAATTTCGGTGTAGGTCGTTCGGTATAAAATCTTACAC
44[71]	GGTCAGACATAGAATGGAAAGCGCAGTCACCG
19[344]	ACTGGAAGGCGGCATAGGCGTGAAGGCACTTG
37[27]	TTTTTTTTTGTGCTGCAAGGCGATTTCTCAGGAGAAGGTCTTGTG
32[423]	ACGGCCGGAGCAGGAAATGATGCTCGGGCCTC
47[392]	GGACTAGCACCCGGCATGCGGATCCCGCCTGAACAGAATG
4[135]	AATGAAACAATAACGGTTAAGAACCTACCATA
32[506]	TTTTTTTTCGTTTGGCACCAATAGAACAGAACTTTTTTTT
45[272]	CGCCGGCGCGTAAGAGCCAACGACCTTGATCCTTTGATCTGGGTTTGTTTGCAAGC
45[432]	CCTGAGCTAATTTGACTGCATTTTATGCTATCTCACTCGGTTGGCTATCGGCGCCC
4[103]	GTCTGCTGAACCTCAATTCGAACGAACCACCA
4[295]	TAAGGCCTATGATTCTAGGACTTGACACACAG
8[55]	AGCGTCTTAGAACCGCACCAGTTGCTATTTTGCACCCAGCTACAATTTTTTTT
43[376]	GTAACTTAGTGCTATTACACTACATTTGTCTC
14[247]	CCAAGAAGACTTGCACAGTAACTTCCGCACAA
45[208]	TGGCGGCATTGGCAGTTTCCCGCACTTGCAAAAACAGGAATGCCCCAACTGATCTT
23[19]	TTTTTTTTCGATATATTCGGTCGCTGAGGCTTGCAGGTGGAGGAT
12[215]	TGTATTCTGTTAGATGGTAATCCAGCAAATAC
32[207]	GGAGTCATACCTAAAGGTTCGCGACAGGATTAAATTGACA
45[21]	TTTTTTTTAAGAGGCTGAGGCCCACGCATAACTTTTTTTT
31[368]	GCTCAGAACAAGTGTAGCGAGATCGACGGAGCATGGCTTAGACGGCGG
33[96]	CTTAAGCTACGTGCTGTGTGTGAAATTGTAAT

43[312]	TCTACAGTAGGGTCACGTCGCTAAAGAGAAGG
30[279]	GCAATAAACCTGCCCTATTAAAAATGAAGTTTGGAGCGGGCGCTATTG
8[111]	GTATTAACACCGCCTGCAACAGTGAAATAAGAAGCGCCACCAGAACCA
43[216]	TCCGTTAACTATACGCTAAGACTCGCGGAGTC
40[223]	GCAGCTTTCACCCCACACCGTGATCGGATTGTAACGATAG
40[287]	AGCGAGATCCACCGCAGCTTGGCTGCTCCATGTCGGATTG
23[400]	ACGGTAAGCTGCCCCCTTCAAAGTACTGCGTT
38[383]	CTACCTCGGGGGTCCGGGTTCATAAGGGTCCG
6[455]	ACTGCCGCCTAGTAATGCGTAACATAACTGATAAGAAGGTTTTATTGAGTTTTAGT
14[311]	TGGAAAGTGGATGCTACGAAGCCAAAGCGATC
44[103]	AGCGTCAGTCCTAGCGTTTGCCATCTTTTGGG
23[304]	CAGCAGCCTGCGAGTCAGGTTTTGCGTTTGAA
21[192]	CGGTCCAAAATCTTGACAAGAGACGAGAAACA
41[328]	GCATCAAGACAAGCAGTGATGATGTATCCATATCCTCCAAGAGGTAGC
4[263]	AATGGATGAGATACCCGTCATGTCCGCATCGA
33[64]	TGCTGAATTGTCAGTGGACAGTGCGGCCCCGGCCAGTGCC
40[447]	CAGAATTGGAGAATTGCACCGGAGCTATCCGTCTGCTCGT
23[240]	GTGAGTACCTATTTAACCATACTAACTTTGAC
44[359]	CGTAAGCAGTCTGTTCAACGACTTAAAATCAG
37[408]	TTCGCGGCATTGCGCGCTCTATTTCTATAAGT
34[327]	GTGGGGTGTGGGCAGGGCCACGCTAGTTAGTG
40[159]	CTTATTACGCAGTGGTAAGACACCACGGAAAGCCCTTTTT
11[24]	TTTTTTTTCACCCTCATTTTCAGGCCACCCTCTTAAATCAAGATTGAG
23[272]	GACTCCCCCATACTCCGCGAGGGTCCAGCTAA
23[432]	CGGATGAACAGGTTCGTCAGCGCAACCATAAA
34[359]	TATTCAGACATCGCCGCGCTTAATGCGATCGC
12[375]	TGAGGATTACTAAGAGCGGTTAACATCAACAG
35[120]	ACATTATCGGCCAACGAGTCGAGTAGATTTAGGTGTCGAAATCCGCGA
37[152]	TCCACGCTAGGTGTTTGATGGTGGTTCCCAGC
12[439]	GTCTGCGTTACTTAGAGAAGGAGGATATTACA
43[16]	TTTTTTTTTACCAGGCGGATAAGTGCGGGGTTTTGCTCAGTTTTTTTT
36[119]	TAACGAGCTCGAATTCGTAATCAC
47[184]	AGCAAGTAGTCAGTTTACAAGATATAGGCTAA
44[199]	TCAGATACCGCAGCTCCTGTCGACGCTTTCTT
36[343]	TTATGACATCTCGAATAACTTAGA
6[103]	TATCAGCAGCAAATGACACTGAGGCGGTCAGTTAATCAGTAACGATTTAATCACCG
28[55]	AGGAAGTTACGAAGGCCTGCCAAAAAAAAGGCTCCAAAAGGAGCCTTTTTTTT
24[175]	TCAGTAAATCAATTGGAGCGACTGAGAGAGGAAACGCAATAATAAAAA
6[295]	TTGATTTCGCGGCCTGGGCCAACCGACGTCCCGCATCTCAAACATTAGTCCGAAAG
14[471]	CCCATCGATGGAAAGAAACGGATCATACACAT
28[215]	CACGAGTCAAAACCCAGGAAGGTACCCCTCGG
6[71]	CATAATCAGTGAGGCCCACGGATTTTAGACAGGCCGCCGCCAGCCATACCGTTCCA
42[47]	TAGGATTAGCCGTCGAGAGATGGCTTTTGATGGATTTTAA
26[343]	ATAACTGGCCTCTAAGGCATGCCTAGATGAAT
26[311]	AGGCGGTGCTACATGGCTTGAGTCCAACCTTC
32[79]	GCGCCGCTACAGGTTCCGATGCCATCTGTAAGCAACTCGT
37[376]	GGGCCAGTACATATCGCACGTCGCGAGCTTTC
0[231]	CTGGATGACCTGCTGTCATTGATCTATCGCGCAATTGTAACATCGAAACTTATAAA

35[176]	TGGAACTAAAGGGCCCAAGTTCCATATGCAGCGGATAATT
0[423]	AAGATAAGCGCCAGATATGAAGACCAGCGGGACGTTGTTTAAGGTGTTAATCCATT
18[135]	TAGTCCATATAACAGTGCGAAAGCGAACCAGA
35[21]	TTTTTTTTGAGAATAGAAAGGAACTATTACGCCTAAGTGGCCG
41[208]	AACAAAGAGCCCGCCCCGTAGGTCAGCGAACCCGGCATTCGACGCCAG
6[327]	GAAGCTGCTGCCGTCAGAGTCACTCTTCATAACTCTAGATAGGGTCAT
35[376]	TTTGAGGGTATGGACGCCAGCTAGGGCGCTGGAAGATCACGTTAGCTA
45[456]	AATCGCTCAAATTTACTAGTGTTTTTTTT
35[64]	CCAAAATATAGGGCGATCGGTGCGCCCAGCGATTATACCA
40[319]	AGATATCAACTGCGGAATCTAGACGACTAGTT
2[423]	ATGTTCGATTACCGCAGTGCCGAACTGCACAA
20[135]	TCATGCAACTAAAGTATACGGTCAGGATTAGA
34[103]	TGTTACCTTTTCATGGTCATAGCTGTTAAAGC
35[464]	CAGCTCGTCAAGAACTGAATCCAAACCCTTTTAATCAGGCGTCGCCTA
0[391]	GCCGTGTACAGGAGTACCCACACGTCCACGACTGTGAGCGACGTTGGGAATATGGA
32[135]	CGCTGGCAAGCGCAACAGCTGATTGCCCTTCA
38[415]	CTGATACCCCAACAGATACACGCGAATGGTGG
28[311]	CTAACTACGGCTAGGTCCGACCGCTGCGCGCC
0[71]	AAGTACCGCTGAGTAGGGTTGCTTTGACGCGCTTGTAAAACAGACGTTGTCCTTAG
4[359]	CCTTCCCCAGGGGGTACGGATGGGTCATACCA
43[280]	TCTCTTCGTGCACCTTCGTAGTCCCCGCGCTA
3[344]	AATCTGTCTTGCGACAAGGGCATCCCTGATGT
33[288]	AACACGAAAGCAAGATCTCATTTGGCCATAAG
36[471]	GACGGCGTTCGCCGTTCTAAGGTT
12[247]	CAAACGGCGTGCTGCAATCCCTACACGGCGAA
34[263]	AAAGGAAGCCGCGGTCACGCTGCGCGTGTGTG
40[255]	TGAACGCCTGGCTTAGGTTGTTATCTGCACAATTCCAATG
40[415]	GTGAGGTAATATGGGCATGAAGGGCCTAAGTAATCTTTGC
32[295]	GTGTCACTGCAGCCGTGGGACATAATAACCTA
42[63]	GAGTGTACGTATCGGCCTCAGGTTAAAATTCG
20[103]	GCAGGTAGCTATTTTTCTGTGCCTGAGTAATG
20[231]	AATGGGAATAAGGGCGCGCTGAGATCCAGTTC
20[367]	CTGATTAGTGTACGAGCTCCCTATCTCGGCGC
20[423]	GACTGGGGACTGCTGAAAAACGATAGATTTCA
20[167]	TTGGGAATTACGAGGCGGAAATCTACGTTAAT
28[279]	GTGACCCACGCTCGCAACAGTTACCAATGTCA
6[191]	TAATAAGACAAAGGTTGACGCGACATGGAGCTAACGAGGCTTCTCCCC
30[471]	TATCCCATACTTTGTTAGCAACTATGAGGTTGACCGGCCT
38[55]	AAGCTTAAACTTAAGTAGTAAATGAATTTTCT
32[103]	ACCACCACACGTATCCGCTCACAATTCCACAC
46[484]	TTTTTTTTCTACGTAACAGCATATGCCCG
20[327]	GCATACCGGATACCTGGGTACAAAAATCGACG
28[87]	TTCCTGTAGCCAGGATAAAGCCCCAAAAAAGA
34[295]	GGGCAGTGAAGACCTTTTGCTACTCAATCCTT
8[71]	TACGCCAGAATCCTGAAAAATAAACAGCCGTATAAACAGT
44[55]	CGGGGTCAGTGGAAACATGAAAGTATTTTTTTTT
44[135]	AAGACAAACCAGAAATTATTCATTAAAATGTT
43[88]	GAACCGCCACTCCGCCACCCTCAGAGCAGGCA

42[490]	TTTTTTTTTAAGCTTTTTAGTGTCCACGTTCTGGCTCCGGTGATAGTCAC
14[279]	TCTACGCCCCGTACGGATCACGTGCAATATGA
43[440]	CAGGTAGTTCACCCAAGCCCGGTCAATACGCA
40[351]	ATCACCAAGGGCTACACCCGACGTGTCCCTGCTGCTCCTC
33[480]	CAGACTATAACTCCTTTTTAAGAACCGATTTTTTTTT
36[279]	TAAGGGCGCTGGCAAGTGTAGTAA
33[320]	AAGACGAAGCAGGGATATCTATAGCTCGTTAT
41[312]	TCCATAACAATTCTTGGTCATTGCTATCAAGC
35[88]	CGAACTAAAGACGGAGATTAGAGAATCGATGACAACGGAGATTTGTAT
34[199]	GCGAACAAAGCGAAAAATAAACAAATAATTCG
4[391]	AGACTAATTAGGATACTATGACCTGGTTATTG
18[455]	CGGTCGTTCGTGTTTTATCATTTAAGTCATTC
6[487]	AAGTGACCAAGATTGCTGACGCAGTTTCGTTGGGTGATTAAAATTTTTTTT
35[248]	ACCCTAGAAAGCGAAATAATGCAAAAAAGCGGACGTGCCAGTGCCTGC
47[456]	TGCGCGCTGCCGCGTGGACTTATGCGCTGTGTTCTGTCGG
28[407]	GGAGAATGCCGTGCGTCCAGTATCCCCCGAAT
35[312]	GGACCCGAGCTCTCGCGGCAGGTATCTCAGTTCTGCGCCCACGCGGGG
18[367]	TCTCGCTATCCATCTGTATGGCCGGCCAGGTA
30[407]	TTTCACTCCGCCGGGCTACGACTAGGAACGAGCGGGTTAGAGGACTTC
28[119]	AAACATTATGACCTATCATTTGGGGCGCGTGT
18[167]	CGCCATAACCCTCGTTAAACTCATTATACCAG
0[263]	AATCATCGTCGGTCCATTCCTTGCCGCAAAACGTGAACCATTGACATACGGGGAAA
18[207]	TGGGAGTTTCTTGAGAGGAGGGGACATATGAA
13[192]	TTAATCGGTGTTTACAACTGGTTACGGGGCGA
2[514]	TTTTTTTTAGAATCTAAATTCACGATTAACTTTTTTTT
33[128]	GAAACCTGTCGTGCGAACCAGTGAGACGGCGG
0[455]	GTGGGTCGCATTTGGGAGGCATATAGACCAGACGCGACTCTAGCACATCGGAAAAT
22[514]	TTTTTTTTAACGGTCTAATAGCAGGACTCGTTTTTTTT
40[95]	GAACCAGAGCCACCTCCACCCTCAGAGCCGACAGAATCAA
36[497]	TTTTTGCTTCTTAATTGCTTGGCTCTCCGTGCAGGTCTCTTAAAATGTATAGTACTTC
20[514]	TTTTTTTTTAAATACTGCGCATGTCTCCCCTTTTTTTT
45[240]	CAGTAACGCTCGATATCCTCTATGATCGCTACAGGCATCGTATAGAGTAAGTAGTT
0[295]	CTAGCCACGGCCTTACTTTCCGGGATTATAGACCGGTTAACATGACACAAATTATT
16[514]	TTTTTTTTACCATGAGCCATAATAGGGCATCATCTACGTTTTTTTT
40[199]	TCGACACCCTGAACAAAGTCAACTATAATAAG
30[375]	CAGGTGTCAGGCCAAATACCCATGCAAAGGATTGTGCGCGTAACCACC
30[508]	TTTTTTTTGTTGTTGTAAACCAGAGCGATTCAGATTT
25[192]	ATATTCATTACAGAGCTAAATTCAGGCGACTTAAATGCCCCTGTGCGG
30[215]	CAAGTGCCCTTCGCTTACTCGAACATGCATCACTCCATATTTGAATGT
37[464]	CTCTCGCGGAGAGACCTCCGGGTTACGTCATT
3[19]	TTTTTTTTATCCGGTATTCTAGTAACACTTTTTTTTT
26[247]	GCGACCGAGTTGCGTACTTACTGTCATGCTGT
14[87]	CGTAAGAATACGTTATAAGAACTCAAACTAAA
28[375]	TGGCCGCACATGTGAGTGTCCGGGAAATAAAA
12[471]	TTATATCGAAACTTGGCACCGATACTAAATAC
42[447]	CCTCTGCGTCAGATGGGAGTTTGTCCTCGCCG
44[167]	AGCAGATATAGCGGAATACCCAAAAGAGAGGG
4[455]	AACCGCGCTGAATCTGACTTGGACTGGCAGCA

20[207]	GTTCCACCTAAAAGACGCAACCCGTGGGCTGA
44[231]	TCGTATGGGTAGTCTTACAAATGTAAAATCGG
28[508]	TTTTTTTTGAGCCTATGACAGGATCAGGTCACTGAT
4[327]	CGTCGCCCGAACACTGATCTATGAACGTTACT
28[343]	CTAAGTGAGCAAACCCTCAACTTCCAGGTGGA
30[119]	GAAGCCTTGATAAATTTTTGACCATTAGATACGATCCCCGGGTACCTC
14[335]	TGTGCCAGTGCGGTTACCACTCAAGCTGATAC
43[344]	TTTGTTTGTCCAGCGCGTGCTCAATGGTTCAT
2[487]	CCAGCTAGTGTCGGGTGTACCATCTTATCGTG
43[408]	GCTTCTGGCTCAATCGCCTTATGATAGAATCT
12[407]	GGGTGCAAGACCGTGTGACTCCCAAACTCACA
14[151]	TAAGACGCTGAGAAATTCATTTCAATTACATA
18[71]	ATCTTCGCCATTCAGGACCGTGGGAACAAACG
25[24]	TTTTTTTTTCGTCACCCTCAGCAGGGCCGCTTTTGCGGGATTTTTTT
6[351]	CATCGGACTGGTTCGGGGTTCTAGAATACCCTTAGGGTCATTCCGTTA
28[439]	CTGCCCGGAAAGACGTGGGTAGCATCACGCGA
18[327]	GATGAAGCGTGGCGCTGTATTTCCATAGGCTC
45[376]	TAAGTGTCAAGTCAACAGGACAAGAGTCCACTTCAGCGTGACAAGCTT
10[55]	CAGAACCGGATAGCAATAATTTTAGCGAACCTCCCGACTTGCGGGTTTTTTTT
5[19]	TTTTTTTTAGGTTTTGAAGCCAGAGCCACTTTTTTTT
18[514]	TTTTTTTTCACTTCTCGTACATTCTTGTGGTTTTTTTT
13[488]	ATAAACGTTTAACTTTTTTTT
42[359]	TGGTTACCAGGCCCCTAGCTTCATCTCATTAAAATGTCCTTAGAACCG
34[506]	TTTTTTTTGGTATTTCGACCTTCACCGCCTTTTTTTTT
12[311]	CTATTGGATTGTCTCCAAGGCTTATACAGTAG
2[71]	GTATCTTTGATTAGTACAAAGCACGTATAACG
32[367]	AGGAAACTATACCCAGGGCAGCTTGATGCCCCTTTATACAT
6[514]	TTTTTTTTGATATCTGACATCTACTAGGTTTTTTTTT
6[135]	TTTAATATACAGTAACGAAAAACAGAAATAAACAATAGAATAGCAGCCACCGTCAC
15[40]	GCGTAACGTTCCACAGACCGCGCCCAATAACA
37[440]	GTAAGTAACGATGGGCCCATTTCGTGGTTGCG
36[199]	ATTTAACTAAATCGGACATCCATTTTCAAGTGAGGGAACCGAACTGAC
2[359]	GGGTGTACGTAGACCCGTTGTCCAGCTCGTAC
47[296]	GGGCATGAGTATGAGCCACTCAGCCTCCGCACACGAATGA
26[151]	CTGACTATTATAGTACATATTCATTGAATATA
1[352]	GCGAACGTCCAACGCGTCGTTACGTAAACGAGCAATTGTCACCTCATT
7[344]	CTCGCTTATGTTGTTTAATCTACCGCCAGGTAAAACAGGTGCTGCTTGAGTTAGTT
14[407]	TTTCCGCGGCACAGGCAAGGTACGGGGTAACT
47[320]	ATTTGCGCGACGTTAAAGTGGAGATTAATGAG
12[175]	TCGATGGTACCAGTATAAAGCGTCCAGACGAC
2[103]	TTATATCTGGTCAGTTTGAGATAGCCCTAAAA
10[343]	ACGAACCAAAGTTTTGAGGGTCTTTCTATATA
30[151]	TTCAAATAATGTTACTAGGCTTTTGCAAAAGACGCGGGGAGAGGCGAA
33[416]	ACCCTATGTGCTTCCTTCTGCGAATATTACGA
47[72]	GGTTGCACTGAATTTATTATTTATCCCAATCCAAATGGATTATTTCGG
18[295]	TTCTGGTCATGAGATTTTACTTCGGAAAAAGA
27[192]	CCTTCATCAAGAGTAATAAAACCAACAAGGCC
30[55]	CTAAAACGCTTTGACCGGCCTCTTCGCAACTAAAGGAATTGCGAATTTTTTT

45[80]	CAAATCCTCAGAAGTAGCGCGTTTATCACCGTTCTAGCTGCCAGGGTGAGAAAGGC
44[263]	TCCCGTGATACGAAGTCAATAGCGGACGACCT
26[407]	CCTTGTGTACGGCTACTTCTCAGGACCTCCTG
32[239]	AGTCATAGTGTCCCCGGCGATCAGGGCGATGGCCCACTAC
34[391]	CGACTCGACGTAATAGTTCCTAATACTCGGTC
23[464]	GATCTACAAGGCATCCGGAACGTAATCCGAGT
9[24]	TTTTTTTTAGTACCGCCTGCCTATTTTTTTTTT
24[215]	CCTTACGGTGATCCCATGTCTAGTGCAGTTACCCATATGAAAGTAGGAGTTAATAA
26[175]	CCAGAACGACGGTCTCCCAACACAACTTGTAC
36[407]	AAGCCATAGTGTCAATAATGCGTG
27[24]	TTTTTTTTGAGGCTTTGAGGACTAGGTAGCAATTGCGCCGACAATCCA
26[119]	CTCAGAGCATAAAAGGATTCTACTAATAGCAA
47[360]	CCTGGTTTTGCCTCGATGTCATCGGGCTACCTGGGAAGCT
47[104]	GTTTGGGAATAATCAATTTGTTTAACGTCAAAGACAACTC
11[360]	CTCAAAATCGCATGCGGTTAGAAGCGCGGTAC
23[144]	AAAACGAGATAGGAAGGTAAATATTGACGGCA
33[224]	TCAAAAGAATAGATTACGAAAAACCGTCTTTA
38[127]	AAGCATAAAGTGTTCCCCCGCTTTGGTCACGC
8[431]	ATGTTTGAGTCCCATAAAGTCCTGAGGTATATTCAATTGTCCTGTGCC
2[391]	GGGTCCACCCATTGATTATGGCTGAAGTTTAT
38[95]	ATAGGGGCCTTGAACCTAATGAGTCGCTTAAT
44[423]	TATTTCCAGAGAAGAAAATCTGGCGGAGCCTG
44[484]	TTTTTTTTATACAGGTCCTAACAGCCCAAAATTTCTC
13[360]	ACTTTGGCAAATGCGAGTGATCATGGGCAGTA
32[167]	AGGGAAGACCCCCGTCTATCAACTACGTGAACTTTATATG
11[192]	TTCAGCTCTTCGTTGCCGTTGAGAACATAGAG
18[391]	ACGCGATGGCCGGAATAGGTGTAAATTTTAGC
43[184]	TAATTGAGGTGAAGAATTGAGTTAAGCAAGTA
9[368]	GCAGGAGATGTACCAGTTCCGCTCGCTGTGGA
10[215]	ATGCTATTTTGTATAGAACAGGAATCGAAAGC
46[367]	GACATCACTAGGCGTTGGTTTATCACGCGCCCGGGCGAGT
14[439]	GCGCCAAGAGATCGCGGCCTGACGCCCTCCCT
44[295]	AGGAAGCAGCTTAACATTGTAGCATGACATAA
40[383]	CCGTCGGTGAATCTTCTCAGTTCTTTCTGATGGCAACAAA
4[514]	TTTTTTTTGGGGGTGAAGAATGGGGCCGTTTTTTTTT
11[488]	TGAGCGACTTTCATTTTTTTT
9[488]	CAGCACGATAAACTTTTTTTT
10[279]	TTAGCTTAGACTTATGAGAGATTAGGTATCCA
24[55]	GAGTTAAACGAAAGACCAGGACAACAACCATCACTCCTCAAGAGATAA
2[455]	GTTTGTGTATTTACGACGAGTCAGTATAGAGT
36[87]	TTTTCTATTTACGCTCGCCCTGGG
36[439]	CTAGGTAGGTGAAAGGTTTGGATT
28[247]	GGGATAATACCGCAGGCACTCATGGTTATAGC
21[472]	ACACGAGTCGGTAATTATAAAGCACACCGTCG
26[375]	ACTAAGTATTTTCAGGTAATACATCTCTTAAA
26[215]	GTTAACTGAGCACAGTAAAGATACTGCAGAAA
1[19]	TTTTTTTTCGTAGGAATCATTACAGCCCTTTTTTTTT
10[311]	AGATCGGGCCAGAACACCTCGCCTGTGGGACG

0[199]	GGTCTGTTTATCAACAGGGTCATGCAAGGGACCGACAAGTAAATTCCC
20[455]	TCGCCAACTCAGGCGCGCGATCCTCAAGTATG
38[447]	GTCGATCTAACTGACTCTCTCGGTTCTCTGTT
3[192]	GACAACAGGGTCTGTAATTATTCCTTATTGGT
38[500]	TTTTTTTTGGTTTCTCGCCATCAATCATGTAGTCCTGGAGTGACTAAGTATCA
47[168]	AAGAACCAGGCATGATCGCATTAGACGGGAGACCGCTGTTAACCTAAA
7[19]	TTTTTTTTTTTTATCCTGAATCTTACCAACGCTAACG
29[24]	TTTTTTTTAATACGTAATGCCACTTCCATTAAGTTTATCAGCTTGACC
38[255]	TAATCAAGTTTTTGGGGAGCTTGACCTGTTCT
40[63]	GTAAGCGTCATACGGTTGAGTAACAGTGCCATTGACAGGA
47[136]	ACCAGGCAGAATTATCTTTACAGAGAGAATAAAATCAATA
26[279]	ACCATCTGGCCCCTTGATCTCAGCGATCTCTA
32[455]	CAAGTTACACGCGATCTGAGCCGCGAGGCTGT
33[184]	AAGCCAGGGGCACGCGTTACCCGGGTTCCGCG
40[127]	CGACTTGAGCCATTTCAACGTCACCAATGTCATATGGTTT
19[192]	ATGTGCACGGCGCATAGGCTGGCTTGAGATGG
20[71]	TAAGCTTCTGGTGCCGTTCATAGGTCACGTTG
38[199]	CACATTCAAGTTTTTTGGGGTGTCGACGGGGA
47[264]	GAGAGGGTTAATGGGGTCTAACGACACTGCTTCAGTAAGC
10[247]	GAAACCCTGCCAGAGAGTTCAGATCGTGTTAC
47[424]	TTCAGGTCTGCCTATTCGATTTTATAGGTGCTCGGGCTGC
0[167]	CAAGAAAAAAAAAAGCCGAGAGACTACCTTGATCCGCCTGGAAGCGAAAAAATCCCT
42[223]	GCTACGGCCGTTTCTGGGTGATTCTGTGACTG
47[232]	GCAAACGATAATTTATTCCTGATTGAACACCGGAATTTCC
17[48]	AATCTCGCCTTCAGCTGGCGAAAGGGGGATTTTTTTT
44[391]	AGTGTATCCCCTTTGTCTAGCGCCTAACTTAC
34[55]	AATTCATGCGCACAGTTTCAGCGGAGTTTTTTTT
30[87]	TGAGCGAGACAAAGTAACGGTAATCGTAAAACACCCCGCTTCTAACTC
0[135]	GCATGTAGCGAATTATATAATCCTGATTGAGCAACATACGAGTGTAGCCCAGTCGG
19[19]	TTTTTTTTTTTAATTGTATCGACGGGTAATTTTTTTT
12[343]	GTCTTCAGGATGCAGCGATCTAGGTCGCCTGA
44[327]	ACTGAATACACAAGGCAGATAGCGCACTGGGT
4[423]	GCTACTTTCCTTGGCGTCTTCAGTCGCGGTCT
8[271]	ACACCCTGACCTGCTAGGACAGAGCCACTGCAGTATCGCAAATGCGAT
45[336]	GCACGGAGTGAACGCCATAGCAGTCGATGGCCGGTTAGCCCTCGATCTTAAAGGAC
0[359]	TAATTCCGACCACTGGCCCAAGGCAACTCGGACGCTGTCCGTTCCGGT
6[263]	GCTAATCGCTGAAGGGAATCCTTAGGGAAAATACTCTCATTATATACTAATAGGCT
34[71]	ATGACAATCCAGGAGTGACTCTATGATATCGG
4[71]	TGCCCATCACGCAAATGCCAGAGCGGGAGCTA
33[344]	TCTAGTTGATAGCTTGCAAGCTACCTACAGGG
12[87]	CCAACAGAGATAGTTTTCCAGAACAATATTCA
30[439]	ATCGCTGCGCGGCTCAATATACCAACCTTGGGCTGGAAATTCGCTTGA
39[360]	CAATGACAGCCTGTAGGGTTTGAACGGGTAAGCCTCCGGTTCGAGGCT
0[103]	TTCCTTATTGAAAGGATAATGCGCGAACTAAGCGGTGGGCACCCGCCGAAACAGGG
8[239]	AGATATCTCGGTAGTTCTGAGTTAAATCAGCACGCAGAACTTCTTCAA
24[343]	AGCGGATGCACATAAACTATAAACCGAGGCCCCCCCGTTCAAACTTGGGGATACCC
40[490]	TTTTTTTTTTGAAGAGAACACAGCCCGCGGTGTAGGCCTTTTTTTTTT
42[191]	TATCAGAGCTAAAATGTTTCTACAAATCAACGTAACAAAGCTGCTCAT

26[439]	ACATGGCTGCCATGACCCCCCCAGGATAAGTG
43[448]	GGTCTTTCTCAACGTGTTCTACCGGGAGCAGGGGTTTTTTTT
26[55]	GGAACGAGAAGACTTTGAACTTTCGAGGTGAATTTCTTAAACAGCTTTTTTTT
42[95]	AGAGCCGCAATATGATATTCACAATAAATCAT
8[303]	CAGAACATTAGGAGCCGTATGTTCTGCGGGGCGTCACCCATGCGTTCG
8[399]	AAACAGTCAATTAAGCCATGTATGTTCGGATTGCCACTGTTGTGTCAC
18[103]	TATTCAGGTCATTGCCTAAATTTTTAGAACCC
8[463]	ATGTCGAGCTTTATGCCGGCAGGGACTGTTCGCGGTGGTTCAGATCCG
37[280]	GAAGTGCAATTTCGATGTTAGACAGCAGCGAG
18[423]	CGCGCCTTGGAGTCAAGGTGCGGATACTCGCA
14[119]	GGAATTATCATCACTTATTGAGGAAGGTTTCG
36[311]	CAAGGCACCGGGGTCTCGTGCGTC
6[167]	ACGATCTTCTGACCTATGCAGACAAAGAACGCCTATCTTACAGGGAAGTAAGACTC
25[360]	TGAGCAGTGTCTCATGATTGACTCAGTGCGTCGCCCCATGGTAGAGCG
36[359]	ACACCCTGATGTTTGGATCGTCGAATAGGCCAATGCATAGGGCTACTGAATATCTA
35[280]	CAGTCCCCGATGAAGCCTCTAGATCCTTTTAACACAGTCGTTTCGAAT
45[56]	TAAGTTGGCCTTGATATTTGAAGATCGCACTCAGCAACCGTGCATCTG
35[440]	ACGTCATCTCCTGCATACGCTCACCGTATAAGTCATAACGGGGCTGGC
12[55]	TAGGAACCACAAACTAATAGCAAGCAAATCAGATATAGAAGGCTTTTTTTTT
34[231]	ATAGGGTTTTTGGACTCCAACGTCAAATGGGG
1[192]	CGCGCGATTACGCCTATAAGTTGGCATCACCCCAGGGCGAACGTGGCG
41[16]	TTTTTTTTGTGTATCACCGTACTCGTATAGCCCGGAATAGTTTTTTTT
35[216]	TTTTATGTTGTTCCAGTCCATTTATCAGGGTTGGCCTGCACACGGGTG
33[21]	TTTTTTTTTGCTAAACAACTTTCAACGGTTGGGTAACGCCTTTTTTTT
42[383]	TAGATAGTGTTCCAGTTTGGACCTCGCAAGTA
26[87]	GGAACGCCATCAATAGAATATTTAAATTGAAT
42[287]	AGGTAACAAGGATCTCAAGAAATCGCCACTGG
38[287]	ATCGACCAAATTAAACTTACGTCGGTTGACTA
15[24]	TTTTTTTTCATAGTTAGTATGGGATTTTTTTTTT
27[352]	CCCCTAACGTGTCCCACCATTGGGGAATCTCT
33[448]	TAGTTGCGAAGGCACTGACCGTGTCTGGCGAG
45[304]	AGATTAAATCTAAGGGCTAGTCTGTGTCTCGTGCGCTCTCGTAAACCCGACAGGAC
38[159]	GAGAGTTGCAGCATTCAATCGGCAGGAGCGGG
4[207]	CGTGCGTCGCAAAGGTAAAGTAATCATCAGAA
0[55]	GAGAACAAGCAAGCCGTTTTTATTTTCATTTTTTTT
5[192]	GACAAATACTTTTTCTTGCACTCGTTGAACCG
8[143]	TATGTATTGCGTAGATTTTCAGGTAATGAAAAAATAAACCATCGATAG
36[183]	ACCCAAGAGTCCACTATTAAATTG
4[487]	GAGCAGAGCCCTCATACCTAAGTTTCACATCT
36[151]	GGCGGTTTGCGTATTGGGCGCAAT
31[24]	TTTTTTTTACACTAAAACACTCATAAAGAGGCACGTTGAA
23[112]	ACAGGCAAAGTCGGTCATAGCCCCCTTATCTC
38[319]	CCGTGCGCGCCTCGAACGGCTTGGGCACTATA
14[175]	GGGGCTCTTGTTTAGTATCATCTAATGCAGAA
24[508]	TTTTTTTTCACACTGCTACCCTTGGATCCTTGCACCACGCTTTTTTTT
45[144]	GGGAGAAGGTGGCGAACAAAGTTAGTGCATTCAACTAATGTTTACATTATTACAGG
2[207]	TTCGGTGAATGTAAACAACATGTTACCCGGGG
10[175]	TACTTTGCTTAATTGAGAATCTATAAAGTACC

5[344]	TAGTCTCTAAGAATCTGAACTTTAGTACAGAA
42[127]	AAATCACCGCTTAATTGCTGAAAACAGTTCAG
7[168]	ACATGTAATTTAGGCAGAGGCATTTTCGACGTAATGTACG
43[152]	AGCAAACGGCCAACATATAAAAGAAACCGCCA
45[112]	ATTTTAGCACCATGCGACATTCAATTTATATAATGCTGTATAATTTTGATAAGAGG
32[327]	TCTTGGCGGTATGTTGCTCCCCGCTAATAAGAGCTACAAC
37[184]	TGCCGTAAACCAGCCCCCGATTTAGAGAATAG
37[88]	CTGACGCACGATCTGACCTCCTGGTTGACCTT
30[351]	AGACAAGGAACCGTAAACATCGTAAGTACCTTCTGTCTTAAACAGAGG
39[200]	CGAAAAGTGCCACCTATTTATAGCCTGAGACTTAGACGTATATTAACC
31[200]	CAAAGGGCTGTTTTTAATTGTCTCATGTGAACCTACTCTTTCACAAGTTGAAAGAG
43[248]	GTTTTCTCCGAGAGGTCATACAGCTTCCCAAG
29[352]	TTCCTAAATGGGGAATATGTGAGGTCCTCTCC
18[231]	TGTTCATACTCTTCCTCATGGGGCGAAAACTC
42[415]	CTATTGCCATAAGAGCACAGATCTCAGCAACA
17[19]	TTTTTTTTTAATAATTTTTTCAAAAGAATTTTTTTTT
2[231]	ACGAAAGTTTAAAGTTTGCCTTCGAACGGTAT
19[472]	TGGGTAAGTGGTCTTGACACGGGCGTCAGAGC
33[256]	GCCGGCGAACGTGCCGCACCACACCCGCCCCC
35[336]	TTGCCGCTGGGGGATTTAGCTCGAGCTTAGTCACGCTGAG
6[231]	CCGACGATAATGACGTTCATGGCGGAATAATAGGGTCGTACGGGCTGATTTCTGTG
21[344]	CTGTGATCCAATCTTACTTCTATACGGGATGG
2[167]	ATTAATAAACACCGGATTATTTAACCTCCGGC
2[135]	GATATACCAAGTTACAAGGTTTGGATTATACT
10[87]	TCACACGACCAGTAGGAGGAAAAACGCTCATC
18[263]	GGTGATCAAGGCGAGTACTGCGCAGAAGTGGT
20[391]	ATAAACGTCAAAGGGCCCAGAACATGATCTTG
32[487]	TATGCTGCGAATTATTTTGGATTTTTTTT
46[207]	GATTGTTGCCTACTGTAATATCATCGGAGTTGAAGTTTGA
23[80]	CATTAAATCACAAATCCTCATTAAAGCCACAG
38[359]	CGCTCCTCCTAGGTAAAAGACGGCCGAGCTTA
34[135]	TGCATTAACCTCAGGGTGGTTTTTCTTAGCGG
10[375]	CCCAGTTAACGCTTACTGGGACCTTAGACGTG
46[47]	TAATGCCCCCACCCTCTCCAGAGCAGGAGGTTTTTTTTTT
34[167]	CCCGAGATGGTGAACGTGGACTCCAACCGAGG
21[19]	TTTTTTTTTTGATACCGATAGCGGCTACATTTTTTTT
4[231]	GGACCATGGTGGGCTTGAGTGTCTCTTGATCC
30[247]	AAAGTGCTGGTTGATTTTAGCTCCTTCGGTCCTTTGGAACAAGAGGGA
26[471]	TAAACAGAGGGATCCATGGCCTACAAATGGGC
12[279]	ATTATGATACTCTCAGATAACGAGTTTGTGAG
10[151]	TGTAAATCGTCGCAAAATTTAACAATTTCTTA
33[384]	TGAGTGCGCAAGCCTTTTACAGCTCACTGTGC
37[312]	AAGAGTTTCAGCACCCCTGAGAGCATGTTGTC
38[223]	ATATTTTGTTAAAGGGCAAAATCCCCGCGCCG
0[327]	TTCCAACCCGTTGTACAGTGGAGAAGAATACAAACGCGATTGTCTGTCGACCTTGG
35[408]	ATGGAGGGGCGGAGTGCGGCCTCACTTAACGATTCTCTTCGTTATTCC
37[216]	CGTTAAATGAGACCAATAGGCCGAAATGCATA
26[508]	TTTTTTTTATGAACAAACACCCCGGATTCATCCGTTT

24[375]	TTACCGACCGATCAATTATTCCTGTACGACCGAGAAAAATAGCATGGT
20[263]	GCATGGTATGGCTTCACAACCATCCAGTCTAT
37[248]	TCGAGGTGGGAAAAGGGAGCCCCCGATCCGAG
2[327]	AAATTGGCACTCGACTCCTTACGGGTAAACAA
2[295]	GAGCGACGGATGGCGTGCCCCGCGTCTGCTGG
10[407]	CCCGGTGTTTCCCATCACATACACCTTCACGA
2[263]	TATCTTTTAAGCTGGCTCTCGAAACTGTTCCT
6[423]	GCCTGTAAGAGGAGACGCGTCAGGGGAGATAATCCTCCATTGGACCTGTTTCTTCC
36[247]	ATCTCCACTATTAAAGAACGTTGT
12[151]	AGAATCCTTGAAAGAAGAAACAAACATCATAA
43[120]	AATTAGAGAGGTACCATTAGCAAGGCCCCTTT
32[399]	TGTCGCAGATTATCCTCTTTGTCGATGCACGTGGCGGGTG
8[191]	GGCACCAGATTAACTGGGAGAAACAATGAAAT
12[119]	TTTTGCGGAACAACGCGCACTAACAACTACGC
20[295]	TATTCAGTGGAACGAATACGCAAACAAACCAC
33[160]	TATAAATCAAAAGCTTAAAGGGCGAAAAATGA
32[263]	TTATCGGTTCAGCGCTTAATGCTACATAGTTC
41[152]	CATAAAAACCGATAAGTTTATTTTGTCACAAT
	**Square-capture-Z_1**
5[56]	AGGTCGTTAGACACCATAAAAGAGTCTGTAACAGGCCGATTAAAGCCT
7[56]	AGGACGTGGTAAGTCTGAAGTGTTTTTATTTTGACGCTCAATCGTCTG
24[87]	TTTTGTTAAATCAGTTTGAGGGGACGACGACAATGTTGGAACCTTGTA
21[56]	GCTTTCCGGCACCGCAATGGGCGCATCGTATCGGACTGGGCCCGGACG
1[56]	AGGTCGTTAGACACCATCACTTGCCACTCATCTTTTGTCGTCTGCGCG
3[56]	AGGACGTGGTAAGTCTTTGTAGCAATACTATACCTCGTTAGAATCCAA
19[56]	AGGCAAAGCGCCAAGATGACCGTAATGGGATGATGTTGGAACCTTGTA
36[63]	TGGATGTTAACTGTTGGGAAGCATCGTCGGATTCTCCAACGGACTGGGCCCGGACG
11[72]	AACAATAAAAGGGACAATATACCGCCAGCCATCAAGCTATGAAACCGG
46[79]	CCACCAGAGAAACATTGGCAGATTAAAATGGAAATACCTATAAGAGGGTGACTTGA
25[72]	TGTACCCTATAGTAGTCCAGCTCATTTTTTAAATATAAACGTTAATATTTCACAAA
27[72]	AACGCACGTAAATCATGTGAAATAATTCGCGTGAGCAGGAAGATTGTA
15[72]	TACGGCACAGACAATAGAACGGGTATTAAACCCAAGCTATGAAACCGG
13[72]	TGCAACCCTTCTGACCGGCATCGGCCTTGCTGTAAGAGGGTGACTTGA
29[72]	TGTACCCTATAGTAGTGCGCTTTCATCAACATTGAACCCCGGTTGATA
31[72]	AACGCACGTAAATCATAGCGCGAATAACAACCGTCAATCATATGTGAG
5[88]	TTCTGATCAATTACAGTAAAGCATCACCTAATGATAAAACAGAGGCAG
7[88]	AGGGAGAAAGAACTTACCACGCTGAGAGCTAGACTTTACAAACAATTC
24[119]	GGCAAAGAATTAGAGACAGTCAAATCACCATCGCCACACGGATCATCA
21[88]	TAATGCCGGAGAGTCATAAAGATTCAAAAATAGGTATCTTTGGGAAGG
1[88]	TTCTGATCAATTACAGTCAACAGTCATTCCAATTTTTGAATGGCTATT
3[88]	AGGGAGAAAGAACTTAAACCCTCAATCAAGGACATTAAAAATACCTGG
19[88]	TCTACAAAGGCTATTTATTTTAAATGCAAGCCGCCACACGGATCATCA
36[95]	ATAAATCATCTGGAGCAAACATCGCGCAAGGATAAAAATGGGTATCTTTGGGAAGG
11[104]	GCAAGTTTGAGTAACAATTATAGATTAGAGCCAATGTGAATTAGTCTG
46[111]	CAGCACCGATTAAATCCTTTGCCCAGTGAGGATTTAGAAGCAGGCTTCGCAGTCCT
25[104]	CGGTAGCAAAAGATCCCGGCAAAATTAAGCAAGCTTAGTAGCATTAACTCATCGGC
27[104]	CGCACACGCGTTGTTGTGTGCTAAATCGGTTGCGGAGCTGAAAAGGTG
32[111]	TGCGCGTAAGTTATTCCTGATTATCAATAATCGGCTGTCTAATGTGAATTAGTCTG

13[104]	CATAGAAACCACCAGAAAAATCTAAAATATCTCAGGCTTCGCAGTCCT
29[104]	CGGTAGCAAAAGATCCTCACTGTAATACTTTTTGAACCTGTTTAGCTA
31[104]	CGCACACGCGTTGTTGCATCGCCTTATTTCAACAAATGGTCAATATTC
5[120]	AGGCGGACTAGGAAGTTTTTACATCGGGATACTTATTTGCACGTACGT
7[120]	GACCCAGATAGGACTCTTAACGTCAGATGTTAATGGAAACAGTACATA
24[151]	AATGACCATAAATTTTTTGCGGATGGCTTAGACGCTATTGAACGGGTC
21[120]	CATGTTTTAAATATAACTTTAATTGCTCCAGCAGTGGACGGAACACGC
1[120]	AGGCGGACTAGGAAGTCGCAGAGGAAACCAATCAGATGATGGCAATTC
3[120]	GACCCAGATAGGACTCCTGATTGCTTTGAGATTAATGGAAGGGTTTCA
19[120]	CTGGAAGTTTCATACTGCAAACTCCAACACAACGCTATTGAACGGGTC
36[127]	GAGTGAGCCCAATTCTGCGAATTTTAATTCGAGCTTCGGAAGTGGACGGAACACGC
11[136]	TCATATTAATTAATTTAAAAGAAAACAAAATTTAAGACCTTAATTCGC
9[136]	GAAGAGTGAATAACCTAATATTTGAATTACCTGAAATCAGCTAAGAGT
25[136]	CGAACACAGAGAACTCTCACAAAAATCAGGTCCAGCCCCTCAAATGCCCGATTGA
27[136]	GCGCCTTCGAGGACTAGAGTCAGAAGCAAAGCATATACTGCGGAATCG
32[143]	CGCTAGGGATCAGAGTCAATAGTGCCATCCTAATTTACGATAAGACCTTAATTCGC
13[136]	TCTACATAGCGATAGCATCCTGAGCAAAAGAAGAAATCAGCTAAGAGT
29[136]	CGAACACAGAGAACTCCCGAAGAGGAAGCCCGTACATAGTAAAATGTT
31[136]	GCGCCTTCGAGGACTACCTGCTCCTCGCGTTTGCCAGAGGGGGTACAG
5[152]	ATTCGTACGGGTAAGCATGGTTTGAAATAGGCAAATCCAATCGCATTC
7[152]	TTCCGCCAAGGTGCGATTTAGTTAATTTCCCAACTTTTTCAAATATAT
42[159]	ATACATACTTTAGGAATACCAGTAGATTCATCAGTTGAGAGACTGGAAAAGATCGG
21[152]	CATAACGCCAAAAGCAAACTAACGGAACAACCGGGCTGAGCCATGAGC
1[152]	ATTCGTACGGGTAAGCATTACTAGATAATATCAATTTATCAAAATCAT
3[152]	TTCCGCCAAGGTGCGAGGCGTTAAATAAGCTTTGGGTTATATAACCTT
19[152]	AGAGCAACACTATACGCGTTGGGAAGAAATTGGACTGGAAAAGATCGG
36[159]	CCCCAGCAACGACGATAAAAAGCTTTTTAAGAACTGGGACGGGCTGAGCCATGAGC
11[168]	TGATATGGGCTGCGATTCTCAACGCTCAACAGGGAGCACATTTTTTTT
46[175]	AGCAATAGGAGCACTGAAGCTCAGGAAGCCATATTTAACAGTTCTCGATTTTTTTT
25[168]	TTTTTTTTTTCTATCTAGGAATAAGGCTTGCTACGCGCGCTGAGGTCCCAGAAGG
27[168]	TTTTTTTTTTAGCTTCAAAACGAGTAGTAAATCGCATGAAAACTAGGT
32[175]	AGAAAGGAAGGATAGATATGGCCTATAGATAAGTCCTGAAGGAGCACATTTTTTTT
13[168]	TTACAGTGGGAAGTCGCAGATGCGTTATACAAGTTCTCGATTTTTTTT
29[168]	TTTTTTTTTTCTATCTCATTTCAACTTTAATTGCCGAGGAATTATAT
31[168]	TTTTTTTTTTAGCTTCCAGACGGTTTATGCGAAGGCGGCTAGGTGGAC
	**Square-capture-Z_3**
5[216]	AGGTCGTTAGACACCAAAGGTGAAGACCTTAACTCTACGAGGGGTAGT
7[216]	AGGACGTGGTAAGTCTCGGAACATCCGGCGGCCGCTCTGGCATATCAC
24[247]	TCAACCAAGTCATCATCTTTTACTTTCACCAGATGTTGGAACCTTGTA
21[216]	ATGCCGCAAAAAATCTACCCACTCGTGCAGGCGGACTGGGCCCGGACG
1[216]	AGGTCGTTAGACACCATCGGGTCCTAGTATACCGATTACATGCTGACG
3[216]	AGGACGTGGTAAGTCTTGACCGACTGACAAATCTGAGTCGATAAAAAC
19[216]	AAATGTTGAATACTATATCTTACCGCTGTCTCATGTTGGAACCTTGTA
36[223]	TAAATCAGATATTATTGAAGCGATGAAAACGTTCTTCATCGGACTGGGCCCGGACG
11[232]	CTCGTTAGGCTCGAAAATCTAAACACTACCTGCAAGCTATGAAACCGG
46[239]	TGGTGAACGCTGAGTACATCAAACGCCGACTAATCCGGGGTAAGAGGGTGACTTGA
25[232]	TGTACCCTATAGTAGTCAGTCTGAGAATAGTGTGGCATCCGTAAGATGGACTAAGC
27[232]	AACGCACGTAAATCATGATTCTTGCCCGGCGTTTCGGCAGCACTGCAT

15[232]	GAGCTCGGGGATCCAAGGGACTTTAGACCACCCAAGCTATGAAACCGG
13[232]	TACTCTTACGTATGCCGTCGCACAACCACAAATAAGAGGGTGACTTGA
29[232]	TGTACCCTATAGTAGTTCAGCCACATAGCAGATACAAGTTGGCCGCAG
31[232]	AACGCACGTAAATCATAGTCCGTTCATCATTGCGTTGTCAGAAGTATG
5[248]	TTCTGATCAATTACAGCAACTGTCCATTCATACGTGGATACGAGCTAA
7[248]	AGGGAGAAAGAACTTAAATGTCGCGGTATCGTGTCCGTATCGGCGTAC
24[279]	GTCGTGTAGATAACAGTTAATAGTTTGCGCAAGCCACACGGATCATCA
21[248]	CACGCTCGTCGTTCCTTTGCCGGGAAGCTGCGGGTATCTTTGGGAAGG
1[248]	TTCTGATCAATTACAGAGTCATTTTGGCTCATATATGGTGTGATCCGC
3[248]	AGGGAGAAAGAACTTATTGAAGCAACCGCACAATGCAATCTGACCGCG
19[248]	TCCGGTTCCCAACCTGACTTTATCCGCCTTACGCCACACGGATCATCA
36[255]	AGCACTAAATCCCCCATGTTGATCCCGGAAGGGCCGATTAGGTATCTTTGGGAAGG
11[264]	TTGACAGGATGTAACAAGGGCGTTATCATGTGAATGTGAATTAGTCTG
46[271]	GCAGTCCATCCTACCCCATCTCCTTCCAGTACTGTTGGATCAGGCTTCGCAGTCCT
25[264]	CGGTAGCAAAAGATCCCGCCTACGATACGGGATTTGTCTATTTCGTTCTTATTTGT
27[264]	CGCACACGCGTTGTTGTAAAGTGCTGCAATGAAACCTTAATCAGTGAG
32[271]	TGCTTGCCAAGGGGCATCCTAGTAGGTGCATACCAAAATTAATGTGAATTAGTCTG
13[264]	ACGCGTCAGAAGCGGATTCTATCTGTTCGTGCCAGGCTTCGCAGTCCT
29[264]	CGGTAGCAAAAGATCCCCTACCGGCTCCAGATATCTATGAGTAAACTT
31[264]	CGCACACGCGTTGTTGTCACCTATCCAGCCAGAATCTAAAGTATAAAA
5[280]	AGGCGGACTAGGAAGTCGAAGTAGCGAGTGTAAGCCACTTCGGGAGCC
7[280]	GACCCAGATAGGACTCTTATGAGTCTAGCAAATTGGGTCGGCATCAGT
24[311]	ACTGGTAACAGGAAGATTACGCGCAGAAAAAACGCTATTGAACGGGTC
21[280]	CGGGGTCTGACGCCGTTAGCGGTGGTTTTCTTAGTGGACGGAACACGC
1[280]	AGGCGGACTAGGAAGTAGTGATGACTCCCCTTTCCGGTTCGTCAATCC
3[280]	GACCCAGATAGGACTCGCACACACGCTTAGCGGGAGTGGTTCTGGCCA
19[280]	CGTTAAGGGATTTAGCAGCTCTTGATCCGCGCCGCTATTGAACGGGTC
36[287]	TGGCGGGGAAGGATCTTCACCCAATGAAGCCAGTTACTCAAGTGGACGGAACACGC
11[296]	CCTAGTCCGACTAGCTGGCAGCAATACATAAATAAGACCTTAATTCGC
9[296]	AGCGATCCCGCGATTTTGGTGCGCCTACGTTTGAAATCAGCTAAGAGT
25[296]	CGAACACAGAGAACTCAGCTTAGCAGAGCGAGCTGCGGTAAGACACGACGGGCAAT
27[296]	GCGCCTTCGAGGACTACGCGAGTTCTTGAAGTTCCCTTATCCGGTAAC
32[303]	GGACCTAATACTCGAGCAGATTAAAGCTCGGCTCGGATCGTAAGACCTTAATTCGC
13[296]	CCGCAAGCGTTGTCGTACCCTGCTTAGGACTCGAAATCAGCTAAGAGT
29[296]	CGAACACAGAGAACTCGTTCACTAGAAGAACATTCCACGAACCCCCCG
31[296]	GCGCCTTCGAGGACTACCGCCTCACGCTCTGCGCTGGGCTGTGTGTCA
5[312]	ATTCGTACGGGTAAGCTACCGTAGAACGACACAATTAGCTACGATTTT
7[312]	TTCCGCCAAGGTGCGACCGGAATCTGGTATGCATGCCATGATGGGCAG
42[319]	GTTTCCTCCCCTGGAAGCTCCTAAGATACCAGGCGTTTCCGACTGGAAAAGATCGG
21[312]	CGACCCTGCCGCTCCGTCAGAGGTGGCGATGTGGGCTGAGCCATGAGC
1[312]	ATTCGTACGGGTAAGCTTCAGGCCGGTGACGCGGGGTCCTCACCGCTC
3[312]	TTCCGCCAAGGTGCGAAGCCTCTTTGGTGACGGTCAGCTGTCGGCTAA
19[312]	TTTCTCCCTTCGGACCCCTGACGAGCATCGGCGACTGGAAAAGATCGG
36[319]	CTTTTATGTAGCTCACGCTGTATCCGTTGCTGGCGTTTTTGGGCTGAGCCATGAGC
11[328]	GCCGATTACGTTAAAGTGACCTATTGTTAAGAGGAGCACATTTTTTTT
46[335]	TTATGAAGACTGATGATATTATACGAAGAGGGGGATACAAGTTCTCGATTTTTTTT
25[328]	TTTTTTTTTTTCTATCTATTGAGGTATCCTAACTTAGCGGTAACGTTACCGCTTCG
27[328]	TTTTTTTTTTAGCTTCCTCGGTCGTCGTGCGGTCTTTTCAATTTTTGT

32[335]	GTCTATGGAAGTCGCGTATTCCAAAGGGTAGCGCAGGTGTGGAGCACATTTTTTTT
13[328]	CCCGCGGACAGTTATAGGAAAGGTCAAATACGGTTCTCGATTTTTTTT
29[328]	TTTTTTTTTTCTATCCGCAGGCCAGCAAAAGTAGAGCCATTTGCTGA
31[328]	TTTTTTTTTTAGCTTCGAAGCGCCAAAGGCCGATTGCCAATTTGTACC
	**Square-capture-Z_5**
5[376]	AGGTCGTTAGACACCAACCAAATGAGCTTTTTCAGGGACGGTAGGACA
7[376]	AGGACGTGGTAAGTCTAGCAGCCCCGTAGTGTTTCTAGGGGGACGTCT
24[407]	CGCTCGACGCCAACGAGATAGGGTTGAGTGTTATGTTGGAACCTTGTA
21[376]	GAACGTGGACTCCCGAGTGTAGAATATCACTCGGACTGGGCCCGGACG
1[376]	AGGTCGTTAGACACCACCCCGCAAACACGAACAGCGTTCCCTTCTTTC
3[376]	AGGACGTGGTAAGTCTCCACGTGCAACGCACCTCCGAGTCGGGACAGC
19[376]	CCGTCTATCAGGGATCAGTTTGTACCCGCTTGATGTTGGAACCTTGTA
36[383]	TAATGAGTCCCGGGAGCGGGCAGCCTATGATTGGCTATACGGACTGGGCCCGGACG
11[392]	CTATAGTGCTCTGCCCTAATAAGTGCAAACTACAAGCTATGAAACCGG
46[399]	GAGGTTCGGCACTTGGCATTCACTACTGCTCCATAGGGCTTAAGAGGGTGACTTGA
25[392]	TGTACCCTATAGTAGTGCCCTTGTAATACCAGATAGTCTAGACGCGAGTGACATAA
27[392]	AACGCACGTAAATCATCCTGTTTCGAGTTGAGCAACGGTTGCCATTCT
15[392]	ACGATCTCAATCTATGCTATTGAAAATACCGACAAGCTATGAAACCGG
13[392]	GTCAATTTCGCAACTGCGGATGGATAAGCGGATAAGAGGGTGACTTGA
29[392]	TGTACCCTATAGTAGTATACGACTGCCGAATCAAATACAGATCGGACA
31[392]	AACGCACGTAAATCATTTGGCGCGTCCGGACTGTGAAAAGGATCTAGA
5[408]	TTCTGATCAATTACAGGTAATGTACCAAATACCATGTGTGTTTGCGTC
7[408]	AGGGAGAAAGAACTTACGGAAGTGTGCAAACTCAGCGAGGGTTTGCGA
24[439]	TGACGTGACGAAGGCGCTATAATGGCATCGTAGCCACACGGATCATCA
21[408]	TCAGTGGACAAAGTGACGGGTCGCTAGCGACGGGTATCTTTGGGAAGG
1[408]	TTCTGATCAATTACAGTAGGGGATTTGTATATGAAGCATTACGCCGAT
3[408]	AGGGAGAAAGAACTTAAGCGAGTTCAGTGATCGGTCCCTAATTCCGTG
19[408]	GCAGGGTGTCCGTGTAGTGAAATGCCCAGTACGCCACACGGATCATCA
36[415]	ACTTATCCCATCCTGGTACCTTCGAGTAGCACGCTACACTGGTATCTTTGGGAAGG
11[424]	CTCACGTGGTGGCCCGCCACAATGCTGGCCAGAATGTGAATTAGTCTG
46[431]	AGCGTCAACTCCCTTACACCGGGGTGTTCCAGCGATTCACCAGGCTTCGCAGTCCT
25[424]	CGGTAGCAAAAGATCCGTAGGGTAGTTATCCTCTCTTCGGTAGAGGGGCCGGTCGC
27[424]	CGCACACGCGTTGTTGGCGATCGTCCTTAGTGTTGCAGCCATCGGCTA
32[431]	TGTCACCGAGTGCGAAGATCTTCCAATCCTTCCCTCCAGGAATGTGAATTAGTCTG
13[424]	GCCCATTCAAAACGCATATGCGCTAATAAAACCAGGCTTCGCAGTCCT
29[424]	CGGTAGCAAAAGATCCTAGCTTCCCCGAGGGAAAGAAAAGCTGTGGTA
31[424]	CGCACACGCGTTGTTGGGCACTTTACGGGGGATCCCGGGACCACGGCG
5[440]	AGGCGGACTAGGAAGTGTCGGGGACCCCAGTACACGGGTTTCCTCTAC
7[440]	GACCCAGATAGGACTCCCAATCCCACAACGTAATTCTTAGCTTCGTCG
24[471]	CTCCTCGAAAATGCCATGGGATCTCGGCGACCCGCTATTGAACGGGTC
21[440]	TACTTACTACAATGGTCAGATGATTCAAGGATAGTGGACGGAACACGC
1[440]	AGGCGGACTAGGAAGTCGTGGAGGGTCATGATATCTCCGCACTTAGTC
3[440]	GACCCAGATAGGACTCCCCCCAAGAAGTCGGAGCGACTATTCGACTCG
19[440]	GATTCATTACAAGTGAATAGCGGATGCTGTCCCGCTATTGAACGGGTC
36[447]	CTCTAAAGCAGCTGAAATTGCCGCTCGATCGTCTTATTTGAGTGGACGGAACACGC
11[456]	CCCTACGAGGGCGTGACTACTGCCACGAGAGCTAAGACCTTAATTCGC
9[456]	GGGGTGACTCTTCATATTAGCAACCTGAGAAGGAAATCAGCTAAGAGT
25[456]	CGAACACAGAGAACTCTAAAATATACGCCATGTGTTGATTGACCTTGT

27[456]	GCGCCTTCGAGGACTACACAGTTGTCAAACAATATCTACGCTATGCCT
32[463]	TGAAAAAGGGCCCGTAGCCCCAGTGACGAGACCCCGAATATAAGACCTTAATTCGC
13[456]	GCCTAATAGTCGTCGGGGTTAAAAGGGGACAAGAAATCAGCTAAGAGT
29[456]	CGAACACAGAGAACTCGAAACTCTAGTTCAATTTGGGTCAGTCTCACG
31[456]	GCGCCTTCGAGGACTATTTCATTTTATATGGCGAGCCTTAGGAATCAA
5[472]	ATTCGTACGGGTAAGCTGCCGTCCGCAAACGCCCTACCTTGCGGTTTT
7[472]	TTCCGCCAAGGTGCGAAAGTACGCCAGCCGAGGTACACGCCCGCACCC
22[487]	GACGTATGTCCCGACATCTCAAACCGGCTTAAGACTGGAAAAGATCGG
20[487]	CATCGTGTCCGACCTTACCCTCGTAGTACTCCGGGCTGAGCCATGAGC
1[472]	ATTCGTACGGGTAAGCCAACACTCTAAGAGTAGTACAGCAGATCAACT
3[472]	TTCCGCCAAGGTGCGACTAACTTTGAGCAAAACCTATTACGAGATGAA
18[487]	GGCCAAATACGAAATAATGTTGTCGTCATATAGACTGGAAAAGATCGG
35[480]	TCCTAGCCGTCAGGGACCGTTTCCTTTTTGCTATCAACTTGGGCTGAGCCATGAGC
12[508]	TTTTTTTTAGTCCCTTTCAGACTGTACATATGGACCCGGAGCACATTTTTTTT
10[508]	TTTTTTTTACTCTTGCTTTCCCGCATGAGCAAAGCATGTTCTCGATTTTTTTT
25[488]	TTTTTTTTTTTCTATCATGGTCCGTCCTATTTTTTTT
27[488]	TTTTTTTTTTAGCTTCTGCACTACTCATATTTTTTTT
0[514]	TTTTTTTTTGCGAGGATCGGCCGAGCTGGAGCACATTTTTTTT
14[508]	TTTTTTTTGACGTCAGGTCTGCTTGGCCCGCTATTAGGTTCTCGATTTTTTTT
29[488]	TTTTTTTTTTTCTATCTACTACCAGCTGTTTTTTTTT
31[488]	TTTTTTTTTTAGCTTCGTGCGCGCAAACATTTTTTTT
	**Kagome-capture-Z_1**
5[56]	TACCGGTTGGATCAGTTAAAAGAGTCTGTAACAGGCCGATTAAAGCCT
7[56]	AGGACCGTCAAATGGGGAAGTGTTTTTATTTTGACGCTCAATCGTCTG
24[87]	TTTTGTTAAATCAGTTTGAGGGGACGACGACACGCAGAGATACTGGCT
21[56]	GCTTTCCGGCACCGCAATGGGCGCATCGTATCTTGTTAGCCCAAGTCC
1[56]	TACCGGTTGGATCAGTTCACTTGCCACTCATCTTTTGTCGTCTGCGCG
3[56]	AGGACCGTCAAATGGGTTGTAGCAATACTATACCTCGTTAGAATCCAA
19[56]	AGGCAAAGCGCCAAGATGACCGTAATGGGATGCGCAGAGATACTGGCT
36[63]	TGGATGTTAACTGTTGGGAAGCATCGTCGGATTCTCCAACTTGTTAGCCCAAGTCC
11[72]	AACAATAAAAGGGACAATATACCGCCAGCCAT
46[79]	CCACCAGAGAAACATTGGCAGATTAAAATGGAAATACCTA
25[72]	TGCCCAGACACTCTAGGCCATACCACACGGGACCAGCTCATTTTTTAAATATAAACGTTAATATTTCACAAA
27[72]	AGAGCGTGATGACTTGCCCTTTCCGAAGCATCGTGAAATAATTCGCGTGAGCAGGAAGATTGTA
15[72]	TACGGCACAGACAATAGAACGGGTATTAAACC
13[72]	TGCAACCCTTCTGACCGGCATCGGCCTTGCTG
29[72]	TGCCCAGACACTCTAGGCCATACCACACGGGAGCGCTTTCATCAACATTGAACCCCGGTTGATA
31[72]	AGAGCGTGATGACTTGCCCTTTCCGAAGCATCAGCGCGAATAACAACCGTCAATCATATGTGAG
5[88]	AATGCGGATGGGAGTGTAAAGCATCACCTAATGATAAAACAGAGGCAG
7[88]	GTAATGCCAGTTTCTGCCACGCTGAGAGCTAGACTTTACAAACAATTC
24[119]	GGCAAAGAATTAGAGACAGTCAAATCACCATCTCTACAGAAATCACAG
21[88]	TAATGCCGGAGAGTCATAAAGATTCAAAAATAGGTTGGTAACCCTTCA
1[88]	AATGCGGATGGGAGTGTCAACAGTCATTCCAATTTTTGAATGGCTATT
3[88]	GTAATGCCAGTTTCTGAACCCTCAATCAAGGACATTAAAAATACCTGG
19[88]	TCTACAAAGGCTATTTATTTTAAATGCAAGCCTCTACAGAAATCACAG
36[95]	ATAAATCATCTGGAGCAAACATCGCGCAAGGATAAAAATGGGTTGGTAACCCTTCA
11[104]	GCAAGTTTGAGTAACAATTATAGATTAGAGCC
46[111]	CAGCACCGATTAAATCCTTTGCCCAGTGAGGATTTAGAAG

25[104]	GGCCATCTCTACCGCTGGGATGTAGTAACTGCCGGCAAAATTAAGCAAGCTTAGTAGCATTAACTCATCGGC
27[104]	TCCGTACCGCTTCAAGTATTCAGCGCTAGTGATGTGCTAAATCGGTTGCGGAGCTGAAAAGGTG
32[111]	TGCGCGTAAGTTATTCCTGATTATCAATAATCGGCTGTCT
13[104]	CATAGAAACCACCAGAAAAATCTAAAATATCT
29[104]	GGCCATCTCTACCGCTGGGATGTAGTAACTGCTCACTGTAATACTTTTTGAACCTGTTTAGCTA
31[104]	TCCGTACCGCTTCAAGTATTCAGCGCTAGTGACATCGCCTTATTTCAACAAATGGTCAATATTC
5[120]	GTTCGGTTAGCTCACTTTTTACATCGGGATACTTATTTGCACGTACGT
7[120]	ATCTCGCAGTGTTTGATTAACGTCAGATGTTAATGGAAACAGTACATA
24[151]	AATGACCATAAATTTTTTGCGGATGGCTTAGATTACCACTCACCATTC
21[120]	CATGTTTTAAATATAACTTTAATTGCTCCAGCGGTGGACGTATTATCT
1[120]	GTTCGGTTAGCTCACTCGCAGAGGAAACCAATCAGATGATGGCAATTC
3[120]	ATCTCGCAGTGTTTGACTGATTGCTTTGAGATTAATGGAAGGGTTTCA
19[120]	CTGGAAGTTTCATACTGCAAACTCCAACACAATTACCACTCACCATTC
36[127]	GAGTGAGCCCAATTCTGCGAATTTTAATTCGAGCTTCGGAGGTGGACGTATTATCT
11[136]	TCATATTAATTAATTTAAAAGAAAACAAAATT
9[136]	GAAGAGTGAATAACCTAATATTTGAATTACCT
25[136]	ACTACCAGAGTAACGACGCGACGTACGTGCCCTCACAAAAATCAGGTCCAGCCCCCTCAAATGCCCGATTGA
27[136]	TGGCGCCTTTATTCCATGGCGCGTCGGCTCATGAGTCAGAAGCAAAGCATATACTGCGGAATCG
32[143]	CGCTAGGGATCAGAGTCAATAGTGCCATCCTAATTTACGA
13[136]	TCTACATAGCGATAGCATCCTGAGCAAAAGAA
29[136]	ACTACCAGAGTAACGACGCGACGTACGTGCCCCCGAAGAGGAAGCCCGTACATAGTAAAATGTT
31[136]	TGGCGCCTTTATTCCATGGCGCGTCGGCTCATCCTGCTCCTCGCGTTTGCCAGAGGGGGTACAG
5[152]	TAAGCCGTCCGAGTCGATGGTTTGAAATAGGCAAATCCAATCGCATTC
7[152]	AAATTGAAGACACAGGTTTAGTTAATTTCCCAACTTTTTCAAATATAT
42[159]	ATACATACTTTAGGAATACCAGTAGATTCATCAGTTGAGATCGCCATCCTGTAAAC
21[152]	CATAACGCCAAAAGCAAACTAACGGAACAACCAACCTAGTCGCAACAG
1[152]	TAAGCCGTCCGAGTCGATTACTAGATAATATCAATTTATCAAAATCAT
3[152]	AAATTGAAGACACAGGGGCGTTAAATAAGCTTTGGGTTATATAACCTT
19[152]	AGAGCAACACTATACGCGTTGGGAAGAAATTGTCGCCATCCTGTAAAC
36[159]	CCCCAGCAACGACGATAAAAAGCTTTTTAAGAACTGGGACAACCTAGTCGCAACAG
11[168]	TGATATGGGCTGCGATTCTCAACGCTCAACAG
46[175]	AGCAATAGGAGCACTGAAGCTCAGGAAGCCATATTTAACA
25[168]	TTTTTTTTATCAACCCTAGGAATAAGGCTTGCTACGCGCGCTGAGGTCCCAGAAGG
27[168]	TTTTTTTTCGCAGTTGAAAACGAGTAGTAAATCGCATGAAAACTAGGT
32[175]	AGAAAGGAAGGATAGATATGGCCTATAGATAAGTCCTGAA
13[168]	TTACAGTGGGAAGTCGCAGATGCGTTATACAA
29[168]	TTTTTTTTATCAACCCTCATTTCAACTTTAATTGCCGAGGAATTATAT
31[168]	TTTTTTTTCGCAGTTGCAGACGGTTTATGCGAAGGCGGCTAGGTGGAC
	**Kagome-capture-Z_3**
5[216]	TACCGGTTGGATCAGTAAGGTGAAGACCTTAACTCTACGAGGGGTAGT
7[216]	AGGACCGTCAAATGGGCGGAACATCCGGCGGCCGCTCTGGCATATCAC
24[247]	TCAACCAAGTCATCATCTTTTACTTTCACCAGCGCAGAGATACTGGCT
21[216]	ATGCCGCAAAAAATCTACCCACTCGTGCAGGCTTGTTAGCCCAAGTCC
1[216]	TACCGGTTGGATCAGTTCGGGTCCTAGTATACCGATTACATGCTGACG
3[216]	AGGACCGTCAAATGGGTGACCGACTGACAAATCTGAGTCGATAAAAAC
19[216]	AAATGTTGAATACTATATCTTACCGCTGTCTCCGCAGAGATACTGGCT
36[223]	TAAATCAGATATTATTGAAGCGATGAAAACGTTCTTCATCTTGTTAGCCCAAGTCC
11[232]	CTCGTTAGGCTCGAAAATCTAAACACTACCTG

46[239]	TGGTGAACGCTGAGTACATCAAACGCCGACTAATCCGGGG
25[232]	TGCCCAGACACTCTAGGCCATACCACACGGGACAGTCTGAGAATAGTGTGGCATCCGTAAGATGGACTAAGC
27[232]	AGAGCGTGATGACTTGCCCTTTCCGAAGCATCGATTCTTGCCCGGCGTTTCGGCAGCACTGCAT
15[232]	GAGCTCGGGGATCCAAGGGACTTTAGACCACC
13[232]	TACTCTTACGTATGCCGTCGCACAACCACAAA
29[232]	TGCCCAGACACTCTAGGCCATACCACACGGGATCAGCCACATAGCAGATACAAGTTGGCCGCAG
31[232]	AGAGCGTGATGACTTGCCCTTTCCGAAGCATCAGTCCGTTCATCATTGCGTTGTCAGAAGTATG
5[248]	AATGCGGATGGGAGTGCAACTGTCCATTCATACGTGGATACGAGCTAA
7[248]	GTAATGCCAGTTTCTGAATGTCGCGGTATCGTGTCCGTATCGGCGTAC
24[279]	GTCGTGTAGATAACAGTTAATAGTTTGCGCAATCTACAGAAATCACAG
21[248]	CACGCTCGTCGTTCCTTTGCCGGGAAGCTGCGGGTTGGTAACCCTTCA
1[248]	AATGCGGATGGGAGTGAGTCATTTTGGCTCATATATGGTGTGATCCGC
3[248]	GTAATGCCAGTTTCTGTTGAAGCAACCGCACAATGCAATCTGACCGCG
19[248]	TCCGGTTCCCAACCTGACTTTATCCGCCTTACTCTACAGAAATCACAG
36[255]	AGCACTAAATCCCCCATGTTGATCCCGGAAGGGCCGATTAGGTTGGTAACCCTTCA
11[264]	TTGACAGGATGTAACAAGGGCGTTATCATGTG
46[271]	GCAGTCCATCCTACCCCATCTCCTTCCAGTACTGTTGGAT
25[264]	GGCCATCTCTACCGCTGGGATGTAGTAACTGCCGCCTACGATACGGGATTTGTCTATTTCGTTCTTATTTGT
27[264]	TCCGTACCGCTTCAAGTATTCAGCGCTAGTGATAAAGTGCTGCAATGAAACCTTAATCAGTGAG
32[271]	TGCTTGCCAAGGGGCATCCTAGTAGGTGCATACCAAAATT
13[264]	ACGCGTCAGAAGCGGATTCTATCTGTTCGTGC
29[264]	GGCCATCTCTACCGCTGGGATGTAGTAACTGCCCTACCGGCTCCAGATATCTATGAGTAAACTT
31[264]	TCCGTACCGCTTCAAGTATTCAGCGCTAGTGATCACCTATCCAGCCAGAATCTAAAGTATAAAA
5[280]	GTTCGGTTAGCTCACTCGAAGTAGCGAGTGTAAGCCACTTCGGGAGCC
7[280]	ATCTCGCAGTGTTTGATTATGAGTCTAGCAAATTGGGTCGGCATCAGT
24[311]	ACTGGTAACAGGAAGATTACGCGCAGAAAAAATTACCACTCACCATTC
21[280]	CGGGGTCTGACGCCGTTAGCGGTGGTTTTCTTGGTGGACGTATTATCT
1[280]	GTTCGGTTAGCTCACTAGTGATGACTCCCCTTTCCGGTTCGTCAATCC
3[280]	ATCTCGCAGTGTTTGAGCACACACGCTTAGCGGGAGTGGTTCTGGCCA
19[280]	CGTTAAGGGATTTAGCAGCTCTTGATCCGCGCTTACCACTCACCATTC
36[287]	TGGCGGGGAAGGATCTTCACCCAATGAAGCCAGTTACTCAGGTGGACGTATTATCT
11[296]	CCTAGTCCGACTAGCTGGCAGCAATACATAAA
9[296]	AGCGATCCCGCGATTTTGGTGCGCCTACGTTT
25[296]	ACTACCAGAGTAACGACGCGACGTACGTGCCCAGCTTAGCAGAGCGAGCTGCGGTAAGACACGACGGGCAAT
27[296]	TGGCGCCTTTATTCCATGGCGCGTCGGCTCATCGCGAGTTCTTGAAGTTCCCTTATCCGGTAAC
32[303]	GGACCTAATACTCGAGCAGATTAAAGCTCGGCTCGGATCG
13[296]	CCGCAAGCGTTGTCGTACCCTGCTTAGGACTC
29[296]	ACTACCAGAGTAACGACGCGACGTACGTGCCCGTTCACTAGAAGAACATTCCACGAACCCCCCG
31[296]	TGGCGCCTTTATTCCATGGCGCGTCGGCTCATCCGCCTCACGCTCTGCGCTGGGCTGTGTGTCA
5[312]	TAAGCCGTCCGAGTCGTACCGTAGAACGACACAATTAGCTACGATTTT
7[312]	AAATTGAAGACACAGGCCGGAATCTGGTATGCATGCCATGATGGGCAG
42[319]	GTTTCCTCCCCTGGAAGCTCCTAAGATACCAGGCGTTTCCTCGCCATCCTGTAAAC
21[312]	CGACCCTGCCGCTCCGTCAGAGGTGGCGATGTAACCTAGTCGCAACAG
1[312]	TAAGCCGTCCGAGTCGTTCAGGCCGGTGACGCGGGGTCCTCACCGCTC
3[312]	AAATTGAAGACACAGGAGCCTCTTTGGTGACGGTCAGCTGTCGGCTAA
19[312]	TTTCTCCCTTCGGACCCCTGACGAGCATCGGCTCGCCATCCTGTAAAC
36[319]	CTTTTATGTAGCTCACGCTGTATCCGTTGCTGGCGTTTTTAACCTAGTCGCAACAG
11[328]	GCCGATTACGTTAAAGTGACCTATTGTTAAGA

46[335]	TTATGAAGACTGATGATATTATACGAAGAGGGGGATACAA
25[328]	TTTTTTTTATCAACCCTATTGAGGTATCCTAACTTAGCGGTAACGTTACCGCTTCG
27[328]	TTTTTTTTCGCAGTTGCTCGGTCGTCGTGCGGTCTTTTCAATTTTTGT
32[335]	GTCTATGGAAGTCGCGTATTCCAAAGGGTAGCGCAGGTGT
13[328]	CCCGCGGACAGTTATAGGAAAGGTCAAATACG
29[328]	TTTTTTTTATCAACCCCGCAGGCCAGCAAAAGTAGAGCCATTTGCTGA
31[328]	TTTTTTTTCGCAGTTGGAAGCGCCAAAGGCCGATTGCCAATTTGTACC

Kagome-capture-Z_5

5[376]	TACCGGTTGGATCAGTACCAAATGAGCTTTTTCAGGGACGGTAGGACA
7[376]	AGGACCGTCAAATGGGAGCAGCCCCGTAGTGTTTCTAGGGGGACGTCT
24[407]	CGCTCGACGCCAACGAGATAGGGTTGAGTGTTCGCAGAGATACTGGCT
21[376]	GAACGTGGACTCCCGAGTGTAGAATATCACTCTTGTTAGCCCAAGTCC
1[376]	TACCGGTTGGATCAGTCCCCGCAAACACGAACAGCGTTCCCTTCTTTC
3[276]	AGGACCGTCAAATGGGCCACGTGCAACGCACCTCCGAGTCGGGACAGC
19[376]	CCGTCTATCAGGGATCAGTTTGTACCCGCTTGCGCAGAGATACTGGCT
36[383]	TAATGAGTCCCGGGAGCGGGCAGCCTATGATTGGCTATACTTGTTAGCCCAAGTCC
11[392]	CTATAGTGCTCTGCCCTAATAAGTGCAAACTA
46[399]	GAGGTTCGGCACTTGGCATTCACTACTGCTCCATAGGGCT
25[392]	TGCCCAGACACTCTAGGCCATACCACACGGGAGCCCTTGTAATACCAGATAGTCTAGACGCGAGTGACATAA
27[392]	AGAGCGTGATGACTTGCCCTTTCCGAAGCATCCCTGTTTCGAGTTGAGCAACGGTTGCCATTCT
15[392]	ACGATCTCAATCTATGCTATTGAAAATACCGA
13[392]	GTCAATTTCGCAACTGCGGATGGATAAGCGGA
29[392]	TGCCCAGACACTCTAGGCCATACCACACGGGAATACGACTGCCGAATCAAATACAGATCGGACA
31[392]	AGAGCGTGATGACTTGCCCTTTCCGAAGCATCTTGGCGCGTCCGGACTGTGAAAAGGATCTAGA
5[408]	AATGCGGATGGGAGTGGTAATGTACCAAATACCATGTGTGTTTGCGTC
7[408]	GTAATGCCAGTTTCTGCGGAAGTGTGCAAACTCAGCGAGGGTTTGCGA
24[439]	TGACGTGACGAAGGCGCTATAATGGCATCGTATCTACAGAAATCACAG
21[408]	TCAGTGGACAAAGTGACGGGTCGCTAGCGACGGGTTGGTAACCCTTCA
1[408]	AATGCGGATGGGAGTGTAGGGGATTTGTATATGAAGCATTACGCCGAT
3[408]	GTAATGCCAGTTTCTGAGCGAGTTCAGTGATCGGTCCCTAATTCCGTG
19[408]	GCAGGGTGTCCGTGTAGTGAAATGCCCAGTACTCTACAGAAATCACAG
36[415]	ACTTATCCCATCCTGGTACCTTCGAGTAGCACGCTACACTGGTTGGTAACCCTTCA
11[424]	CTCACGTGGTGGCCCGCCACAATGCTGGCCAG
46[431]	AGCGTCAACTCCCTTACACCGGGGTGTTCCAGCGATTCAC
25[424]	GGCCATCTCTACCGCTGGGATGTAGTAACTGCGTAGGGTAGTTATCCTCTCTTCGGTAGAGGGGCCGGTCGC
27[424]	TCCGTACCGCTTCAAGTATTCAGCGCTAGTGAGCGATCGTCCTTAGTGTTGCAGCCATCGGCTA
32[431]	TGTCACCGAGTGCGAAGATCTTCCAATCCTTCCCTCCAGG
13[424]	GCCCATTCAAAACGCATATGCGCTAATAAAAC
29[424]	GGCCATCTCTACCGCTGGGATGTAGTAACTGCTAGCTTCCCCGAGGGAAAGAAAAGCTGTGGTA
31[424]	TCCGTACCGCTTCAAGTATTCAGCGCTAGTGAGGCACTTTACGGGGGATCCCGGGACCACGGCG
5[440]	GTTCGGTTAGCTCACTGTCGGGGACCCCAGTACACGGGTTTCCTCTAC
7[440]	ATCTCGCAGTGTTTGACCAATCCCACAACGTAATTCTTAGCTTCGTCG
24[471]	CTCCTCGAAAATGCCATGGGATCTCGGCGACCTTACCACTCACCATTC
21[440]	TACTTACTACAATGGTCAGATGATTCAAGGATGGTGGACGTATTATCT
1[440]	GTTCGGTTAGCTCACTCGTGGAGGGTCATGATATCTCCGCACTTAGTC
3[440]	ATCTCGCAGTGTTTGACCCCCAAGAAGTCGGAGCGACTATTCGACTCG
19[440]	GATTCATTACAAGTGAATAGCGGATGCTGTCCTTACCACTCACCATTC
36[447]	CTCTAAAGCAGCTGAAATTGCCGCTCGATCGTCTTATTTGGGTGGACGTATTATCT

11[456]	CCCTACGAGGGCGTGACTACTGCCACGAGAGC
9[456]	GGGGTGACTCTTCATATTAGCAACCTGAGAAG
25[456]	ACTACCAGAGTAACGACGCGACGTACGTGCCCTAAAATATACGCCATGTGTTGATTGACCTTGT
27[456]	TGGCGCCTTTATTCCATGGCGCGTCGGCTCATCACAGTTGTCAAACAATATCTACGCTATGCCT
32[463]	TGAAAAAGGGCCCGTAGCCCCAGTGACGAGACCCCGAATA
13[456]	GCCTAATAGTCGTCGGGGTTAAAAGGGGACAA
29[456]	ACTACCAGAGTAACGACGCGACGTACGTGCCCGAAACTCTAGTTCAATTTGGGTCAGTCTCACG
31[456]	TGGCGCCTTTATTCCATGGCGCGTCGGCTCATTTTCATTTTATATGGCGAGCCTTAGGAATCAA
5[472]	TAAGCCGTCCGAGTCGTGCCGTCCGCAAACGCCCTACCTTGCGGTTTT
7[472]	AAATTGAAGACACAGGAAGTACGCCAGCCGAGGTACACGCCCGCACCC
22[487]	GACGTATGTCCCGACATCTCAAACCGGCTTAATCGCCATCCTGTAAAC
20[487]	CATCGTGTCCGACCTTACCCTCGTAGTACTCCAACCTAGTCGCAACAG
1[472]	TAAGCCGTCCGAGTCGCAACACTCTAAGAGTAGTACAGCAGATCAACT
3[472]	AAATTGAAGACACAGGCTAACTTTGAGCAAAACCTATTACGAGATGAA
18[487]	GGCCAAATACGAAATAATGTTGTCGTCATATATCGCCATCCTGTAAAC
35[480]	TCCTAGCCGTCAGGGACCGTTTCCTTTTTGCTATCAACTTAACCTAGTCGCAACAG
12[508]	TTTTTTTTAGTCCCTTTCAGACTGTACATATGGACCC
10[508]	TTTTTTTTACTCTTGCTTTCCCGCATGAGCAAAGCAT
25[488]	TTTTTTTTATCAACCCATGGTCCGTCCTATTTTTTTT
27[488]	TTTTTTTTCGCAGTTGTGCACTACTCATATTTTTTTT
0[514]	TTTTTTTTTGCGAGGATCGGCCGAGCT
14[508]	TTTTTTTTGACGTCAGGTCTGCTTGGCCCGCTATTAG
29[488]	TTTTTTTTATCAACCCTACTACCAGCTGTTTTTTTTT
31[488]	TTTTTTTTCGCAGTTGGTGCGCGCAAACATTTTTTTT

Supplementary Table 2 | DNA sequences of seed-H (see Supplementary Fig. 5 for the strand diagram)

Colored sequences represent captures, 18 nt

Start	Sequences
	**Core Strands**
39[428]	GAAGCGAGTTAATTTGCCAGCTTCGGCCTCGTTTGGCACCTTTTTTT
17[140]	TCAGAAGCGGGGAGCAAGCAAATCAGGCAGCCTAATCAATATC
21[371]	GCTCCTTTATTTCTCACGTCTCTTATGGTCTTGTTGCACAGGGC
11[77]	ATTTCACATAAAAAATTAATCTAGCTGATAAA
45[189]	TGAATCTGCTAGGTGACGTTGTATGCGCGAACTGATCAATAG
40[363]	TAGACAGAAACCGTCTATCAATATACTAGGGTACTCTCAAATG
52[160]	AAGTCAGAGCGGAATAGGTGTATCACCGTACG
11[130]	GTTCACAACCTCAAGAGAAGGATTCCGGTATT
12[461]	TCCATTTCGTGGCCTTTTGGGTCTGAAGGGACGGTCGG
50[265]	AAAATAGGCTACTGACCGACTGACCCTGTTCG
17[182]	TGGTCAGAAGATTAGACTTACCAATTACCTGAGCACTGATTATC
14[473]	TTTTTTTAACTTCTAACCGTAGCCCCAGTGTACATGAAAGGCTCTTTC
30[468]	CTGACCGCCTCAGCTGGCGTTCGCCGAACCGATTTTTTTT
39[84]	TTTTTTACCTCGACATTTCGCAAATGGCTGCGGAAAGAATACA
21[140]	GTTTATAAATATAATTATCAGTAGCCAGCAAAATCATTGTATCG
22[272]	TGATCATTGGAAAACGGTCTGTCATGTATATCCGTGCGTAGAA
18[494]	TTTTTTTTCTCGCGCCGTGTCTGGCCGATTTTCGAATGTT
21[392]	CTGCGAGTAGGCTAGTTGTCCCAAAATGCGATATGGTAAATA
26[188]	CTCATGGATTAGTCTTTAAGCAATACGAGTAAAGCACAAACG
51[378]	CGATAGCTCATCACCTTCCGGCTTAACTCCTCGAAAAGCCCAGA
26[419]	GGCTAATTTTTCTTCCGTGAGCAGACTGAGTGAGGTCAAGCGCA
43[252]	CTCTTTTGTTCCAGCTAAGGAGCTTGCAGTCTCGATAAATGT
22[314]	CTTTGCTGAACCTACTCTACGGGGCCCCGTTCAGCCCGATAAAT
14[132]	GAGGTGATCACCGCGACGAAAGAAAAGGCCGCTTTCAGCTGAATAAAGA
25[98]	CCGGATGCTGAATCGCATAACCGATATGGGTAGCCATTTGGGAA
22[412]	AAGTCAACTGTCAGGCTTAACTATAAACCGACACGAAATGTCAAACAAAT
13[107]	ATTCGGGATCGTTCAACGGTGTTCAACGCAAGGAATAAGAGGTC
26[440]	TCAGGTCTGACGCAGTTTCGCAGACACCTCGACTCACGGCGACG
31[224]	GGACTGGCCTGTACGAAAACGGATCTAGGGGTAAGGGCGCTAG
51[273]	AGGTGAGATCCTTTGGTGCAATAAAGTCAATACGGGAGGGGTCG
37[168]	ACCAAACCTAAATGAACGAACCACCAGCTGTCCATAAAGGGATT
42[160]	CCGCAAGAAAATAGGAACCCATGTCCACCCTT
32[471]	TTTTTACGGGTATTTCGAACACCCCCAAGGAGGG
37[252]	ATAAAGGCGAACCTGAGAATAGTGTAGCCGAGCGTTCGGGAAG
31[448]	GAGGGCGTGACCTGGTACGTCAACGTCGGGGACCGGCTGCATGCTTGGCT
49[105]	GTCTGATTATTACGAAATCGTCAGTTTGACCATTAGTAATTCGC
31[266]	GGCGCGCACTGCATTAATTGTTGCCGGTTTCCCCATCTTAAAG
1[130]	TGCGTCATACATGGCTTTTGATGCAAGATTAG
49[336]	CGACCGACAAAGCAGTGCAGCATGCTCCACCGACCTTGGGAAGT
20[132]	TTACAGCTTTCAGGTCTTTACCCAAGAGGAAGCGGGGAGAGGCGGTTTTT
5[130]	CGCAGTCTGAGTAACAGTGCCCGACTTGCGGC
41[63]	TTTTTCTGCAAGGCGATTCTCAAAAGGGGGATGTGTTTTT
37[294]	CGTGGTGAAGGCACGATTACATGCCCGATTATCACCGAAAGAG
40[405]	AGCGTTAGCCTACACACTGCTACCGCATCACTAGCGTCTTGAA

45[441]	ATTGACCACAACGCCACAAGTATCTGTTGAAGTACGCTTCGTTT
3[130]	ATACCGTTGGTAATAAGTTTTAATTGAAGCTT
37[336]	CGAGAGAGCCAAATGAATCTAGACGACATCCCGGTACAAATGT
34[471]	TTTTTCAAGAACTGAAATATCCTCGTGGATCACG
40[447]	GACTTAGACTAGGCCTTCTACGTGACGTGTCGGATGCGCGGAC
48[422]	TATACTGGGAAACTCATACTATGAACAAACAC
23[140]	CCGCCCAGTACCCATCGTAGGAATCAGAGAATAACCTTGCTGA
47[168]	CCAGAGCTATTAAGGCCTTAATGGTTCCTAATTTACGAAAGTTA
12[244]	TATAGACTCAAGAGGTCTAGAGCTCTGAATTACGGGTACTTATAA
29[63]	TTTTTCCAGGGTTTTCCCATGAGTACGAGCTGAA
10[179]	AAATCAACGAAAATAATATAGAAGGCTTATAGGATTCGATTGGCCTTGATATTT
21[161]	CATTCATAAAGCGTTGATACAATAAGGCAGAGGCATTAGGGCGA
13[336]	ACTGCCGACTCGAACTATGTGAACGTTCTCTACTAAACACCGCC
45[231]	ACAATGAACGGTACGTAACGGTTACTCTAGGGAGGCAACATCAA
12[494]	TTTTTTTCATCAATCTGTGCGGGCAAGAAAGGC
20[181]	GAAAAAGCCTCAACGCTCAGCGCCAAAGACATGACGGACCAAAAGGAGCCT
25[140]	TTAGAGCGACAGCAACATGTAATTTAACAACATGTCTGAAATGGATTATA
22[461]	TAGTTTGTCACCGACGGCAAAGAACGAGCAGAGGCAAACCGAGTGATGGTC
45[210]	ATGAGACTAATCGTCACCGCGTCGCTTTCCTCGTTACCATTCGG
52[265]	AGCTCATTTGACTATGCTTGCCTTATCGGTTG
9[235]	TCTTGGAATTAGCTGGGGGAGTGCCCAACTAC
12[97]	CTTTAATTGGTTGCGCTGCCTAATGAGTGTTTTT
18[104]	AATAGCAAACTCCAACACCGCTTTGGAAGCATAAAGTTTTTT
49[126]	CCAAAAACCGCCGCCCCACGGAGTGAACGAAAGAGGCATAAAAA
14[160]	ATTGAGGGAGGGAATTGCAGCTAATAGCAGATTAG
11[235]	TGATCGGCCGAGCTTATCTAGTTAACCCACAC
44[107]	TGTTAAATCAGCTAAAAACAGCTTCTGGTGCC
20[473]	TTTTTTTTTAAGGATAATAGTCGTCGGCCCTAATAGCGCTT
42[265]	AGTTGAGTGGATCCCTCAGACTCTATTACGCA
50[317]	CGTAATGCATAGGGAGCACACAATGATACCGC
20[223]	GACTCCAACCAAATCAAGAAGAACTCAAACTCATTGCATACTA
25[189]	AGAATAAAGGGAGCCCCCGAATGCGCCCAAGCCACTTCGGGGTCTAGCATC
26[473]	TTTTTTTCTCGGGCCTCCGAGCCCATCATCTTTTTTTT
7[235]	ACAGGAGTGACTAGTGTACAGGTAGCATCTTA
22[494]	TTTTTTTACTTAGGTTCACTGCGGTCCCTTACA
47[399]	TCGTCGTCTGCCGTATAACCCAATCTGTCTTAGGCAATGTTG
24[104]	GCTGAATCGGTTCAAAGGGAAACCTCACAATTCCACATTTTT
2[258]	CTTGTTGCTCCCCGAGCCTCGCCATACAAGAATGCTCCAAGAATAAACCG
31[308]	GACTGTTTTGTAGGACGCGACCCTACTCATTAGCGTCGATGCAC
51[294]	TGCTGGTAATAACGGCGTATTCCTCTCAGTTCGGTGCCCCAG
13[357]	GGACGCTTGTGGACACGATTCAACAAACCAAGTCGAGGGTAT
3[235]	ACGCAACAGCATCTATAGCTCGTGGGTGAGCG
26[209]	GAGGTGCCGCACGTATAACGTTGCTGGCGAGAACAAGAGGGGTC
35[84]	TTTTTGTACCGATTCCATATAACAGTTATCCCCCACGGGTAAA
43[294]	TAATCCCCTTCGAGAGACGGGAGCTGGAAGCTCCCCAATGCT
43[273]	CAAGATGACAGTTACTCTCCAGTCTATAATTCTCTTATTTGGAA
24[146]	AGCTCCAAAACTTGAGCAACGGCTCCATAAATCGCCCAATAAT
26[230]	CAGTTTGGTGGCCTATGATCACTAAGTAGTCTTACTCTGTGTTC

31[350]	GTGGCGTTCCTAACGCTGCGCGTAACTGACGCGGTTCTGGTGTGGCACAG
27[84]	TTTTTTTCGAGCCCAACGCGCCCGAAAACAACCATC
17[287]	CCGGTAGAAGATAGCTAGGCGGCTAGGTTCCACCGAGAGCGCT
49[357]	CGCGGAACACGGCTTCAATTCCGTATCTTGGTCATTATCCCC
15[231]	ATCAATCACAGTGTAGCGGTCGAGCCGTATGTTCCTTATG
17[243]	ACGGACCCCATTGTCTTACCGCTGTTGAGATTTCCCTGAGTCCAAC
35[336]	AGCATGCAATAGCCTCTGCAAATCTAACGCTAGGGGCCCTACG
35[126]	ATACGCAACTAAGCCCCCTTATTAGCGGACACCAAAAATAATA
37[378]	GATTGATTGGATCCATTGCCAATTTGTGTATTCCGAATCGCCTTATGATTCT
21[182]	ATATGCGCCAGCATACGAAAGCGTTTACATTGGCATAGTATC
41[119]	CCCAGCTGGCTCAACAACTTCAGAACCAGGGTTGAAATACATT
27[119]	AAAAAGATACCGTCACCGAAAAAGGCTAATTATTAT
25[239]	TGCCTCAAGGATTGCAAAGAAGGCGAGTTACATTCATGGTAAAAAGGCCG
52[317]	CAGAGCTAAATTCATGCGGCGATTTTGGTCAT
37[428]	GCTCATCTGACACAAAGCTCGGATGAGTGACTATACTTAGTTTTTTT
15[84]	TTTTTTCTTTTCACAAAAACATTCTCGCC
38[335]	CATGCGCATAGATATGGCCTGATACTATTTTGGGGCAACGCCAAC
41[161]	AAACCTTAGACGTTCAAATACTGCAACATTTAACAGCAAGTAT
19[140]	TTACCCACCGTACGAGCCAGTAATAAGACGACGTCACCAGTCA
47[189]	GACTAGAAAATTAAATTCACGCACCATTAAAAATATCTGAGA
27[154]	ATTTAGGTTTACCAACAGTATTTTGACTCATAATACAGGAATA
25[287]	CGTTGAAAGATATGATTAGACGTATAGAGCTAAAATAATTGACA
13[126]	AGCAGAACGTCACCAACTCCAGTAAGGCTGACCTTCACACCCTC
26[335]	GCGCAGGCCATAACTTTGCGTGCAATCGTTGAAAGGGTGTAACTTTATTGA
21[413]	ACGAAGCCTGCAGGTTCCTGAGTTCTCAGATCCGAGCCCACTCT
40[494]	TTTTTTTCCTCGCGCGTCAGTATAGAGTGCCTG
48[475]	TCAAACCACGGACAAGTTGGACACAACTGATG
26[251]	GTTAGCAACAGCACCGTTAAAAGCGAGTTTCAGCTCCTGGGT
45[252]	GGCGGGATAGGGAATGAGGGAAGCGTCGAAATTATAGGGGTT
11[287]	GCAGAAAAAAAGATCGCACCTCACGGAAGGTA
29[140]	GAGCCTAAGTGCTCGAGAACAAGCAAAACAGGGGCCAGCAGCA
7[287]	CAAACAAACCACCTAGGTTAGAGGAGGGGATG
24[195]	CTACGTGGCACCGGAAGCTCAATCGTTCAGCTTCATATACAACGCAATCA
3[287]	TGCGCTCTGCTGCGCTGAGGTAAGGTTACACA
20[265]	GCTGCGCGTACGTCGTAAGACGTCACAGGGTATAGGGTACGTG
5[235]	GGTGCAGCATAAGATAAACGAGGTCACCAGCT
26[104]	CAAATATCAACTAAAGTACGGCTTTAAAGAACAATGACAGACTT
12[286]	GCATCACAGTCTCTTTCTACAGAGTGAATACTCATACATCGGATCCCGCG
35[168]	TCCCATTGAAATAGCCCTAAAACATCGAATTAACATCAGAGCGG
24[405]	ACGCTTGCAACGAGTCATAGCTGAATGAGGTCACCACATCGTA
43[91]	AAATATTATTCATGGTCATAGTCGTGCCAGCTGCA
17[371]	CCCATCTTGGGCCCTTAATCTGTGATCATATGTGGCTTTGTCT
5[287]	GAAAAAGAGTTGGGTCTCCCGCAACCCGCGCG
41[203]	ATAGTCGCGCACATGTGAATATAAGAATTTCAACGCGAGAAAAC
19[63]	TTTTTGTAAAGCCTGGGGTCACTGCGGTCAGGAT
20[307]	GAGCGGATGTCGTGCGTAGTTCGCCAGTTAACACGCTCGTCAC
43[316]	TAGGTACGGGCACCAACAGGTTTTGCCGTTGAGAGGGCTGTTTT
31[399]	CCTTTTTGTCCAGCTCGTAGAAAATCTATCGCTCAAATTCACACGATAAC

51[316]	CCACGAAAGTAGCGTTAATCGGGTCAGAGGGGCACGGGGAAGCG
13[378]	CATAATCCCTTCCTAGTTCCTCTCGGGGATCTCGGCGCATGAGC
27[196]	AATCGCTGAGTAGTTTTTGTCTAGCTACAGGCACTTGCGAACC
29[182]	AATGAACAAAATGTGTCCGTATCGGCAGCGGAGCTACCTCGCC
51[83]	GCCTCTTCATATGGCTATGCTTGTTTTTT
9[287]	TTTTTTGTTTGCGGAAAACAGTACTCGAACAT
49[147]	TTAGCAACCCACACTGAACATATGAACCAGAGCCACTCATAG
45[273]	TCCAAATGAGTAACTGCACTTTATCAAGATGCTTTTCCGTGGAC
29[224]	TGTGGACGCCCGAATAACCTAAACGCGGCACTATCTTTAAAAG
49[378]	TACTCCTGGCTTTGTAGATCCCGACACCTTGTATGTGACGCGGA
2[300]	CGAAGCCAGTTACCAAGAATCCTTGTTGCCATTGC
14[202]	TCCAGAACAATATTTCCTGTTAAAACCCTTCCAGT
47[420]	AATCTGGATTTAGTATGTTGCGCTTGCCCTGGTGCCTAGGGTCA
13[147]	CATCGCCGACAAAATTCTTAAATATACAGGAGTGTATGAAAC
42[317]	TGCACACGGGTGACCAAGTGAGTGAGGCACCT
17[413]	AGCGCTTTTGTTTTACATATGGGGCCGCAGTAGGTATCTTT
21[203]	GCTGGCCATCACCGTCCCCGCGCTTATTTAGAGCTTGCGGCCTT
19[182]	CACGACTGACCTGGGGAAAGCCGGCGCACACCCGAGCCATTAG
50[422]	GTTAAAGCGGTGTATTTGCGGTGATAGTCACA
19[98]	TAGAGTGGCTTAGGCTTGCAGGGAGTTCAGCATCAGTAGCACCA
16[97]	ATCCAATAATCTGACCAGTGCCAAGCTTTTTTTT
43[105]	AAACGACCACATTCAACAGTTCAACATGTTTTAAATTAAATTGT
17[453]	CACGGGCCACTCTAATGGGGTGCAAGACCCAGTGTTTTTTTTT
26[272]	GGCTTCACATGCCATCCGTCGCCTCCAGTAAGGCCAGCAGGTAT
35[210]	GAGCTGGATTATCACGCTTACGACGGTGTACTGCACGAAAGCA
16[132]	GGTAATCATATTGTGTCGAAATCGCTATTTTTTAAC
21[434]	CGCGTAGATCAATTCTTCACACATAACAGTCCCTTTCCCGGC
20[349]	GATACCCCAATTCCGACCGGCTCGGATCGCTATGGGACTGAACC
35[252]	ATTGTGAAAGGATGACTGGTGAGTACGTCCTGCATTACCGGAT
44[265]	AAAGACCGAAATAATAGCTTCTCGTCGCTTCT
16[174]	CTCTTACAGATTACCGCGCCCAATTTTTGCTAGCATTGACAGGA
42[422]	ACAAGTGGAGATTTCAAACTTATCAACGGGCG
5[336]	TGTTATCTGCATTTAACCATACTAACTCTC
3[392]	TTATAATACATCAAGTCTACATCACAATACCC
46[107]	GAACGCCATCAAAATCAGAAGCCATTCGCCAT
47[231]	TCGAATGATCTAGTACCCGGCCCGTATGGCGTTTCTGGTTAGGC
0[216]	GTTTTGCACGTAAAAGAAAGCGACACAACGTGGCGAGATGATGGTGTCTTCT
27[224]	AAAGATGAGTGTAGCGAATCTATTGTTCATCTACGGTTCCCTG
29[266]	TGCTCTAAGCGTCTGTGTGCACGAACCTCTGACGCCCCAAGTT
53[83]	ACGGCGAACTAGCACAACCCAACAAGAGCGTTAATAA
6[419]	CATGGTTGGTCATCAGGTGTCTCATGTCACTCTGTATGGATTAC
51[357]	CCTCGGGGGTGGATTGAGGGCGAAGTCATGTCGGACTCAGGA
19[243]	CTCAATATAATTAGTTGCAAAAAAGCGGTCGCAGTGAGGCTCCGCC
45[294]	CTGTCTTCATATGTATCGTGAGGTCCGACCCTGCCCAGCGAT
23[63]	TTTTTTAAAACGACGGCCTCCTGGTTAATAGTAG
27[266]	TAGCGGTCGTTTAAAGGCCAGAGTAAGGCTCTTGATTAAATGT
29[308]	CCATAAAATGCCCGCTTGGCTGCTCCAATGGAGCTTGTGTACGG
43[343]	GGAGTCAAAGCTCACTGATATCAAGCCAGTCGTGGGGTA

52[422]	TAGTTTTATGAAGCTCTAGAGCGCGCCGGGCT
14[223]	TCTATCAACTACGTAATCGGCGCGTAACAAGGAGCGGGCGCTCAGGAAA
13[168]	TAATTACATTCTGGCAAAATAGATAACCCAGCTACAAGGTAAAG
24[258]	TCGTCTGGCGGTCGCCCAGACCTGATTTCGCTGACACACAGCCCACTATT
7[392]	GACCGATCGCAGTAACTTAGCCCCATGGTAGA
43[399]	TTTGCTGGGGATAATACGCGTCGCTATTCAGAACAAAACCTT
51[105]	ACATTACTAACGTTGATCCAATATCAATAACCTGTTTTTCATCA
2[335]	GTCATACTAGACCCGGACAGTGTATATATAAGCAC
13[189]	GATAGGGAAGGGAACAAAGGGTTATTAGACTTTACCAACAGA
44[317]	GTGCCAGTGCCTGGGGTTCATATTTCGTTCAT
24[300]	GGCCAGGCGGAAGCTAGGAACCGTTATGGCATGGCAAGAACGA
25[343]	TTTGATATCGCACTCAATAAAGATTTGGCCAAATAGCAACTGGATTACTAA
35[378]	CTATGACGCAACGTAGGCCAAGTACCTGGCAACTTTCTGGGAGAAACTACCC
49[189]	TGGGTTACGAGCGTCCAAAGAGTCAGAAGATAAAACTTAGGT
35[294]	ACCTGCCCCTAAGCCGCTTTATAGCCTGCTCTTCTAACATTGT
21[224]	CGAAAGCCCGAGCTCACTTATGAACAACCGACGTCCAAAGGG
13[422]	TTATTGAGCGACAGCTGCGAGACTGTTGTGTCACTTTCCACTCG
13[399]	AGGGTAGACGCATAGATTACACTTCTCTCTGATTACGTTGGG
19[287]	CCCCTCCCGACAGAATGCAGGTAATCCGGCATTCCCAACAAAG
25[392]	TTGGCATTGATACGGAGCTATCTGGTGTCAGCGCAT
45[83]	GGCACCGGAAGATAACAGCTCGAATTTTT
43[126]	GCAGACATTTTCAGGTGTATAATTTCTTTGAGGACTAAACTAAT
42[475]	TTTGCGACGCCACGCGCTGAATCTGCCGCCTT
5[392]	CCGATAATATTCAGATAGTTGGGTCTCCGTAA
35[428]	AAAAGATTGCACAGTGACTCTTCATACTCCTTTCCGGGTTTTTTTT
19[329]	AAATCCTTCTAACATAAATATGGATGAGAGGACCCCGTGCAGC
20[391]	TTCAGCAGGAATGGGTACGCTTAATGCGCCGCTTTGCCCGACT
53[119]	TAGTACAGTCAGTCAGGAGATTTTCTGTATAGCCGG
47[252]	GTTACAAAGAATAATTGTGGCGACGGGCAGTGATTGTTCTGA
16[223]	AGTACGAATGCCAATTACTGGCGCCTTAGCTGATGAAAAAATATCAAACC
49[420]	ATGATTCGGGCTCTGCGTCGGCGAGAGCATGAAGGGTGTATCCG
53[231]	CTGTATGACATAAAAAGTTTACACGTTTTTAACCATCGGAAGTTAAATAAA
7[445]	ACGACCTGCAACGCTCCACCCATTATGGAATC
11[392]	TTGTGGCCGCACAAGGCCGCCGTTAATCACCA
28[97]	TTTGGGGCGAACAGGGAAGTTGGGTAACGTTTTT
19[371]	CGTTTCAGGGAGGTTTGAACGGGTCCCAACTCGCCAATCCTCC
29[350]	CGTTTAACGTCAAACAGAGGGATAGTAGCTGTGAGCGGTAACGTTATGCA
50[475]	CATGGCCAGACGAGAGCAACGTACGTTTCCTC
9[445]	TGTTCCGACAACAGGAGTAAAGGGCACAATGA
20[433]	TCTCAACGTGTAGTGGTCCCTGAATGGGTCAGTTGCAGCCGGA
2[398]	ACTCAGTGCTTTTGCTTCACTGATCATCGTGTCGGTTCCATTACCGTTAGGG
2[356]	AAGCCAGCGCGCTCGTATATTACTTATCCAAAGCTGATGGTGCATACCAA
11[445]	ATGAAGTTAAGCGGAGGGACCGAGTGGGATCT
53[154]	TAATTCAGTATGTAACTGAACATAAATACAAAGATT
16[265]	TCAAGGATCTATGGTCGTTTTGGAGACTTTTTCATGGAGCATC
4[125]	AACAAAGCTACTGACCATGTGTAGGTAAAGAATCGGTTACAGTG
40[174]	TTTGGAGAATTTAGCAAACGTAGAATATAAGTATGGGATTTTGC
26[293]	TGAGCAAGCGCTCTCCTGTCTTCTATAATATACCAGGCTAAG

40[90]	GTGGGAATAAGCTACGTGGGCTATATCTGAGAGTGATAGCGCC
12[328]	GGGCTGAGTGTACGAGACGCATATTGTACCGGTCAATGAAATCTCATCTAAC
43[357]	ATCCTATACATGGCATAGCGGTCATACTAGATTACAAAAGAC
52[475]	GGAGGATATTCGACTGGACTGGCAGCACCCTC
14[265]	CTTAATGTCTCGGCCTTATGTTCTCGACCATCTCA
41[266]	ACCGCTTGTTAAATTTATCAAGGTCGTGAACGAAAGACAAGGT
13[317]	ATCCTGATTGAAACATAGGCTAATGTTTCTAGAGATAAACAGGA
23[98]	TAGCATGAGAGATTCATCGCCTGATAATGTGAATACCAGAGCCG
27[308]	CCTTATTAGACCACCAAGTATGTTGAAGTTTGAGCCCGCCCGCTTTGCAG
21[266]	ACCCGGTGGTGTTAGCATTTATCACGATCCCCCATGTCCACCAC
9[392]	CTAAGTATTTTCGGTGAATTTGTTCAACGACT
23[182]	ACCTCCAAACATACCTACCCCATCTCTGATACTCTTGAAATTG
51[252]	TTACACGAAATCTTTATAGAGCTTGCTACTCAAGAGGTGGGC
41[308]	TACCGTCTAGTGCTCGGGGTAAACCTTGTCAGTTCGGATAACA
51[147]	TTCCAAATATCAACTATACATAATCAGAGCCGCCAGTCGTCT
27[329]	CAGCTCACTGGATGTAATGTGTTTAGCTACCTCGCCACTTCCCCGCCGAGA
45[316]	GATTTCGACGACATTTAGCTTTGTTACAACTGGTTCCGTTGGTT
19[413]	ATTCACGGTCGCCAGAGTACCATCTTACTAAGTTATGAACG
46[160]	AAAAGAATTAGCATTCCACAGACAACCCTCAC
49[210]	GTCCAACTACCTCGCGCGTCAATCAGGAACGGTACGCATTTTCG
1[77]	AATGTCCCGCCACTTTTGCGGGAGAAGCCTTT
51[399]	TACCGCGACGTTGAAGTACGGCTAGGTTTTCAATTCTCGTAG
10[127]	AATTTGTTACTTAGCCGGAATGCCGGGCAAAGAATTAGCATCATTT
6[125]	CTTGCCCTGCATAAGGAAGGCCGGAGACAGTGAGCATACTCGTC
49[441]	GCAGGGGGGAGCATCACCTCGTCTGATAAGTGACCCAACCGGGA
8[471]	CCGCGTACGGGGTAATGGATTATGTCTTTATACATTAACGTCCAGCTAAATTC
8[125]	GTAGTAAATCGCAGACATGATATTCAACCGTGCAATAATTGAAT
19[453]	TCTGGGCCCAAACCCTCTGAGCCGCGAGGCATGTAGTTTTTTT
13[441]	TCTTGCTCGGTCGGACGTAGCCTTGTAAGAGTAGACGTTTCAAC
37[210]	TTAGACCTACCTGTGATGAGGCCTTAATCTTCAGATGAAGCGT
6[167]	TTATTTATCGGCGTTTGGAACCTATTATTCTATTAAAGTAAGG
2[111]	GGCTCATATATTTTACAGTGAGATCAGCCAGGGTGGTTTTTTTTT
5[445]	GCTAATCCGCTTCTAATTAGGATGCCGATAAG
25[427]	GAAATAAACGTTCGCCCGCGGCTAGGTAAGTAAAAT
43[168]	AGCTAAAACATAGCGGGTAAGGCGTGAACGCGCCTGTTAGCAAT
48[265]	TTGGCAAAACAAGGTGAAGACCTCAGTGTCCA
39[126]	CTAAAGTTTCAGCCGGAACCGCCTCCCAGGTGGCTCTTTCCTT
25[462]	CTCCTCGCCTACTCCTGGAGAGAGACTTTTTTT
10[209]	ATTCATTTAGGATGTAACAAGGACAGAAGATCCCGGCTGCCGATGCAAC
45[105]	AAATTTGAGATTAGAATCAAATGTGTCTGGAAGTTTTTCGCATT
13[84]	ATTGCCTCCTTTTGTATGTAATAAAATAACCCCGCCACATTA
43[147]	AGCCCACAATGAAATTTTTGTCATTAGCGTCAGACGATAGCA
26[314]	GATGATAGCTGTATTCTGTTATTCTTGCCTTGTTTCCAACCCTG
48[107]	CTTCCTGTAGCCATACCCCGTTGGGAAGGGCG
3[445]	GGTAGGCTAGTGCATGCACTCTTGAAGACCGC
29[399]	TCTAGAATCTAGTGTATCGACAGCGCGGTGAAGAATGGACCGCGCGGTACTAGC
53[189]	TGAATACCATGTGCGTTATACGAAACTCAAAACGGTGTGATTG
47[273]	ATCAGTCAATCTAATCCCAGAAGTGTCAACCAAGTCAACCGTCT

16[307]	ACACTTCTCCCTTTGACGGTAACTATCGTCTCGAAAAGAACTC
23[224]	GGTCGCGTACCGCGCCTCTCCTTAAGATTGGCGTTTCTTCGGG
4[167]	TTTGCCAGTCCTCCCGTATAAACAGTTAATGGGAAAGCAACGT
24[363]	TCTGCAATAGGGTGCGTAGTTGTGGGGGCAAGTCGTAGGTCTCGGTGACG
41[350]	TTGTAAATAGTAACGGTAAGCGCTTACAAGGCTTACGCCTATA
40[132]	TAATTATACAAATGTTTAGACTGCTGGAGCAGTCGGATTCTCC
53[280]	AGGATGACCCACGTTAAGGGTTAACTGCTACTGTTACGGTGGTGCGGTGA
29[452]	ACATTCTCTCACCTTCTGAAACACTTGGCAGGATCGCTTTTTTT
21[350]	CGGCTGTGAGCGCTAACATAGTGGTCGCGAGTACGGGGTCTTCG
22[363]	AAGGACTCCCGAGTTGAGAAATACATTGTTGCCCATGCGTTCGGCCAGGA
24[447]	CTACCCCAGTCCGGAATTACGAGAACCATAACACTGAGTGTCC
2[377]	TCACTCGACGCGAAAACGGTCAATGACAGCCTGTATATTTCTTTCGGGT
21[287]	AACGTCTGGTCGCAGTGGACTATCTGGCGTTTTTCTTTGCGC
13[462]	TTGCGATTATTTTGTCAGCGCCGTATGAGCAGCCCAAATTAG
16[356]	AAACTGCCGAATCGGTAAGAACTTGTACAATTCCAATGTAACAAAAATATA
23[266]	GCGAATGCCACCCCGCTGCGCCTTATCTCTTTTCTTTTCAAGT
4[216]	GTTATACAGTAACAGTATTGTTTGTAATTTTAAAAGTTGGAGCACTCCTAA
8[167]	ATTTTTTGTTTCTAAGTAAGAGGCTGAGACTACAAATAAACGA
38[471]	TTTTTTATGCTGCGACCTACTAAACTGCCGCTTA
25[63]	TTTTTCAACATACGAGCCCCAGTCGCGAACCAGA
12[356]	TAGTGCGCAAGCTAATCATGGAGTATCGCAAATGAGAG
14[279]	TACAGGCACGAAATATAAAAGTTGGCTAGCTCCTTCGGTGGTGGACTCGTTT
45[357]	CTAGGTAGTTATTGTCGCGCTGGACAGTCACCCCGGATACGA
12[377]	GTCCAGAGCGATCAACTTTACGCATGCACCAGACT
8[209]	CCAAGTTACATGATGGCGGAGCGGAATTATCGGAATTGAAAACG
46[265]	CTTAAATCAGGAACATCCGGCACGGCACCATA
14[307]	TCTGGATAACTGGCCCCAGGGCGGGCGAAAGACGA
27[427]	ACGTACGTAATCAACAGAAAGGTCGATCGAAACTAATGATGTTTTTTT
28[132]	CCTTAAGAACGATTATACCAAGCGTCATTGCTTTCA
39[168]	ATCATTTAATTTCGAGGTGAGGCGGTCCCGAGTAAGAATCCTGA
51[168]	ATTAAGTGAGTGATAGCTATTTTAGTCCAAGAACGGGTGGCATG
49[231]	TACGTTGACGGAATAGGGGACTACCCCGAAGCCAAAGTGCTTCT
16[494]	TTTTTTTCGATTACTGCTCTGCCCTCTGGTGCAAAGGTGGTGTCGCAG
44[422]	CAAGCAGAAACAGGCTTCGGCTGTTGAGCCTA
16[405]	CTAGGAGGTCATTGGCCGGTTAGCGGTCGTCTACTCTAGTTCAATCTATT
23[308]	GACCTAGATACTGAGTCCAACTCTCATCTTCAAGGGAGAATGCCGTGCGAG
6[209]	AATAACGGATCAATATAATTTTGCGGAACAAATCTAAAACCATA
28[258]	AAAGGACCTCATGCCGTGGGACATAACACTGAATACATAAATA
28[174]	AGAAAGCGCAAAGTACCGCACTCACGTCGAGGCCACCCTCAGAG
39[210]	GAAGTTCCGGTTCTGCTTAGGACTCGGGCACTCGTCTCGTGCA
13[231]	TGGCAATAGTTCCCGGTGGAGGTTGGTCCTCACCGCTAGGGCGC
12[139]	CAAGGCCGCTGGCCCTCTTCTGAATTCAAGAACCGGATTTGCCCTATAGAAG
22[104]	AAGGGCATCAATTCTACTGGTGTAAGTCACGACGTTGTTTTT
16[447]	ATGACACGGTTTTGTCGGGTCTGGGTCACCACCCTTCAAAGTA
23[357]	AACGTTAATTAACACTGTTCCGGGACGGCCCAGCTTCATCTACCAGGTGA
6[251]	TGATGTTTGGGACGAAGATTAGCTACGATTCGCTATCTGAAAC
1[235]	GCCTGACATCTCGAATAACTTGCAGGAAGGCA
53[329]	CACCAAGTCTCAAGACGCTCCTCGCAAGCGCTATGA

10[251]	CTACCCCTGAGAGCATGGGTAGCTCGATTTGAGTTTAGGAATT
43[378]	TCGTCCAGTTGTTGTATACGTATGCCGAGCCTTAGGATGCCATA
24[494]	TTTTTTTCTTTAAGTTCTAAGCAGGCGTGCATACCGTGTTATATCTAA
51[420]	TGATTTGGTTTAAGCGTATGCCCCGAGGTTCGGAAGACCAAAGT
40[216]	CCTTGCCCCCGTAGGGCATACAGAGCGGCCCCGCAAGCGGTT
47[316]	TGCCGCTCACTCTCTGAATTCAGAGTAAAGGTTCGCACCTATCC
47[294]	TGACTAACAAGTCTCGACGGCGCTCCGCCTTTCTCAGTTGCC
4[265]	TTTTTACTTTTCTCCTAGGTAAAAGAGTGGGCTGTCGGCTATGAAGGATT
53[364]	TAATTGGGTCGCGCAGCCAATTCCGGCGTCCGTCTATTGGCTA
14[321]	AATTAATGTCTTGCTCGAAGAACCCAGCAAATAGAGGGTATGAGACAACG
29[98]	AAGGTGCTATCAGGCGAAACAAAGTACTGCGATTACCACCCTCA
13[252]	AAAGACTCCGATCGGGCAAAAACAGGGCCACGCTAGTTAAGC
10[293]	CAAAGACACGACTTATCCGCGCACATCCAGTTCGATGTGTCAC
21[308]	GTATCCGTGGCTCAGCCGACCGTCGTTAGGCGATTCGCATTGAG
45[378]	AGACTACTTCGTGAGCAGTCTAATAGGTGACTTTTATCCCGGAA
4[315]	TGTAATGTGTGTGGCAACGTAGCTCTTGATCCAGGTATGTATTATTGAAGCA
39[252]	CCTTTTGACGGGTGCTCTTGCCCGGCCCAGCCAGTCACGCTGT
8[251]	AAGTAAGGATTTGGGACATAATGCCATGATGCAGAAGTGAATA
26[356]	ATCGCGGCTGCCATAATCTCAAGTGTGGATGTCTCCCGGGGT
41[392]	TGCACAAGATCATTATAGTTGGCCATGGTAACCCAACAGCACA
14[174]	TCTTACCAGGTTTGCCGGGCTGTCCAGAGAATATAAAGAAGCGGTGAGGAGG
23[406]	CATAAGGTTACCGGCCCCTCCGCCTATACCTAACTTAACATCCGGAAG
39[294]	AGGTATAAATGGGGGAGTCATCGACAATGTATGGCTTCGGCTG
27[379]	CGAAGCATGCGTCGACCTTGAGATGTCGGTCAAAGGGCATAGTCCTCTCC
8[293]	GCAGATTACCAGCAGCCATTTAGAAAAATAATCGTGCATGCGC
41[434]	TGAGTTTGGGACAGACTGTGAAGTATCTAGGCGTAGCAACCTGA
28[216]	AGCAGATCTAACTGTTGGATGCTCTAATTACAGTGCCACGCTG
1[287]	CTACACTAGAAGTATCAATTGGAGCGACTGCT
26[125]	TGCGCCGCTTTTTCATGAGGCGAATAAGCATTATCACCGATAGT
40[237]	CGGGTCCCTAGAGCCTCTTCATTTGGCAAGTGTCCCCGATTAT
2[153]	TTCGGGGTCAGTGCGAGAGAGGGTCAGCTTGCTTTC
41[462]	GGGCTGATTGTTTCTAGGGCTGACGCTTTTTTT
44[475]	GGACTCAGCAAGTCTGTGTATTTAGCCAATCG
17[329]	ATCCCATCGGATCTTTTCACTCTCCGGGAGTCCAACACTTTAT
12[398]	GAAGGGCAGCTTAGCCTATGTGCATACCCATCTATC
46[317]	CACAGTCGTTTCGGAGTCAACCCCGTCGTGTA
40[279]	CAGAATTCGCCCCTAAAGGGAGCCACTCATGAAAGTTCGGACCT
28[342]	CCTGGAGTACGTAGTCCCCGGGTACAACATGCATAGCGAACAA
21[119]	TAGTCTTCTTAAAACCGGAACGAATTCGGTCGCTGACTATTA
2[419]	TGCTCCTCGGGCGGAGGAACTTTAGTACATTCGGGGTTCGTGCCATCCCGAG
53[406]	ATGTTGTCTTCACCGCTTGAGGCGAGTCTCGGTGAC
45[147]	TGAGTAATAAGATTGACGGAATAGCATTTTCGGTCGTAACAC
10[335]	TGGTGACGATATCCTCCCTAGGCCGTGCGAGGAATTATGATCT
49[252]	AGCAGTCCCTTAACGTGAAAGCCATCTTAGGCACCATGACGT
43[189]	AGATTAATGTCGCGACTTCTTTGATATTTTTGAATATAGCTT
6[293]	GTAGCGGTGCAGGATTATCTCATGAGCGGATGATCTTCCCATC
43[420]	AGCGCAAGGTATATATCATTTTAGGCGGATTGTGCCAGGCAGAT
12[419]	GTCGTAATCTTTGCTAGGATTGCCAGAGCGTTCTTG

21[245]	GGGGGTAATTAAAATGGTGGAAGAAAGGCTGGCTGATTCTATGA
51[210]	CTATACTACACGAGATCCTAGTAGTTTTTATAATCATGTTCGTG
53[441]	CAGTTACCACGGATGTCCTTCGACTATTCAAT
21[84]	TTTTTTTTGCGTATTGGGCAAAAGATTTGAGAGCTT
49[273]	ATCACAAAATGAATAGCCCGGAAGGTGCGGCGACCGAGTGAACC
10[377]	ACTATATGGCTCGATCGGAACAAACTCTATGATTGGCTAACCG
6[377]	AAGTCGTTCAAGTAATACGTGTAGAATATCAGTTTGTATTCTT
36[471]	TTTTTCAGACTATCTTCATACATAAAAGGATTAC
26[146]	AAGGTGAGCGTTTTCATCGAGTTTATATAGAATCGCCCATTA
47[83]	CAAAGCAAGCCCCCAGCTCCCCGGTTTTT
51[441]	TGATACTAACATCGAATAAATTCTTGTCCCCCTCATAGTACTAG
26[377]	ATCAGGCTCAGTCTCACGCTCCGTTTGTTCGCTAAAATCATAGG
43[210]	GCTGAGGGCCGCTCGCCGTCCGGTATGGTTGCTTTGGTATAATC
22[146]	GAGCCACCAGAACCACCTACCTTAAACGGAGATTTGTACTACA
28[391]	TGCCTAGATGGTACGCGGCCATGCTCTCCTAAAGGGCGATACCAGTGCACG
47[105]	CCAATAAAGATTACGATCATTGAGATTCCCAATTCTTTTTTTAA
38[188]	TAACACCAATGCTGATGCAGATTGATTCGTGAGGCATTAGGT
43[441]	GCGATAGTAATTGTGTTTGAGTCGTAACTCACCCGATTGGACCT
13[273]	AAGTAAATCGACGCTTAGTGGTGGCAAAGGGAATAAGTTGTCAG
4[377]	CAACTCAGGGCTGATCTTGTCTTACAAATGTAAATACGCGAACATGATCT
50[107]	ATGTGAGCGAGTAATGTCAATCGCTATTACGC
21[329]	CCCTTACGCGAACCTTCGACACTAATATCATTTCTCCACCTC
22[188]	AAAAAATCTAAAGCATCACATAAAGCCGTTTTTATTTTAGGCG
47[210]	CTTTTGTTCAGAAGAAATTTCCGAAACAGGAGGCCGGCAACCGC
23[453]	GTGTTAATCTAAGATCGGTGTTTCCCTAGCGCACGGTTTTTTT
48[160]	GAGAGAGATGTAACGATCTAAAGTCCCTCAGA
39[336]	AAAGGAGGAGGATATCAAGCGCATCTCTGGCCGGCTCGCCGGA
8[335]	TTGGCTACGTTTGTCGATCATCTGGGAGTTTGTTTGAAAAGCA
33[84]	TTTTTTTCGTAAGCGCGTTTTAATTTTT
46[422]	CGCCAGGTATGATGGGGCTAATAAGCACACCG
28[300]	ACCTCAGTGTCGCTCCAAGCTGGGTAATATTGCCACATAGCAG
47[441]	AAAATCGGCAGGCGACTGTCGTGACATTTAATTATAGGGTGATT
12[440]	CCTCACCTTGCGGTAATTCGCAAGCCCTCACTTGC
39[378]	TCTACAAGTGTCCATACCTCAACTTCCACAACTGTAAATTGTCCTGTGCGAA
47[336]	TTCCTTGCTGACAAAAAGCGACCGGGAGGAGTATGAGGCGATCA
8[377]	AAATTGCCTATTTAAGCCGAGATAGGGTTGATTTTAGCCAGCA
2[195]	GACGTATTAAATCCGCGAAAAACCGTTATACAAAT
45[399]	ACCAGCACGCGTAAGCCGGAATAAGATCGACGGAGAGCTACT
6[335]	TCCGTTAAGCGCGAGGACCATCCATTTCACAATGAAAACGCTG
2[440]	TCATGATTGGTAGGGCGGTTTTAGTCAGAATTGGATGCTGGATTGTCCA
17[63]	TTTTTCTCAGGAGAAGCCTCCGAACATCATACAG
7[77]	ATCTGTAAGCAAAAGCTAATTCAAAAGGGTGA
45[168]	AAAGTCCCTTAGAAGTACCGACCGTAGTCCTGAACAATTTAAGA
31[98]	TTAATGTAGCTCAGAAAACGAGAATGAACAGAGGTTTCACGTTG
49[399]	TCATAGAATAAGGACCTGCTCGCAGCCATTTGCTGGCACTAC
1[445]	TCTAGGCAGTCAATTAAGCCATGAACTGCACA
31[63]	TTTTTAATTGTTATCCGCTGCTGTTTCGGCCTCAGGAAGGC
49[294]	TACGGCCTCCACAACCGAGGGCGCGCTTTCTCATAACTACGA

51[231]	GCTCGTAGTCATCATACTTGGTGTTAGGCGAAGGCTTAAGACTT
1[392]	GTCCGGGAAATAACATTTTTTTCAGTTCTTTC
17[98]	GCAAGAGAGGGTACGCGACCTGCTCCATCAACTTTTGAGGCAGG
5[77]	TCTATGATACCGGTACCAATGCAATGCCTGAG
47[357]	TACAGACGTGACTTCCGAGCGGGGTCACGTCGCTAAGGATCT
6[471]	CAATCTGATGTGGCTCCCCGGTCGGGACCGAGCCGTGTAACAAGGTCAAATAG
44[160]	GAGCCCAATTTCGTCACCAGTACACACCCTCA
31[140]	AAAATTTTGCCTCAATCAATAGAAAATAATGCATAAATAAGAA
49[168]	TAACGGCTTCTGTGGCAATCTTCTGTCAATAATCGGCGCAATAA
26[167]	TTAATTGCAACAATAGATAGTGATAAACTAAATACCTACAGGGC
4[419]	CCATAGCAGTCCCGTTTCTGTACGACCGTTACATCGTCGCAATC
8[419]	TGCCTCGATTGCCCGTCTCCCACCATTGGGCAACTGGAAAGCTG
10[419]	GGGCTAAGGCACCCGGCATGTGGACAATCACGAGCTCCCTACCA
12[181]	AATAAAACCAGCAGTTATAGATTAACCAACGCTAACCCACGCTGTAACCG
40[321]	AGAGTTACCCAATAAAGACGGCGGAACTCACGCTCACCGGCTC
14[118]	CAAAGCGGATTGCACGGGCAATGTTTGCGGAAGTAC
47[126]	ACCCTGAACCGCAACTCAAAAGGAATAATGCCACTACTATCATA
12[223]	ACGCTGCAAAATCCAATTGTCGTCGTAGATTTTCAGGGTTCCGAGAATAATA
4[471]	ATTACACGTTCAGCCACGTGGTTATTGAAGATAAGTTGTATTCTGCATATCCG
10[471]	AACTGCTCCCAACCACACGTCCACGGCTCATGGGTTAGAATCTAAATGGGCTT
45[126]	GAGGCAGAGCCAACCATAAGGAATTGAAGTTTCCATTGAATTAC
28[440]	TCCTACTAGGTTGGGGTGCTCAATGGCAACAAAGGACTCTCAGAACGGCA
37[84]	TTTTTCGGAGGAGAACGAGTAGATTTATAAATATAGGCACCAA
42[107]	AATATTTTGTTAATGTATAAATCGCACTCCAG
7[130]	ACCAGAATCCCCCTGCCTATTTCTAGCGAATA
26[398]	CAGTTAAAGCTCGAGCTTAAAGCCAATTTCCCAAGCCACACC
43[231]	TCACGACTGAGTCGATGTGTCAAATCCCCACGAAGTATGGCATA
22[230]	CGGTGCGCCTACGTTTTCGTCAGAGTACCAGTAAGCACCAAGA
52[107]	TCGATGAACGGTAATCGTAAGATTGACCGTAA
31[182]	TAAACACAGACAATTAGTAATAACATGCGCGTACAGTGGTTCT
28[494]	TTTTTTTCAGATGCGGACGTCTACAGAAAGCTCCAATGAAAAAGCAAGTTACCCT
37[126]	CCTAAAGAATAGTAATCAAAATCACCGAAAAGAACATGTAGAA
46[475]	GACAGCGATGGCATTTGGGAACGGTTTATGCT
9[77]	AATATAGGGGCCAGCCTCACAAATCACCATCA
13[294]	GAGGTTGCGTCGCAGTGTCGTGGAACAGTATTTGGCAAGTCA
50[160]	CGGAGCGCTGACGTTAGTAAATGAGTTTAGTT
48[317]	TTCTGCGCCCACGAACCACGGAGGGCTTACCA
45[420]	ATCAAGCGGATCTGAATTTCTCTGTAATATGCTTACTGTCATGC
2[471]	GCGATTGTGTGACCAAACACAAGGAATTATTTTTTT
3[77]	AATCTATTTACGATGACCCAAAATTTTTAGAA
49[83]	CAACTGGTTGATAAAATATAAAGATTTTT
18[454]	CTCTAAAGAACCCATCGATGGGCGAATATTTTTTT
47[147]	CCTGTGAGTTAAACAGACGCAAATTTGCCATCTTTTACAACG
49[316]	GTAAAAGCTCCGTCTGTCTGTACATCGAAACCGCGTCCGCCTCA
51[126]	AAGTTACCGCCATTTCCCTTCAACAACACTCATCTTTGCAAAAG
32[342]	GTCAGGTAGGGAGAGTACTCAGCCTCCGCAGCCGCTGTTATT
9[130]	GAATCCTCGAAACATGAAAGTATAACGCGACC
47[378]	GCACGTTGTTAAAACATCTTGCACCCCTTCGGGTTGGGATCGCT

Honeycomb-capture-Z_1

13[63]	TTTTTAGCTAACTTTGTCAACTTTTTTTTTTCTTATGACTTTTTAAGATAGTCTAAGTCCGA
2[82]	CTGGAGTGTAGTGCTTTTTTTTTTTGAATTCTTTTTTATATCCCTGACACCTGTA
4[82]	CGGCCCTGCTTAAGTTTTTTTTTTTGTCCTACTTTTTATCCCACATTCCCTAGGC
6[82]	GGTGGGCATCATGCTTTTTTTTTTGCACGACCTTTTTAAGATAGTCTAAGTCCGA
8[82]	CGGCTGACGTGGTTTTTTTTTTTTGTGAATCGTTTTTATATCCCTGACACCTGTA
10[82]	CAGGGTGGATGTTCTTTTTTTTTTTCTAAGCTTTTTATCCCACATTCCCTAGGC
42[76]	AGCCAGTAGAGTATCCGCTTTTTCCACGACGTTTTTTTTTACAGTATCCTGTGTGATTTTT
44[76]	AGTTGTACTAAGCCTCGGTTTTTGGCAGTTTTTTTTTTTTTGAGGGGAGCTTTCC
46[76]	ATACTTACCCTTCAGAACTTTTTTCCCGTGCTTTTTTTTTATCTGCAAACCAGG
48[76]	AGCCAGTAGAGTATCCGCTTTTTATGGCGCATTTTTTTTTTCGTAAAGGCTGCG
50[76]	AGTTGTACTAAGCCTCGGTTTTTCATTGGTGTTTTTTTTTTTAGATGCGGTGCGG
52[76]	ATACTTACCCTTCAGAACTTTTTTGGGATAGTTTTTTTTTGTCACGGCTGGCGA
1[108]	GAGCCGTCGGATCCAGAGATTACAGACCAGGCGCATAGGCTTTTTTCGGATAATTTTATTCTG
3[108]	GCTCCATTTGTACCGTATCCACAGATGAAGAGTAATCTTGTTTTTAGGACTATGACAACATCC
5[108]	CCTTTAAGGACAGTACTGTAAACTTTGTCATTACCCAAATTTTTTGCATCGCTTTACTCAGCT
7[108]	GAGCCGTCGGATCCAGAGGAGAACCGAGCTCATTCAGTGATTTTTTCGGATAATTTTATTCTG
9[108]	GCTCCATTTGTACCGTATATGGTCAATACGAGAAACACCATTTTTAGGACTATGACAACATCC
11[108]	CCTTTAAGGACAGTACTGTTAACGAGGTGGGCTTGAGATGTTTTTGCATCGCTTTACTCAGCT
42[129]	GCAGTCCATCTCCGAGGGTTTTTACATAACGCCAAATAGGAATTTACGTAATGGTAGGTTTCA
44[129]	GTGGCTATATGCCCGTAATTTTTTAGTAAGAGCAACCATCAGTTTCTTGCAGAAGTTGAGGGA
46[129]	GCAGGTAAATCTAGTCAATTTTTGTTTACCAGACGACAGGTAGAGTTGATCCTGCCTCCCAGT
48[129]	GCAGTCCATCTCCGAGGGTTTTTTAGCGAGAGGCTTGAACAACGCACGTAATGGTAGGTTTCA
50[129]	GTGGCTATATGCCCGTAATTTTTTGCCAGAGGGGGTAAAACGAAACTTGCAGAAGTTGAGGGA
52[129]	GCAGGTAAATCTAGTCAATTTTTACGTTGGGAAGAAAAATCTAAATTGATCCTGCCTCCCAGT
1[161]	ATCGTCAAACTTTCCCATTTGCTATTTTGCATACATTTGAGGATTTAGAAGTAGAA
3[161]	AGTTCCTGAATAAACATAATCCTGAATCTTGAGCCGTCACAATTCGACAACTATAA
5[161]	GTTTTAGCGGACGTAACAGTCTTTCCAGAGAACAACTATGCCCGAACGTTATGATT
7[161]	ATCGTCAAACTTTCCCATCAAAATAAACAGTATCTTTATGAGTAACATTATCATCC
9[161]	AGTTCCTGAATAAACATAAATCCAAATAAGGGAAGGTTAGAAACCACCAGAAAATT
11[161]	GTTTTAGCGGACGTAACAAACGTCAAAAATAGTTGAAAATCATATTCAAAGTTGGC
42[197]	AGAGAAGACGCTGAGAAGATCCTTGATCTTACCGAAGCTATTAGCTCTTGAGAAAT
44[197]	TGACTTATCAAAATCATATTAATTTTAAGCAGATAGCCCATACGATCATTAGATTT
46[197]	TAGGCCTTTTTAACCTCCAAATCGTCAGGAAACCGAGGCCGTCATGCCCGGGTCGC
48[197]	TGGATATATAACTATATGATAACCTTGAATACCCAAAATATTAGCTCTTGAGAAAT
50[197]	CATCAATCCAATCGCAAGCAATATATGACTCCTTATTACATACGATCATTAGATTT
52[197]	TCTGTACCTTTTTTAATGGAAACAGTACACCCTGAACACCGTCATGCCCGGGTCGC

Honeycomb-capture-Z_3

1[198]	CCTACCATATCAAAATTAGGATGTCGAGCAGATTAAGGTTTTTAAGATAGTCTAAGTCCGA
3[198]	TGGGAAATAAAGAAATTGCCTACATAGCAGAGTGGAGATTTTTATATCCCTGACACCTGTA
5[198]	ATATTAACGTCAGATGAACCAAGCGCAACCCAGCGTCATTTTTATCCCACATTCCCTAGGC
7[198]	TGACCTTTTACATCGGGAAACTATTCGTTACTGCCTTGTTTTTAAGATAGTCTAAGTCCGA
9[198]	CATTCGCCTGATTGCTTTCCGACTAACTCTTCATAAACTTTTTATATCCCTGACACCTGTA
11[198]	AGAAAATCGCGCAGAGGCATCGGGCGGCAGCCGGAATCTTTTTATCCCACATTCCCTAGGC
42[234]	AGCCAGTAGAGTATCCGCTTTTTATTTCCAGATAGACGGGGCCAGGGGCCTACCAACTGAC
44[234]	AGTTGTACTAAGCCTCGGTTTTTAAGAAACCCTGCCTCGTGTGATACCCATCGAATTGCCC
46[234]	ATACTTACCCTTCAGAACTTTTTGTCGCGCAAACGGGGGATAAAAGCTGGCGTCCAAACCT
48[234]	AGCCAGTAGAGTATCCGCTTTTTTGCCTCTAACCCAGCTAAACATCACGTGCAATATAGCG

50[234]	AGTTGTACTAAGCCTCGGTTTTTGGATCCAAATATGAATATCCCAATAACGAGTTTGTCCG
52[234]	ATACTTACCCTTCAGAACTTTTTTAACTTCCGCAGCACAACCAGTTCCTACGCAGATGCAA
1[266]	GAGCCGTCGGATCCAGAGAAATGCCGCAAACTAACTACGGTTTTTTCGGATAATTTTATTCTG
3[266]	GCTCCATTTGTACCGTATACACGGAAATGTTTCTTGAATCTTTTTAGGACTATGACAACATCC
5[266]	CCTTTAAGGACAGTACTGTCCTTTTTCAATAGGCGGTGCGTTTTTGCATCGCTTTACTCAGCT
7[266]	GAGCCGTCGGATCCAGAGTCAGGGTTATTGGCAGAGCGGGTTTTTTCGGATAATTTTATTCTG
9[266]	GCTCCATTTGTACCGTATATATTTGAATGTACTGGTAAGTTTTTTAGGACTATGACAACATCC
11[266]	CCTTTAAGGACAGTACTGAAATAGGGGTTCGCCACTGGGCTTTTTGCATCGCTTTACTCAGCT
42[286]	GCAGTCCATCTCCGAGGGTTTTTATACTTGGTCGTCCACTATTAAACGTAATGGTAGGTTTCA
44[286]	GTGGCTATATGCCCGTAATTTTTCCAAGTATATCGTCAAAGGGCGCTTGCAGAAGTTGAGGGA
46[286]	GCAGGTAAATCTAGTCAATTTTTGAGTTTTAAAGGCGATGGCCCATTGATCCTGCCTCCCAGT
48[286]	GCAGTCCATCTCCGAGGGTTTTTTCTTAAATTACCTAATCAAGTTACGTAATGGTAGGTTTCA
50[286]	GTGGCTATATGCCCGTAATTTTTGTCTTCACCTCCGTAAAGCACTCTTGCAGAAGTTGAGGGA
52[286]	GCAGGTAAATCTAGTCAATTTTTGAGATTATCATTTTGTTAAATCTTGATCCTGCCTCCCAGT
1[318]	ATCGTCAAACTTTCCCATAATGAACGCCTGGCTATCGGGTTGGTCGTACGCTGTAAC
3[318]	AGTTCCTGAATAAACATAACATGGGGATGCGACTAACGCTCGCGATTGGGCTAAGTT
5[318]	GTTTTAGCGGACGTAACAAGATAGCGGCTTCCGAGTATTTTCTGTGAAGTCAACAG
7[318]	ATCGTCAAACTTTCCCATCGGTCCAGCGGCAGCTTTCACCCCCGTAATTT
9[318]	AGTTCCTGAATAAACATAGCCCGACTCTAGACGCTACGGCCATACGTAGCGTGACAA
11[318]	GTTTTAGCGGACGTAACACCTATGTTACTAAGACTCGCGATATGTAAAGTGTTGTTC
42[355]	TCCTCACTTCCGTGCCACCTCACTGAAGCTGGCAATGCC TATTAGCTCTTGAGAAAT
44[355]	CCAAGCGTGAAAATGCCCGTACTTTATGGGAGGCTAAACCATACGATCATTAGATTT
46[355]	CAACGATCCTGGGGACTATTGGTTCGATCATGTCGACCTCCGTCATGCCCGGGTCGC
48[355]	TTCTATACGGTCAGTGTGGTTTATATGGGGGGAGTTAATTATTAGCTCTTGAGAAAT
50[354]	GCGAGAGGCCAACAATGGGTCATGCAAGGGGCGTATATCATACGATCATTAGATTT
52[354]	TCGTAATAAGAGCACGATACGTGGTCAATGAAGTCTATCCGTCATGCCCGGGTCGC
	**Honeycomb-capture-Z_5**
1[356]	GATAGGGGCTATCGGCGCCCTAACCATCTATCCATATTTTTTAAGATAGTCTAAGTCCGA
3[356]	TCCCGCCACAGATCTCAGATGGGAGTTGCAAAGTCGTTTTTTATATCCCTGACACCTGTA
5[365]	AAGTTCGTGTACTTACTAAATGGCTTGTTTTTTATCCCACATTCCCTAGGC
7[356]	TGCCTTAGCGGATCGCTTGCGAGATTCCCGTAGACCCTTTTTAAGATAGTCTAAGTCCGA
9[356]	GCTTGCTCATTCGGTGTTTTAAGGCGCGGCGGCATCATTTTTATATCCCTGACACCTGTA
11[356]	CAGTTTGTCTTATCACCGTATAAGATATATTCTGGCATTTTTATCCCACATTCCCTAGGC
42[391]	AGCCAGTAGAGTATCCGCTTTTTTCCGCGCAAACTTAGTGGCGTCCCCCTTTGGGGTACC
44[391]	AGTTGTACTAAGCCTCGGTTTTTGTGTATAGTTCCCCGAGGGAATCGAAGGGGAACGAG
46[391]	ATACTTACCCTTCAGAACTTTTTTCCATTTACTGGGGAAGTAGCACGATGAATGATCGGA
48[391]	AGCCAGTAGAGTATCCGCTTTTTCATAACGGGGGCATAGCGTGTGAATCTCAGCTTGCCA
50[391]	AGTTGTACTAAGCCTCGGTTTTTTGACTTTGCGGATTTCAGCGGACCCGGTAGATCTAGAC
52[391]	ATACTTACCCTTCAGAACTTTTTTCTCTTCGTTTCGGCTAGACTGACCCCCCAGAATGGCA
1[423]	GAGCCGTCGGATCCAGAGTGGATCGGCCGAGCTAGCTGGATTTTTTCGGATAATTTTATTCTG
3[423]	GCTCCATTTGTACCGTATTGCGATTAAACCCCGAATAGTGTTTTTAGGACTATGACAACATCC
5[423]	CCTTTAAGGACAGTACTGGCCCTCTTGTCCTTCCCTCCAGTTTTTGCATCGCTTTACTCAGCT
7[423]	GAGCCGTCGGATCCAGAGAATCAACACATCTATTGAAAATTTTTTTCGGATAATTTTATTCTG
9[423]	GCTCCATTTGTACCGTATTAGGAGCAAACGAACGATGCCCTTTTTAGGACTATGACAACATCC
11[423]	CCTTTAAGGACAGTACTGAGCTATTAGCCTCCGGTTCGAGTTTTTGCATCGCTTTACTCAGCT
42[444]	GCAGTCCATCTCCGAGGGTTTTTTTATAGGTGCTACCCGCCTGACACGTAATGGTAGGTTTCA
44[444]	GTGGCTATATGCCCGTAATTTTTACTTATGCGCTGTACCAAGTGACTTGCAGAAGTTGAGGGA
46[444]	GCAGGTAAATCTAGTCAATTTTTGAAGAGAACACAGACCTTTCACTTGATCCTGCCTCCCAGT

48[444]	GCAGTCCATCTCCGAGGGTTTTTGTTAAGCTTTTTTTCTGTCAATACGTAATGGTAGGTTTCA
50[444]	GTGGCTATATGCCCGTAATTTTTAGGTCCTAACAGGGGGAAGCACCTTGCAGAAGTTGAGGGA
52[444]	GCAGGTAAATCTAGTCAATTTTTCGCTCGCTTTTACGCGCCCGGTTTGATCCTGCCTCCCAGT
1[476]	ATCGTCAAACTTTCCCATAGCCAGAGGTCCTTTTTTTTTTTTCTAATTCCTCAGGAGGTTTCTCGCTTTTTT
3[476]	AGTTCCTGAATAAACATAGGTCTCTCCCGCTTTTTTTTTTTTATGTGTGTTTGCTCGT
5[476]	GTTTTAGCGGACGTAACAGGGAGATAACTCTTTTTTTTTTTTTGAGTCCCATAAAGGC
7[476]	ATCGTCAAACTTTCCCATCTGCGGAAGTGTTTTTTTTTTTTTGCAATGTAAGAGGACT
9[476]	AGTTCCTGAATAAACATACACTCGAGTAATTTTTTTTTTTTTGTACCAAAACTTTCTT
11[476]	GTTTTAGCGGACGTAACATGGCGTAAACAATTTTTTTTTTTTGCGAGTTGAATTTCGC
30[492]	TTTTTGCTTCTTAATTGTTCCTTACACCGTTTTTTTTTTTTGGGGCGTACCCCTATTAGCTCTTGAGAAAT
45[472]	TTTGTATTAGACGTGGTTTTTTTTTTTTTGGCCCGACTTCCATACGATCATTAGATTT
47[472]	CCTCTGCGTTACTTCATTTTTTTTTTTTTTCAAAACGCACCCGTCATGCCCGGGTCGC
49[472]	TCAGAGCGCCAAGAGATTTTTTTTTTTTTCGCGAAGATCTTATTAGCTCTTGAGAAAT
51[472]	CACATCTCCGCACTTATTTTTTTTTTTTTGTCGGCAGAAGGCATACGATCATTAGATTT
53[472]	GCTATAGACCTAATAATTTTTTTTTTTTAACATGATCGAACCGTCATGCCCGGGTCGC

Supplementary Table 3 | DNA sequences of seed-S (G) (see Supplementary Fig. 67 for the strand diagram)

Colored sequences represent captures, 16 nt

Start	Sequences
	**Core strands**
32[135]	GTAATAAAGGGCGTCTATCAGTGCCGTAAAGC
48[353]	TTTTTTTTAGGACTCGAGCCCCATCTCCTTTTTTTT
34[271]	GACCCAGCCGCTGTTATTGCCAAATATGGTGT
46[355]	TTTTTTTCTATTGTTAAGACGTCACAGGTTCCGACCCTGCCTTTTTTT
49[232]	GGGGCAACGGAGAGCGCTATCCCGTGACTTATCTAGTTGTTATCCCTA
6[191]	CCGGAATATTAGGATTAAATTGACAGGTGGTATGTTAAATGCCCTCATAGTTAGCG
40[231]	TATTACGACCGGGCAATAGATATCGAGATCCACTGTCTTG
44[355]	TTTTTTTAGAGCCTCTTTGGTGTTGGCAGAAAACGG
40[350]	TTTTTTTACCCAGCGATCTAGGTCGCCCTTTTTTT
41[216]	ATGCGAGCGATGAAATCCGACCTTCTCTTCGGCTGAAAATTCGAACGG
26[198]	TTTTTTTGGGCGCCAGGGTGGTTAGGCGGTTTAAGCACGTATAACGT
2[135]	AGTCATTACCATTAGCAAAGTCACAATCAATA
49[320]	TTCCCTTACCGAAGCCAAAGCGCTGCTTTTTTTT
36[209]	CCCTAAAGGGAGCCCGGAGCGGGCGCGCCTTGCTGGTAATTTTTT
5[30]	TTTTTTTTCGGAACGAGGGTAGCATA
36[303]	GTTCATTCATATCTACGAGCCCTTAGGGAAGACTTGCTCGGGGATATG
16[103]	CATCGCAAGGATAAAAGACCATTAGATACATT
32[71]	GTGGCACACCCAGAAAGGCCGGAGACAGTCAA
37[144]	ACGCCGATTTAGAGCTCACCGAGAAAGGAAGG
38[95]	GATATTCAACCGTAAGTATCAGGTGAAAGCGT
36[350]	TTTTTTTTAGGGCTACTGAATAAGCCACATCGTAATGCATTTTTTT
38[55]	AAACAATAAGTCAGAAAAGCGCGAAACAAAGTACAACGGAGATTTTTTTT
6[71]	CCATGTTTATCAACAATAAGAGGCGGTACGAC
4[263]	TAACCGAGTTGCTCTTACTCATGGTTATGGCA
4[353]	TTTTTTTGCTGCGCCTTAGCAGCAGCCACTTTTTTT
33[128]	AAATAACCCCGCTGAAAAGGGCGAAAAACTGG
42[295]	AGCGTTCCCAACGCGCGGGCACGCGTTACTGT
47[104]	TTTTCCATTTGAATACCAAGTCTGATTGTTTGGATTAATCACAGAAATTAAAGTAA
34[111]	ACCAAACAAGAGAATCTTAGTTTTCCCAGTCA
35[176]	TTGCAACAGGAAAGAGGTGCCGTAAAGCACTAAATCGCTTATGCTAAC
34[239]	GATTTACGTCGAAATTTAACACCCTTCTTTCC
35[72]	AAATACCTCATATAGCTATTTGAACGGTAATCGTAAAAGG
41[264]	TGCGAGGAATTATATCTCGTGGTGTCACGAAG
33[184]	ACGCTGAACCATCACCCTAATACTGGAAAGCCATCTACTG
39[208]	GGTGTTAGGGCTCGGAATTAGTGTTGCGAATAATAATTTT
6[167]	GGCGCTTTTGATGATACCGTGGAAAGCGCAGTTATTTAACAATTGATA
44[79]	CGTACGTTATTAATTTTAACAGAAGGAGCGGA
40[167]	CCGTGAGTGAATTTATCAAAACTTTATGTAAACATGTAAT
23[20]	TTTTTTTCATCAAGAGTAATCTTGACAA
48[295]	CGACGATTACATGCCCGGGTAGTTACAACTGG
44[271]	GCGGTGGGCTTACATAAGCAGTGACCTATATA
41[136]	TAGATTAAGACGCTGATTGCCATCTTTTCGTA
44[207]	AAGAGGTAATCCAGCATTACATTGCTATCAAG

47[64]	ATCCTTTGCCCGAAAAATCATATTCCTGAACAACTAATAG
21[312]	TCGGCGTATAACAAGGGTCAGCATTTCGCGGC
47[264]	CCATGACTCTCAGCTAGGCGGCAGACTCTACGAGGGGGCTGACGTAGCTCTCTAAG
45[80]	ATTTTACCATATCGAATATACAGTGTTAATTAAGCAATAACAATAGCATTAACATC
41[296]	AGGGGTCCTCACCGCTTCTTTTCTACGGGTCG
23[232]	GCTGCTCCACCCATGCTCTTCATAGTGTCCTG
33[256]	CAATGTACGGGCAAGACAAGCGGTTCCTTCGT
35[224]	ACGAAAGCAATTGAGTAATTTGTCAGTTTTCACTGAAGCTCAGCATAA
49[280]	CACGGCTAAACTATAGCCTGAGTATGCATCATCTGGGAGT
35[272]	CCCAAGAAAATTCTCCAATCACGTGCAATTCA
4[199]	CCAGAGGTTTAGTACCTGAGACTTTAGACCAC
22[207]	TAATAAACAGGCATCACGCAAATTCCTGAGAA
26[55]	CTCATTCACTTGCCCTGTAACTTTGAAAGAGGACATTTTTTT
45[144]	CTGTATCCGGCTTGAAAACTTTTTACAAAGCGGTCCACGCGCTGGCAACAGCTGAT
20[263]	TTAGGTGCAGCGAGCTACTTACAGGTCCATCT
23[296]	AATGTGTGAGAGGAGGGCCATTGGATTGTCCA
32[255]	ACGCTGTGCGGTGGCGTATGGTAATTGCAGTCTCTCCAGCATTGGCTA
2[71]	AGTGTAGAAACCAATCAACATAGCCCTAAAAC
37[296]	GCCCTTCGAGGCTGCGCCCACGCGCTATACGGCTAAATGG
23[136]	ACTCTGACCTCCTGGTACCAATCGTCGCTATT
32[303]	AGTCGAATTGAAGCAACAACACACGGGTGAGT
24[347]	TTTTTTTCTACGTTTTTGAAATTGGGTC
43[112]	ATTAGAAGATGATGAAATGTTACATTTAACAA
10[311]	GAAAACTCACGTTAGTAGCAGCAGATTACGCT
26[119]	ACGCCATCAAAAAATTATTTAAATTGTAAAGG
15[192]	CCGTCTATCAGGGCAATCGCTAGCATTTTCAGGGATATGA
41[232]	TGAGTCGATAAATGTCGAAGGCAAAATGCATTGGGTGAGCAAAAACAG
37[208]	TGATCTGCCTCAAGGAATTCAATATTCTCATA
2[199]	GAACCCTCAGAACCGCCTATGCATACCAAAAT
35[22]	TTTTTTTCCGCGACCTGCTCCATGAAGTTTTAAATCAGGCGAAAGACTTC
18[71]	GAAAACCCTCGTTTACGCTATTATACCAGTCA
20[295]	CTGAGGGACACCACTTCAGGTAGTTGCCGTTG
38[350]	TTTTTTTGCCCTCACACATTGAGGTATCTTTTTTT
18[103]	AGATAATACTTTTGCGTTCTGTTTAGCTATAT
41[168]	AATAAGGCGTTAAATACAATAGGCCGAAATGATAAGTGCC
9[36]	TTTTTTTTCCATTAAACGGGTAGAACGGCTACAGAGGCTTTTTTTT
30[198]	TTTTTTTCATAAAGTGTAAAGCCACATACGAATTACCGC
34[143]	GAATCTAATCTATTTAATGAACGTGGACTCCA
2[167]	AGTTGCCCGTATAAACCAGTTGAGGCAGGTCA
24[271]	CTTCCACCTAAGTGTCACTCATGAGTTCGGCAGTCCAACT
16[175]	CAGACACCCGCAGATAGGGTTGAGGTTATCCGCTCACAAT
1[176]	ATTATCCTCAGAGCCAGAAAATCTGCGAAAAA
47[136]	CAGTACTAGAAAACATAGCGACAATTACCTGAGCAAAGCTTTGAATTAGTAATAAG
18[263]	TATGACATCTCGAATAGTATAAACGAGGTTCT
24[319]	GGCATCAGGGATGCGACCATCAGTCATCGACAAGTCAGAGTGTAGTAA
2[353]	TTTTTTTTGGTAACAGGAACTACGGCTACTTTTTTT
12[311]	ATTATCAAAAAGGAAACACCGCTGGTAGCCCG
2[103]	GAGGGTAATTGAGCGCTTGGCCTAATTTGCCA

8[191]	TTTTTAACAGAATAAATAGGGGCTTAATTGAG
20[167]	GCTAAAATCCTGTTTGGAGAATCGGCCAACGC
18[295]	TATAATCGGTGTATGGTGCCCTACTGTCGTAG
39[280]	CGCAGATGCCGTAATCGCTGAAGGGGCACTAGGCTAATCGAACGCCGCAACGAAAC
24[231]	GCCATAATCTACAGTCCGACCGTCGGAGTATCAGTGAGGCCACCGAGT
28[311]	TGCAGTTACCCTTTCAGTGAATATAAGAGATT
23[72]	CGGATGGCTTAGAGCTCCTGCGGAACAAAGAA
28[198]	TTTTTTTTCCAGTCGGGAAACCTCGCTCACTCTACTACAGGGCGCGT
15[36]	TTTTTTTCAGCGATTATACCACCGATATATTCGGTCGCTTTTTTTT
43[80]	ATTTACTTCTGAATAAGATAAAATTATTTGCA
43[208]	CGCTGAGTCAGGTTTTGCGGTATGAGCTCCGA
36[255]	GAGCTGGAGTAATTTTCTGTGGCAGCTTTAGGCTGGCTGACACGAA
25[320]	GTACCTCGCCTGTGGGTGCGCTTTTTTT
23[48]	GAACCGGAATTCTCAATCAATATCTGGTCTTTTTTT
39[96]	CAACCAGTGCCAAGCTTTCTCAGGCATACATAAAGGTCGAAAGAATTGAGTTAGCC
45[296]	CCGCGATCTAACTGTCAGGCCCGTTGTCACAACTTGTACCGTCTGTCG
45[256]	GACTGACAAAAGTTCCATATGGAGGTCGTCAAGACGCAAC
43[144]	ATCTCATAGGTCTGAGCGAAGGTTGGGTTATA
30[119]	GCGAGTAACAACCCAAAATCATATGTACCTTCAGTAAGTTGGGTAACGCCAGGATG
45[176]	TTTTAATACAAATAGAGCGTGCGTCGCAAAGATGAATCAT
42[191]	TCATATGCAAAAGAGTCTGTCAATCGAAGAAC
39[160]	GGACGCTTAATGGGTCTCTCGGCCAAGAACGTGGACTCAGCGGAACCT
42[327]	GAATCTGGTAGCTGCTTACGAATGGCCAGAAGATAAATAA
40[55]	ATTAGGTTCGACAACTCCGCCTGCAACAGTGCCACGCTGAGAGTTTTTTT
12[215]	AGCGCAGGTGTTTTCAATGTACCGTAACATTG
20[103]	GCAAAATCGGTTGTACTGTTGAAAAGGTGGCA
14[151]	CACCACCCTCAGAGGAAAACCATCGATAGTAA
20[71]	TACATTACGAGGCATAGACCTACGTTAATAAA
47[22]	TTTTTTTCCAGCAGCAAATGAAAATTGAAAGGAATTGAGTTTTTTT
41[312]	CAAGCAGAATGAGGGGCACTAAGGTCAAATACCTCGACTAATTCACTC
20[231]	ACTTTTATGGCTTGGGACGCATAAATACTTCT
12[279]	AGTTACATGATCCCCGTATTAATTGTTGCCAA
12[87]	GTTTTGAAGCCTTGCCGCACTCATCGAGACTG
22[231]	TGCTGCAAATCTAAGTATAACAGGGAGAGATA
21[30]	TTTTTTTGATGAACGGTGTACAGCAT
41[320]	GTGGAGAACGGCTATGAACGTTACTGCCTAAGGCGGTTACGATACAAG
19[312]	ATGATACTTAGGGCATCTATTCGGGACAACCG
20[135]	GAAGCCATTCAGGCTGGCCCGTGCATCTGCCA
45[320]	ACCGTAGATAGATCGGACAGAAGATCCCGCGATTTTTTTT
0[327]	GTATCTGCTTGAAGTGTTTTTCCATAGGCTTT
10[151]	CCAGAGCCACCACAGGGTAGCGCGTTTTCGAG
26[279]	GGCCGCTCTGGCAGTTGGGGGCATCACCTCAT
45[112]	CTTTTAAAATTAATGAGTGAATAACTCTCTGGTGCCGGAAATAGTATCGGCCTCAG
10[119]	ACCCAAAAGAACTCCAAAGCCCTTTTTAACGA
39[312]	TGATTACGCGTGCGGCATGTCCTTAGAACCGA
33[96]	GAGAGTCTGGAGCTTATGTAAAACGACGGTAT
45[56]	AAGTAAAGTTTGAGTATTTTCAACTAATGCAGAAGTTATTACAGGTAG
8[295]	TTCATCCATAGTTGCCGCACGTTTGTAGCCTCGTTTCTAGAGGTGTAA

33[288]	TTTAAGCTGGCGTGGAGTTTTTAGGCCTGTCT
12[183]	TTGTTCCAGTTTGGATAAGAGGCTGAGACACC
0[199]	TTCACGTTCCACCCTCCACCTCCCCTTGGTTACTAATTAACGTCAATCGTCTGAAA
47[200]	AATGCAGCTCAGTCATGTCGGCGGAATCATAA
25[296]	GGGCATTTCACAAGTATCCGACTAGCTCGAGA
10[183]	ATCAAAAGAATAGCTCGATTAGCGGGGTTGTA
22[343]	TTTTTTTTTTGAGTTACGACGCCGGCAGCCGTTCATAAACTATAATGTTTTTTT
42[103]	CAATAAAGGGTTAGAAAAAGAATTAGCAAAAA
38[159]	CTAAAGGGAGCCCTCAAGCGAAAGACCAGTCA
42[71]	ACCACTACATTTGAGGAGGAATACCACATTTG
12[247]	CCTTTTTCAATATATATTCGATGTAACCCCGG
33[160]	GCTAGGGCGCTGGCCTACCACACCCGCCGACC
16[202]	TTTTTTTATCCAGAACAATGCCGGAAGTTTTTTT
4[327]	CTATCGTCTGCACGAATGGAAGCTCCCTCAAC
34[79]	AAAAGATTAAGAGGAAGAAGAGTAATGTGTAG
8[79]	TTATCAAACAACATGTTCAGCTAATAGGAGCATATTAACACGTATTAA
42[135]	AATTAAACATCAAGAATTCCGGCACCGCTCGA
7[176]	GTCGAGAGGGTTGATATAAGTACACAGCTCAT
34[55]	ACCCTGACTATTAAATTGTGTCGAAATTTTTTTT
42[263]	TTTCATTCTCGGTAGTCACGTTGACTATGCGG
26[311]	CTGCAGGACTTAAGAGGATGCTCGAAGGTTCG
10[55]	ACGTAATGGGCACCAACATGCAGCGAAAGACAGCATTTTTTT
37[240]	GTTTTCTTCAAGATTGACCACAAGGAGTCTCA
48[327]	TTATACAGAGCAATACAAGAGGCACACACGCT
20[353]	TTTTTTTGGCGCCTTAGCTTAGACTT
2[295]	ACACCCACGCTCACCGAAATAAAGTATATATG
28[87]	GAAGTTTCATTCCCGTAACTCCAACAGGTAAA
8[135]	AATATTGACGGAAATTATTATTCATCGAGCCACCTTTTTTAATGGAAA
43[176]	GTTAGAAAAAGCCTGTAATTCTTACCAGTATA
2[231]	ATCAACAGTTTCAGCGCAGGAATGTATTTAGA
43[240]	ATCTTGGCGGAATAATCTGGAACATCCGGCAC
4[71]	TTTAAAAATAATATCCCCTAACGAACCACCAG
44[55]	CTTTACAAACAAATCTAAAGCATCACCTTTTTTT
43[28]	TTTTTTTAGTTGGCAAATCAACAAGCCGTCAATAGATAATTAGA
45[22]	TTTTTTTTTGCTGAACCTGCTGGCTGACCTTTTTTTTT
44[111]	TTTAGGTTTAACGTCATGGACGGATTCGCCTG
38[287]	GACCATGTCCGCACCGGAATGTCGCGGTACCT
15[296]	CCCATGAGAAATGAAGAGAGTTGGTAGCTCTA
30[55]	GATGGTTTTTAATCATCTTTTGCAAAAGTTACTTATTTTTTT
30[279]	GTTAGGCTCGAAACTCGTACTTTATGGGCTGT
7[30]	TTTTTTTTTGAGGACTAAAGACTTTT
22[295]	CAGCGCATAGCGAACAGCATCGAAACCGCGCC
25[232]	TCAACTCTCATGTACATTTGACATACCCCGGG
23[264]	ACCTCGGGTCCCCTGCGCTCTGAACCTACTCT
37[28]	TTTTTTTAAAATGTTTAGACTGGGTTCAGAAAACGAGCCTCTTT
37[80]	GTATCTAGCTGATAAAGATTTGAGAGATCTAC
34[207]	TAGCAAGTTTTTTGGGAAACCGATTTAGAGCT
37[112]	CGATGGTTGTGAATTCCGCTGCTGAATTGTCA

41[104]	AATCGCGCAGAGGCGAAGGAAACCGAGGAAAA
42[231]	ACGGATTTTAACATTGACCCCGTTTCCTCTTG
45[216]	GAGGATAAACCTTCGTAGTCCTGTTCTAGGAGTTACGACC
26[247]	GCGAAGTCCTACTAAACTCAAGAGTTTTGGAG
30[87]	ATTCTGCGAACGATTTTTCGAGCTTCAAAGAAGGTATTTTAAATGCAATGCCTTGA
6[295]	GGATGTAGATAACTACATGCAGCGATCTGTCTTCCAAGAGGAAGATTA
43[296]	TCCTACATGGATGAATTAGCTACGCCATCTTCATGGACGT
15[232]	GGCGGGAACTCATGAGCTCTCAAGGATCTAGC
30[311]	TCATGCGGCGTTACACTGCTATACAATAAAGT
6[263]	GTTCCAAGTCATTCTGATACTGTCATGCCATCATCGGACGCCTCTAGG
16[71]	TTTAAATAGCGAGAGGTGTGAATTACCTTATG
6[135]	GTCGAATTATCACCGTCGGGATTGAGGGAGGGAGGCATTTTTTTAGCT
23[104]	ATTCGCATTAAATTTTAACACATCGGGAGAAA
39[28]	TTTTTTTCGGAATCGTCATAAATATTCACATCTTTGACCCCTTTTTTT
42[350]	TTTTTTTCCATGATGCGAACACTGGGCTTTTTTTT
18[167]	ACTCAAAATCCCTTATATCTAACTCACATTAA
48[263]	TGTCTATGGTCTTGGCGGTGAGTGTGCGCAAG
0[103]	GATACCGATAACCCACGCGTCTTTCCAGACTAATCACCATTCTGACCTCATTGCCT
47[296]	GGTTAACTTGGATGTCGAGCAGGAAAACATGAAAACTCTTGCTAAAATGCTTATGT
28[247]	ACTAACGCAACGGGTCACTCTCAAGAACTCAT
35[304]	AAACATTCGAGATCTTAAACCAAAGTCATTTTGGACAAGT
46[79]	TTCTGTCCAGACGCAGCTATTATCAGATGATGGCAATTCATAGAAGGC
16[135]	ATACTGGCGAAAGGGGGTGGGAACAAACGGCG
34[343]	TTTTTGCCCCCTAACGGCGGATGCAGTCGTTTCCTGCTCACCTATCCTTTTTT
30[247]	TGTAACGACATACTAACTTTGATAGCTCGTTG
6[103]	ATAAACATAAAAACAGACGTTTTTGTTTAACGAAGTACCGATCTACAA
4[103]	TTAGAATTAACTGAACCGGTATTATTTATCCC
4[135]	CAGGGAATTAGAGCCACTCCAGCGCCAAAGAC
12[119]	ATTACGCAGTATGACAGAAACAATGAAATAAA
6[353]	TTTTTTTTGTAGGTATCTTCAGCCCGACCTTTTTTT
10[215]	AGCTGATACGCGAGGACAAACTACAACGCGAC
3[184]	CTCAGAACCGCCACCCTCGTGGCTCATGGGTG
19[184]	CACTTGCCAACTCAAAGCCCGCTTTTTTTTT
10[279]	TCATTCAGCTCCGCTGGTTCGCCAGTTAAACG
1[30]	TTTTTTTGAGGCTTGCAGGGAGTAAT
2[263]	CCATAATACCGCGCCAATCGTCAGAAGTAAGT
34[175]	ATACAAGTGTAGCGGTTGAGGTCACGCTGCGC
10[247]	GCGACACGGAAATCGACTTCAGCATCTTTATA
38[223]	CGAATAGACCTGACGGACATAGTTCCCGGATG
40[183]	TTACTTGAACGCTCAACAGTAGCGATTCGGTG
4[295]	TTTTCTGGCCCCAGTGAAGTTACCAATGCTTA
38[255]	GATCGGATAGAACAAGTGGTCAATGAAGTAGG
35[112]	CGATTGTCCTTAGTCGCCCTGGAGTGACTTTTGGAACGAA
38[127]	ATGTTCTTCTAAGCGTTGACAATGTCTGGCCA
40[335]	TCAGCTGTGAATTACGGGTAAACATTTTTTT
35[152]	CGTGGGCTGCGCGTAACCACCCCATGGCAAGTGTAGCCGG
32[355]	TTTTTTTCTAACTCTGGATAACTGGCCTACCGTAAAAAGGCTTTTTTT
0[71]	CCATCGCCTTTCCTTAAATGCGCGAACTGACTTTGAATCCGACAATATGCAAAGCG

30[151]	GTCATAGCTGTTTGAACAGTGCGGCCCTTCAGGCAAGAGTCCACTATTAAAGCGC
42[167]	TCTGACTACCTTTTTAAGAGAGTTGCAGCGGA
44[143]	TAACATAAATCAATATACAATTTTCCCTTAGA
0[167]	AAAAGGAGGCCTATTTCATTGACAGGAGGAGCACTAAATCGCAGATTCGAGCGGGC
26[87]	GTTTTAAATATGCAACTAATTGCTCCTTTCTC
24[55]	TATTCATTACGTAACAATAACCAGGCGCATAGCAAATATCAAACCTAG
28[119]	CTGTAGCCAGCTTCATGCCCCAAAAACAGAAC
28[279]	GAGTACATCAAACAGGTTCGATCAGTGGGCCT
8[103]	ATGAAAATAGCAGCCTTCAATATAACAAAAGGAAAGAAATTGCGTAGA
41[28]	TTTTTTTGAAGGTTATCTAAAATATCTTTCATGAGGAAGTTTTTTTT
39[216]	CATCTTACTCGTTAAGCAAAGATCGACCGATG
0[135]	CTTGCTTTCACCAATGATAAGTTTATTTTATAAGAAGCCAAGGGACATTCCCGCCA
16[343]	TTTTTTTTTTCCGGGAGAAGGCACGGCTTTTTTT
23[208]	CCGGCATTTAGCTAGACGACTATCTTGGTGTA
46[207]	CGTATTAGACGTATATCACATTGGGGCAGAAG
32[199]	GCCCACTAACGTCGTCGGCGAACGTGGCGAGAAAGGAACCACATTTTG
21[184]	TGTAGCAAATTAGTAATGCGTATTTTTTTTT
12[55]	ACGAAAGATACACTAAAATTAAAGGCCGCTTTTGCTTTTTTT
49[201]	TTTTTTTATCATTTCTCCCCAACAAATTTGC
18[231]	TTGGCCATTATCCGTGTCATACAGGGCTATCG
18[135]	AACGATCGGTGCGGGCATGGGTCACGTTGGTG
8[263]	ATGCTTTTCTGTGACTTCTTGATCAAATATAGAGCAAGGTGAAGACCT
0[295]	GGATAAACCAGCCAGCCCATTCGGATGAGATA
44[239]	GATAGGTAACACATGCGCGGCACCATTGATCT
19[30]	TTTTTTTGGAACCGAACTGACCAAGA
0[231]	CGACAACTAAAGGAATGGAAGTGCAAAGGTCT
28[55]	AAACACCATAAATTGGCAGGACGGTCAATCATAAGTTTTTTT
37[272]	GATTCGATTGATGCGTCGGCCTGACCTGCTAG
41[208]	GAGGAAGCTAAATTTTCGTAGACCCCACTGTAGCATTCCACAGATAGC
43[272]	ACAAGGTTTGGCAACGGGTGGTATCAATTGGA
6[231]	GTTTTTTGTCGTCTTTCGGAATTGTAAGCGTTGGGGGTATCAACGCAC
14[87]	TTTTGCACCCAGCTTTTCATTCCAAGAACTAT
17[212]	CTTAGGCACCGGCCCGATGACCGCAGACTAAGCCAG
28[151]	CCCCGGGTACCGAATGTCGGTGGGCACGAGTG
50[215]	GGGCTGATCACCGCCACGCCCAATTTTTTTT
12[151]	GCCGCCACCCTCAAATGACAGAATCAAGTGTG
17[312]	ATCCGGTTGTCACCACGCCTCCACGAGATATCTGTCAGCT
34[327]	TGAGGCTTGGGGAAGCAGACGGCGGAGACAAGGTAAGTGG
3[30]	TTTTTTTGGGATCGTCACCCTCACCT
11[200]	CCTATTCCGACCACTGATTCTGAGTTTCGTCA
2[327]	GCGAGGTACGACTTATGTCAGAGGTGGCGGAG
5[184]	TCACCGTACTCAGGTATGATAGTATACCATAA
37[176]	GTAAGGGAAGAAAGCGGTCAACGCTCATGGAA
33[64]	GATTGCATCAAAAGGCATTCAAAAGGGTGTCAAATGCTTT
39[320]	TGCCAGTGCGAATCCGCCTCAGTAAATTTGCTCTGAAATATCTTGCTA
8[347]	TTTTTTTGCTTACCGGATACCTGTCCGCCTTTCTCC
15[264]	CGCTCCTATCCTTCGGGGTCCTGCAACTTCCA
33[224]	TTAGACAGCACCGACGTAGACGCTACGGCGAG

22[263]	GTTCTTGCCTTATCGGGTGTCTGTCGCACTAT
0[263]	AGATGCTCATCATTGGTTAAAGCTCCTGTCGA
4[231]	ACTTTTTCTGTATGGGGAGTCCGCGCACATTT
44[175]	AAGAAGACAAAGAACGAGACCTAAATTTAATG
25[192]	GTGTTTTTATATGAAGTTTGAGCCCACGTCGC
26[151]	TGGTGCTTGTTACTGAGCTGACGCATTTCTTT
17[30]	TTTTTTTGCCGGAACGAGGCGCAACGCAAGCCAGAGGGGGTAATAGTTTTTTTT
46[159]	AACGCCAATGCTGATGCAAATCCAATCGCCCAAATACCGA
25[264]	AGGGCAGCACAACCACAACCCCCAAGTTTAAA
6[327]	TGTAGGTCAGCGTGGCGCTTTCTCATAGCTCACGCTTTTTTT
4[167]	GATAGTTTTAACGGGGCTCCACAAACAAATAA
33[22]	TTTTTTTTGTATCATCGCCTGATATGCGTCCAATACTGTTTTTTT
16[247]	CAAATGTAAATTCGGGCAGTGATTGGTCTCGTCCAAGTCGGCAAATGC
23[168]	GGCCGATTAAAGGGATGTTGTTAATTTCATCT
16[287]	CTTTTCTTACGTATGCCTCATCGTTGATGGCATCTTCTTA
10[87]	GGCGTTTTAGCGAAAGTTTCATCGTAGGAAGC
13[200]	TAACCAACCGGTGACGGAGGCAAGCCCAATAG
	**Square-captures-Z_1**
3[56]	AGGACGTGGTAAGTCTAATTTACGAGCATACCATTAAAAATACCGAAA
1[56]	AGGTCGTTAGACACCACGGCTGTCCACGCATATTTTGAATGGCTATTA
19[56]	GCAACACTATCATGCATGGGAAGAAAAATGAGATGTTGGAACCTTGTA
21[56]	AACGCCAAAAGGACTTTAACGGAACAACACTGGGACTGGGCCCGGACG
7[56]	AGGACGTGGTAAGTCTTGCAGAACGCGCCATAGCAAGCAAATCAGATA
24[87]	TAATTGCTGAATAGATTCATCAGTTGAGATTTATGTTGGAACCTTGTA
5[56]	AGGTCGTTAGACACCAAGTCCTGAACAAGTATATAAAACAGAGGTAAT
29[72]	TGTACCCTATAGTAGTGGAATATAACAGTTGAGGAGCGAACCAGACCG
31[72]	AACGCACGTAAATCATCGAGTAGATTTAGTTTATTTTTAGAACTCGCG
13[72]	ATCAAATCAAGATTAGTAAGGGTATTAAACCATAAGAGGGTGACTTGA
9[72]	AATCGGTATTCTAAGAGGAATCATTACCGCGCTAAGAGGGTGACTTGA
27[72]	AACGCACGTAAATCATACGAACTAAAGTACGGCAACAGGATTAGAGAG
11[72]	CAGACCTCCCGACTTGACCACAAGCAAGCCGTCAAGCTATGAAACCGG
3[88]	AGGGAGAAAGAACTTAAACAAAGTCAGAGCAAAAATAAACAGCCAGAG
19[88]	CATTATGACCCTGAAATTGGGGCGCGAGCCTGGCCACACGGATCATCA
7[88]	AGGGAGAAAGAACTTATTACAGAGAGAATGCCGAACAAAGTTACCAGA
5[88]	TTCTGATCAATTACAGGCATTAGACGGGACCGAATAAGAAACGATCGA
21[88]	AGAGCATAAAGCTAATCTACTAATAGTAGCATGGTATCTTTGGGAAGG
24[119]	TGTTAAATCAGCTTAAATCATACAGGCAAGGCGCCACACGGATCATCA
31[104]	CGCACACGCGTTGTTGTCGCGTCGGATTCTCCGATGTGCTGCAACTAG
29[104]	CGGTAGCAAAAGATCCTTTTCATCAACATTAACTCCCGGTTGATAATC
13[104]	GTTTTAGCAAACGTAGAAGAGCCCAATAATAACAGGCTTCGCAGTCCT
27[104]	CGCACACGCGTTGTTGTCATAATTCGCGTCTGCGCGAAGATTGTATAA
11[104]	AATGGCATGATTAAGAGCAAGCAATAGCTATCAATGTGAATTAGTCTG
21[120]	CAAAGCGCCATTCTCGGGGGACGACGACAGGAAGTGGACGGAACACGC
19[120]	TGTTGGGAAGGGCTCGGGCGCATCGTAACTTCCGCTATTGAACGGGTC
1[120]	AGGCGGACTAGGAAGTGGAAACGTCGAGGTGAAGAAACGCAAAGACAC
3[120]	GACCCAGATAGGACTCTCACCAGTAGCACAGCTCATATGGTTTACCTT
24[151]	TGGTGTAATGAGTGATCGCACTCCAGCCAGCTCGCTATTGAACGGGTC
5[120]	AGGCGGACTAGGAAGTCTTGAGCCATTTGACTGCGACATTCAACCAAT

7[120]	GACCCAGATAGGACTCATTCATTAAAGGTATAGCCCCCTTATTAGCGT
31[136]	GCGCCTTCGAGGACTAGATCCTGTGTGAAATTTGTTGTTCCAGCTATG
29[136]	CGAACACAGAGAACTCTAGGCTCGAATTCGTAAAAGCCATCTGTAAGC
13[136]	GAAGAACCGCCACCCTAGTCAGCACCGTAATCGAAATCAGCTAAGAGT
27[136]	GCGCCTTCGAGGACTAGTTCTCGATAAAGACGATGATATAGGGGCCTT
11[136]	AAACGGAACCGCCTCCTCATTGCCTTTAGCGTTAAGACCTTAATTCGC
19[152]	GTTCCGAAATCGGATGAGCTGCATTAATGGATGACTGGAAAAGATCGG
3[152]	TTCCGCCAAGGTGCGACCTTGAGTAACAGATTTGGCCTTGATATTAGA
5[152]	ATTCGTACGGGTAAGCTGTACTGGTAATATAGATTAAAGCCAGAAGAA
21[152]	GCCCCAGCAGGCGTTCCACCAGTGAGACGACGGGGCTGAGCCATGAGC
24[183]	TTTAGACAGGAACCCTTCACCGCCTGGCCCTGGACTGGAAAAGATCGG
13[168]	GACGAACAAGAGTCCACACTCTGAAACATGAAGTTCTCGATTTTTTTT
29[168]	TTTTTTTTTTTCTATCTTGCGTTGGTCGTGCCGTTGCTTTGACGACAT
11[168]	ATCACCGAGATAGGGTGCCTCCTCAAGAGAAGGGAGCACATTTTTTTT
27[168]	TTTTTTTTTTAGCTTCGCGGGGAGTTTCTTTTCTCGTTAGAATCACGT
36[63]	CCATAAATACGATAAAAACCATAATAAGAACTGGCTCTGAGGACTGGGCCCGGACG
32[79]	AAGAATACGTCTACAATTTTATCCCCGACAATGACAACAACAAGCTATGAAACCGG
25[72]	TGTACCCTATAGTAGTAAATAATGCTGTAGCTAGCTGATAAGAGGTCAACATTATC
1[88]	TTCTGATCAATTACAGCAGAGAGATAGTTGCGTGAATCTTACCAACGCAGAACCCT
36[95]	CCGGAGAGGCCTTTATTTCAAGTCATGGTCAATAACCCCAGGTATCTTTGGGAAGG
32[111]	ACAGAGATTAAGGCAACATATAAAATTTCTTAAACAGCTTAATGTGAATTAGTCTG
25[104]	CGGTAGCAAAAGATCCCAACATTTTTTAACCAACCACGTTAATATTTTAACAGTAC
46[111]	AGAATATATCAAACGCAATAATAACACGAAAAGTAAGCAGCAGGCTTCGCAGTCCT
36[127]	ACGACTTAGCTATTACGCCAGCCGCCGTAATGGGATATGAAGTGGACGGAACACGC
25[136]	CGAACACAGAGAACTCGAAAAACAGGGCTTAATGGACATAAATCATTTCCTTGCTT
32[143]	CACGACCACACGCCGCCACCAGAATGTATCGGTTTATCAGTAAGACCTTAATTCGC
46[143]	TTAGGCAGAAGATAATCAAAATCACAGATCGGCATTTTCGGAAATCAGCTAAGAGT
7[152]	TTCCGCCAAGGTGCGAAGCGTCATACATGGGAATTTACCGTTCCAGTAGAAGAGTC
1[152]	ATTCGTACGGGTAAGCTGCCCCCTCCTTTAATCCACCACCAGAGCCGCTTACATTG
36[159]	GGAAAGCCAAAGAATAGCCCGCGCCTAATGAGTGAGCATGGGGCTGAGCCATGAGC
32[175]	TGGATTATCGCCCAACGTCAAAGGCCAAAAAAAAGGCTCCGGAGCACATTTTTTTT
31[168]	TTTTTTTTTTAGCTTCTCCACACATGGGGTGCGCTTAATGCGCCGTCGTAGGGCGC
46[175]	AATCGCCACTCTCGGCAAAATCCCGGTTTGCTCAGTACCAGTTCTCGATTTTTTTT
25[168]	TTTTTTTTTTTCTATCTGCGGTACGCCAGAATAACGAGCGGGAGCTAACAAATATA
	**Square-captures-Z_3**
51[328]	TTTTTTTTTTTCTATCTACGACGGATGGCTTTTTTT
0[353]	TTTTTTTACTAGAAGAACAGTATTTGGGAGCACATTTTTTTT
50[247]	ATGTTATTTGTCGGACACTGCGAAATCAGCACATGTTGGAACCTTGTA
3[216]	AGGACGTGGTAAGTCTCTAAACAACTTTCCAGAACAAATAGGGGTGGT
1[216]	AGGTCGTTAGACACCAGAATAGAAAGGAAAAACGGATACATATTTCTC
5[216]	AGGTCGTTAGACACCAGTTAGTAAATGAAGATAAAGTGCCACCTAACA
48[231]	TCAGCCTCCGCACTCCCTGCATATTGGGGAATGGACTGGGCCCGGACG
7[216]	AGGACGTGGTAAGTCTTAACGATCTAAAGTCTTTGTTAAAATTCGCGT
11[232]	CCCGTTGAATACTCATGCCACTCGTGCACCCACAAGCTATGAAACCGG
27[232]	AACGCACGTAAATCATGGTTTTCTAATAGCGGACCTTGGAATTAGCAC
51[232]	TGTACCCTATAGTAGTAACCCGACTCCGCGAGATTCCAATAGACGAAG
31[232]	AACGCACGTAAATCATTATTTAACTAGGCAAAGCACCTTTTGCTATGC
13[232]	AAATATTGAAGCATTTCATTACCGCTGTTGAGTAAGAGGGTGACTTGA

29[232]	TGTACCCTATAGTAGTGATTGGTGTCGAGAGGCGCGCCTCTCCTTGCA
7[248]	AGGGAGAAAGAACTTAGGTGAGTACTCAAGTTGCCATTGCTACAGGCA
50[279]	TAAGTTCAGATCGGGATCGAGCGATATCCTCTGCCACACGGATCATCA
17[248]	AGGGCCACGCTAGACCCCCGCTAATAAGACGCGGTATCTTTGGGAAGG
19[248]	ACAGAGGTTGCCTTGGTAAAAGACTAGTGGCGGCCACACGGATCATCA
3[248]	AGGGAGAAAGAACTTACGTCAATACGGGAGTCCAGTGTTATCACTCTT
5[248]	TTCTGATCAATTACAGGTGTATGCGGCGAGTACATAATTCTCTTAAGG
21[248]	CAGGAGTGAGTGGTATTGGTTAGCTGGGGCCAGGTATCTTTGGGAAGG
11[264]	GCAGTTCCCAACGATCCTGCGGGAAGCTAGAGAATGTGAATTAGTCTG
51[264]	CGGTAGCAAAAGATCCTAATGTGACTAATCCGAAAACGAAAGATATGG
27[264]	CGCACACGCGTTGTTGGATTATCACGAATTTCACGAAGTCGACCTGGT
29[264]	CGGTAGCAAAAGATCCCCTAATTAAGAAACCCGTCTGCGATCATTCCT
13[264]	TGGCCCATGTTGTGCAGCTTATCCGCCTCCATCAGGCTTCGCAGTCCT
31[264]	CGCACACGCGTTGTTGTTGGTCGCGCAAACGGCGTGCCTAGCTTTTTC
50[311]	ACCCCTAGGCCGTTGCCGCTTACTACCTGGGACGCTATTGAACGGGTC
7[280]	GACCCAGATAGGACTCTGACTCCCCGTCGTCTCAAGAAGATCCTTTGA
5[280]	AGGCGGACTAGGAAGTGGAGGGCTTACCAGCAGAGGCACCTATCTGCT
17[280]	AATTATCAGAATTTCGAGGTTGACGCGACCAGAGTGGACGGAACACGC
19[280]	ATAAGTCGGGGTTCATAGTATGCTATTTTATACGCTATTGAACGGGTC
3[280]	GACCCAGATAGGACTCTGATACCGCGTGAAACCTTGGTCTGACAGGCG
1[280]	AGGCGGACTAGGAAGTGATTTATCAGCAAAAATTTTAAATCAATCAAG
21[280]	CCTGGGTCATGCATTGAACTGTATTCTGTCCGAGTGGACGGAACACGC
51[296]	CGAACACAGAGAACTCTTATCCACCTAACAAACTCGAACATGTCCGTA
27[296]	GCGCCTTCGAGGACTAAGAATGCCCACAACATCCAATTAGGTATAGTA
11[296]	ATCAAGGGATTTTGGTAACGGTGGTTTTTTTGTAAGACCTTAATTCGC
29[296]	CGAACACAGAGAACTCGTCACGGTGATCAGAGCCTCGAGTTTGTGCGT
13[296]	AGTATCTTCACCTAGAGTGCTTGATCCGGCAAGAAATCAGCTAAGAGT
31[296]	GCGCCTTCGAGGACTATTGACTGAGCACAGTAATAAATGCTGGCCGTC
20[327]	ACAGGATGCCAGTAAGTATGATTCTAGGGATAGGGCTGAGCCATGAGC
5[312]	ATTCGTACGGGTAAGCGGGCTGTGTTGAGTCCCATAAACCCGACAGGA
3[312]	TTCCGCCAAGGTGCGAGTAAGACATGTAGGCGTCCTCCGCCCCCCTGA
13[328]	CGAGCATCACGCTCAACGCCACTGTCCGGTAAGGAGCACATTTTTTTT
27[328]	TTTTTTTTTTAGCTTCCTGTCCCCGAGTGGCCCACACCTAGCGAAGGC
11[328]	CTATAAAGTTTCCCCCCCCCCCGTCAGTTCGGGTTCTCGATTTTTTTT
31[328]	TTTTTTTTTTAGCTTCCGGAGTGGGACACACACTACCTATTTTTTTTT
29[328]	TTTTTTTTTTTCTATCACGTCCCAGCCGTATGAAGCGGAGTTTTTTTT
19[212]	AAACGCGATGTAGCCGTGGGTCTGCATCTATGGCTAGGACTGGGCCCGGACG
21[212]	AGAGCATGGATCGCAACACGGAACGCCTCTTCCGAGATGTTGGAACCTTGTA
50[347]	TTTTTTTGTTTCTGAAGTGATGAGGTCACGGAAGGTGACTGGAAAAGATCGG
14[347]	TTTTTTTCGCGTTGCTGGCGGTGGCCTATTAGCAGAGTTCTCGATTTTTTTT
39[240]	CGCTAAGAGTTATTGTAGTAGGAGAAAACGTTCTTCGGGGCAAGCTATGAAACCGG
46[239]	AATCCCCAAATCGCAAAAAAGGGAAGATACTTTCACCAGCTAAGAGGGTGACTTGA
1[248]	TTCTGATCAATTACAGAGAACTTTAAAAGAGTTCCTCCGATCGTTAGGCTCGCGAT
39[272]	TGTTCCTACGGTTAGCCCAACTGTCGGAAGGGCCGAGCGCAATGTGAATTAGTCTG
46[271]	TGGTGGACCGTCTCGTCGTTTGGTGATTAGTTTGCGCAACCAGGCTTCGCAGTCCT
39[304]	CCGTTGGTTAAATTAACAAAAGGCGAAGCCAGTTACCTTCTAAGACCTTAATTCGC
46[303]	AATGCTCCATTGTCTGACGCTCAGCAAGCGCAGAAAAAAAGAAATCAGCTAAGAGT
1[312]	ATTCGTACGGGTAAGCCAGAGTTCGCTCTGCTCAGCAAAAGGCCAGGACTGGTCGT

35[320]	GAGGGCGTTTATCCGCCGTCAATCGCCTACAAGCCACAATGGGCTGAGCCATGAGC
7[312]	TTCCGCCAAGGTGCGACTTCGGGAGTTCGCTCTGGGTGCGCTCTCCTGGTCTTTCT
18[340]	TTTTTCCAGATCTACGCCCCGCTCGTCAGTTCTTATGAGTCTTAGGACTGGAAAAGATCGG

Supplementary Table 4 | DNA sequences of the square SST sublattice (see Supplementary Fig. 8 for the strand diagram)

Start	Sequences
	**SSTs-a**
1[31]	CCCAGTCCATTAACAGCCTACAGGACACAGTC
3[31]	TCCAACATTACATCAGCGCTCTAGAACGAGGC
5[31]	AACGCTGCTTAATAGCCAAAGAAACAACGCGG
7[31]	CCCTTAGTAGACGGTAACCGCACGGAAATGAC
9[31]	CGCAATACGCAGTGAGTACCACTCAATCCAGT
11[31]	TCACACATTGAACAAGGCTGGGCTGAACTAGT
13[31]	ACCATTTGCCTCAGATGCTCATCATGGTGTCT
15[31]	ATCGGGTTGACAGTGGGTGAAAGCAGACTTAC
1[47]	GACCGAACCGTCCGGGATGTCACTACTACTAT
3[47]	ACCCACGTTACAAGGTGGCGGTTGATGATTTA
5[47]	CCAACGGGAACCACCTATCCAATGGCTAGATC
7[47]	ATCCCTGACGACGTTTATTTCCAGCTGTTCCG
9[47]	CGTGTCAGCTCCTGTTGCTTCCTCTATGAACC
11[47]	CTTGCGCCTAACCGGGAGTGAATCGAACAATA
13[47]	CCCTCTTAATGTACCACACGTCCTGGCTTGAC
15[47]	ATAGCTTGGCAGCGGAAACGACCTTTCTACTG
1[63]	AAGATACCGAGAGGGTCGTGCGTTCCAAGATA
3[63]	CGTGTGGCCGTTATCGAGGGTACACAAATACC
5[63]	ACAATGGTGAGGTCGAAAGAAGCTCCTTCTGC
7[63]	GCATACGGATTAACTGACAGGAGGCTTGCCTT
9[63]	CGTGGCATAATAGTGCGAATGGTCGGTCAACT
11[63]	ACTTTAATAAAGGTAAGTAAGGGCCCGCCGCC
13[63]	CGCTCGGATCAAGTCACAGGTGTGCTGTAATT
15[63]	CGAAGCAGCCGGTTTCAGAGTGGTTAAGTTCT
1[79]	TTCTAGGGCCTTCCCACTCAGACTGGATCTTT
3[79]	AGCGCGTATGATGATCAGGTACTGCAACAACG
5[79]	CCTCTAAGGCGGATAGAGCTAGCAGTTCCTGT
7[79]	TGCCGATGTTGTATCACTGTACCGACCGAGAT
9[79]	AGGGAATGGAAGATGTCGCTAAAGTTTGGGCC
11[79]	ACGTTTCTGTGCGCCTGATCCTAGCTTGAATG
13[79]	GAAGCCTGTTGTCCATTTCTCCCTCCCTCAAC
15[79]	TTCACATTGCGCCGAAGATCAGAATGGATCGG
1[95]	CGTCCACTATCTATATCGTGTGCGTGTCCGGT
3[95]	CAATAGCGACCTTACCTGCTACCGTCGACAGA
5[95]	CGCTCACAGCTCCGGGTTTGATTGGTATCAAA
7[95]	TAACGTCCTATCGTGATTAGCTGGGACTGCCC
9[95]	TCGAGCGACACCGCAATAGATAACTACGCGAA
11[95]	GCTTTGACGAAAGCATGAAGCAAGTTCGCTGC
13[95]	GCAGACTGAGGACTGCTCCAATCGACTTCCTA
15[95]	CCGGGTGTCAGACTAAGGCAAAGTGAGTCCTA
1[111]	CACGGGATGCGTGTTCAAGGTGTTGAGTTCTC
3[111]	ACGCTCAAGACCCGTTGTAACCGATAGTCCTC
5[111]	CGTGCACGTGCGAAGTATATCCATGAGTTGCC
7[111]	CGCACCTACGTGGTAGTCGGCACTGATGATAT
9[111]	GGCCACGTATTTCAAATCCGAAGCGTCTGTCC
11[111]	TGAAGTACCTCAGACACATAAGGCGAGGCCTG
13[111]	CTGATTTCAACTGGAATCTGGGTCCGGTTGCC

15[111]	AGGTCTTAACTTCGGAGTCCGCCTCTAAACGC
1[127]	CTCAGCCCTTAACAGAGAAGGCGCAGCCTATA
3[127]	TTCCAGTCGCGTTCTTTGTGTTCGGCCAAATT
5[127]	TTGGGATAGAACGGTGCGAACGTATACGTCCG
7[127]	TCTCGTCACGGGTCCATGAACGATATCAAGCC
9[127]	TTGCATGGCACCGCATATGCTTGTAAGTGCTT
11[127]	CCCGCTGTCCCTGAATCTCTCCTTTGAACAGA
13[127]	ATCACTCCACTCTTAGTGCCGTTCGCTTACCC
15[127]	CGTTCGTAGCGAATTATCCACTCGTCGCACCT
1[143]	CGCCGATCGCTCATGGGAACGTACGATAGAAA
3[143]	CTCCTAGCCCGATCTTGCAGCGATGAAGCTAA
5[143]	GCGAGAAACCACTAGGCCGTATGTGACTCGTT
7[143]	TCGTCACAACAACATGCGTACTTGGAGCCTGA
9[143]	TAACAGTTATACCGTCCCATGTCCGCGCAAGG
11[143]	TTACAAGAAGGAACAGGTGGCTCCTCAGCGTG
13[143]	TCGAGAACACCATCGATGGCGGAACGCTTTAG
15[143]	TGTGCTCCATCGTCCAGTACGAATGTTCTGCC
	**SSTs-a***
0[23]	CCTGTAGGTTTTTTTTTTTTTTTTCCACTGTC
2[23]	CTAGAGCGTTTTTTTTTTTTTTTTATCTGAGG
4[23]	CGTGCGGTTTTTTTTTTTTTTTTTCTGATGTA
6[23]	TTTCTTTGTTTTTTTTTTTTTTTTCTGTTAAT
8[23]	GAGTGGTATTTTTTTTTTTTTTTTACCGTCT
10[23]	AGCCCAGCTTTTTTTTTTTTTTTTGCTATTAA
12[23]	GCTTTCACTTTTTTTTTTTTTTTTCTTGTTCA
14[23]	TGATGAGCTTTTTTTTTTTTTTTTCTCACTGC
0[39]	CAACCGCCGACTGTGTACTAAGGGAAACGTCG
2[39]	AGTGACATGCCTCGTTGCAGCGTTAGGTGGTT
4[39]	CATTGGATGTCATTTCATGTGTGACCCGGTTA
6[39]	CTGGAAATCCGCGTTGGTATTGCGAACAGGAG
8[39]	GATTCACTACTGGATTAACCCGATTCCGCTGC
10[39]	GAGGAAGCACTAGTTCCAAATGGTTGGTACAT
12[39]	AGGACGTGGTAAGTCTATGTTGGAACCTTGTA
14[39]	AGGTCGTTAGACACCAGGACTGGGCCCGGACG
0[55]	AACGCACGTAAATCATCAAGCTATGAAACCGG
2[55]	TGTACCCTATAGTAGTTAAGAGGGTGACTTGA
4[55]	CCTCCTGTGATCTAGCACGTGGGTCGATAACG
6[55]	AGCTTCTTCGGAACAGGTTCGGTCACCCTCTC
8[55]	GACCATTCTATTGTTCTCAGGGATCAGTTAAT
10[55]	GCCCTTACGGTTCATACCCGTTGGTCGACCTC
12[55]	ACCACTCTGTCAAGCCGGCGCAAGTTACCTTT
14[55]	CACACCTGCAGTAGAACTGACACGGCACTATT
0[71]	CAGTACCTTATCTTGGCCGTATGCTGATACAA
2[71]	AGTCTGAGGGTATTTGACCATTGTCTATCCGC
4[71]	TGCTAGCTAAGGCAAGATTAAAGTAGGCGCAC
6[71]	CGGTACAGGCAGAAGGATGCCACGACATCTTC
8[71]	CTAGGATCAGTTGACCCTGCTTCGTTCGGCGC
10[71]	CTTTAGCGGGCGGCGGTCCGAGCGATGGACAA
12[71]	AGGGAGAAAGAACTTAGCCACACGGATCATCA
14[71]	TTCTGATCAATTACAGGGTATCTTTGGGAAGG
0[87]	CGCACACGCGTTGTTGAATGTGAATTAGTCTG

2[87]	CGGTAGCAAAAGATCCCAGGCTTCGCAGTCCT
4[87]	CCAGCTAAACAGGAACTACGCGCTGGTAAGGT
6[87]	CAATCAAAATCTCGGTCCCTAGAAATATAGAT
8[87]	GTTATCTACATTCAAGCATCGGCATCACGATA
10[87]	CTTGCTTCGGCCCAAACTTAGAGGCCCGGAGC
12[87]	ACTTTGCCGTTGAGGGAGAAACGTATGCTTTC
14[87]	CGATTGGACCGATCCACATTCCCTTTGCGGTG
0[103]	TCGGTTACACCGGACAGGACGTTACTACCACG
2[103]	AACACCTTTCTGTCGATGTGAGCGACTTCGCA
4[103]	ATGGATATGGGCAGTCGTCAAAGCTGTCTGAG
6[103]	AGTGCCGATTTGATACTCGCTCGATTTGAAAT
8[103]	GCCTTATGTTCGCGTAACACCCGGTCCGAAGT
10[103]	GCTTCGGAGCAGCGAACAGTCTGCTTCCAGTT
12[103]	GACCCAGATAGGACTCCGCTATTGAACGGGTC
14[103]	AGGCGGACTAGGAAGTAGTGGACGGAACACGC
0[119]	GCGCCTTCGAGGACTATAAGACCTTAATTCGC
2[119]	CGAACACAGAGAACTCGAAATCAGCTAAGAGT
4[119]	ATCGTTCAGGCAACTCTTGAGCGTAAGAACGC
6[119]	TACGTTCGATATCATCATCCCGTGTCTGTTAA
8[119]	ACAAGCATCAGGCCTCTAGGTGCGTGGACCCG
10[119]	AAGGAGAGGGACAGACCGTGCACGCACCGTTC
12[119]	CGAGTGGAGGCAACCGGTACTTCAATTCAGGG
14[119]	GAACGGCAGCGTTTAGACGTGGCCATGCGGTG
0[135]	ATCGCTGCTATAGGCTTGACGAGACATGTTGT
2[135]	GTACGTTCAATTTGGCTATCCCAACCTAGTGG
4[135]	ACATACGGGGCTTGATACAGCGGGCTGTTCCT
6[135]	CAAGTACGCGGACGTACCATGCAAGACGGTAT
8[135]	GGAGCCACAAGCACTTTACGAACGTGGACGAT
10[135]	GGACATGGTCTGTTCAGGAGTGATTCGATGGT
12[135]	TTCCGCCAAGGTGCGAGACTGGAAAAGATCGG
14[135]	ATTCGTACGGGTAAGCGGGCTGAGCCATGAGC
0[151]	TTTTTTTTTTAGCTTCGGAGCACATTTTTTTT
2[151]	TTTTTTTTTTTCTATCGTTCTCGATTTTTTTT
4[151]	TTTTTTTTAACGAGTCGCTAGGAGTTTTTTTT
6[151]	TTTTTTTTTCAGGCTCGATCGGCGTTTTTTTT
8[151]	TTTTTTTTCACGCTGATGTGACGATTTTTTTT
10[151]	TTTTTTTTCCTTGCGCTTTCTCGCTTTTTTTT
12[151]	TTTTTTTTCTAAAGCGTCTTGTAATTTTTTTT
14[151]	TTTTTTTTGGCAGAACAACTGTTATTTTTTTT

Supplementary Table 5 | DNA sequences of the kagome SST sublattice (see Supplementary Fig. 14 for the strand diagram)

Start	Sequences
	**SSTs-a**
1[31]	GCTAACAATGAGTATGGGCTCCGCTATCGAGA
3[31]	TCTCTGCGTGACCGGATTTACACCTATGGATC
4[16]	AGATATACAATAGTGAGACACGGCGGCAGACG
6[16]	AGTCGACCTAGGTAAACTCCTTGCCAAGTTCC
9[31]	CATGCGTGCTACCAAGAATTCCTATGCGCTTT
11[31]	TCAAGAACCGAGTAACTCGAGTCATCCATGCG
12[16]	CTCGGGATCCCATTTGACGGTCCTATATGCTC
14[16]	TTCGCAGAACTGATCCAACCGGTACTACAAGG
1[47]	GTACTTGTGGACTTGGATGTTAGGTCCCGTGT
3[47]	ACCTGCCTAGCCAGTAACCCTAATGATGCTTC
9[47]	ATTGACGACCATGTACTCGATCGACTGGTTGC
11[47]	GAGAAGTACTGCTAATACCATGGGCCCACCTG
1[63]	TACCAACCATCACAACGGAAAGGGCAAGTCAT
3[63]	TCTGTAGACAGTCTGCGGTATGGCCTAGAGTG
4[48]	TCTACGGCGTGTACGACCTCTACTTCACGAAG
6[48]	GCTCAGTGTATAAGACAAACCTGGTGCTGGCA
9[63]	TGACACGAATCCTTCGAACAGGCCTACGACTA
11[63]	GCTCTCGTAACAACCGTTGCAGACCCAATGTA
12[48]	TTCAGAGCCAGAAACTGGCATTACCCATGCAT
14[48]	AATCGGGACACTCCCATCCGCATTCCAATAAC
1[79]	TGGGTTTATGAAGGGTTCTGGGCAGCAGTTAC
3[79]	CAGATGCCCTGTGATTCACGCTCTTCACTAGC
9[79]	CTCCAGTGGGACTCTTGAGACGAGGAAGTGAA
11[79]	CTTTGTGAGCGTCGAGAAGCGACAGGAAAGTC
1[95]	CGTCCACCACCTCTTGGCTGAATACTTGAAGC
3[95]	AGTGGTAACGTATGCCTACATCCCAGCGGTAG
4[80]	CTCCACGCTTCACCTCAACAGGGAATTGGTTC
6[80]	ACTTTCATTGCTAAACGCGAACCGCATCGTGT
9[95]	AGGTTGCGTAATGTGCTAACTCAGCAAGGTGC
11[95]	GGGTGTGTGCACATCTCCTAAGATTTGCTGGG
12[80]	TATAAGGTTCAAACACTGCGAGATCCGGCCCT
14[80]	TCCAACACAGTGAGCTAACCGAACGTTAGCGA
1[111]	CTAGGTGGAGATAATAAGATGGCCGGGCACGT
3[111]	CACATTGTGAATGGTGGGTACGGAATGAGCCG
9[111]	GAGTCCCTTCCACAGAGTAATCATGTCGCGGT
11[111]	ATGGTCTAGTCAATACGGGTGGATGCCGTCAC
1[127]	ACTAGGTTCTCGTCCAACGCGCCATGGAATAA
3[127]	GATGGCGACTAAGCTTACGTCGCGTCGTTACT
4[112]	TACATGTTCACCGGGCTGGTGGTTTAACCTGA
6[112]	ATCACAGACTTCGCCAGCCATGACCACATACA
9[127]	GGCAAAGGTGCGTCAGTACGGTCTAAACCTCT
11[127]	TGGATTGGGCGGTCATAACGAGGCGTCATTAA
12[112]	ACCGAGGACCTGTGTCTTCAATTTAGCCCTGG
14[112]	ATGGGTTCCGACTCGGACGGCTTAAGATCAAC
1[143]	ACATCTGTCTGTTGCGCTGGTAGTGGGTTGAT
3[143]	GAAGTTGTGTTTACAGAGGCGCCACAACTGCG
9[143]	AAGGACTCCCTACACTCGGCGTTTCGAGAGAA

11[143]	TGATGTTGCCCGCAGTCAGTGACCCGATATTG
	**SSTs-a***
0[103]	TCCGTACCGCTTCAAGTATTCAGCGCTAGTGA
0[151]	TTTTTTTTCGCAGTTGTGGCGCCTTTATTCCATGGCGCGTCGGCTCAT
0[39]	ATTAGGGTTCTCGATAGCGGAGCCTTTTTTTT
0[71]	AGAGCGTGATGACTTGCCCTTTCCGAAGCATC
2[103]	GGCCATCTCTACCGCTGGGATGTAGTAACTGC
2[151]	TTTTTTTTATCAACCCACTACCAGAGTAACGACGCGACGTACGTGCCC
2[39]	CCTAACATGATCCATAGGTGTAAATTTTTTTT
2[71]	TGCCCAGACACTCTAGGCCATACCACACGGGA
4[103]	TCCCTGTTGAGGTGAAACACACCCGTATTGAC
4[119]	AACATGTAGAACCAATACAATGTGAAGCTTAG
4[135]	AACCACCAGCCCGGTGCCAATCCAACTGCGGG
4[151]	TTTTTTTTTCAGGTTAACAACTTCTTTTTTTT
4[23]	GTATATCTTTTTTTTTTTTTTTTTTCCGGTCA
4[39]	GCCGTGTCTCACTATTGTTCTTGAATTAGCAG
4[55]	GCCGTAGACGTCTGCCAGGCAGGTGCAGACTG
4[71]	AGTAGAGGTCGTACACACGAGAGCCTCGACGC
4[87]	GCGTGGAGCTTCGTGAGGCATCTGGGCATACG
6[103]	CGGTTCGCGTTTAGCACGCAACCTTCTGTGGA
6[119]	TCTGTGATACACGATGCCACCTAGTGGACGAG
6[135]	GTCATGGCTGGCGAAGCCTTTGCCAGTGTAGG
6[151]	TTTTTTTTGTATGTGACAGATGTTTTTTTT
6[23]	GGTCGACTTTTTTTTTTTTTTTTTCATACTCA
6[39]	GCAAGGAGTTTACCTACACGCATGGTACATGG
6[55]	CACTGAGCGGAACTTGACAAGTACGTTGTGAT
6[71]	CCAGGTTTGTCTTATATCGTGTCAAAGAGTCC
6[87]	ATGAAAGTTGCCAGCATAAACCCACAAGAGGT
8[103]	ATCCACCCGCACCTTGCTGAGTTAGACTTTCC
8[151]	TTTTTTTTCAATATCGGGTCACTGAGAGGTTTAGACCGTAGTGACGGC
8[39]	CCCATGGTAAAGCGCATAGGAATTTTTTTTT
8[71]	TGTCGCTTTAGTCGTAGGCCTGTTCAGGTGGG
10[103]	ATGATTACCCCAGCAAATCTTAGGTTCACTTC
10[151]	TTTTTTTTTCTCTCGAAACGCCGTTAATGACGCCTCGTTACCGCGAC
10[39]	TCGATCGACGCATGGATGACTCGATTTTTTTT
10[71]	CTCGTCTCTACATTGGGTCTGCAAGCAACCAG
12[103]	ATCTCGCAGTGTTTGATTACCACTCACCATTC
12[119]	TCCTCGGTAGGGCCGGTAGACCATATGACCGC
12[135]	AAATTGAAGACACAGGTCGCCATCCTGTAAAC
12[151]	TTTTTTTTCCAGGGCTCAACATCATTTTTTTT
12[23]	ATCCCGAGTTTTTTTTTTTTTTTTTGTTACTCG
12[39]	AGGACCGTCAAATGGGCGCAGAGATACTGGCT
12[55]	GCTCTGAAGAGCATATTACTTCTCCGGTTGTT
12[71]	GTAATGCCAGTTTCTGTCTACAGAAATCACAG
12[87]	ACCTTATAATGCATGGTCACAAAGAGATGTGC
14[103]	GTTCGGTTAGCTCACTGGTGGACGTATTATCT
14[119]	GAACCCATTCGCTAACAGGGACTCCTGACGCA
14[135]	TAAGCCGTCCGAGTCGAACCTAGTCGCAACAG
14[151]	TTTTTTTTGTTGATCTGAGTCCTTTTTTTTT
14[23]	TCTGCGAATTTTTTTTTTTTTTTTTCTTGGTAG
14[39]	TACCGGTTGGATCAGTTTGTTAGCCCAAGTCC

14[55]	TCCCGATTCCTTGTAGTCGTCAATCGAAGGAT
14[71]	AATGCGGATGGGAGTGGGTTGGTAACCCTTCA
14[87]	GTGTTGGAGTTATTGGCACTGGAGGCACATTA

Supplementary Table 6 | DNA sequences of the honeycomb SST sublattice (see Supplementary Fig. S19 for the strand diagram)

Start	Sequences
	**SSTs-a**
1[29]	GTAGCAGCGTTACCATGGATTCGCGTATTGAATTAA
3[29]	TATGGACTCTGCGAAAGTTAGTGTCGTCTATCAACG
5[29]	CCTTCTTCCTCGTATCCGTGTAAAGACACATCAGTT
7[29]	GTCCATCTACTGTCCGGGTTTCTGTACTCTTTGTAC
9[29]	CAGTTAGAATTGTTTATGCGTGGAGGATGGCGTAAG
11[29]	TCTTATGAATGACAGGAGGCGAGGACTTTTGATTGT
13[29]	CATACATCGAACATCTTTGTACGACCTGCAAACTCC
15[29]	GCTTATTGGAAAGACTGGTTCTCACCAATTTCGGTC
17[29]	TGTGCTATTCAGTCGTTTGTCGCGTACCTATAACTT
1[46]	AAACTACCCTCCAAATGTGGTCCTTATCACCACTGG
3[46]	AATACACGGTGACTACCTGCTCACACTGATTCCAAC
5[46]	ACCTTCCACAAGGTCAGTAACTTCCCTACTTAGCAG
7[46]	ACGGCCTATGTATTAGTGGTCTAGCTGATAAGCCGA
9[46]	AGAGAGTAAGTGGCTTTGTAGGTGCGTGTAATTCCC
11[46]	AGCACTACTTCAGCGTCTAAACAGTAAAGGCCGTAG
13[46]	AGCCAGTAGAGTATCCGCAAGATAGTCTAAGTCCGA
15[46]	AGTTGTACTAAGCCTCGGATATCCCTGACACCTGTA
17[46]	ATACTTACCCTTCAGAACATCCCACATTCCCTAGGC
1[64]	TCGCGCTCAATCTTACATCATATTGGACTCTGGATC
3[64]	AACCTCCGGATCAAATGTCCAAACTACATACGGTAC
5[64]	TCGCATGTTTGCCTTATACCCGAATAACAGTACTGT
7[64]	TGGTACCTCTCCCGAATGATCACACACTACAAGTAA
9[64]	GGAACCAACCAGATACGTTAGTATGGGGAAACGAAA
11[64]	TGAAGTTAGGAAGTCGCGATCACTAATCTTGAAGGC
13[64]	CCATTACGTAGCCAATGTGTACAGCAAACTCCAATG
15[64]	TTCTGCAAGTGTTCAGTTAACAAACGCGCATCTTTA
17[64]	CAGGATCAAGAGACATTAGCATTGCATACTCCCGAA
1[81]	CAGATAGGTTGCTGTTAACCTTAAAGGGTTCGTGCC
3[81]	CCAGTATCGTTTACGTATCGACGGCTCACTAAAGTG
5[81]	CCCACTTTCACAGTTCCGAAATGGAGCTTCGGTATA
7[81]	CCGAGCGTCTTGACCGTTATAGAAGGAAGCATATCA
9[81]	CCTACAGTGTCTGAGAGCCCATACCTGAATCGATTT
11[81]	CGATCGGGTGCCCATCTGATCTAAGATGTAATCGTT
13[81]	CGGTGCATCTGAAACCTACTTCTTAGTGTGAACCTC
15[81]	CGTACCCAGTCCCTCAACCCATATAGAAAGTTCATG
17[81]	CTTGACACAACTGGGAGGTATTGAGTGCCCTAAACT
1[99]	CGATTTCGATTATGTCGGATCGGTAAGGTTTACGGT
3[99]	CCAAGCTGTGATAGGCTTGATTTGAATTAGCCGAAC
5[99]	GTACAGTAGTATGTCGTGCCCTCGCGTATCTACTAA
7[99]	CTATCCCTCACGGGACCATATTGTGTAGGGTATCTC
9[99]	GGGACAGCGATACAATAAAGCGGAGGTGTTCTATAC
11[99]	CCTAGCAAGTCTACCGTAAGTGTCATTCACACAGTG
13[99]	GCAGTCCATCTCCGAGGGTCGGATAATTTTATTCTG
15[99]	GTGGCTATATGCCCGTAAAGGACTATGACAACATCC
17[99]	GCAGGTAAATCTAGTCAAGCATCGCTTTACTCAGCT
1[116]	GAAAGGAATCGGTGTACCATGTGACCTATGGGAAAG
3[116]	GAAAGTGTGGTTACGCCTGGACTCCGGTATGTTTAT

5[116]	GAACCATTTATTATCCAGCCCGCAGGTTGTTACGTC
7[116]	GAAGACGGCTACTGAGATCCCAACAGATGTTTGATG
9[116]	GAAGCACCCACACTGTGAGTCATTTGCATTTACTCC
11[116]	GACTGCCAGTCTCAAGAATAATTGAATCCGGACCAG
13[116]	GAGCTAATAGAACTGAGTACGTCTCGACGGTTCACT
15[116]	GATCGTATGGTATATGACCCGTCGTCACGGTACAAT
17[116]	GCATGACGGTCTACTTGGTTATCGCTCTAAACTCAG
1[134]	ATGTTATTCGTCGTGGGACGCTAAAACACTCTATGG
3[134]	GCACGAAGCAGCTGACTATTTGACGATCTTCAATTG
5[134]	GTCTTAGGAACGGAGGTCTCAGGAACTGGTCATTAA
7[134]	GACCGAGAGTCAATGGAGACTAATGTTTACGCGTTT
9[134]	GTCTCAATCGCTTTAGAGTCCGAATCCGTTACGATC
11[134]	TTCAGTACATCTCTTCGCGAACTTATCGCGCTAATC
13[134]	CGACCTCGTATTTCTCAAATCGGTTGACTTCCTTGT
15[134]	TCCCAATTGAAATCTAATGGACAAACTGTAGCGGCA
17[134]	AGTTAATTCGCGACCCGGTTAAAACGATTGACAAGT
	**SSTs-a***
4[90]	AAGCGATGCGGCACGAACGAAAGTGGGCACGACATA
8[90]	ATTCAAATCAACGATTACCACTGTAGGTTATTGTAT
10[37]	AGGGAAGTTGTACAAAGATTCATAAGAAGACGCTGA
14[37]	ACGCACCTAAAGTTATAGCCAATAAGCCCGAGGCTT
12[125]	AGTTTGTCCAGTGAACCGGCCGTCTTCCTCCATTGA
14[72]	CACTCAATATAAAGATGCGTTGGTTCCGCTCTCAGA
4[107]	ACCTGCGGGAGCTGAGTATCGAAATCGGGTACACCG
8[107]	GCAAATGACGTTCGGCTACTTGCTAGGTTCTTGAGA
10[55]	ATTAGTGATCTGCTAAGTATAGGCCGTCATTCGGGA
6[37]	ATAAGGACCCTTACGCCATAGATGGACCACTAATAC
14[90]	ACCTCCGCTAGTTTAGGGCTGGGTACGTTACGGGCA
4[125]	GTTTTAGCGGACGTAACACCGTCATGCCCGGGTCGC
8[125]	GATAAGTTCGGAGTAAATCACACTTTCTAGTCAGCT
10[72]	TCCTTCTATGCCTTCAAGAACATGCGACGGAACTGT
6[107]	TCTGTTGGGACCGTAAACCGCTGTCCCTCACAGTGT
6[55]	GTGTGTGATCCAGTGGTGTTACTCTCTACGTATCTG
14[107]	TGACGACGGGTATAGAACTTTACCTGCCCAAGTAGA
10[90]	ACGCGAGGGTGATATGCTACCCGATCGTACGGTAGA
6[72]	CAGGTATGGTTACTTGTATGAGCGCGATTAACAGCA
14[125]	TCGTTTTAAATTGTACCGGGGTGCTTCCTCTAAAGC
10[107]	ATTCAATTATTAGTAGATGAGGGATAGATCTCAGTA
12[37]	CAGCTAGACGACCGAAATCGATGTATGGCGGATACT
12[55]	TTGCTGTACTCGGCTTATAGTACAACTAACTGAACA
6[125]	GGATTCGGACATCAAACAATTCCTTTCTCCCACGAC
10[125]	AACATTAGTCTGGTCCGGAAATGGTTCGACCTCCGT
12[72]	TCTATATGGCATTGGAGTGAGGTACCAAACGGTCAA
4[55]	TTATTCGGGGCCTAGGGAGGGTAGTTTATGTAAGAT
0[37]	GACTATCTTCGTTGATAGCGCTGCTACACATTTGGA
12[90]	TACACAATACATGAACTTGATGCACCGCCCTCGGAG
0[55]	TCCAATATGTCGGACTTACCGTGTATTACATTTGAT
6[90]	CTTACCGATAAATCGATTGACGCTCGGTGGTCCCGT
12[107]	TCGAGACGTGAGATACCCTATAGCCACGTCATATAC
0[72]	GAGCCGTCGGATCCAGAGACGTAATGGTAGGTTTCA
16[107]	GAGCGATAACACTGTGTGATGGACTGCACTCAGTTC

4[72]	CCTTTAAGGACAGTACTGTTGATCCTGCCTCCCAGT
0[90]	ATTATCCGACACTTTAGTACCTATCTGCCGACATAA
2[37]	CAGGGATATAACTGATGTGAGTCCATAAGGTAGTCA
0[107]	AGGTCACATCAGAATAAAACAGCTTGGAGGCGTAAC
2[55]	GTAGTTTGGTACAGGTGTGTGGAAGGTTATAAGGCA
14[55]	GCGTTTGTTGGGAATTACGGTAAGTATTAATGTCTC
0[125]	ATCGTCAAACTTTCCCATTATTAGCTCTTGAGAAAT
2[72]	GCTCCATTTGTACCGTATCTTGCAGAAGTTGAGGGA
16[37]	TTACTGTTTGGAGTTTGCAATAGCACAGTTCTGAAG
2[90]	CATAGTCCTTATACCGAACGATACTGGAAGCCTATC
16[55]	ATGCAATGCCTACGGCCTCTACTGGCTACATTGGCT
8[37]	AGTGTGAGCACAATCAAATTCTAACTGCAAAGCCAC
16[90]	AATGACACTGAGGTTCACTGTGTCAAGTTGACTAGA
2[107]	CCGGAGTCCGGATGTTGTCTACTGTACCTGGATAAT
16[72]	ACTAAGAAGTTCGGGAGTCTAACTTCACAGATGGGC
8[55]	CCCATACTAGTTGGAATCAGTAGTGCTCGCGACTTC
4[37]	ATGTGGGATTTAATTCAAGGAAGAAGGACTGACCTT
2[125]	AGTTCCTGAATAAACATACATACGATCATTAGATTT
8[72]	ATCTTAGATTTTCGTTTCCCGGAGGTTATACGTAAA
16[125]	TCAACCGATCTGAGTTTACTGGCAGTCGCGAAGAGA
0[143]	TTTTTTTTTCAATTGAAGGAATAACATTTTTTTTTT
0[20]	ACGACACTATTTTTTTTTTTTTTTTTTTAAAGATGTT
2[143]	TTTTTTTTTTTAATGACCGCTTCGTGCTTTTTTTTT
2[20]	GTCTTTACATTTTTTTTTTTTTTTTTTCCAGTCTTT
4[143]	TTTTTTTTTCCATAGAGTTCCTAAGACTTTTTTTTT
4[20]	TACGCGAATTTTTTTTTTTTTTTTTTTTAAACGACTG
6[143]	TTTTTTTTTGATCGTAACCTCTCGGTCTTTTTTTTT
6[20]	TCCTCCACGTTTTTTTTTTTTTTTTTTCCATGGTAA
8[143]	TTTTTTTTTGATTAGCGCGATTGAGACTTTTTTTTT
8[20]	AGTCCTCGCTTTTTTTTTTTTTTTTTTACTTTCGCA
10[143]	TTTTTTTTTAAACGCGTATGTACTGAATTTTTTTTT
10[20]	GTACAGAAATTTTTTTTTTTTTTTTTTTCGGATACGA
12[143]	TTTTTTTTTTGCCGCTACACGAGGTCGTTTTTTTTT
12[20]	TGGTGAGAATTTTTTTTTTTTTTTTTTTCCCGGACAG
14[143]	TTTTTTTTTACTTGTCAACAATTGGGATTTTTTTTT
14[20]	GTACGCGACTTTTTTTTTTTTTTTTTTTCATAAACAA
16[143]	TTTTTTTTTACAAGGAAGGAATTAACTTTTTTTTTT
16[20]	AGGTCGTACTTTTTTTTTTTTTTTTTTTCTCCTGTCA

Additional References

1 Kim, T., Lee, C., Lee, J. Y. & Kim, D.-N. Controlling chiroptical responses via chemo-mechanical deformation of DNA origami structures. *ACS Nano* **18**, 3414-3423 (2024).

2 Jie, S., Wei, S., Di, L., Thomas, S. & Peng, Y. Three-dimensional nanolithography guided by DNA modular epitaxy. *Nat. Mater.* **20**, 683-690 (2021).

3 Ke, Y. G. *et al.* Multilayer DNA origami packed on a square lattice. *J. Am. Chem. Soc.* **131**, 15903-15908 (2009).

4 Lee, J. Y. *et al.* Rapid computational analysis of DNA origami assemblies at near-atomic resolution. *ACS Nano* **15**, 1002-1015 (2021).

5 Ke, Y. G., Ong, L. L., Shih, W. M. & Yin, P. Three-dimensional structures self-assembled from DNA bricks. *Science* **338**, 1177-1183 (2012).

6 Karthika, S., Radhakrishnan, T. K. & Kalaichelvi, P. A review of classical and nonclassical nucleation theories. *Cryst. Growth Des.* **16**, 6663-6681 (2016).

7 Ong, L. L. *et al.* Programmable self-assembly of three-dimensional nanostructures from 10,000 unique components. *Nature* **552**, 72-77 (2017).
